# Identification of *GGT5* as a Novel Prognostic Biomarker for Gastric Cancer and its Correlation With Immune Cell Infiltration

**DOI:** 10.3389/fgene.2022.810292

**Published:** 2022-03-18

**Authors:** Yuli Wang, Yuan Fang, Fanchen Zhao, Jiefei Gu, Xiang Lv, Rongzhong Xu, Bo Zhang, Zhihong Fang, Yan Li

**Affiliations:** ^1^ Department of Oncology II, Shanghai Municipal Hospital of Traditional Chinese Medicine, Shanghai University of Traditional Chinese Medicine, Shanghai, China; ^2^ Information Center, Shanghai Municipal Hospital of Traditional Chinese Medicine, Shanghai University of Traditional Chinese Medicine, Shanghai, China; ^3^ Department of Oncology I, Shanghai Municipal Hospital of Traditional Chinese Medicine, Shanghai University of Traditional Chinese Medicine, Shanghai, China

**Keywords:** bioinformatics, GGT5, gastric cancer, immune infiltration, prognostic biomarker, TCGA database, gene expression

## Abstract

Gastric cancer (GC) is a common malignant tumor of the digestive system. Recent studies revealed that high gamma-glutamyl-transferase 5 (*GGT5*) expression was associated with a poor prognosis of gastric cancer patients. In the present study, we aimed to confirm the expression and prognostic value of *GGT5* and its correlation with immune cell infiltration in gastric cancer. First, we compared the differential expression of *GGT5* between gastric cancer tissues and normal gastric mucosa in the cancer genome atlas (TCGA) and GEO NCBI databases using the most widely available data. Then, the Kaplan-Meier method, Cox regression, and univariate logistic regression were applied to explore the relationships between *GGT5* and clinical characteristics. We also investigated the correlation of *GGT5* with immune cell infiltration, immune-related genes, and immune checkpoint genes. Finally, we estimated enrichment of gene ontologies categories and relevant signaling pathways using GO annotations, KEGG, and GSEA pathway data. The results showed that *GGT5* was upregulated in gastric cancer tissues compared to normal tissues. High *GGT5* expression was significantly associated with T stage, histological type, and histologic grade (*p* < 0.05). Moreover, gastric cancer patients with high *GGT5* expression showed worse 10-years overall survival (*p* = 0.008) and progression-free intervals (*p* = 0.006) than those with low *GGT5* expression. Multivariate analysis suggested that high expression of *GGT5* was an independent risk factor related to the worse overall survival of gastric cancer patients. A nomogram model for predicting the overall survival of GC was constructed and computationally validated. *GGT5* expression was positively correlated with the infiltration of natural killer cells, macrophages, and dendritic cells but negatively correlated with Th17 infiltration. Additionally, we found that *GGT5* was positively co-expressed with immune-related genes and immune checkpoint genes. Functional analysis revealed that differentially expressed genes relative to *GGT5* were mainly involved in the biological processes of immune and inflammatory responses. In conclusion, *GGT5* may serve as a promising prognostic biomarker and a potential immunological therapeutic target for GC, since it is associated with immune cell infiltration in the tumor microenvironment.

## Introduction

Gastric cancer (GC) is one of the most common digestive malignancies, ranking fifth in tumor morbidity and fourth in mortality worldwide (Sung et al., 2021). In recent years, with the increasing popularity of screening gastroscopy and surgical intervention, there has been a decline in the incidence and mortality of non-cardia gastric cancer. However, the progressively increased incidence of early-onset GC with more aggressive features is garnering attention and warrants deeper investigation (Bergquist et al., 2019). Since inhibitors of human epidermal growth factor receptor 2 (HER2), such as trastuzumab, pertuzumab, and lapatinib, were introduced for the treatment of HER2-overexpressing GC, the outcome of advanced gastric cancer patients has been significantly improved (Bang et al., 2010). Nevertheless, previous studies have indicated that the majority of gastric cancer patients suffer acquired resistance to trastuzumab within a relatively short period (Zhang et al., 2019). In addition, GC is a highly heterogeneous tumor, and HER2 positivity is found in only approximately 13.0%–22.0% of GC cases (Cappellesso et al., 2015; Van Cutsem et al., 2015; Abrahao-Machado and Scapulatempo-Neto, 2016). There is still a lack of specific biomarkers for early diagnosis or use as potential therapeutic targets for GC. Hence, there is a great need to identify potential prognostic biomarkers or therapeutic targets to improve the survival of gastric cancer patients.

As a crucial liver enzyme involved in extracellular glutathione metabolism, gamma-glutamyl transferase can cleave glutathione peptides to maintain the glutathione balance in the human body (Heisterkamp et al., 2008). Gamma-glutamyltransferase 5 (*GGT5*) is one of the two GGT family members (*GGT1* and *GGT5*) with catalytic activity identified to date and was first reported in detail in 2008 (Heisterkamp et al., 2008). *GGT5* is widely distributed in a variety of tissues, with relatively high expression in liver, kidney, and alveolar macrophages (Hanigan et al., 2015). Functional analyses showed that *GGT5* played a pivotal role in oxidative regulation, drug metabolism, and immune modulation in the human body (Wickham et al., 2011).

Recent studies have demonstrated that upregulated *GGT5* is correlated with tumorigenesis and the progression of a variety of malignancies, including GC (Ren et al., 2020; Wei et al., 2020; Wen et al., 2020; Wu et al., 2021; Ye et al., 2021). Three bioinformatics studies based on the clinical gastric cancer samples retrieved from the Gene Expression Omnibus (GEO) and The Cancer Genome Atlas (TCGA) databases indicated that *GGT5* was included in prognostic gene signatures, and overexpression of *GGT5* was inversely correlated with the survival in GC patients (Wei et al., 2020; Wen et al., 2020; Ye et al., 2021). It was presumed that the underlying mechanism by which *GGT5* affects the prognosis of GC patients might be associated with metabolic regulation, immune modulation, and antioxidant effects (Wei et al., 2020; Wen et al., 2020; Ye et al., 2021). These findings indicated that *GGT5* might serve as a promising prognostic biomarker or potential therapeutic target for gastric cancer. However, the correlation of *GGT5* expression levels with immune cell infiltration in the tumor microenvironment of gastric cancer has not yet been investigated yet.

In the present study, integrated bioinformatics analysis was carried out based on RNA sequencing data retrieved from the TCGA (https://www.cancer.gov/tcga/) and validated in the GEO (https://www.ncbi.nlm.nih.gov/geo/) (Barrett et al., 2012) database. We first compared the differential expression levels of *GGT5* between GC tissues and normal gastric mucosa in the TCGA database and simultaneously validated them in the other two independent RNA profiles, GSE54129 (https://www.ncbi.nlm.nih.gov/geo/download/?acc=GSE54129) and GSE29272 (Wang et al., 2013; Cheng et al., 2017) from the GEO database. Subsequently, we identified the differentially expressed genes (DEGs) between the *GGT5*-high and *GGT5*-low expression groups. The Kaplan-Meier method, Cox regression, and univariate logistic regression (Zhu C. et al., 2021) were performed to investigate the relationships between *GGT5* and clinical characteristics in gastric cancer patients. A nomogram model for predicting overall survival was constructed and computationally validated. Moreover, single-sample gene set enrichment analysis (ssGSEA) (Bindea et al., 2013) was employed to analyze the correlation of *GGT5* expression with infiltration patterns for 24 immune cell types in GC samples. We also explored the correlation of *GGT5* expression with immune-related genes and immune checkpoint genes to further understand the underlying mechanism by which *GGT5* is correlated with immune cell infiltration in gastric cancer. Finally, gene ontology annotation, Kyoto Encyclopedia of Genes and Genomes (KEGG), and Gene Set Enrichment Analysis (GSEA) (Chen et al., 2022) were applied to explore the potential functions of *GGT5* in gastric cancer.

## Materials and Methods

### Data Acquisition and Preprocessing

High-throughput sequencing data (HTSeq) with clinical information, including 375 GC tissues and 32 adjacent normal tissues were downloaded from the TCGA database (https://www.cancer.gov/tcga/). The Fragments Per kilobase Million (FPKM) data were quantified in transcripts per million (TPM). All FPKM values were then log-transformed to obtain a normal distribution with log2(FPKM+1) for further statistical analyses. Similarly, the raw values of the microarray expression data were downloaded from the GEO database. Two datasets, GSE54129 and GSE29272, were ultimately screened out according to the following inclusion criteria: (1) achievable comparison of gastric cancer tissues with normal gastric mucosa limited to *Homo sapiens*; (2) no medical intervention (chemotherapy, radiotherapy, and/or targeted therapy) before sample collection; and (3) more than 100 samples in the full study, and the raw gene expression data can be downloaded in CEL format for further analysis. Subsequently, the data were log2 transformed and quantile normalized using R software (version 3.6.3). The probe ID for each gene was then converted into a gene symbol, and the average expression value was taken while multiple probes were converted to one gene symbol. Moreover, the batch effects were corrected by applying the “remove Batch Effect” function in the “limma” package of R software.

### Differential Expression Analysis of *GGT5* in the TCGA and GEO Databases

We compared the expression level of *GGT5* across cancers and corresponding normal tissues using the Wilcoxon rank-sum test based on the TCGA Pan-Cancer dataset (https://gdc.cancer.gov/about-data/publications/pancanatlas). For gastric cancer, the difference in *GGT5* between tumor tissues and normal tissues was evaluated in TCGA and the Human Protein Atlas (HPA) (Access date: August 2021, https://www.proteinatlas.org/) databases. Additionally, we utilized the area under the ROC curve using R software (version 3.6.3) to assess the predictive value of *GGT5* in distinguishing gastric cancer tissues and normal gastric tissues. Two RNA expression datasets, GSE54129 (https://www.ncbi.nlm.nih.gov/geo/download/?acc=GSE54129) and GSE29272 (Wang et al., 2013; Cheng et al., 2017), retrieved from the GEO database were used as the external validation cohorts.

### Identification of Differentially Expressed Genes Relative to *GGT5*

Gastric cancer tissue samples were classified into high and low expression groups by median value splitting. The identification of DEGs between the *GGT5*-high and *GGT5*-low expression groups was performed by Wald’s test with the R package DESeq2 (version 1.26.0) (Love et al., 2014). The screening threshold for statistical significance was set as absolute log2-fold change (FC) > 2 and adjusted *p value* < 0.05. The upregulated and downregulated DEGs were visualized using volcano plots, and *GGT5*-related DEGs were displayed in a heatmap plot.

### The Relationship Between *GGT5* Expression and the Clinical Characteristics of Patients With Gastric Cancer

First, we determined the cut-off of *GGT5* expression according to its median value. To investigate the relationship between *GGT5* expression and the clinical characteristics of gastric cancer patients, we used univariate logistic regression analysis. The 10-years overall survival (OS), progression-free interval (PFI), and disease-specific survival (DSS) in the *GGT5*-high and *GGT5*-low groups were compared using the Kaplan–Meier curve and the log-rank test (Huang X. et al., 2021). Univariate and multivariate Cox regression analyses based on *GGT5* expression and clinical characteristics were applied to screen the independent survival risk factors for gastric cancer. Additionally, subgroup analysis was conducted and displayed in the forest plots by R software (version 3.6.3). The forest plots show the hazard ratio (HR) and 95% confidence interval of the prognostic factors by univariate and multivariate analyses. All *p value*s less than 0.05 were considered statistically significant.

### Construction and Evaluation of a Prognostic Model for Gastric Cancer Patients

Based on the results of univariate and multivariate Cox regression analyses, prognostic factors were included to build a nomogram model, which aimed to predict the overall survival of gastric cancer patients at 1, 2, and 3 years. The nomogram analysis was carried out using the “RMS” package in R software (version 3.6.3). The nomogram was assessed graphically by plotting the calibration curves, which compared the observed values (Kaplan-Meier method) with the nomogram-predicted probabilities. For a well-calibrated model, the scatter points of the nomogram prediction model will fall on a 45-degree diagonal line (Balachandran et al., 2015). Furthermore, the Harrell concordance index (C-index) was also employed to evaluate the overall predictive ability of the nomogram model. The value of the C-index ranges from 0.5 to 1, and the higher the C-index, the better the prediction performance. The significance level for this study was set at 0.05, and all statistical tests were two-tailed.

### The Correlation of *GGT5* Expression With Immune Cell Infiltration and Immune-Related Genes

To explore the associations between *GGT5* expression and immune cell infiltration, we applied the ssGSEA algorithm using the R package ‘GSVA’ (version 1.34.0) (Bindea et al., 2013) to investigate the immune infiltration landscape of 24 different immune cells in both the *GGT5*-high and *GGT5*-low groups. The Pearson correlation coefficients were calculated to determine the relationship between *GGT5* expression and infiltrating immune cells. Representative immune-infiltrating cells with significant correlations are displayed in scatter plots and column bar graphs. Moreover, to further understand the potential mechanisms by which *GGT5* is correlated with immune cell infiltration, we also assessed the correlation of *GGT5* expression with immune-related genes, including MHC genes, immune activation genes, immunosuppressive genes, chemokine receptors, and chemokines (Zhu H. et al., 2021).

### Immune Checkpoint Analysis

The expression level of immune checkpoint biomarkers was closely related to the therapeutic response to immune checkpoint blockade treatment. Therefore, the present study focused on eight critical immune checkpoint genes, including *SIGLEC15*, *TIGIT*, *CD274*, *HAVCR2*, *PDCD1*, *CTLA4*, *LAG3*, and *PDCD1LG2*. Different expression levels of these immune checkpoints were compared between the *GGT5*-high and *GGT5*-low expression groups by heatmap and bar charts. We also applied the R package “immuneeconv” to assess the co-expression of *GGT5* with immune checkpoint biomarkers based on the TCGA database (https://www.cancer.gov/tcga/). Additionally, the TIDE (Jiang et al., 2018) algorithm was employed to predict the potential response to immune checkpoint blockade therapy.

### Functional Analyses of *GGT5* in Gastric Cancer

Before performing gene functional enrichment analysis, we transformed the gene symbols into EnterzID and obtained GO and KEGG signaling pathway annotations with R software. To further understand the functions of *GGT5* in gastric cancer, the clusterProfiler (version 3.14.3) package (Yu et al., 2012) was utilized to carry out GO functional enrichment analysis and it displayed the three aspects of GO enrichment, including biological processes, cellular components, and molecular functions. Enrichment analysis of DEGs was also conducted with the clusterProfiler package, and the significant DEG-related signaling pathways were mapped into a bubble graph. For GSEA, based on the mean expression of *GGT5*, the candidate genes were divided into the *GGT5*-high group and the *GGT5*-low group. Functional predefined gene sets were obtained from the Molecular Signatures database, MSigDB (https://www.gsea-msigdb.org/gsea/msigdb). The candidate genes involved in the pathway with the screening criteria of *p* < 0.05 and false discovery rate (FDR) < 0.25 were considered significantly enriched. The normalized enrichment score and adjusted *p value* were applied to select the significantly enriched signaling pathways.

## Results

### The Expression Level of *GGT5* was Elevated in Human Gastric Tissues

**FIGURE 1:**
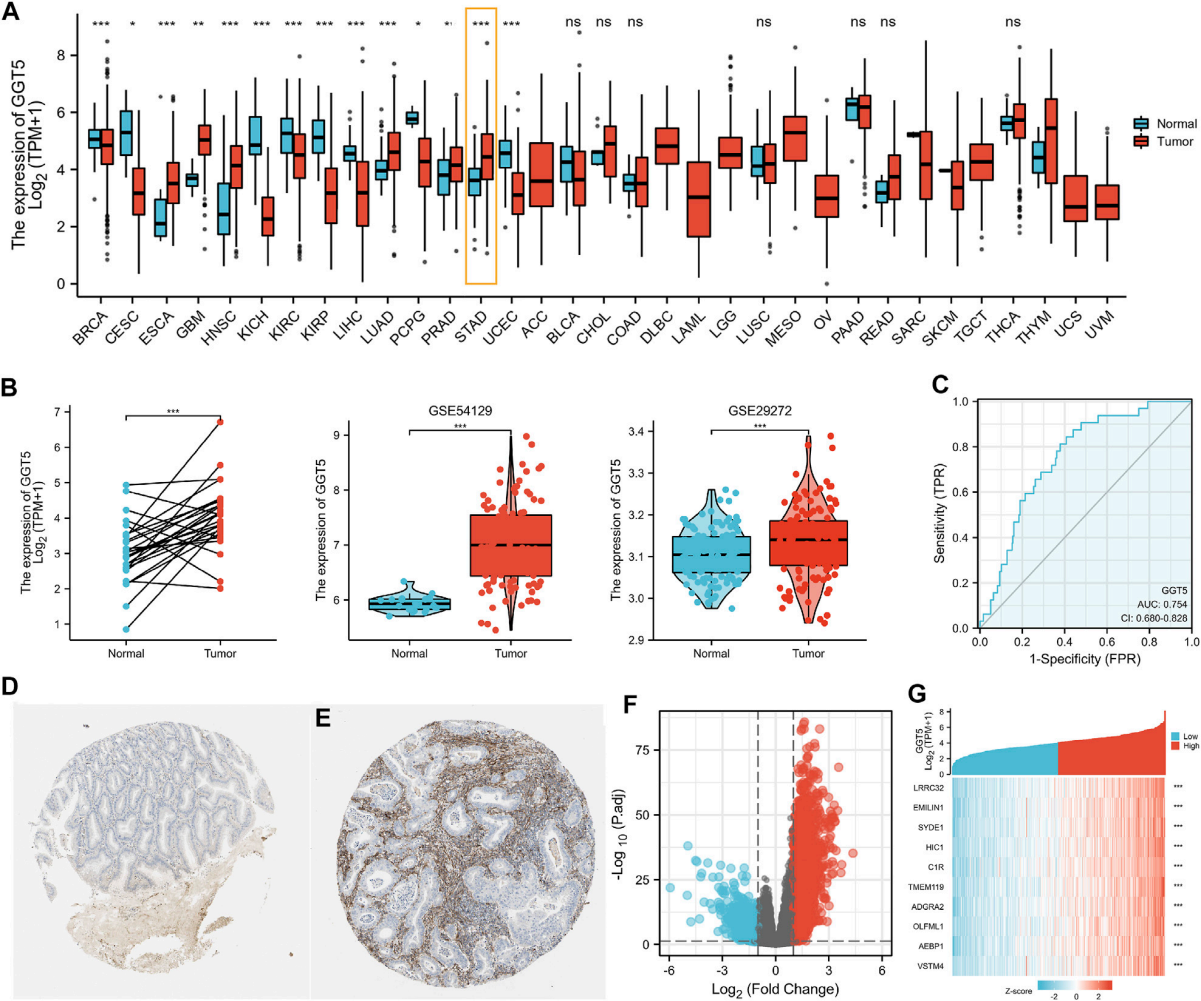
Pan-cancer analysis of *GGT5* expression levels and upregulated *GGT5* in human gastric cancer. **(A)** Detection of the expression level of *GGT5* in a pan-cancer dataset from TCGA. **(B)** The expression level of *GGT5* in gastric cancer compared to paired and normal gastric mucosa from the GEO (GSE54129, GSE29272) and TCGA datasets. **(C)** ROC curve showing the predictive value of *GGT5* for identifying gastric cancer tissues based on the TCGA database. **(D,E)** Comparison of *GGT5* between normal gastric mucosa **(D)** and gastric cancer tissue **(E)** based on the Human Protein Atlas database (antibody HPA008121, 10×) (accession date: August 2021, https://www.proteinatlas.org/). **(F)** The volcano plot presents the *GGT5*-related differentially expressed genes (DEGs) based on the TCGA database. Red and blue dots represent up and downregulated genes, respectively. **(G)** The heatmap shows the top 10 DEGs related to *GGT5*. *, *p* < 0.05; **, *p* < 0.01; ***, *p* < 0.001; ns, not significant, *p* > 0.05. ACC: adrenocortical carcinoma; BLCA: bladder urothelial carcinoma; BRCA: breast invasive carcinoma; CESC: cervical squamous cell carcinoma and endocervical adenocarcinoma; CHOL: cholangiocarcinoma; COAD: colon adenocarcinoma; DLBC: lymphoid neoplasm diffuse large B-cell lymphoma; ESCA: esophageal carcinoma; GBM: glioblastoma multiforme; HNSC: head and neck squamous cell carcinoma; KICH: kidney chromophobe; KIRC: kidney renal clear cell carcinoma; KIRP: kidney renal papillary cell carcinoma; LAML: acute myeloid leukemia; LGG: brain lower grade glioma; LIHC: liver hepatocellular carcinoma; LUAD: lung adenocarcinoma; LUSC: lung squamous cell carcinoma; MESO: mesothelioma; OV: ovarian serous cystadenocarcinoma; PAAD: pancreatic adenocarcinoma; PCPG: pheochromocytoma and paraganglioma; PRAD: prostate adenocarcinoma; READ: rectum adenocarcinoma; SARC: sarcoma; SKCM: skin cutaneous melanoma; STAD: stomach adenocarcinoma; TGCT: testicular germ cell tumors; THCA: thyroid carcinoma; THYM: thymoma; UCEC: uterine corpus endometrial carcinoma; UCS: uterine carcinosarcoma; UVM: uveal melanoma.

We first investigated *GGT5* expression across cancers in a pan-cancer dataset from TCGA. The results indicated that the expression level of *GGT5* was significantly elevated in esophageal carcinoma (ESCA), glioblastoma multiforme (GBM), head and neck squamous cell carcinoma (HNSC), lung adenocarcinoma (LUAD), prostate adenocarcinoma (PRAD), and stomach adenocarcinoma (STAD) (*p* < 0.05). In contrast, significantly decreased *GGT5* expression was noticed in invasive breast carcinoma (BRCA), cervical squamous cell carcinoma and endocervical adenocarcinoma (CESC), kidney chromophobe (KICH), kidney renal clear cell carcinoma (KIRC), kidney renal papillary cell carcinoma (KIRP), liver hepatocellular carcinoma (LIHC), and uterine corpus endometrial carcinoma (UCEC) (*p* < 0.05) (Figure 1A). There was a large degree of heterogeneity across cancers, and only a few of the tumor types, including gastric cancer, had high *GGT5* expression levels.

Next, we compared the expression level of *GGT5* between gastric cancer tissues and adjacent normal tissues using the TCGA database and further validated it using related array data (GSE54129, GSE29272) from the GEO database. All of the results indicated that *GGT5* expression levels were elevated in gastric cancer tissues compared with normal tissues (*p* < 0.05) (Figure 1B, Supplementary Table S1). The ROC curve was plotted to determine the sensitivity and specificity of *GGT5* to distinguish gastric cancer tissues from normal gastric mucosa. The results showed that the area under the ROC curve was 0.754, which indicated that *GGT5* might potentially contribute to the identification of gastric cancer tissues (Figure 1C). Similar results were also obtained in the validation cohorts of two GEO datasets, as shown in Supplementary Figures S1A, S2A. Furthermore, the protein level of *GGT5* was also found to be higher in gastric cancer tissue than that in normal tissues in the HPA database (Figures 1D,E).

### Identification of Differentially Expressed Genes in Gastric Cancer

According to the gene expression level of *GGT5*, the TCGA stomach adenocarcinoma samples (https://www.cancer.gov/tcga/) were stratified into a high-expression group (*N* = 203) and a low-expression group (*N* = 204) with a total number of uni-genes of 56,493 (Supplementary Table S2). Finally, a total of 330 DEGs were screened out with the criteria of |log2(FC)|>2 and adjusted *p value* < 0.05, including 216 upregulated genes and 114 downregulated genes (Figure 1F, Supplementary Table S3). The HTSeq-Count data (Wang et al., 2013; Cheng et al., 2017) were further analyzed using the DESeq2 package in R software. The heatmap showed the top 10 DEGs that were most closely related to *GGT5* expression (Figure 1G). Similarly, 507 DEGs, including 304 upregulated genes and 203 downregulated genes were identified from GSE54129 (Supplementary Figures S1B,C and Supplementary Table S4), and a total of 38 DEGs, including 37 upregulated genes and one downregulated gene, were detected from GSE29272 (Supplementary Figures S2B,C and Supplementary Table S5).

### Correlation Between *GGT5* Expression Level and the Clinicopathological Features of Gastric Cancer Patients

**FIGURE 2:**
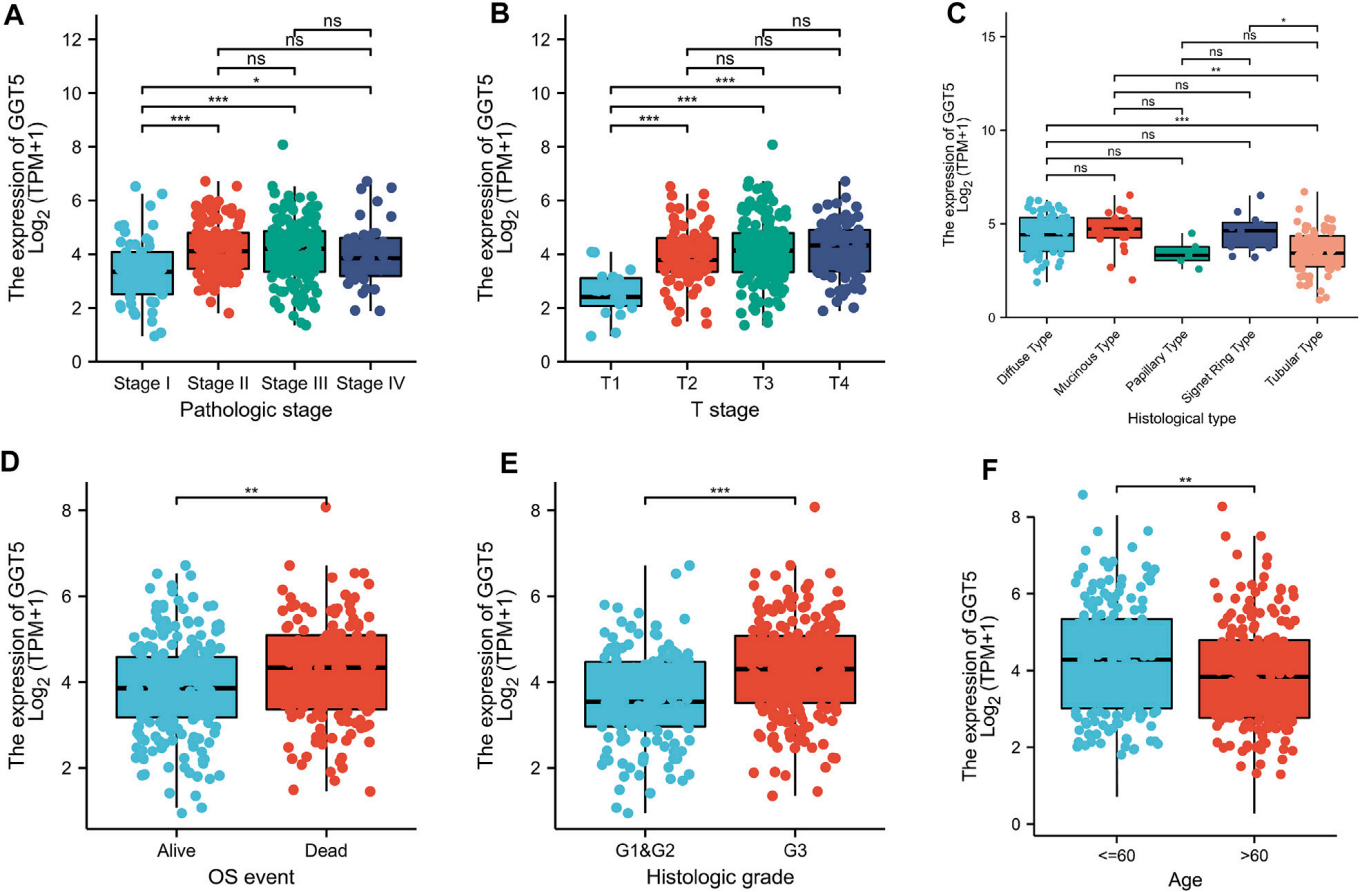
Correlation between *GGT5* expression level and the clinicopathological features of gastric cancer patients for **(A)** pathologic stage, **(B)** T stage, **(C)** histological type, **(D)** OS event, **(E)** histologic grade, and **(F)** age. *, *p* < 0.05; **, *p* < 0.01; ***, *p* < 0.001; ns, not significant, *p* > 0.05.

To define the clinical correlation between the *GGT5* expression level and the clinicopathological features in gastric cancer, we further analyzed the differences in clinical characteristics between the *GGT5*-high and *GGT5*-low expression groups based on the TCGA database. After removing duplicates, a total of 375 patients, including 241 (64.3%) men and 134 (35.7%) women, were included in the analysis, as displayed in Table 1. Our results revealed that *GGT5* overexpression was closely correlated with the pathological stage (Stage II & Stage III & Stage IV vs. Stage I, *p* < 0.05), T stage (T2–T4 vs. T1, *p* < 0.001), histological type (diffuse type and mucinous type and signet ring type vs. tubular type, *p* < 0.05), overall survival status (alive vs. dead, *p* < 0.01), histologic grade (G3 vs. G1 & G2, *p* < 0.001), and age (less than or equal to 60 vs. greater than 60, *p* < 0.01) (Figures 2A–F). Nevertheless, the expression level of *GGT5* had no significant correlation with M stage, N stage, or residual tumor (*p* > 0.05). Furthermore, the univariate logistic regression analysis indicated that the expression level of *GGT5* was closely associated with the clinical characteristics of a poor prognosis in patients with gastric cancer (Table 2). Specifically, *GGT5* was positively correlated with the T stage (T3&T4 vs. T1&T2, OR = 1.437, 95% CI: 1.167–1.783, *p* < 0.001), histologic grade (G3 vs. G1&G2, OR = 1.738, 95% CI: 1.420–2.151, *p* < 0.001), and histological type (tubular type vs. not otherwise specified, OR = 0.658, 95% CI: 0.505–0.847, *p* = 0.001). Collectively, *GGT5*-high expression gastric cancers were associated with a relatively higher pathological stage (TNM stage and T stage) and histological grade. All of the above results suggested that upregulated *GGT5* was strongly correlated with a poor tumor differentiation grade and a poor prognosis of gastric cancer.

**TABLE 1:** Clinical characteristics of gastric cancer patients in the *GGT5*-high and the *GGT5*-low expression groups.

Characteristic	Levels	Low expression of *GGT5*	High expression of *GGT5*	*p*	Method
*n*		187	188		
T stage, n (%)	T1	17 (4.6%)	2 (0.5%)	<0.001	Chi-square
	T2	46 (12.5%)	34 (9.3%)		
	T3	75 (20.4%)	93 (25.3%)		
	T4	45 (12.3%)	55 (15.0%)		
N stage, n (%)	N0	59 (16.5%)	52 (14.6%)	0.135	Chi-square
	N1	50 (14.0%)	47 (13.2%)		
	N2	41 (11.5%)	34 (9.5%)		
	N3	28 (7.8%)	46 (12.9%)		
M stage, n (%)	M0	166 (46.8%)	164 (46.2%)	1.000	Chi-square
	M1	13 (3.7%)	12 (3.4%)		
Pathologic stage, n (%)	Stage I	38 (10.8%)	15 (4.3%)	0.003	Chi-square
	Stage II	50 (14.2%)	61 (17.3%)		
	Stage III	67 (19.0%)	83 (23.6%)		
	Stage IV	22 (6.2%)	16 (4.5%)		
Primary therapy outcome, n (%)	PD	33 (10.4%)	32 (10.1%)	0.764	Fisher’s test
	SD	7 (2.2%)	10 (3.2%)		
	PR	1 (0.3%)	3 (0.9%)		
	CR	111 (35.0%)	120 (37.9%)		
Gender, n (%)	Female	66 (17.6%)	68 (18.1%)	0.945	Chi-square
	Male	121 (32.3%)	120 (32.0%)		
Race, n (%)	Asian	43 (13.3%)	31 (9.6%)	<0.001	Chi-square
	Black or African American	10 (3.1%)	1 (0.3%)		
	White	97 (30.0%)	141 (43.7%)		
Age, n (%)	≤65	74 (19.9%)	90 (24.3%)	0.153	Chi-square
	>65	110 (29.6%)	97 (26.1%)		
Histological type, n (%)	Diffuse Type	26 (7.0%)	37 (9.9%)	0.005	Chi-square
	Mucinous Type	4 (1.1%)	15 (4.0%)		
	Not Otherwise, Specified	103 (27.5%)	104 (27.8%)		
	Papillary Type	4 (1.1%)	1 (0.3%)		
	Signet Ring Type	5 (1.3%)	6 (1.6%)		
	Tubular Type	45 (12.0%)	24 (6.4%)		
Residual tumor, n (%)	R0	150 (45.6%)	148 (45.0%)	0.262	Chi-square
	R1	5 (1.5%)	10 (3.0%)		
	R2	10 (3.0%)	6 (1.8%)		
Histologic grade, n (%)	G1	6 (1.6%)	4 (1.1%)	<0.001	Fisher’s test
	G2	89 (24.3%)	48 (13.1%)		
	G3	87 (23.8%)	132 (36.1%)		
Anatomic neoplasm subdivision, n (%)	Antrum/Distal	73 (20.2%)	65 (18.0%)	0.054	Fisher’s test
	Cardia/Proximal	22 (6.1%)	26 (7.2%)		
	Fundus/Body	56 (15.5%)	74 (20.5%)		
	Gastroesophageal Junction	25 (6.9%)	16 (4.4%)		
	Other	4 (1.1%)	0 (0%)		
Anti-reflux treatment, n (%)	No	79 (44.1%)	63 (35.2%)	0.386	Chi-square
	Yes	17 (9.5%)	20 (11.2%)		
Reflux history, n (%)	No	92 (43.0%)	83 (38.8%)	0.258	Chi-square
	Yes	25 (11.7%)	14 (6.5%)		
*H pylori* infection, n (%)	No	90 (55.2%)	55 (33.7%)	0.464	Chi-square
	Yes	9 (5.5%)	9 (5.5%)		
Barrett’s esophagus, n (%)	No	111 (53.4%)	82 (39.4%)	0.152	Chi-square
	Yes	12 (5.8%)	3 (1.4%)		
OS event, n (%)	Alive	122 (32.5%)	106 (28.3%)	0.099	Chi-square
	Dead	65 (17.3%)	82 (21.9%)		
DSS event, n (%)	Alive	135 (38.1%)	128 (36.2%)	0.203	Chi-square
	Dead	39 (11.0%)	52 (14.7%)		
PFI event, n (%)	Alive	136 (36.3%)	115 (30.7%)	0.023	Chi-square
	Dead	51 (13.6%)	73 (19.5%)		
Age, median (IQR)		68 (58.75, 74)	66 (57.50, 72)	0.118	Wilcoxon

**TABLE 2:** Univariate logistic regression assesses the relationships between *GGT5* and clinical characteristics of gastric cancer patients.

Characteristics	Total (*n*)	Odds ratio (OR)	*p-* value
T stage (T3&T4 vs. T1&T2)	367	1.437 (1.167–1.783)	<0.001
Histological type (Tubular Type vs. Not Otherwise, Specified)	276	0.658 (0.505–0.847)	0.001
Histologic grade (G3 vs. G1&G2)	366	1.738 (1.420–2.151)	<0.001

### *GGT5* Overexpression was Found to be Associated With Poor Outcomes in Gastric Cancer Patients

**FIGURE 3:**
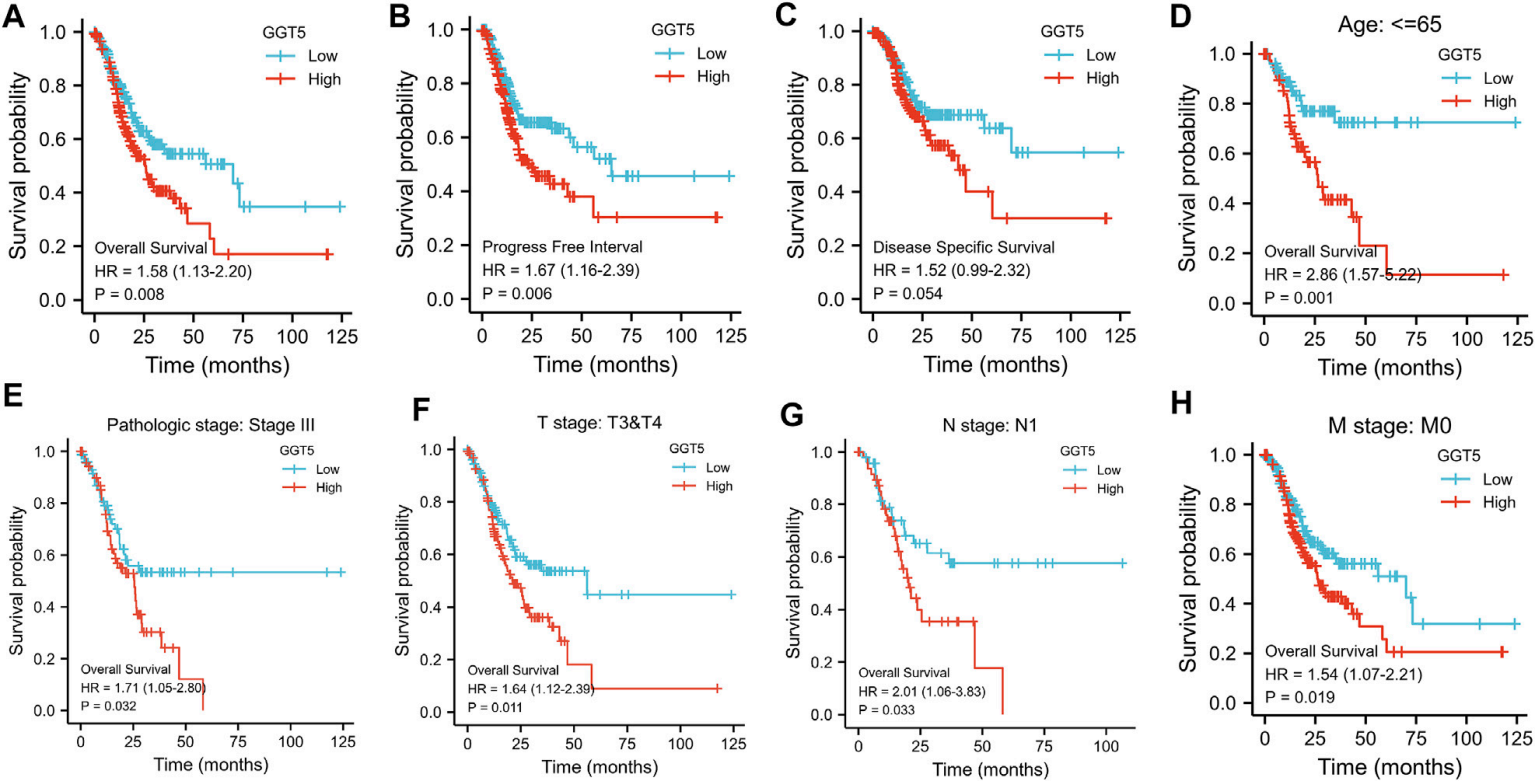
Kaplan-Meier analysis of gastric cancer patients according to *GGT5* expression level and subgroup analysis. The overall survival curves **(A),** progression-free interval curves **(B)**, and disease-specific survival curves **(C)** between the *GGT5*-high and *GGT5*-low expression groups. **(D–H)** Comparison of the overall survival curves in age ≤65, pathologic stage III, T3 & T4 stage, N1 stage, and M0 stage subgroups between the *GGT5*-high and *GGT5*-low expression groups.

To evaluate the prognostic value of *GGT5* in gastric cancer, we calculated the survival of patients with different *GGT5* expression levels using the Kaplan-Meier method. The results indicated that the 10-years overall survival of patients with low *GGT5* expression was better than that of patients with high *GGT5* expression (HR = 1.58, 95% CI: 1.130–2.200, *p* = 0.008, Figure 3A). Similar results were also obtained for the progression-free interval (PFI, HR = 1.67, 95% CI: 1.160–2.390, *p* = 0.006, Figure 3B). Patients with high *GGT5* expression showed a similar prolonged survival trend for disease-specific survival (DSS, HR = 1.52, 95% CI: 0.99–2.32, *p* = 0.054, Figure 3C), but the difference was not statistically significant. Furthermore, the subgroup analysis demonstrated that the overall survival of patients with *GGT5*-high expression showed a significantly poor prognosis in age ≤65, pathologic stage III, T3 & T4 stage, N1 stage, and M0 stage (Figures 3D–H).

**FIGURE 4:**
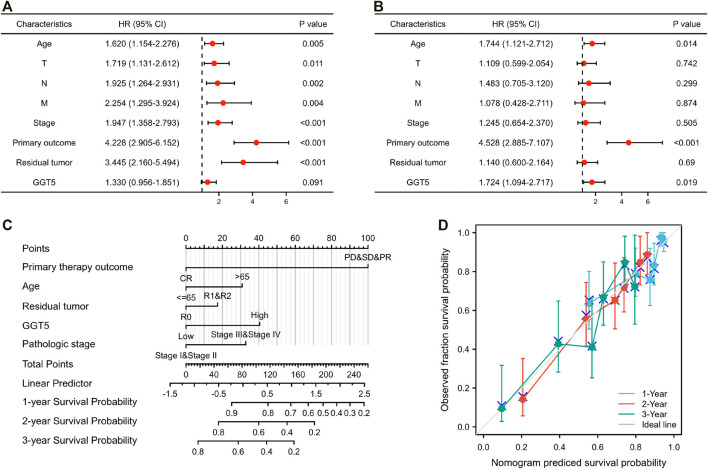
The prognostic factor analysis and nomogram model construction. The results of univariate **(A)** and multivariate Cox regression **(B)** for the overall survival of GC patients are displayed in the form of forest plots. **(C)** A nomogram model was constructed for predicting the 1-year, 2-years, and 3-years overall survival probability of GC patients. **(D)** Calibration curves for the nomogram model predicting the probability of the 1-year, 2-years, and 3-years overall survival of GC patients.

Univariate Cox regression analysis revealed that T stage, N stage, M stage, pathologic stage, primary therapy outcome, residual tumor, and age were closely correlated with the overall survival of the GC patients, as shown in Table 3 and Figure 4A. However, the prognostic value of *GGT5* for GC patients was not statistically significant (HR = 1.330, 95% CI: 0.956–1.851, *p* = 0.091). Further multivariate analyses were carried out to screen independent prognostic factors. The results showed that in addition to the previously mentioned primary therapy outcome (PD & SD & PR vs. CR, HR = 4.528, 95% CI: 2.885–7.107, *p* < 0.001) and age (>65 vs. ≤65, HR = 1.744, 95% CI: 1.121–2.712, *p* = 0.014), high *GGT5* expression was also an independent prognostic factor for worse overall survival in gastric cancer patients (HR = 1.724, 95% CI: 1.094–2.717, *p* = 0.019) (Table 3 and Figure 4B). To summarize, our study revealed that upregulated expression of *GGT5* was correlated with shorter overall survival and progression-free interval of GC patients.

**TABLE 3:** Univariate and multivariate Cox regression analysis of the risk factors for OS in patients with gastric cancer.

Characteristics	Total(N)	Univariate analysis		Multivariate analysis	
		Hazard ratio (95% CI)	*p value*	Hazard ratio (95% CI)	*p value*
T stage (T3&T4 vs. T1&T2)	362	1.719 (1.131–2.612)	**0.011**	1.109 (0.599–2.054)	>0.05
N stage (N1&N2&N3 vs. N0)	352	1.925 (1.264–2.931)	**0.002**	1.483 (0.705–3.120)	>0.05
M stage (M1 vs. M0)	352	2.254 (1.295–3.924)	**0.004**	1.078 (0.428–2.711)	>0.05
Pathologic stage (III & IV vs. I & II)	347	1.947 (1.358–2.793)	**<0.001**	1.245 (0.654–2.370)	>0.05
Primary outcome (PD & SD & PR vs. CR)	313	4.228 (2.905–6.152)	**<0.001**	4.528 (2.885–7.107)	**<0.001**
Residual tumor (R1 & R2 vs. R0)	325	3.445 (2.160–5.494)	**<0.001**	1.140 (0.600–2.164)	>0.05
Age (>65 vs. ≤65)	367	1.620 (1.154–2.276)	**0.005**	1.744 (1.121–2.712)	**0.014**
*GGT5* (High vs. Low)	370	1.330 (0.956–1.851)	>0.05	1.724 (1.094–2.717)	**0.019**

Statistical *p* values < 0.05 are shown in bold.

### Construction and Computational Validation of a Nomogram Model for Gastric Cancer Patients Based on *GGT5*

To establish a novel model for predicting the outcomes of patients with gastric cancer, a nomogram was constructed for overall survival (Figure 4C). The variables of the nomogram were selected according to the results of the univariate and multivariate regression, and a 100-point scale was used to assign a point value to each variable. Each point of the variable was summed, and the total prone score was calculated, ranging from 0 to 240 points. Subsequently, by drawing a vertical line from the total points scale to the survival probability lines, we could obtain the estimated probabilities of 1-year, 2-years, and 3-years overall survival for GC patients.

To evaluate the accuracy and reliability of the nomogram model in predicting survival, the C-index and calibration curve were both used for further computational validation. The results revealed that the C-index of the nomogram model was 0.724 (95% CI: 0.698–0.749), which implied that the novel model was moderately accurate and appropriate for the overall survival prediction of GC patients. Additionally, it was found that the bias-corrected lines of 1 year, 2 years, and 3 years were close to the ideal 45° diagonal line in the calibration plot, which indicated that the theoretical values were in agreement with the observed values (Figure 4D). The above outcomes confirmed that the nomogram model could be applied for predicting the 1-year, 2-years, and 3-years overall survival of patients with gastric cancer.

### Relationship Between *GGT5* Expression and Immune Cell Infiltration in Gastric Cancer

**FIGURE 5:**
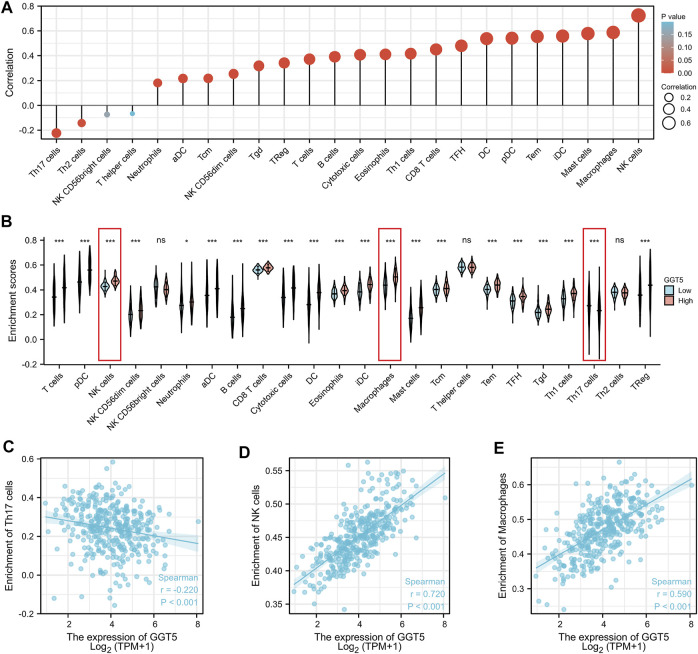
The expression level of *GGT5* was closely associated with the degree of immune cell infiltration in the tumor immune environment. **(A)** Correlation analysis between the expression level of *GGT5* and the degree of immune cell infiltration. **(B)** Comparison of the different immune cells infiltration levels under high and low *GGT5* expression conditions. **(C–E)** The scatter plots show the correlation between *GGT5* expression and the infiltration degrees of Th17 cells, macrophages, and NK cells. *, *p* < 0.05; ***, *p* < 0.001; ns, not significant, *p* > 0.05.

To investigate the association between the expression level of *GGT5* and immune infiltration, we first compared the relationship between *GGT5* expression and the degree of immune cell infiltration. The results indicated that high levels of *GGT5* expression were significantly correlated with the high-level infiltration of the majority of immune cells, including T cells, pDC, NK cells, NK CD56dim cells, neutrophils, aDC, B cells, CD_8_^+^ T cells, cytotoxic cells, DC, eosinophils, iDC, macrophages, mast cells, central memory T-cell (Tcm), effective memory T-cell (Tem), follicular helper T-cell (TFH), T gamma delta (Tgd), Th1 cells, and Treg cells (*p* < 0.05) (Figure 5A). Then, the correlation between *GGT5* expression and the immune cell enrichment was analyzed with the Spearman correlation test and determined by the ssGSEA algorithm. *GGT5* expression was positively correlated with the infiltration of NK cells (*r* = 0.720, *p* < 0.001) and macrophages (*r* = 0.590, *p* < 0.001). In contrast, a relatively low level of Th17 infiltration was observed in the high *GGT5* expression group compared with the low *GGT5* expression group. (*r* = -0.220, *p* < 0.001), as shown in Figures 5B–E. Additionally, similar to the TCGA cohort, upregulated *GGT5* was positively associated with the infiltration of both NK cells and macrophages in the two GEO validation datasets. However, *GGT5* did not show a significant negative correlation with Th17 in the GSE54129 validation cohort, which may be related to sample size and selection bias, as shown in Supplementary Figures S1D, S2D **.**

### Correlation of *GGT5* With Immune-Related Genes and Immune Checkpoint Genes

**FIGURE 6:**
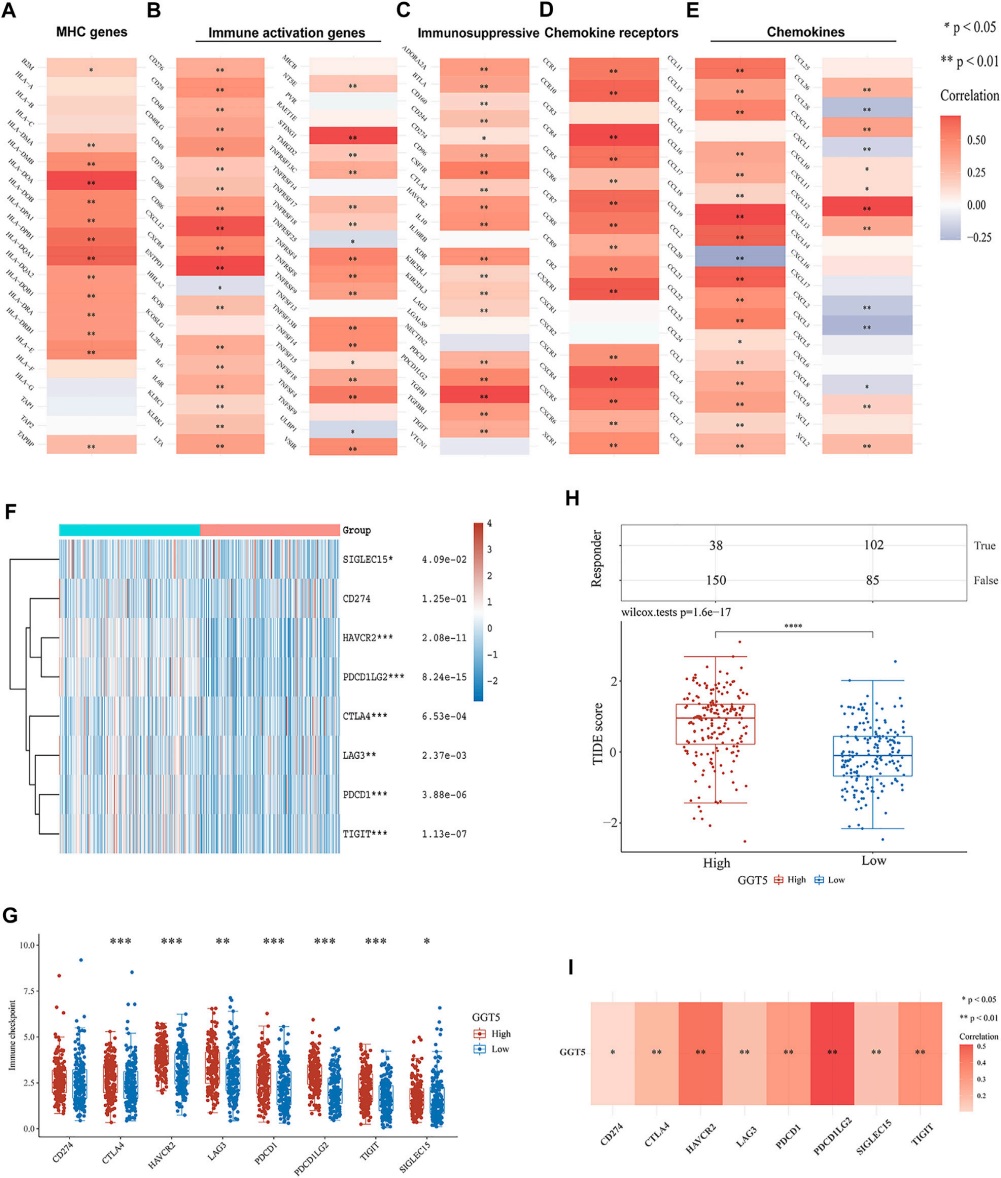
Correlation of *GGT5* with immune-related genes and immune checkpoint genes in gastric cancer. Correlation of *GGT5* and MHC genes **(A)**, immune activation genes **(B)**, immunosuppressive genes **(C)**, chemokine receptors **(D)**, and chemokines **(E)**. **(F,G)** The expression patterns and levels of immune checkpoint genes in different *GGT5* expression groups. **(H)** The predicted response to immune checkpoint blockade therapy in different *GGT5* expression groups. **(I)** Correlation of *GGT5* with immune checkpoint-related genes. *, *p* < 0.05; **, *p* < 0.01; ***, *p* < 0.001; ****, *p* < 0.0001.

To further understand the relationship between *GGT5* expression and immune cell infiltration in the microenvironment of gastric cancer, we performed a correlation analysis of *GGT5* and immune-related genes, including MHC genes, immune activation genes, immunosuppressive genes, chemokine receptors, and chemokines. The results indicated that *GGT5* had a positive relationship with MHC genes (Figure 6A) and immune activation genes (Figure 6B), especially *HLA-DOA*, *CXCL12*, *ENTPD1*, and *STING1*. Remarkably, the majority of immunosuppressive (Figure 6C) and chemokine receptors (Figure 6D) were positively correlated with *GGT5* expression. In addition, more than half of the chemokines (Figure 6E) were positively co-expressed with *GGT5* based on the TCGA cohort, and similar positive correlations of *GGT5* with chemokines were also observed in the validation cohorts of GSE54129 and GSE29272 (Supplementary Figures S3A–E and Supplementary Figures S4A–E). Therefore, it could be hypothesized that these immune-related genes, especially immunosuppressive and chemokines and related receptors may be involved in the regulation of immune cell infiltration patterns.

Since immunotherapy has been clinically utilized for the treatment of various tumors, we next compared the expression levels of immune checkpoint-related genes in different *GGT5* expression groups. The results showed that in addition to *CD274* (*p* = 1.25e-01), seven other genes, *CTLA4* (*p* = 6.53e-04), *HAVCR2* (*p* = 2.08e-11), *LAG3* (*p* = 2.37e-03), *PDCD1* (*p* = 3.88e-06), *PDCD1LG2* (*p* = 8.24e-15), *TIGIT* (*p* = 1.13e-07), and *SIGLEC15* (*p* = 4.09e-02), were obviously upregulated in the *GGT5*-high expression group (Figures 6F,G). A similar positive correlation was also seen in the validation dataset of GSE29272 (Supplementary Figures S4F,G). However, the expression of *GGT5* did not show a significant positive correlation with these immune checkpoint-related genes in GSE54129, which may be related to the small sample size and selection bias (Supplementary Figures S3F,G). Based on the TIDE algorithm to test the clinical response to immune checkpoint blockade, we demonstrated a significantly lower predicted response rate of gastric cancer patients in the *GGT5*-high expression group (Figure 6H). Furthermore, co-expression analysis revealed that the expression of *GGT5* was positively associated with these immune checkpoint-related genes in the TCGA cohort (Figure 6I), especially for *PDCD1LG2* and *PDCD1*, which were validated in the cohorts of GSE54129 and GSE29272, respectively (Supplementary Figures S3H, S4H). All of the above results indicated that *GGT5* may serve as a potential immunotherapy target.

### Functional Analysis of *GGT5* in Gastric Cancer

**FIGURE 7:**
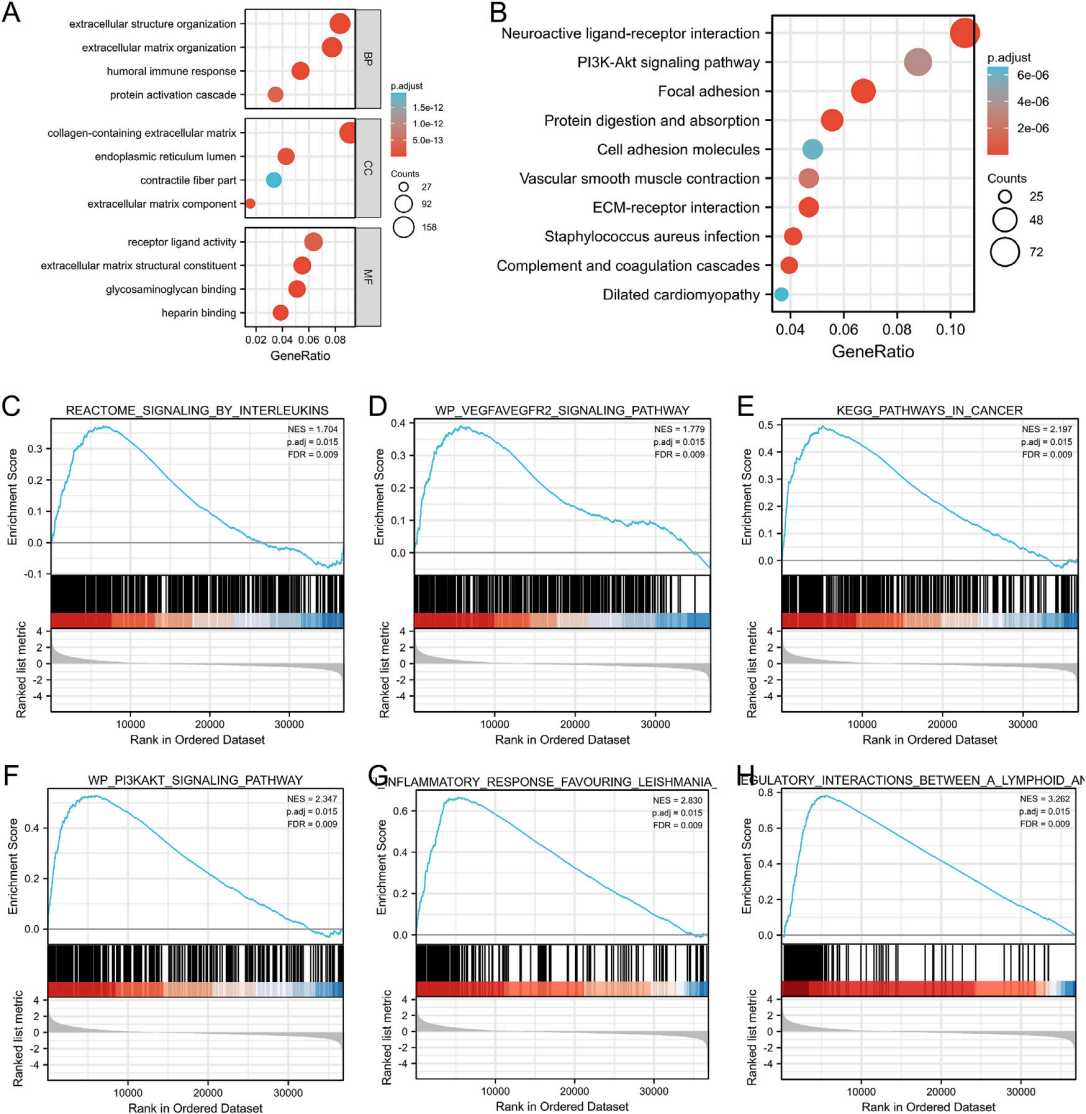
GO categories and pathway enrichment analysis of *GGT5* in gastric cancer. **(A)** GO annotations of DEGs. **(B)** KEGG pathway enrichment analysis determined the top 10 significantly enriched pathways. **(C–H)** GSEA was applied to identify *GGT5*-relevant signaling pathways and biological processes including signaling by interleukins, the VEGFA-VEGF2 signaling pathway, pathways in cancers, the PI3K/Akt signaling pathway, anti-inflammatory response favoring Leishmania, and immunoregulatory interactions between lymphoid and non-lymphoid cells. NES: normalized enrichment score; *p*. adj: adjusted *p value*; FDR: false discovery rate.

To gain further insight into the potential functions of *GGT5* in gastric cancer, GO categories and KEGG pathway enrichment analyses were carried out based on the TCGA database. The results showed that for the biological process, these *GGT5*-related DEGs were mainly enriched in extracellular structure/matrix organization, humoral immune response, and protein activation cascade. For the cellular component, DEGs were mainly involved in the collagen-containing extracellular matrix, endoplasmic reticulum lumen, and contractile fiber part. For the molecular function, the DEGs were mainly enriched in the processes of receptor-ligand activity, extracellular matrix structural constituent, and glycosaminoglycan binding (Figure 7A, Supplementary Table S6). In addition, the neuroactive ligand-receptor interaction, PI3K-Akt, focal adhesion, protein digestion, and protein absorption signaling pathways were also closely correlated with the regulation of *GGT5*-related DEGs (Figure 7B, Supplementary Table S7). Similar functional annotations, such as extracellular matrix structure constituents, collagen-containing extracellular matrix, glycosaminoglycan binding, and focal adhesion pathways, were also enriched in the two GEO validation cohorts (Supplementary Figures S1E,F, Supplementary Figures S2E,F, and Supplementary Tables S8, S9).

### GSEA Identified *GGT5*-Relevant Signaling Pathways

GSEA was performed to ascertain the related signaling pathways of *GGT5* in gastric cancer. The enrichment results indicated that pathways related to tumor proliferation and differentiation as well as the immune and inflammatory response were enriched in the *GGT5* high expression group, including signaling by interleukins, the VEGFA-VEGF2 signaling pathway, pathways in cancers, the PI3K/Akt signaling pathway, the anti-inflammatory response favoring Leishmania, and immunoregulatory interactions between lymphoid and non-lymphoid cells (Figures 7C–H, Supplementary Table S10). Taken together, *GGT5* is an immune-related gene and may play a critical role in inflammatory responses, angiogenesis, and the tumor immune response to promote the development and progression of gastric cancer.

## Discussion

To the best of our knowledge, the correlation between *GGT5* and immune infiltration levels in the tumor microenvironment of gastric cancer has not been previously reported. Herein, the present study confirmed that *GGT5* was upregulated in GC tissues and associated with a poor prognosis of patients with GC. ROC analysis implied that *GGT5* could be applied as a prognostic biomarker with moderate predictive value (AUCs between 0.7–0.8 are considered to be moderate) to distinguish GC tissues from normal tissues. Moreover, highly expressed *GGT5* was proven to be closely associated with a high histological grade, pathological stage, and poor prognosis of gastric cancer patients.

Recent studies have found a correlation between elevated serum levels of *GGT* and various digestive tumors, including pancreatic head carcinoma (Lyu et al., 2021), colorectal carcinoma (Gong et al., 2021), and gallbladder carcinoma (Su et al., 2021). A linear association was also detected between increased levels of *GGT* and cancer-related deaths (Albhaisi and Qayyum, 2021). Furthermore, as a traditional biochemical indicator, *GGT* has been widely used to evaluate the severity of digestive system-related diseases. Thus, the intrinsic connections and underlying mechanisms between *GGT* gene members and digestive tumors deserve further in-depth study.

Among the *GGT* gene family members, at least two members, *GGT1* and *GGT5*, have gained more attention due to their remarkable catalytic activity. Both *GGT1* and *GGT5* are type II membrane glycoproteins, which are mainly involved in the metabolism of tripeptide glutathione and leukotriene C4 (LTC4) (Heisterkamp et al., 2008; Hanigan et al., 2015). However, several differences in the organ/tissue distribution of the two enzymes have been observed. Previous studies have shown that *GGT1* is usually located on the apical surface of many epithelial cells, such as the surface of renal proximal tubules, prostate glands, and salivary glands, while *GGT5* is not localized to a specific region of the cell surface. Specifically, in the liver expressing both GGT1 and *GGT5*, *GGT1* is mainly distributed on the bile canaliculi surface, while *GGT5* is significantly expressed in Kupffer cells. Additionally, *GGT5* was found to play a critical role in converting LTC4 to LTD4 in the spleen, liver, and uterus (Hanigan et al., 2015). Its specific high expression in macrophages and its involvement in the metabolism of inflammatory mediators suggest that *GGT5* plays a crucial role in the regulation of the immune system.

According to the results of pan-cancer analysis, we found great heterogeneity in the expression levels of *GGT5* among various tumor types, which indicated that *GGT5* might play different roles in different tumors. Previous studies have concluded that *GGT5* overexpression is correlated with a poor prognosis in patients with lung cancer, gastric cancer, and colon cancer. A recent study showed that for lung adenocarcinoma (LUAD), *GGT5* was specifically highly expressed in cancer-associated fibroblasts instead of the tumor cells, which was proven to contribute to accelerating tumor cell proliferation and drug resistance in LUAD. Meanwhile, significantly poor OS and PFS were observed in LUAD patients with high *GGT5* expression (Wei et al., 2020). Additionally, a study on B-cell malignancy suggested that *GGT5* was overexpressed in follicular dendritic cells, which suggested a mechanism for B-cell confinement mediated by P2RY8 and the ligand S-geranylgeranyl-L-glutathione (Lu et al., 2019). Our present study has confirmed that *GGT5* plays a critical role in the progression of gastric cancer and could serve as an independent prognostic factor for gastric cancer patients.

Since the potential clinical value of *GGT5* in predicting the outcomes of gastric cancer patients was demonstrated, we established a nomogram including *GGT5* and other clinical characteristics (age, primary therapy outcome, residual tumor, and pathologic stage) according to the results of the univariate and multivariate Cox regressions. Such a scoring approach attempts to provide a more accurate and personalized prognostic assessment for gastric cancer patients by incorporating proven risk factors. Both the calibration curves and the C-index showed good agreement between the predicted and actual observed values for the 1-year, 2-years, and 3-years overall survival. Thus, our constructed nomogram model can be treated as a practical tool for individualized survival assessment of gastric cancer patients.

Because overexpression of *GGT5* was highly associated with a poor prognosis in gastric cancer, we further investigated the correlation between immune cell infiltration and *GGT5* expression to understand the potential mechanisms. It is well known that cells that infiltrate the tumor microenvironment, including immune cells, cancer cells, stromal cells, and extracellular matrix, have an impact on immune evasion, tolerance, and tumor progression. Our findings suggested that the infiltration levels of NK cells and macrophages were increased in the *GGT5*-high expression group, which showed significant correlations. Conversely, a negative correlation was observed between the Th17 infiltration level and *GGT5* expression. It has been confirmed that the function and phenotype of infiltrated NK cells in the tumor microenvironment are impaired, even leading to the dysfunction or exhaustion of NK cells (Zhang et al., 2020; Riggan et al., 2021). A recent study on non-small cell lung cancer (NSCLC) showed that increased cytotoxic T-lymphocyte-associated protein 4 (CTLA-4) in the tumor microenvironment could suppress the function of dendritic cells (DCs), resulting in immunosuppressive effects (Russick et al., 2020). The major proportions of macrophages infiltrating the tumor microenvironment are tumor-associated macrophages (TAMs) (Noy and Pollard, 2014). TAMs have emerged as a critical factor in promoting tumor progression by generating a complex mixture of inflammatory cytokines, chemokines, and growth factors (Noy and Pollard, 2014). In contrast, studies have demonstrated that the infiltration and function of Th17 cells in the tumor microenvironment are associated with tumor regression and survival improvement in patients diagnosed with epithelial carcinoma (Wilke et al., 2011). Animal experiments have also confirmed that Th17 cells promote the upregulation of CD_4_^+^ T lymphocytes in the tumor microenvironment, thus inhibiting tumor growth and prolonging survival in a mouse model of pancreatic carcinoma (Gnerlich et al., 2010). Therefore, our results suggest that the poor prognosis in the *GGT5*-high expression group might be attributed to an imbalance in the immune function homeostasis and an impaired anti-tumor immune response.

To gain further insight into the underlying mechanisms by which *GGT5* was correlated with immune cell infiltration, we performed a correlation analysis between *GGT5* and immune-related genes. The results revealed a positive correlation of *GGT5* with these immune-related genes, especially for immunosuppressive and chemokine receptor genes. Therefore, it could be inferred that the poor prognosis in the *GGT5*-high expression group is closely related to the high expression levels of immunosuppressive-related genes and chemokine receptors. The immune checkpoint analysis indicated that gastric cancer patients with upregulated *GGT5* expression showed a higher TIDE score and expression of *CD274*, *CTLA4*, *HAVCR2*, *LAG3*, *PDCD1*, *PDCD1LG2*, and *TIGIT* than patients in the *GGT5*-low expression group. Studies have shown that patients with a higher TIDE score suggest T cell dysfunction in the tumor microenvironment, which is associated not only with poor immune checkpoint blockade treatment but also with poor survival under anti-PD-1 and anti-CTLA4 therapy (Jiang et al., 2018). Therefore, these findings demonstrated that targeting *GGT5* may be a potential strategy for immune checkpoint blockade treatment.

To explore the biological function of *GGT5* in gastric cancer, *GGT5*-related DEGs were screened and subjected to GO functional annotation, GSEA, and KEGG pathway enrichment analyses. The GO functional annotations showed that extracellular matrix, receptor activity, and immune response were significantly enriched based on the TCGA database, and the extracellular matrix was especially enriched in both the TCGA and GEO cohorts. Interestingly, as a major component in the extracellular matrix, glycosaminoglycans are involved in all stages of cancer progression. For instance, cancer cells can secrete glycosaminoglycans such as heparinase and hyaluronidase to penetrate the basement membrane and extracellular matrix to invade surrounding tissues (Yip et al., 2006). Recent studies have shown that glycosaminoglycans can also interact with chemokines and drain biologics with chemokine neutralization functions, leaving free chemokines available to combine with chemokine receptors and promote cancer progression (Ortiz Zacarias et al., 2021). GSEA indicated that signaling by interleukins, the VEGFA-VEGF2 signaling pathway, pathways in cancers, the PI3K/Akt signaling pathway, the anti-inflammatory response favoring Leishmania, and immunoregulatory interactions between lymphoid and nonlymphoid cells were closely correlated with the *GGT5*-high phenotype. Based on the enrichment analyses, we may infer that *GGT5* plays important role in promoting the carcinogenesis and development of gastric cancer via a series of biological processes, such as immune response, angiogenesis, and inflammatory response. These results are consistent with the co-expression analysis of *GGT5* and immune-related genes.

Several studies have demonstrated that IL-6-, IL-8-, IL-10-, and IL-33 mediated pathways are involved in the invasion and metastasis of gastric cancer (Chung and Lim, 2017; Yang et al., 2018; Chen et al., 2019; Ham et al., 2019). For example, as a proinflammatory factor, IL33 was shown to promote the malignant progression of gastric cancer by activating the downstream ST2/MAPK/ERK1/2 signaling cascade (Yu et al., 2015; Zhou et al., 2020; Huang N. et al., 2021), which turned out to be a valuable prognostic biomarker for gastric cancer patients (Sun et al., 2011; Hu et al., 2017). In addition, IL33 could also inhibit platinum-induced apoptosis and promote cell invasion via the ST2/MAPK/JNK pathway, conferring resistance to gastric cancer chemotherapy (Ye et al., 2015). The PI3K/Akt/mTOR signaling pathway is commonly accepted as a vital pathway involved in various human cancers, and mediating epithelial-mesenchymal transformation and chemoresistance is considered to be the major factor promoting gastric cancer progression (Fattahi et al., 2020). Previous studies also revealed that VEGFR-2 mediated the major angiogenic functions of VEGF-A, including stimulating the proliferation and migration of endothelial cells, increasing vascular permeability, and promoting angiogenesis (Park et al., 2015). The circulating VEGE-A levels have been reported to be closely correlated with the treatment response, pathological characteristics, and prognosis of gastric cancer patients. Furthermore, antiangiogenic therapy targeting VEGF-A/VEGFR2, such as bevacizumab and ramucirumab, was recognized as an effective treatment strategy for advanced gastric cancer (Hironaka, 2019).

Our research confirmed the differential expression of *GGT5* between gastric cancer and normal tissues and increased our understanding of *GGT5* and the progression of gastric cancer from the perspective of immune cell infiltration. However, several limitations exist in the present study. First, all samples involved in the current study were based on RNA sequencing data from online databases. In addition to the data heterogeneity and platform differences, lacking or inconsistent clinical information may affect the accuracy of the outcomes. Therefore, prospective studies with large sample sizes are required to confirm our findings. Second, the association between *GGT5* expression and immune cell infiltration as well as the direct molecular mechanism of *GGT5* involvement in the progression of gastric cancer need further validation. Finally, the sample sizes of our study were still inadequate, particularly the results of some subgroup analyses, which may be affected by random chance. Additional studies with larger sample sizes are needed to prove these findings.

## Conclusion

Taken together, we confirmed that *GGT5* was highly expressed in gastric cancer tissues compared to normal samples, and it could be identified as a specific biomarker for distinguishing gastric cancer tissues from normal gastric mucosa. Upregulated *GGT5* was proven to be closely associated with poor overall survival and progression-free intervals in gastric cancer patients and could be applied as a clinically independent prognostic factor. A nomogram model was further constructed and computationally validated for individualized overall survival assessment. The immune cell infiltration analysis showed that *GGT5* expression was positively correlated with NK cells and macrophages but negatively correlated with the infiltration of Th17 cells. Additionally, we revealed that *GGT5* was co-expressed with immune-related genes and immune checkpoint genes and that *GGT5* may be a potential target for immune checkpoint blockade treatment. Finally, functional annotation and pathway enrichment analysis supported that *GGT5* was mainly involved in the biological processes of the immune response, angiogenesis, and inflammatory response. Our research provides a novel perspective for further understanding the mechanisms by which *GGT5* is correlated with immune cell infiltration in the tumor microenvironment of gastric cancer.

## Data Availability

The datasets presented in this study can be found in online repositories. The names of the repository/repositories and accession number(s) can be found in the article/Supplementary Material.

## Supplementary Material

Supplementary Figure S1 Independent validation of *GGT5*-related functional annotation, pathway enrichment, and its relationship with immune cell infiltration in GSE54129. **(A)** ROC curve showing the predictive value of *GGT5* for identifying gastric cancer tissues based on GSE54129. **(B,C)** Volcano plot **(B)** and heatmap clustering **(C)** of *GGT5*-related DEGs. **(D)** Correlation analysis between *GGT5* expression and immune cells infiltration. **(E)** GO annotations of *GGT5*-related DEGs. **(F)** KEGG pathway enrichment analysis displayed the significantly enriched pathways.

Supplementary Figure S2 Independent validation of *GGT5*-related functional annotation, pathway enrichment, and its relationship with immune cell infiltration in GSE29272. **(A)** ROC curve showing the predictive value of *GGT5* for identifying gastric cancer tissues based on GSE29272. **(B,C)** Volcano plot **(B)** and heatmap clustering **(C)** of *GGT5*-related DEGs. **(D)** Correlation analysis between *GGT5* expression and immune cells infiltration. **(E)** GO annotations of *GGT5*-related DEGs. **(F)** KEGG pathway enrichment analysis displayed the significantly enriched pathways.

Supplementary Figure S3 Validation of *GGT*5’s correlation with immune-related genes and immune checkpoint genes based on GSE54129. Correlation of *GGT5* and MHC genes **(A),** immune activation genes **(B),** immunosuppressive genes **(C),** chemokine receptors **(D),** and chemokines **(E)**. **(F,G)** The expression patterns and levels of immune checkpoint genes in different *GGT5* expression groups. **(H)** Correlation of *GGT5* expression level with immune checkpoint-related genes. *, *p* < 0.05; **, *p* < 0.01.

Supplementary Figure S4 Validation of *GGT*5’s correlation with immune-related genes and immune checkpoint genes based on GSE29272. Correlation of *GGT5* and MHC genes **(A),** immune activation genes **(B),** immunosuppressive genes **(C),** chemokine receptors **(D),** and chemokines **(E)**. **(F,G)** The expression patterns and levels of immune checkpoint genes in different *GGT5* expression groups. **(H)** Correlation of *GGT5* expression level with immune checkpoint-related genes. *, *p* < 0.05; **, *p* < 0.01; ***, *p* < 0.001.

Supplementary Table S1 List of *GGT5* mRNA expression levels in gastric cancer and normal gastric tissues in GEO and TCGA databases.

Supplementary Table S2 List of all differentially expressed genes.

Supplementary Table S3 List of 330 significantly differentially expressed genes with the absolute log2 fold change (FC)＞2 and adjusted *p*-value＜0.05 as cutoff criteria identified from the TCGA database.

Supplementary Table S4 List of 507 significantly differentially expressed genes detected from GSE54129.

Supplementary Table S5 List of 38 significantly differentially expressed genes detected from GSE29272.

Supplementary Table S6 List of GO enrichment analyses of DEGs based on the TCGA database.

Supplementary Table S7 List of KEGG enrichment analysis results based on the TCGA database.

Supplementary Table S8 List of GO annotations and KEGG pathway enrichment analysis based on GSE54129.

Supplementary Table S9 List of GO annotations and KEGG pathway enrichment analysis based on GSE29272.

Supplementary Table S10 List of gene set enrichment analysis results.
