# Supplementary material for: Identification of GGT5 as a Novel Prognostic Biomarker for Gastric Cancer and its Correlation With Immune Cell Infiltration
Source: Front Genet. 2022 Mar 18;13:810292. doi: 10.3389/fgene.2022.810292 (PMC8971189; doi:10.3389/fgene.2022.810292)
Supplement: Supplementary file 1 [file DataSheet7.PDF]

ONTOLOG	ID	Descriptior	GeneRatio	BgRatio	pvalue	p.adjust	qvalue	geneID	Count
KEGG	hsa04974	Protein dig	38/683	103/8076	7.50E-16	2.17E-13	1.74E-13	ATP1A2/AT	38
KEGG	hsa04080	Neuroactiv	72/683	341/8076	9.88E-14	1.43E-11	1.15E-11	ADCYAP1/	72
KEGG	hsa04512	ECM-recep	32/683	88/8076	2.42E-13	2.33E-11	1.88E-11	CD36/COL	32
KEGG	hsa04510	Focal adhe	46/683	201/8076	2.19E-10	1.58E-08	1.27E-08	CAV1/COL	46
KEGG	hsa04610	Compleme	27/683	85/8076	6.43E-10	3.71E-08	2.99E-08	A2M/SERP	27
KEGG	hsa05150	Staphylocc	28/683	96/8076	2.75E-09	1.33E-07	1.07E-07	C1R/C1S/C	28
KEGG	hsa04270	Vascular sr	32/683	135/8076	5.38E-08	2.22E-06	1.79E-06	ACTA2/AC	32
KEGG	hsa04151	PI3K-Akt s	60/683	354/8076	9.70E-08	3.50E-06	2.82E-06	CD19/CHR	60
KEGG	hsa04514	Cell adhesi	33/683	149/8076	1.88E-07	6.04E-06	4.87E-06	CD22/CNT	33
KEGG	hsa05414	Dilated car	25/683	96/8076	2.28E-07	6.60E-06	5.31E-06	ACTC1/AD	25
KEGG	hsa04020	Calcium sig	39/683	201/8076	5.94E-07	1.56E-05	1.26E-05	ADCY2/AD	39
KEGG	hsa04972	Pancreatic	25/683	102/8076	7.95E-07	1.91E-05	1.54E-05	ADCY2/AD	25
KEGG	hsa05412	Arrhythmo	21/683	77/8076	9.28E-07	2.06E-05	1.66E-05	ACTN2/CA	21
KEGG	hsa05410	Hypertropl	23/683	90/8076	9.98E-07	2.06E-05	1.66E-05	ACTC1/CA	23
KEGG	hsa04924	Renin secr	19/683	69/8076	2.61E-06	5.04E-05	4.05E-05	ADCY5/AD	19
KEGG	hsa04060	Cytokine-c	48/683	295/8076	6.32E-06	0.000114	9.19E-05	CXCR5/BM	48
KEGG	hsa05205	Proteoglyc	36/683	205/8076	1.72E-05	0.000293	0.000236	ANK2/CAV	36
KEGG	hsa04022	cGMP-PKC	31/683	167/8076	2.16E-05	0.000339	0.000273	ADCY2/AD	31
KEGG	hsa04024	cAMP sign	37/683	216/8076	2.34E-05	0.000339	0.000273	ADCY2/AD	37
KEGG	hsa04970	Salivary se	21/683	93/8076	2.35E-05	0.000339	0.000273	ADCY2/AD	21
KEGG	hsa04933	AGE-RAGE	21/683	100/8076	7.35E-05	0.000999	0.000804	AGTR1/CC	21
KEGG	hsa04915	Estrogen s	26/683	138/8076	7.60E-05	0.000999	0.000804	ADCY2/AD	26
KEGG	hsa05146	Amoebiasi	21/683	102/8076	9.94E-05	0.001249	0.001006	CD1C/CD1	21
KEGG	hsa04713	Circadian e	20/683	97/8076	0.000142	0.001706	0.001373	ADCY2/AD	20
KEGG	hsa04310	Wnt signal	28/683	160/8076	0.000156	0.001758	0.001415	CTNND2/N	28
KEGG	hsa04975	Fat digesti	12/683	43/8076	0.00016	0.001758	0.001415	APOA1/AP	12
KEGG	hsa04925	Aldosteror	20/683	98/8076	0.000164	0.001758	0.001415	ADCY2/AD	20
KEGG	hsa05144	Malaria	13/683	50/8076	0.000189	0.001953	0.001572	CD36/CON	13
KEGG	hsa04611	Platelet act	23/683	124/8076	0.000248	0.002467	0.001986	ADCY2/AD	23
KEGG	hsa04015	Rap1 signa	33/683	210/8076	0.000346	0.00333	0.002681	ADCY2/AD	33
KEGG	hsa04061	Viral prote	19/683	100/8076	0.000614	0.005727	0.00461	CXCR5/CC	19
KEGG	hsa04923	Regulation	13/683	57/8076	0.000751	0.006785	0.005462	ADCY2/AD	13
KEGG	hsa04640	Hematopo	18/683	99/8076	0.00144	0.012409	0.009988	CD1C/CD1	18
KEGG	hsa04971	Gastric aci	15/683	76/8076	0.001503	0.012409	0.009988	ADCY2/AD	15
KEGG	hsa05133	Pertussis	15/683	76/8076	0.001503	0.012409	0.009988	SERPING1/	15
KEGG	hsa04261	Adrenergic	24/683	150/8076	0.001667	0.013382	0.010772	ACTC1/AD	24
KEGG	hsa04657	IL-17 signa	17/683	94/8076	0.002051	0.016018	0.012894	DEFB4A/IL	17
KEGG	hsa04340	Hedgehog	11/683	50/8076	0.002562	0.019487	0.015686	EVC/GAS1	11
KEGG	hsa05165	Human pa	43/683	331/8076	0.002815	0.020857	0.016789	ATP6V1G2	43
KEGG	hsa04918	Thyroid hc	14/683	75/8076	0.003664	0.026471	0.021308	ADCY2/AD	14
KEGG	hsa04350	TGF-beta s	16/683	94/8076	0.005113	0.036043	0.029013	DCN/FBN1	16
KEGG	hsa04014	Ras signali	31/683	232/8076	0.006942	0.047769	0.038452	CALML3/C	31
KEGG	hsa04913	Ovarian ste	10/683	51/8076	0.00929	0.062434	0.050257	ADCY2/AD	10
KEGG	hsa04921	Oxytocin s	22/683	154/8076	0.010063	0.066096	0.053204	ADCY2/AD	22
KEGG	hsa04730	Long-term	11/683	60/8076	0.010865	0.069779	0.056169	CRH/GNAC	11
KEGG	hsa00980	Metabolisr	13/683	77/8076	0.011791	0.074081	0.059632	ADH1B/AD	13
KEGG	hsa04911	Insulin sec	14/683	86/8076	0.012532	0.077059	0.062029	ADCY2/AD	14
KEGG	hsa03320	PPAR signa	13/683	78/8076	0.013093	0.078828	0.063453	APOA1/AP	13
KEGG	hsa04724	Glutamate	17/683	114/8076	0.014851	0.087206	0.070197	ADCY2/AD	17
KEGG	hsa00982	Drug meta	12/683	71/8076	0.015088	0.087206	0.070197	ADH1B/AD	12
