# Supplementary material for: Identification of GGT5 as a Novel Prognostic Biomarker for Gastric Cancer and its Correlation With Immune Cell Infiltration
Source: Front Genet. 2022 Mar 18;13:810292. doi: 10.3389/fgene.2022.810292 (PMC8971189; doi:10.3389/fgene.2022.810292)
Supplement: Supplementary file 2 [file DataSheet4.PDF]

id	logFC	AveExpr	t	P.Value	adj.P.Val	B
GGT5	1.217207	7.028053	14.7143	6.97E-28	1.43E-23	52.495
COL6A2	1.454747	8.825142	10.68163	9.13E-19	4.50E-15	32.12111
BOC	1.169597	7.221645	10.64761	1.09E-18	4.50E-15	31.94473
COL4A2	1.412146	9.970069	10.55554	1.79E-18	6.12E-15	31.46736
EMILIN1	1.545154	7.659343	10.40394	4.00E-18	9.14E-15	30.68105
PCOLCE	1.373304	8.954507	10.17222	1.38E-17	2.11E-14	29.47902
ITGA7	1.67998	6.874814	10.16367	1.44E-17	2.11E-14	29.43466
FLNA	1.917432	10.00233	10.11916	1.82E-17	2.44E-14	29.20376
SELENOM	1.26459	9.373184	10.10906	1.93E-17	2.44E-14	29.15138
COL15A1	1.440375	10.13648	10.02674	2.98E-17	3.23E-14	28.72448
IGFBP7	1.030368	9.059133	10.00392	3.37E-17	3.30E-14	28.60611
DENND5A	1.080595	8.496833	9.984473	3.74E-17	3.49E-14	28.5053
PDLIM7	1.01196	6.500325	9.919771	5.27E-17	4.51E-14	28.16986
TMEM119	1.232617	6.737529	9.902503	5.78E-17	4.68E-14	28.08034
CAVIN1	1.459924	8.344953	9.874026	6.72E-17	5.12E-14	27.93274
EFEMP2	1.243878	8.130478	9.809152	9.49E-17	6.73E-14	27.59657
PDGFRB	1.130269	8.033	9.748954	1.31E-16	8.67E-14	27.28472
LHFPL6	1.264697	7.530612	9.70096	1.69E-16	1.05E-13	27.03617
LRRC32	1.025677	7.871603	9.689709	1.79E-16	1.08E-13	26.97791
ELN	1.026134	6.920617	9.645688	2.26E-16	1.26E-13	26.75003
HSPG2	1.1368	7.881679	9.548504	3.79E-16	1.86E-13	26.24718
PALD1	1.040659	5.89401	9.520164	4.41E-16	2.11E-13	26.10062
TGFB1I1	1.706111	9.520273	9.511281	4.62E-16	2.16E-13	26.05469
IGFBP4	1.519654	10.3454	9.48332	5.36E-16	2.39E-13	25.91013
TNS1	1.218982	7.282174	9.443417	6.62E-16	2.78E-13	25.70389
ZCCHC24	1.44775	8.442869	9.398304	8.41E-16	3.46E-13	25.47083
C1R	1.290344	10.36831	9.375906	9.47E-16	3.82E-13	25.35515
RGMA	1.015766	5.755405	9.35741	1.04E-15	4.05E-13	25.25965
SERPINF1	1.400609	10.47666	9.335785	1.17E-15	4.38E-13	25.14802
CNRIP1	1.159352	8.066512	9.323877	1.25E-15	4.58E-13	25.08656
TUBB6	1.192539	7.514835	9.312401	1.33E-15	4.70E-13	25.02733
FBXL7	1.047679	6.394683	9.276157	1.61E-15	5.59E-13	24.84033
MYL9	1.400284	8.324489	9.260726	1.74E-15	5.97E-13	24.76074
FXYD6	1.61247	8.021948	9.234838	2.00E-15	6.73E-13	24.62725
MCAM	1.065528	7.228631	9.208182	2.30E-15	7.43E-13	24.48984
HOOK1	-1.40088	6.044556	-9.20706	2.32E-15	7.43E-13	24.48404
PLEKHO1	1.299645	7.713279	9.20156	2.38E-15	7.54E-13	24.45572
RASL12	1.045081	6.641312	9.182957	2.63E-15	8.19E-13	24.35986
CRYAB	1.775849	8.293493	9.154676	3.05E-15	9.25E-13	24.21417
SOD3	1.372564	7.730472	9.130833	3.46E-15	1.03E-12	24.09139
KCNJ8	1.194732	6.652415	9.125245	3.57E-15	1.05E-12	24.06262
JAM3	1.181666	6.148118	9.117745	3.71E-15	1.07E-12	24.02401
COL4A1	1.137935	10.10491	9.110778	3.85E-15	1.09E-12	23.98814
FRK	-1.17349	5.31956	-9.08309	4.46E-15	1.15E-12	23.84566
ZNF423	1.667503	6.945089	9.059778	5.04E-15	1.28E-12	23.72572
ECSCR	1.022157	7.085912	9.049323	5.33E-15	1.34E-12	23.67195
PRICKLE2	1.398729	6.750544	9.021263	6.18E-15	1.51E-12	23.52767
FLRT2	1.146844	5.507095	9.006469	6.68E-15	1.60E-12	23.45163
EDNRA	1.502926	7.209581	8.912907	1.09E-14	2.39E-12	22.97113
LIMS2	1.468963	7.178858	8.891918	1.22E-14	2.64E-12	22.86344
MXRA7	1.089597	7.753265	8.873288	1.35E-14	2.85E-12	22.76788
THBS4	2.571255	8.700926	8.824985	1.74E-14	3.50E-12	22.52027
MN1	1.688159	6.18721	8.822155	1.76E-14	3.52E-12	22.50577
CSRP1	1.587452	10.84407	8.798405	2.00E-14	3.95E-12	22.3841
GUCY1B1	1.322337	7.716802	8.781867	2.18E-14	4.27E-12	22.29942
ITGA5	1.503128	8.150882	8.761139	2.43E-14	4.67E-12	22.19331
FBLN5	1.269473	8.019912	8.757237	2.48E-14	4.72E-12	22.17334

CPXM2	1.070506	6.188556	8.744679	2.65E-14	4.95E-12	22.10908
THY1	1.169304	8.592314	8.715418	3.09E-14	5.67E-12	21.95942
CD248	1.046976	8.388485	8.711116	3.16E-14	5.75E-12	21.93742
BGN	1.292377	8.331402	8.69819	3.38E-14	6.04E-12	21.87134
COX7A1	1.288984	8.021955	8.693453	3.47E-14	6.14E-12	21.84713
MIR100HG	1.498636	5.999346	8.678028	3.76E-14	6.55E-12	21.7683
OLFML2B	1.304684	7.630308	8.668741	3.95E-14	6.82E-12	21.72085
CARMN	1.844978	7.530244	8.652613	4.30E-14	7.36E-12	21.63847
LMOD1	2.03992	7.687589	8.595058	5.81E-14	9.38E-12	21.34472
NR2F1	1.210243	7.184545	8.591515	5.92E-14	9.42E-12	21.32665
TAGLN	1.672516	10.49644	8.583158	6.18E-14	9.77E-12	21.28403
PRELP	1.311306	7.447741	8.573202	6.51E-14	1.01E-11	21.23326
RERG	1.758153	6.599272	8.566179	6.76E-14	1.01E-11	21.19746
MSRB3	1.337102	7.313543	8.539086	7.78E-14	1.13E-11	21.05939
SVIL	1.001688	6.997307	8.518704	8.66E-14	1.22E-11	20.95558
MEOX1	1.366544	5.910336	8.498649	9.62E-14	1.32E-11	20.85349
C1QTNF2	1.161997	5.458549	8.471401	1.11E-13	1.50E-11	20.71484
BARX1	1.521115	6.488389	8.447613	1.26E-13	1.65E-11	20.59388
COL6A3	1.204932	11.63037	8.44464	1.28E-13	1.66E-11	20.57876
FLNC	1.378949	7.679344	8.423801	1.42E-13	1.83E-11	20.47286
CAV1	1.317852	9.735359	8.367742	1.90E-13	2.33E-11	20.18823
TNS2	1.053515	7.412437	8.35535	2.03E-13	2.45E-11	20.12537
ASB2	1.448814	6.027167	8.352107	2.07E-13	2.45E-11	20.10892
AOC3	1.928572	7.927847	8.350457	2.08E-13	2.45E-11	20.10056
C14ORF13	1.185821	6.363085	8.345412	2.14E-13	2.50E-11	20.07497
MGP	1.957658	9.043371	8.342958	2.17E-13	2.51E-11	20.06253
KCNMB1	1.228273	5.886994	8.324611	2.38E-13	2.74E-11	19.96952
FBLN2	1.062896	6.511393	8.308435	2.59E-13	2.88E-11	19.88756
FERMT2	1.390026	8.312131	8.293521	2.80E-13	3.05E-11	19.81202
SLIT2	1.743049	6.741017	8.267905	3.20E-13	3.36E-11	19.68235
DSC2	-1.42781	6.369757	-8.21829	4.14E-13	4.17E-11	19.43146
C1ORF210	-1.37513	6.183969	-8.21237	4.27E-13	4.26E-11	19.40151
ACTA2	1.114502	7.36087	8.206346	4.41E-13	4.37E-11	19.3711
DDR2	1.306332	7.638474	8.18959	4.81E-13	4.66E-11	19.28647
CDH5	1.052001	6.914318	8.182018	5.00E-13	4.80E-11	19.24824
TCEAL7	1.536707	6.270371	8.178235	5.10E-13	4.85E-11	19.22914
ATP2B4	1.059458	7.833371	8.170691	5.30E-13	5.02E-11	19.19106
TUBA1A	1.1029	11.04806	8.146081	6.02E-13	5.52E-11	19.06691
MAP1B	1.313353	7.303344	8.128434	6.60E-13	5.97E-11	18.97793
LTBP1	1.179853	8.291497	8.125949	6.68E-13	6.02E-11	18.9654
DAAM2	1.170435	7.109177	8.111636	7.20E-13	6.43E-11	18.89328
DMPK	1.109967	6.573508	8.107512	7.35E-13	6.54E-11	18.87251
SPON1	1.420637	6.855186	8.082441	8.37E-13	7.20E-11	18.74626
AEBP1	1.335596	8.952577	8.077241	8.60E-13	7.36E-11	18.72009
MRGPRF	1.797163	7.633585	8.050655	9.87E-13	8.34E-11	18.58634
NDN	1.18581	7.703547	8.039925	1.04E-12	8.75E-11	18.53239
MEOX2	1.016105	3.613889	8.027547	1.11E-12	9.29E-11	18.47018
MYLK	1.308466	7.042026	8.006528	1.24E-12	1.02E-10	18.36459
HSPB6	2.150715	7.102728	7.986465	1.37E-12	1.12E-10	18.26387
DPYSL3	1.29134	7.026802	7.964513	1.54E-12	1.24E-10	18.15375
BNC2	1.125149	5.787871	7.961623	1.56E-12	1.25E-10	18.13925
HMGCS1	-1.10268	6.742785	-7.96101	1.57E-12	1.25E-10	18.13616
PPP1R14A	1.665333	7.140152	7.955549	1.61E-12	1.28E-10	18.1088
ELOVL6	-1.18917	6.165525	-7.95391	1.63E-12	1.28E-10	18.10056
NXN	1.088246	9.012435	7.943717	1.71E-12	1.35E-10	18.04949
PLPPR4	1.391603	4.700289	7.942178	1.73E-12	1.35E-10	18.04178
ZFPM2	1.652663	5.768506	7.922771	1.91E-12	1.48E-10	17.94456
TNC	1.627777	6.760048	7.82182	3.20E-12	2.31E-10	17.43986

CDS1	-1.02495	6.34124	-7.8156	3.31E-12	2.38E-10	17.4088
CASQ2	1.473034	6.114102	7.803644	3.52E-12	2.47E-10	17.34918
DCLK1	1.216579	5.014538	7.793282	3.71E-12	2.57E-10	17.2975
MPDZ	1.102218	7.151198	7.793165	3.71E-12	2.57E-10	17.29692
CCL28	-1.33235	5.884944	-7.78099	3.95E-12	2.71E-10	17.23621
GEM	1.151076	9.59994	7.770942	4.16E-12	2.81E-10	17.18617
NEURL1B	1.108205	8.076906	7.767067	4.24E-12	2.85E-10	17.16687
RNF150	1.064602	5.21886	7.748826	4.66E-12	3.06E-10	17.07604
PBX3	1.135096	8.219265	7.742246	4.82E-12	3.13E-10	17.04329
PDLIM3	1.17879	6.414109	7.740271	4.87E-12	3.14E-10	17.03347
FBLN1	1.056369	7.397382	7.716962	5.48E-12	3.49E-10	16.91753
NAP1L3	1.523752	5.341613	7.712948	5.59E-12	3.55E-10	16.89758
FBXL22	1.293576	5.620096	7.680951	6.59E-12	4.03E-10	16.73862
ANGPTL2	1.14201	7.194799	7.679433	6.64E-12	4.05E-10	16.73108
HSPB8	1.105076	6.825334	7.660209	7.32E-12	4.39E-10	16.63568
ITIH5	1.085078	6.500435	7.64805	7.79E-12	4.60E-10	16.57537
GUCY1A1	1.41523	7.843413	7.641064	8.07E-12	4.74E-10	16.54074
INMT	1.251879	6.405333	7.632314	8.44E-12	4.88E-10	16.49737
SPARC	1.026122	10.7649	7.632174	8.45E-12	4.88E-10	16.49667
AKAP12	1.155635	6.064236	7.624941	8.77E-12	5.02E-10	16.46084
STARD9	1.038666	4.500982	7.616931	9.13E-12	5.20E-10	16.42116
DES	1.577595	7.284723	7.594252	1.02E-11	5.78E-10	16.30889
CDC42EP3	1.121158	7.326009	7.591431	1.04E-11	5.82E-10	16.29493
KCNMA1	1.011811	4.982679	7.586895	1.06E-11	5.92E-10	16.2725
OMD	1.696379	5.697506	7.582707	1.09E-11	6.02E-10	16.25178
CNN1	2.348648	9.646429	7.563754	1.20E-11	6.50E-10	16.15808
FBN1	1.264444	7.971206	7.5536	1.26E-11	6.78E-10	16.10791
SERPING1	1.173797	9.672456	7.541202	1.34E-11	7.17E-10	16.04668
MBNL1-AS	1.568226	6.646379	7.533034	1.40E-11	7.41E-10	16.00636
ZEB1	1.129339	6.631622	7.528833	1.43E-11	7.53E-10	15.98562
MXRA8	1.018946	8.865445	7.517741	1.51E-11	7.86E-10	15.9309
CYP1B1	1.425017	7.005762	7.512975	1.55E-11	8.03E-10	15.90739
PDZRN4	1.840463	4.6423	7.494293	1.70E-11	8.68E-10	15.81529
RBPMS2	1.674201	6.879927	7.484915	1.79E-11	9.01E-10	15.76909
DNAJB5	1.179687	6.079115	7.472116	1.90E-11	9.48E-10	15.70606
FXYD1	1.298087	6.912193	7.470239	1.92E-11	9.54E-10	15.69682
NIBAN1	1.29212	8.758821	7.455337	2.07E-11	1.02E-09	15.62349
C1S	1.118265	10.18992	7.452032	2.11E-11	1.04E-09	15.60723
TMEM125	-1.02596	7.720258	-7.43745	2.27E-11	1.09E-09	15.53553
NRXN3	1.213203	5.432326	7.434698	2.30E-11	1.10E-09	15.522
PLTP	1.073577	8.536523	7.394171	2.82E-11	1.31E-09	15.32298
SPARCL1	1.290067	11.70031	7.392478	2.85E-11	1.32E-09	15.31467
CCDC80	1.556387	7.695237	7.380615	3.02E-11	1.38E-09	15.25649
LOC72839	1.009801	7.046307	7.370015	3.19E-11	1.43E-09	15.20453
SELP	1.021945	7.260613	7.36724	3.24E-11	1.45E-09	15.19092
CALD1	1.056836	7.889221	7.366689	3.24E-11	1.45E-09	15.18822
DSG2	-1.60475	8.460379	-7.36138	3.33E-11	1.49E-09	15.16219
CRISPLD1	1.836075	6.174041	7.351059	3.51E-11	1.56E-09	15.11166
TTC7B	1.063074	6.119708	7.346791	3.59E-11	1.59E-09	15.09076
SCRG1	1.326334	5.066141	7.33349	3.84E-11	1.68E-09	15.02566
HAND2-A	1.853082	5.10487	7.319634	4.11E-11	1.79E-09	14.95788
OCLN	-1.00836	6.639281	-7.31803	4.15E-11	1.79E-09	14.95002
PYGM	1.046311	5.330279	7.313057	4.25E-11	1.83E-09	14.92573
C8ORF88	1.583532	5.487691	7.276351	5.11E-11	2.13E-09	14.74645
EBF1	1.014703	5.59995	7.256307	5.65E-11	2.34E-09	14.64868
PIP4P2	1.017947	6.136698	7.255382	5.68E-11	2.35E-09	14.64417
CCNE2	-1.0289	4.976899	-7.24024	6.13E-11	2.51E-09	14.57039
PSAT1	-1.1235	6.921556	-7.23037	6.44E-11	2.62E-09	14.5223

JAM2	1.276142	6.890308	7.222791	6.69E-11	2.70E-09	14.48541
CORO2A	-1.21733	6.566842	-7.22203	6.71E-11	2.71E-09	14.48169
CCDC69	1.157315	6.995498	7.221015	6.75E-11	2.72E-09	14.47677
PLIN4	1.10576	6.142977	7.2165	6.90E-11	2.77E-09	14.45479
GFPT2	1.111017	6.557954	7.207596	7.22E-11	2.87E-09	14.41147
PRRX1	1.141466	6.218176	7.203066	7.38E-11	2.92E-09	14.38944
GLIS2	1.070003	7.202391	7.191178	7.83E-11	3.07E-09	14.33165
ATP1A2	1.514788	5.805595	7.186061	8.04E-11	3.13E-09	14.30679
SYNC	1.491106	6.48261	7.17555	8.47E-11	3.28E-09	14.25573
SHISAL1	1.081002	5.52557	7.163454	9.00E-11	3.45E-09	14.19701
COLEC12	1.217892	6.990108	7.161655	9.08E-11	3.47E-09	14.18827
MYH11	1.781967	8.092194	7.151323	9.56E-11	3.64E-09	14.13815
PGM5-AS1	1.713454	6.226884	7.141581	1.00E-10	3.78E-09	14.09091
RAB23	1.301239	7.764862	7.135324	1.04E-10	3.87E-09	14.06058
C4B	1.337213	8.998809	7.131134	1.06E-10	3.91E-09	14.04027
EML1	1.340449	6.7722	7.119055	1.12E-10	4.14E-09	13.98176
GPRASP1	1.272782	6.680861	7.101132	1.23E-10	4.48E-09	13.89501
HGD	-1.00959	5.197292	-7.08673	1.32E-10	4.81E-09	13.82535
CILP	1.528033	7.747016	7.082011	1.35E-10	4.89E-09	13.80255
LOC10192	1.248566	5.10251	7.064078	1.48E-10	5.24E-09	13.71591
CFL2	1.410237	7.652482	7.064069	1.48E-10	5.24E-09	13.71587
SOWAHA	-1.37287	5.430817	-7.05367	1.56E-10	5.50E-09	13.66565
NID2	1.015036	7.992101	7.043802	1.63E-10	5.73E-09	13.61806
C7	1.544673	6.846922	7.033531	1.72E-10	5.96E-09	13.56853
IGFBP6	1.096175	7.835218	7.032759	1.73E-10	5.96E-09	13.5648
SYNPO2	1.878025	8.337426	7.031426	1.74E-10	5.99E-09	13.55838
MFAP4	1.481861	8.534841	7.028605	1.76E-10	6.06E-09	13.54478
FMO5	-1.21319	5.441509	-7.01315	1.90E-10	6.47E-09	13.47033
CLMP	1.247266	6.840748	7.008303	1.95E-10	6.58E-09	13.44699
COL21A1	1.480922	6.165122	7.005526	1.97E-10	6.65E-09	13.43362
RHPN2	-1.5222	8.424013	-6.99869	2.04E-10	6.83E-09	13.40069
SYNM	2.369043	9.252245	6.997063	2.06E-10	6.86E-09	13.39289
PGM5	1.048703	5.534761	6.995116	2.08E-10	6.89E-09	13.38352
GAS1	1.268991	5.894409	6.985674	2.18E-10	7.18E-09	13.3381
CPED1	1.001276	7.237573	6.981219	2.23E-10	7.29E-09	13.31669
TENT5B	1.289987	6.109857	6.975102	2.30E-10	7.49E-09	13.28728
APOD	1.865131	9.331002	6.962757	2.44E-10	7.84E-09	13.22797
CHRDL2	1.807168	7.517799	6.955792	2.53E-10	8.06E-09	13.19453
SRPX	1.670977	7.787066	6.947455	2.63E-10	8.33E-09	13.15452
SMOC2	1.263309	7.468089	6.944524	2.67E-10	8.44E-09	13.14045
EP300-AS1	-1.0611	4.297255	-6.94055	2.72E-10	8.56E-09	13.12136
SLC27A2	-1.42114	6.236599	-6.93962	2.74E-10	8.56E-09	13.11694
ACTG2	1.109653	8.273216	6.909758	3.17E-10	9.71E-09	12.97382
C11ORF96	1.095992	10.34198	6.896866	3.38E-10	1.03E-08	12.9121
AGTR1	1.340408	5.068311	6.894816	3.41E-10	1.04E-08	12.9023
TACR2	1.515788	6.221509	6.892864	3.45E-10	1.04E-08	12.89296
CXCL12	1.470563	8.11953	6.890888	3.48E-10	1.05E-08	12.88351
UGT8	-1.48587	6.485509	-6.8862	3.56E-10	1.07E-08	12.86109
DACT1	1.065458	7.265471	6.87478	3.77E-10	1.12E-08	12.80649
FILIP1	1.42451	5.059528	6.86049	4.04E-10	1.19E-08	12.73823
FHL1	1.696589	9.246401	6.859313	4.07E-10	1.19E-08	12.7326
PRSS16	-1.16996	5.753462	-6.85567	4.14E-10	1.21E-08	12.71523
ANGPTL1	1.535646	5.423177	6.854419	4.17E-10	1.22E-08	12.70924
INAFM2	1.052846	7.074009	6.837208	4.53E-10	1.31E-08	12.62713
ZNF165	-1.18954	5.246994	-6.83004	4.70E-10	1.35E-08	12.59295
ADGRD1	1.260299	5.973246	6.808632	5.22E-10	1.48E-08	12.49097
ZNF521	1.037613	5.696295	6.805711	5.29E-10	1.49E-08	12.47707
MYOCD	1.628478	7.276134	6.797694	5.50E-10	1.54E-08	12.43892

AHNAK2	1.029682	6.12156	6.783971	5.89E-10	1.63E-08	12.37366
RRM2	-1.22959	8.752001	-6.76914	6.33E-10	1.74E-08	12.30317
ISLR	1.356673	7.475445	6.760789	6.59E-10	1.81E-08	12.26353
CTSG	1.070703	5.83703	6.733832	7.52E-10	2.03E-08	12.13567
HSPA2	1.180844	7.407722	6.728349	7.73E-10	2.08E-08	12.10969
FAT4	1.005238	6.139643	6.726068	7.81E-10	2.09E-08	12.09888
EMCN	1.11513	6.629437	6.717921	8.13E-10	2.17E-08	12.0603
RASSF6	-1.62187	6.377613	-6.70293	8.75E-10	2.32E-08	11.98938
VIP	1.420777	4.374842	6.68061	9.75E-10	2.55E-08	11.88385
NCAPG	-1.11155	5.588896	-6.6722	1.02E-09	2.65E-08	11.84414
LRRN4CL	1.119497	6.577438	6.669519	1.03E-09	2.68E-08	11.83147
NEXN	1.593736	7.548251	6.666562	1.04E-09	2.71E-08	11.81751
CCL2	1.01911	8.583296	6.663717	1.06E-09	2.74E-08	11.80409
OGN	2.203528	8.014376	6.661443	1.07E-09	2.76E-08	11.79336
FAM3B	-2.45492	7.828195	-6.65582	1.10E-09	2.82E-08	11.76684
LINC0127[illegible]	1.916749	7.847854	6.644314	1.16E-09	2.97E-08	11.71258
MAP3K21	-1.13789	6.469173	-6.63952	1.19E-09	3.03E-08	11.68999
ZNF667-A	1.021826	5.741017	6.620686	1.31E-09	3.27E-08	11.60131
GXYLT2	1.183392	6.448483	6.618253	1.32E-09	3.30E-08	11.58986
TCEAL2	1.783229	6.074692	6.612001	1.36E-09	3.39E-08	11.56045
GRAMD1C	-1.02489	5.450715	-6.59777	1.46E-09	3.61E-08	11.49354
ITGA1	1.06127	6.254008	6.590529	1.51E-09	3.72E-08	11.45954
MRVI1	1.217443	7.504533	6.573024	1.64E-09	4.02E-08	11.37737
FOXF1	1.067171	8.646647	6.552432	1.82E-09	4.40E-08	11.28082
FMO1	1.283891	6.545772	6.546042	1.87E-09	4.53E-08	11.2509
LINC0179[illegible]	1.259486	5.229042	6.539367	1.94E-09	4.65E-08	11.21964
PBK	-1.51677	7.400494	-6.53831	1.95E-09	4.66E-08	11.21472
FRMD6	1.067257	8.204729	6.536887	1.96E-09	4.68E-08	11.20803
KIF11	-1.13197	6.03933	-6.5151	2.18E-09	5.10E-08	11.10616
MEIS1	1.101355	7.461453	6.514852	2.18E-09	5.10E-08	11.10498
MELK	-1.14439	7.592183	-6.51049	2.22E-09	5.19E-08	11.08458
AQP1	1.212085	9.106095	6.503099	2.31E-09	5.34E-08	11.05008
TMEM45B	-1.64975	8.093177	-6.49762	2.37E-09	5.47E-08	11.02451
PRUNE2	1.701231	6.723996	6.479248	2.59E-09	5.92E-08	10.9388
ARMCX2	1.024376	7.918054	6.473586	2.66E-09	6.06E-08	10.9124
TMEM47	1.119931	8.117804	6.46768	2.73E-09	6.20E-08	10.88489
COL16A1	1.069201	8.127345	6.456331	2.89E-09	6.48E-08	10.83204
ECRG4	2.402482	8.641374	6.434821	3.20E-09	7.11E-08	10.73199
SLC44A4	-1.60361	7.884705	-6.43271	3.23E-09	7.16E-08	10.72219
DHCR24	-1.11326	8.203451	-6.43157	3.25E-09	7.19E-08	10.71688
PLN	2.069451	7.720268	6.427867	3.31E-09	7.30E-08	10.69968
PLSCR4	1.047892	7.502867	6.416742	3.49E-09	7.64E-08	10.64802
LOC10050	-1.00216	6.218829	-6.41564	3.51E-09	7.67E-08	10.64291
TMEM35A	1.479746	5.005797	6.406169	3.67E-09	7.99E-08	10.59896
PEG3	1.106567	4.237056	6.391939	3.93E-09	8.47E-08	10.53299
NAT1	-1.03237	7.862801	-6.37849	4.19E-09	8.98E-08	10.47069
TTK	-1.35661	6.402422	-6.33989	5.04E-09	1.06E-07	10.29224
BEX4	1.045138	7.346701	6.317045	5.62E-09	1.16E-07	10.18689
FOXA1	-1.13468	4.854117	-6.31659	5.63E-09	1.17E-07	10.18477
EHF	-1.04227	6.504369	-6.31599	5.65E-09	1.17E-07	10.18203
CAP2	1.341875	6.803936	6.315142	5.67E-09	1.17E-07	10.17811
GHR	1.414178	5.330908	6.304654	5.96E-09	1.22E-07	10.1298
F2RL1	-1.42531	7.985931	-6.28516	6.54E-09	1.32E-07	10.04009
KIF15	-1.02002	5.623784	-6.25779	7.45E-09	1.47E-07	9.914389
OLFML3	1.128828	8.603627	6.208454	9.41E-09	1.81E-07	9.688436
ANXA3	-1.0816	8.830479	-6.20604	9.52E-09	1.82E-07	9.677377
STMN2	1.032817	5.116837	6.193964	1.01E-08	1.92E-07	9.622233
RBP7	1.074634	6.558266	6.166455	1.15E-08	2.16E-07	9.496754

ACTC1	1.098717	7.232065	6.163791	1.16E-08	2.19E-07	9.484618
HMMR	-1.05554	6.877043	-6.16276	1.17E-08	2.19E-07	9.479933
ASPN	1.566182	9.368585	6.162172	1.17E-08	2.20E-07	9.477239
CXADR	-1.2931	6.777473	-6.16173	1.17E-08	2.20E-07	9.475213
CAVIN2	1.216279	6.58849	6.158524	1.19E-08	2.23E-07	9.460625
CHRDL1	1.777542	6.840428	6.130065	1.36E-08	2.50E-07	9.331179
FMO2	1.146289	6.536721	6.116043	1.45E-08	2.64E-07	9.267508
ADAMTS1	1.106632	7.902857	6.093357	1.62E-08	2.90E-07	9.164646
SFRP2	1.949268	9.876289	6.083511	1.69E-08	3.02E-07	9.120057
SPC25	-1.1696	4.984072	-6.07559	1.76E-08	3.12E-07	9.084203
MUC13	-1.6796	8.079818	-6.05094	1.97E-08	3.45E-07	8.97282
RSPO3	1.384591	7.825574	6.040737	2.07E-08	3.58E-07	8.926777
GPM6B	1.133882	5.657858	6.039994	2.08E-08	3.59E-07	8.923426
C3	1.227073	11.16065	6.033982	2.13E-08	3.67E-07	8.896319
OR7E14P	-1.07612	5.355523	-6.02098	2.27E-08	3.86E-07	8.837748
SPOCK1	1.492438	7.352012	5.995579	2.55E-08	4.26E-07	8.723466
PRIMA1	1.44319	7.089664	5.984751	2.68E-08	4.45E-07	8.674831
TMEM100	1.536598	6.97137	5.980557	2.74E-08	4.53E-07	8.656002
ESRP1	-1.27055	8.473908	-5.97058	2.87E-08	4.71E-07	8.611244
LRRC66	-1.45566	4.718893	-5.9538	3.10E-08	5.00E-07	8.536033
SMPX	1.77825	5.103755	5.945076	3.23E-08	5.15E-07	8.497
MORN5	1.123863	4.699184	5.930601	3.45E-08	5.47E-07	8.432273
BCHE	1.909211	5.089695	5.887698	4.21E-08	6.50E-07	8.240896
RYR3	1.069128	3.750221	5.860551	4.77E-08	7.30E-07	8.120173
IGF1	1.075668	6.518329	5.821358	5.71E-08	8.48E-07	7.946393
HLF	1.064844	4.650034	5.810946	5.99E-08	8.81E-07	7.900331
GIPC2	-1.07499	6.24784	-5.80061	6.28E-08	9.18E-07	7.854665
DSP	-1.20434	9.732196	-5.79914	6.32E-08	9.23E-07	7.848138
PLP1	1.397005	5.274283	5.789435	6.61E-08	9.62E-07	7.805305
CPXM1	1.110865	6.205924	5.784485	6.76E-08	9.82E-07	7.783467
CHMP4C	-1.2293	7.484011	-5.77862	6.94E-08	1.01E-06	7.757599
CCNB1	-1.06614	7.043264	-5.77092	7.19E-08	1.03E-06	7.723683
SLC7A11	-1.12349	5.767418	-5.76716	7.32E-08	1.05E-06	7.707082
PTGDS	1.198081	9.380104	5.759666	7.57E-08	1.08E-06	7.674106
PRR15L	-1.29676	8.414873	-5.75794	7.63E-08	1.08E-06	7.66652
C4ORF19	-1.25339	6.354428	-5.72348	8.93E-08	1.25E-06	7.515106
E2F8	-1.07403	6.2394	-5.71939	9.10E-08	1.27E-06	7.497182
ZBTB16	1.242733	6.02467	5.715887	9.24E-08	1.28E-06	7.48182
SCN7A	1.127797	4.302714	5.713765	9.33E-08	1.29E-06	7.472521
CEP55	-1.11776	6.601234	-5.68106	1.08E-07	1.47E-06	7.329414
PLS1	-1.43823	9.144583	-5.67955	1.09E-07	1.48E-06	7.322817
POF1B	-1.01559	5.288489	-5.67867	1.09E-07	1.48E-06	7.318956
SMIM6	-1.09723	6.741731	-5.67864	1.09E-07	1.48E-06	7.318863
TMPRSS3	-1.20261	6.671954	-5.67141	1.13E-07	1.52E-06	7.287277
LINC0070[illegible]	1.085526	4.119873	5.660024	1.19E-07	1.59E-06	7.237607
ADTRP	-1.35616	6.074976	-5.65066	1.24E-07	1.65E-06	7.196813
FABP4	1.299695	6.013218	5.646246	1.27E-07	1.67E-06	7.177579
ITGBL1	1.215208	6.011996	5.643995	1.28E-07	1.69E-06	7.167778
MLLT11	1.066804	7.35209	5.64212	1.29E-07	1.70E-06	7.159614
CCDC68	-1.01988	6.974943	-5.62477	1.40E-07	1.82E-06	7.084183
PRSS3	-1.30995	8.553439	-5.62156	1.42E-07	1.85E-06	7.070238
TSPAN1	-1.47244	9.396813	-5.59206	1.62E-07	2.06E-06	6.942283
SLC6A14	-2.17218	6.03704	-5.59023	1.63E-07	2.07E-06	6.934339
MYO1A	-1.15304	6.366786	-5.53759	2.07E-07	2.54E-06	6.70699
HTR2B	1.228509	5.497957	5.526032	2.18E-07	2.65E-06	6.657237
HMCN1	1.15732	6.225081	5.496982	2.48E-07	2.97E-06	6.532438
FN1	1.001459	8.976845	5.49569	2.49E-07	2.98E-06	6.526899
TOX3	-1.14177	6.733509	-5.44918	3.06E-07	3.58E-06	6.327904

C3ORF70	1.0199	6.182846	5.425871	3.40E-07	3.92E-06	6.228524
FABP1	-1.88812	5.698993	-5.4026	3.76E-07	4.29E-06	6.129566
PDGFRL	1.146579	5.970366	5.400478	3.80E-07	4.32E-06	6.120549
TRIM31	-1.03117	5.459674	-5.39967	3.81E-07	4.33E-06	6.117119
MAP7D2	-1.40526	4.856075	-5.3943	3.91E-07	4.43E-06	6.094311
SFRP1	1.167693	5.575803	5.392063	3.94E-07	4.46E-06	6.084832
KLF5	-1.22759	8.506565	-5.37895	4.18E-07	4.68E-06	6.02923
TAFA4	1.140793	3.366589	5.361471	4.51E-07	4.99E-06	5.955257
BCL2L15	-1.00257	6.273002	-5.35916	4.56E-07	5.04E-06	5.945506
FXYD3	-1.11273	9.171945	-5.35285	4.69E-07	5.16E-06	5.918807
REEP1	1.204306	7.000576	5.349599	4.75E-07	5.22E-06	5.905089
VLDLR	1.012647	5.981777	5.346464	4.82E-07	5.28E-06	5.891854
MAL2	-1.43839	9.543304	-5.34531	4.85E-07	5.30E-06	5.886969
SGPP2	-1.1752	7.110029	-5.33752	5.01E-07	5.45E-06	5.854114
MEP1A	-2.13076	6.047242	-5.33424	5.09E-07	5.51E-06	5.840296
TPSB2	1.007253	9.304243	5.321431	5.38E-07	5.78E-06	5.786319
XDH	-1.05401	5.834292	-5.31886	5.44E-07	5.84E-06	5.775498
LYPD6B	-1.22747	6.109619	-5.30247	5.85E-07	6.20E-06	5.70657
LCN2	-2.04621	10.19807	-5.30068	5.89E-07	6.24E-06	5.699072
GDA	-1.11826	5.833596	-5.28904	6.20E-07	6.51E-06	5.650178
SFRP4	1.678509	7.209401	5.288682	6.21E-07	6.52E-06	5.648692
FAM110C	-1.30392	6.403371	-5.27694	6.54E-07	6.82E-06	5.599456
TAC1	1.28111	3.836614	5.275855	6.57E-07	6.85E-06	5.594927
SIX2	1.081324	5.305446	5.275418	6.58E-07	6.85E-06	5.593095
ERICH5	-1.02284	3.977499	-5.26959	6.75E-07	7.00E-06	5.568674
FA2H	-1.0168	6.541506	-5.25686	7.13E-07	7.36E-06	5.515463
S100A14	-1.16337	7.837784	-5.23288	7.92E-07	8.04E-06	5.415344
NUF2	-1.11805	6.117048	-5.20171	9.06E-07	9.07E-06	5.285645
RAB25	-1.15374	9.262357	-5.19853	9.19E-07	9.18E-06	5.272447
CYP3A5	-1.21999	7.003758	-5.19476	9.34E-07	9.32E-06	5.256821
DLGAP5	-1.10143	6.329678	-5.17613	1.01E-06	1.00E-05	5.179571
IL33	1.214255	6.991852	5.156785	1.10E-06	1.08E-05	5.099562
PROM1	-1.66529	8.853104	-5.15666	1.10E-06	1.08E-05	5.099046
CCL19	1.37413	8.436761	5.153329	1.12E-06	1.09E-05	5.085286
SFN	-1.12144	9.163391	-5.12504	1.26E-06	1.20E-05	4.968666
RNF128	-1.52776	8.070689	-5.10411	1.38E-06	1.30E-05	4.882614
NBEA	1.071166	6.074159	5.098691	1.41E-06	1.32E-05	4.860368
ANKRD22	-1.02756	7.574728	-5.09022	1.46E-06	1.37E-05	4.825623
C15ORF48	-1.50914	9.284396	-5.08627	1.49E-06	1.39E-05	4.809437
CFD	1.234791	8.865942	5.082773	1.51E-06	1.40E-05	4.795116
CCN1	1.018674	9.671814	5.082145	1.52E-06	1.41E-05	4.792545
CDC6	-1.02169	4.975853	-5.04368	1.79E-06	1.61E-05	4.635425
RNF186	-1.07175	5.436793	-5.03766	1.83E-06	1.65E-05	4.610874
LINC02535	-1.10647	4.28827	-4.9978	2.17E-06	1.91E-05	4.448963
RCAN2	1.011076	8.101175	4.992925	2.21E-06	1.94E-05	4.429204
ANKRD40(	-1.16518	5.360351	-4.98652	2.28E-06	1.99E-05	4.40329
FAM83D	1.059568	8.766019	4.969447	2.45E-06	2.12E-05	4.334258
CMA1	1.122111	4.476806	4.956723	2.58E-06	2.22E-05	4.282925
CPE	1.139297	8.150695	4.932343	2.86E-06	2.43E-05	4.184791
SOSTDC1	-1.75948	6.480663	-4.92392	2.96E-06	2.51E-05	4.15096
AGR2	-1.42579	10.87664	-4.92239	2.98E-06	2.52E-05	4.144831
RAB27B	-1.03515	6.323104	-4.88249	3.52E-06	2.92E-05	3.985086
MUCL3	-2.21709	7.045584	-4.86177	3.84E-06	3.14E-05	3.90249
MGAM	-1.00536	4.380591	-4.85907	3.88E-06	3.17E-05	3.891732
C1ORF116	-1.0379	7.360271	-4.85437	3.96E-06	3.22E-05	3.873033
CKB	1.126851	8.018839	4.848483	4.06E-06	3.29E-05	3.849613
CES1	1.143682	6.879975	4.827511	4.43E-06	3.55E-05	3.766372
HPGD	-1.38103	7.619788	-4.82615	4.45E-06	3.57E-05	3.760986

MAMDC2	1.290843	5.944371	4.803309	4.90E-06	3.86E-05	3.670597
RBP2	-1.4509	4.631149	-4.76403	5.76E-06	4.44E-05	3.515834
ASB5	1.231707	3.832574	4.733773	6.52E-06	4.95E-05	3.397182
VILL	-1.02597	7.618648	-4.70691	7.28E-06	5.45E-05	3.292242
CDH17	-1.867	8.390081	-4.65608	8.95E-06	6.55E-05	3.094833
CYP2C18	-1.50894	6.31906	-4.64509	9.36E-06	6.81E-05	3.052298
APOBEC1	-1.15956	5.353096	-4.61377	1.06E-05	7.58E-05	2.931548
GCNT3	-1.41322	8.792451	-4.60491	1.10E-05	7.82E-05	2.897504
C6ORF58	-1.77944	5.3372	-4.59131	1.16E-05	8.18E-05	2.845302
PLAC8	-1.343	9.505762	-4.58767	1.18E-05	8.29E-05	2.831354
THBS2	1.12752	8.765694	4.583914	1.20E-05	8.40E-05	2.816963
CCL20	-1.75722	7.456122	-4.58306	1.20E-05	8.42E-05	2.81369
GPA33	-1.31738	6.552469	-4.57811	1.23E-05	8.56E-05	2.794729
PRSS2	-1.21401	7.301963	-4.56562	1.29E-05	8.97E-05	2.746995
SLPI	-1.17457	9.146116	-4.54034	1.43E-05	9.82E-05	2.650608
GKN1	-3.39669	8.330699	-4.53846	1.44E-05	9.87E-05	2.643476
AKR1C3	-1.10111	10.21274	-4.5248	1.52E-05	0.000103	2.591546
SCGB2A1	-1.54424	5.112968	-4.52181	1.54E-05	0.000104	2.580228
PLLP	-1.00823	7.936103	-4.51575	1.58E-05	0.000107	2.55721
GKN2	-2.99557	8.186266	-4.50092	1.67E-05	0.000112	2.501064
GREM1	1.255162	10.39347	4.490937	1.74E-05	0.000116	2.46334
CDH1	-1.20069	8.026646	-4.4676	1.91E-05	0.000126	2.375369
C3ORF85	-1.37783	3.588476	-4.44447	2.09E-05	0.000137	2.288492
DUOX2	-1.58502	6.471609	-4.43674	2.15E-05	0.000141	2.259499
FOXA3	-1.05206	7.66193	-4.42508	2.26E-05	0.000147	2.215885
CKMT1B	-1.1199	8.463065	-4.42336	2.27E-05	0.000148	2.209474
EPCAM	-1.24247	11.34495	-4.41445	2.35E-05	0.000152	2.176183
ADGRG7	-1.29473	4.811096	-4.39403	2.55E-05	0.000163	2.100088
PDZK1IP1	-1.22413	8.044053	-4.3642	2.86E-05	0.00018	1.989407
BAMBI	-1.04955	6.349237	-4.31954	3.41E-05	0.00021	1.824661
KIAA1324	-1.15635	7.106662	-4.30099	3.66E-05	0.000223	1.756588
GP2	-1.03208	5.534031	-4.29647	3.73E-05	0.000226	1.74005
GBA3	-1.21119	4.094497	-4.28391	3.91E-05	0.000237	1.694103
PIGR	-1.49202	8.992724	-4.27408	4.06E-05	0.000244	1.65821
S100P	-1.34538	11.26535	-4.26687	4.18E-05	0.00025	1.631931
LRRC31	-1.10319	5.360588	-4.23001	4.81E-05	0.000282	1.498062
NMU	-1.29078	6.673378	-4.22495	4.91E-05	0.000287	1.479726
CD24	-1.14779	10.72747	-4.22413	4.92E-05	0.000287	1.476769
FNDC1	1.405827	6.11624	4.214163	5.11E-05	0.000297	1.440752
CLRN3	-1.4614	7.595513	-4.19018	5.60E-05	0.000322	1.354312
FOXQ1	-1.19841	10.6089	-4.17796	5.87E-05	0.000335	1.310443
SULT1C2	-1.26906	7.496508	-4.17059	6.03E-05	0.000344	1.284013
CAPN8	-1.37929	7.982791	-4.16402	6.19E-05	0.000352	1.260461
SI	-2.15532	5.421788	-4.11002	7.59E-05	0.000419	1.068071
TFF2	-2.2094	10.03394	-4.10834	7.63E-05	0.000421	1.062128
BPIFB1	-1.84485	7.998931	-4.02218	0.000105	0.000558	0.759059
BCAS1	-1.05417	6.725661	-4.01216	0.000109	0.000577	0.724102
MUC5AC	-1.59684	7.994603	-3.98985	0.000119	0.000617	0.646551
APOB	-1.01412	4.216989	-3.96421	0.00013	0.000671	0.557836
CLDN3	-1.14532	7.641241	-3.95465	0.000135	0.000691	0.524859
UPK1B	-1.23958	5.963816	-3.92606	0.00015	0.000757	0.426618
SPINK1	-1.50783	11.26829	-3.92246	0.000152	0.000765	0.414292
ZG16B	-1.07622	7.82987	-3.88347	0.000175	0.000866	0.281235
REG3A	-1.2556	6.169596	-3.8799	0.000177	0.000876	0.269105
VSIG1	-1.67138	6.693189	-3.87956	0.000177	0.000876	0.26797
CA9	-1.15691	6.606582	-3.86717	0.000186	0.000909	0.22592
AOC1	-1.26834	8.2692	-3.83911	0.000205	0.000988	0.131106
C19ORF33	-1.01805	9.018954	-3.83307	0.00021	0.001007	0.110758

AZGP1	-1.38952	6.618803	-3.81798	0.000222	0.001054	0.060068
SMIM31	-1.43595	5.772545	-3.80717	0.00023	0.001089	0.02383
SDR16C5	-1.21473	6.863969	-3.80324	0.000234	0.001103	0.01067
ARL14	-1.28393	7.844107	-3.78918	0.000246	0.00115	-0.0363
PSCA	-1.77199	7.058609	-3.74363	0.000289	0.001326	-0.18754
CA2	-1.51018	10.14382	-3.68466	0.000355	0.001588	-0.38123
CAPN9	-1.07099	6.02036	-3.68288	0.000357	0.001595	-0.38704
PCK1	-1.37705	5.82872	-3.66017	0.000387	0.001706	-0.46098
TFF1	-1.8332	10.98625	-3.63318	0.000425	0.001845	-0.54837
C9ORF152	-1.07435	7.479209	-3.63254	0.000426	0.001848	-0.55045
ACE2	-1.31172	5.236016	-3.63121	0.000428	0.001856	-0.55475
BTNL8	-1.0127	6.557123	-3.5886	0.000495	0.002114	-0.69162
PI3	-1.30124	7.649623	-3.56889	0.00053	0.002243	-0.7545
VSIG2	-1.01819	7.202391	-3.56116	0.000544	0.002294	-0.77911
TSPAN8	-1.04032	12.27822	-3.55353	0.000559	0.002347	-0.80332
ADIPOQ	1.127997	4.040151	3.534912	0.000595	0.002479	-0.86228
TM4SF20	-1.41188	6.547359	-3.52782	0.00061	0.002532	-0.88466
MMP12	-1.4645	8.202721	-3.52573	0.000614	0.002547	-0.89124
MUC6	-1.0283	6.178385	-3.41657	0.000886	0.0035	-1.23115
MSMB	-1.87643	6.359449	-3.40139	0.000932	0.003654	-1.27774
HSD17B2	-1.26321	7.897431	-3.39371	0.000956	0.003736	-1.30126
LGALS4	-1.12025	10.94699	-3.39162	0.000962	0.003757	-1.30766
CXCL17	-1.34294	7.629528	-3.35461	0.001087	0.004184	-1.42027
GUCY2C	-1.04641	6.350085	-3.35275	0.001094	0.004203	-1.42588
AGR3	-1.41198	9.099459	-3.30602	0.001274	0.0048	-1.56658
LIPF	-2.49624	8.138467	-3.29542	0.001318	0.004939	-1.59826
AKR1B10	-1.73209	9.061856	-3.28213	0.001376	0.005122	-1.63789
GC	-1.14382	4.302979	-3.26147	0.001471	0.005423	-1.69919
TM4SF4	-1.26396	6.667193	-3.2238	0.00166	0.006009	-1.81018
CLDN18	-1.36355	9.246824	-3.15885	0.002039	0.007167	-1.99902
KRT20	-1.73527	7.333686	-3.11942	0.002307	0.007964	-2.11209
SST	-1.5027	6.602526	-3.11126	0.002366	0.008135	-2.13534
DAZ4	-1.74937	5.256719	-2.92454	0.00418	0.01319	-2.65332
DAZ2	-1.49269	5.249326	-2.87442	0.004849	0.014989	-2.78771
DMBT1	-1.0575	9.032916	-2.829	0.005539	0.016795	-2.90777
DAZ1	-1.37091	4.984634	-2.79178	0.00617	0.018415	-3.00492
TCN1	-1.25921	7.894796	-2.77226	0.006526	0.019317	-3.05543
LTF	-1.20137	8.609734	-2.75575	0.006842	0.020107	-3.09789
PGC	-1.68493	10.16105	-2.71546	0.007673	0.022189	-3.20061
REG1A	-1.52579	9.70405	-2.71313	0.007724	0.022317	-3.20652
DEFA5	-1.01961	4.588091	-2.52407	0.013009	0.034678	-3.67039
REG4	-1.13867	7.770066	-2.49532	0.014049	0.036971	-3.73833
CBLIF	-1.44336	5.624854	-2.46414	0.015262	0.039699	-3.81123
CTSE	-1.02042	10.33819	-2.43738	0.016375	0.042126	-3.87312
