# Supplementary material for: Identification of GGT5 as a Novel Prognostic Biomarker for Gastric Cancer and its Correlation With Immune Cell Infiltration
Source: Front Genet. 2022 Mar 18;13:810292. doi: 10.3389/fgene.2022.810292 (PMC8971189; doi:10.3389/fgene.2022.810292)
Supplement: Supplementary file 3 [file DataSheet6.PDF]

ONTOLOG	ID	Descriptior	GeneRatio	BgRatio	pvalue	p.adjust	qvalue	geneID	Count
BP	GO:003019	extracellula	127/1638	368/18670	3.45E-44	9.98E-41	7.65E-41	A2M/AEBP	127
BP	GO:004306	extracellula	137/1638	422/18670	3.78E-44	9.98E-41	7.65E-41	A2M/AEBP	137
BP	GO:000695	humoral in	88/1638	356/18670	1.36E-19	2.38E-16	1.83E-16	A2M/BPI/S	88
BP	GO:007237	protein act	57/1638	198/18670	2.72E-16	3.59E-13	2.75E-13	A2M/APOI	57
BP	GO:007026	cornificatic	41/1638	112/18670	4.14E-16	4.37E-13	3.35E-13	CSTA/DSC	41
BP	GO:000693	muscle cor	79/1638	360/18670	1.33E-14	1.17E-11	8.97E-12	ACTA2/AC	79
BP	GO:000695	compleme	50/1638	175/18670	2.56E-14	1.93E-11	1.48E-11	A2M/SERP	50
BP	GO:000252	acute infla	57/1638	220/18670	4.34E-14	2.86E-11	2.20E-11	A2M/ADCY	57
BP	GO:004358	skin develc	86/1638	419/18670	5.24E-14	3.07E-11	2.35E-11	COL1A1/C	86
BP	GO:003563	multicellula	54/1638	204/18670	7.81E-14	4.12E-11	3.16E-11	ANK2/ATP	54
BP	GO:000301	muscle sys	91/1638	465/18670	1.81E-13	8.67E-11	6.65E-11	ACTA2/AC	91
BP	GO:005148	regulation	76/1638	357/18670	2.25E-13	9.87E-11	7.57E-11	ADCY5/AD	76
BP	GO:003142	keratinizati	56/1638	224/18670	3.65E-13	1.48E-10	1.14E-10	CSTA/DSC	56
BP	GO:003021	keratinocy	68/1638	305/18670	4.27E-13	1.53E-10	1.17E-10	CSTA/CTSI	68
BP	GO:000720	positive re	70/1638	319/18670	4.35E-13	1.53E-10	1.17E-10	ADCY5/AD	70
BP	GO:000150	ossificatior	81/1638	398/18670	4.63E-13	1.53E-10	1.17E-10	AHSG/BMI	81
BP	GO:003158	cell-substr	74/1638	354/18670	1.21E-12	3.76E-10	2.88E-10	ACTN2/AP	74
BP	GO:006144	connective	62/1638	273/18670	1.98E-12	5.81E-10	4.46E-10	ACTA2/AN	62
BP	GO:000292	regulation	40/1638	134/18670	2.10E-12	5.83E-10	4.47E-10	A2M/SERP	40
BP	GO:000751	muscle org	81/1638	410/18670	2.37E-12	6.25E-10	4.80E-10	ACTC1/AD	81
BP	GO:000267	regulation	44/1638	159/18670	3.04E-12	7.40E-10	5.67E-10	A2M/ADCY	44
BP	GO:005121	cartilage d	52/1638	209/18670	3.09E-12	7.40E-10	5.67E-10	ANXA6/NK	52
BP	GO:000687	cellular cal	87/1638	458/18670	3.41E-12	7.83E-10	6.01E-10	ADCY5/AD	87
BP	GO:000695	compleme	40/1638	137/18670	4.59E-12	9.83E-10	7.54E-10	SERPING1/	40
BP	GO:005090	leukocyte	92/1638	499/18670	4.66E-12	9.83E-10	7.54E-10	ADD2/APC	92
BP	GO:007250	cellular div	91/1638	493/18670	5.71E-12	1.16E-09	8.88E-10	ADCY5/AD	91
BP	GO:000991	epidermal	73/1638	358/18670	6.07E-12	1.17E-09	8.95E-10	CSTA/CTSI	73
BP	GO:000245	humoral in	42/1638	150/18670	6.20E-12	1.17E-09	8.95E-10	SERPING1/	42
BP	GO:005507	calcium ior	88/1638	471/18670	6.53E-12	1.19E-09	9.11E-10	ADCY5/AD	88
BP	GO:190352	regulation	64/1638	297/18670	1.02E-11	1.80E-09	1.38E-09	AGTR1/AG	64
BP	GO:000854	epidermis	86/1638	464/18670	1.77E-11	3.01E-09	2.31E-09	CSTA/CCN	86
BP	GO:003044	regulation	35/1638	115/18670	2.71E-11	4.46E-09	3.42E-09	A2M/SERP	35
BP	GO:003019	collagen fil	23/1638	54/18670	3.18E-11	5.09E-09	3.90E-09	AEBP1/CO	23
BP	GO:200025	regulation	35/1638	116/18670	3.55E-11	5.51E-09	4.23E-09	A2M/SERP	35
BP	GO:006133	cardiac cor	40/1638	146/18670	4.12E-11	6.21E-09	4.76E-09	ANK2/ATP	40
BP	GO:009874	cell-cell ac	59/1638	273/18670	5.81E-11	8.51E-09	6.53E-09	APOA1/CE	59
BP	GO:009917	regulation	81/1638	437/18670	6.88E-11	9.81E-09	7.52E-09	ADCYAP1/	81
BP	GO:006053	muscle tiss	77/1638	408/18670	8.42E-11	1.17E-08	8.97E-09	ACTC1/AC	77
BP	GO:009028	regulation	61/1638	292/18670	1.20E-10	1.62E-08	1.24E-08	AGTR2/CA	61
BP	GO:000801	regulation	55/1638	251/18670	1.49E-10	1.89E-08	1.45E-08	AGTR2/AN	55
BP	GO:005080	modulatior	80/1638	436/18670	1.51E-10	1.89E-08	1.45E-08	ADCYAP1/	80
BP	GO:004274	defense re	66/1638	330/18670	1.51E-10	1.89E-08	1.45E-08	ADGRB1/B	66
BP	GO:006004	heart conti	59/1638	280/18670	1.69E-10	2.07E-08	1.59E-08	ACTC1/AG	59
BP	GO:000301	heart proc	60/1638	290/18670	2.55E-10	3.05E-08	2.34E-08	ACTC1/AG	60
BP	GO:001814	peptide cro	23/1638	60/18670	4.08E-10	4.79E-08	3.67E-08	BGN/COL3	23
BP	GO:000716	cell-matrix	50/1638	225/18670	6.08E-10	6.97E-08	5.35E-08	ACTN2/AP	50
BP	GO:004239	regulation	78/1638	434/18670	6.99E-10	7.82E-08	6.00E-08	ACTN2/AD	78
BP	GO:000257	platelet de	35/1638	128/18670	7.12E-10	7.82E-08	6.00E-08	A2M/ACTN	35
BP	GO:001046	negative re	55/1638	262/18670	8.09E-10	8.69E-08	6.66E-08	A2M/AHSG	55
BP	GO:001648	protein pro	64/1638	328/18670	8.24E-10	8.69E-08	6.66E-08	A2M/AEBP	64
BP	GO:000150	action pote	35/1638	133/18670	2.18E-09	2.25E-07	1.73E-07	ANK2/ATP	35
BP	GO:001470	striated mu	71/1638	390/18670	2.32E-09	2.35E-07	1.80E-07	ACTC1/AC	71
BP	GO:001993	second-me	77/1638	439/18670	2.81E-09	2.79E-07	2.14E-07	ADCY2/AD	77
BP	GO:005080	synapse or	73/1638	408/18670	3.01E-09	2.92E-07	2.24E-07	ADD2/ADC	73
BP	GO:007061	regulation	42/1638	180/18670	3.05E-09	2.92E-07	2.24E-07	A2M/SERP	42
BP	GO:001095	negative re	52/1638	250/18670	3.26E-09	3.07E-07	2.35E-07	A2M/AHSG	52
BP	GO:001095	regulation	71/1638	394/18670	3.63E-09	3.36E-07	2.58E-07	ACTN2/AD	71
BP	GO:190331	regulation	42/1638	182/18670	4.33E-09	3.94E-07	3.02E-07	A2M/SERP	42
BP	GO:004576	regulation	69/1638	383/18670	6.09E-09	5.44E-07	4.18E-07	AGTR1/AP	69
BP	GO:000691	phagocyto	26/1638	84/18670	6.35E-09	5.59E-07	4.28E-07	ADGRB1/C	26
BP	GO:001081	regulation	46/1638	215/18670	1.04E-08	8.97E-07	6.88E-07	APOA1/AP	46

BP	GO:00507:	regulation	81/1638	485/18670	1.11E-08	9.40E-07	7.21E-07	A2M/ADC'	81
BP	GO:00197:	antimicrob	32/1638	122/18670	1.15E-08	9.61E-07	7.37E-07	BPI/CTSG/	32
BP	GO:00020(	chondrocy	32/1638	123/18670	1.42E-08	1.17E-06	8.99E-07	ANXA6/NI	32
BP	GO:00426(	muscle cel	68/1638	385/18670	1.78E-08	1.45E-06	1.11E-06	ACTC1/AC	68
BP	GO:00076:	learning o	51/1638	256/18670	2.09E-08	1.67E-06	1.28E-06	ATP1A2/A	51
BP	GO:00071:	transmeml	63/1638	349/18670	2.57E-08	2.02E-06	1.55E-06	AFP/BMP3	63
BP	GO:00075(	digestion	34/1638	139/18670	2.73E-08	2.12E-06	1.63E-06	APOA1/AF	34
BP	GO:00508(	cognition	56/1638	296/18670	2.89E-08	2.21E-06	1.69E-06	ATP1A2/A	56
BP	GO:00603:	cell chemo	57/1638	304/18670	3.02E-08	2.28E-06	1.74E-06	AGTR1/CX	57
BP	GO:00108:	positive re	31/1638	121/18670	3.51E-08	2.61E-06	2.00E-06	APOA1/CD	31
BP	GO:00016	osteoblast	46/1638	225/18670	4.50E-08	3.30E-06	2.53E-06	BMP3/COL	46
BP	GO:00702!	actin-med	30/1638	116/18670	4.62E-08	3.32E-06	2.54E-06	ACTC1/AC	30
BP	GO:00160(	immunogl	45/1638	218/18670	4.66E-08	3.32E-06	2.54E-06	SERPING1/	45
BP	GO:00068:	potassium	48/1638	240/18670	4.75E-08	3.32E-06	2.54E-06	ACTN2/AD	48
BP	GO:00508!	B cell rece	32/1638	129/18670	4.87E-08	3.32E-06	2.54E-06	BLK/CD19/	32
BP	GO:00074(	axonogen	77/1638	468/18670	4.88E-08	3.32E-06	2.54E-06	APBB1/AD	77
BP	GO:00352(	organ grov	43/1638	204/18670	4.90E-08	3.32E-06	2.54E-06	AGTR2/AN	43
BP	GO:00604(	mesenchy	53/1638	278/18670	5.18E-08	3.46E-06	2.65E-06	ACTA2/AC	53
BP	GO:19013	regulation	71/1638	422/18670	6.60E-08	4.35E-06	3.34E-06	AGTR1/AP	71
BP	GO:00069:	phagocyto	30/1638	118/18670	7.01E-08	4.56E-06	3.50E-06	ADGRB1/C	30
BP	GO:00197:	B cell med	45/1638	221/18670	7.13E-08	4.58E-06	3.52E-06	SERPING1/	45
BP	GO:00072(	phospholi	27/1638	100/18670	8.19E-08	5.20E-06	3.99E-06	ACTN2/AG	27
BP	GO:00080:	cell recog	44/1638	215/18670	8.52E-08	5.35E-06	4.10E-06	ADGRB1/C	44
BP	GO:00432:	positive re	52/1638	275/18670	9.10E-08	5.64E-06	4.33E-06	ACTN2/AD	52
BP	GO:00718(	cellular po	44/1638	217/18670	1.13E-07	6.83E-06	5.24E-06	ACTN2/AN	44
BP	GO:00718(	potassium	44/1638	217/18670	1.13E-07	6.83E-06	5.24E-06	ACTN2/AN	44
BP	GO:00975:	myeloid le	43/1638	210/18670	1.17E-07	7.02E-06	5.38E-06	CALCA/CC	43
BP	GO:00017(	morphoge	41/1638	196/18670	1.24E-07	7.33E-06	5.62E-06	AGTR2/AR	41
BP	GO:00511	striated mu	54/1638	293/18670	1.25E-07	7.33E-06	5.62E-06	ACTC1/AC	54
BP	GO:00071!	neuron cel	10/1638	16/18670	1.29E-07	7.46E-06	5.72E-06	CEL/NCAM	10
BP	GO:00900!	regulation	47/1638	241/18670	1.44E-07	8.28E-06	6.35E-06	BMP3/CAV	47
BP	GO:19037:	regulation	22/1638	73/18670	1.56E-07	8.85E-06	6.79E-06	ANK2/ATP	22
BP	GO:00343:	cell junctio	53/1638	290/18670	2.16E-07	1.21E-05	9.28E-06	ACTN2/AN	53
BP	GO:00076:	learning	33/1638	145/18670	2.71E-07	1.50E-05	1.15E-05	ATP1A2/A	33
BP	GO:00030:	vascular pr	37/1638	173/18670	2.82E-07	1.54E-05	1.18E-05	ACTA2/AD	37
BP	GO:00902(	negative re	36/1638	166/18670	2.83E-07	1.54E-05	1.18E-05	AGTR2/CA	36
BP	GO:00516(	protein ma	66/1638	397/18670	2.99E-07	1.61E-05	1.23E-05	A2M/AEBF	66
BP	GO:00456(	regulation	30/1638	126/18670	3.32E-07	1.77E-05	1.36E-05	GDF10/GL	30
BP	GO:00302:	regulation	41/1638	203/18670	3.37E-07	1.78E-05	1.36E-05	AHSG/COI	41
BP	GO:00509(	leukocyte t	12/1638	25/18670	3.52E-07	1.82E-05	1.40E-05	ADD2/CX3	12
BP	GO:00973(	response t	45/1638	233/18670	3.53E-07	1.82E-05	1.40E-05	ACTC1/AD	45
BP	GO:19040(	regulation	59/1638	342/18670	3.56E-07	1.82E-05	1.40E-05	ACTN2/AP	59
BP	GO:00611:	morphoge	38/1638	182/18670	3.73E-07	1.89E-05	1.45E-05	AGTR2/AR	38
BP	GO:00347(	regulation	76/1638	483/18670	3.79E-07	1.91E-05	1.46E-05	ACTN2/AP	76
BP	GO:00990:	plasma me	30/1638	127/18670	3.99E-07	1.98E-05	1.52E-05	ADGRB1/C	30
BP	GO:00525	regulation	72/1638	452/18670	4.78E-07	2.35E-05	1.81E-05	A2M/AHS(	72
BP	GO:00109:	cellular co	28/1638	115/18670	4.97E-07	2.38E-05	1.83E-05	ACTC1/AC	28
BP	GO:00103:	membrane	31/1638	135/18670	5.01E-07	2.38E-05	1.83E-05	ADGRB1/C	31
BP	GO:00509:	positive re	31/1638	135/18670	5.01E-07	2.38E-05	1.83E-05	CCR4/CCR	31
BP	GO:00990(	postsynap	13/1638	30/18670	5.01E-07	2.38E-05	1.83E-05	CBLN1/CE	13
BP	GO:00550(	muscle cel	38/1638	186/18670	6.64E-07	3.13E-05	2.40E-05	ACTC1/AC	38
BP	GO:00974(	neuron pro	50/1638	277/18670	6.93E-07	3.24E-05	2.48E-05	CSF1R/EPH	50
BP	GO:00303:	lung devel	36/1638	172/18670	7.05E-07	3.26E-05	2.50E-05	CRH/CCN2	36
BP	GO:00860(	cell comm	18/1638	56/18670	7.18E-07	3.28E-05	2.51E-05	ANK2/ATP	18
BP	GO:00351!	regulation	32/1638	144/18670	7.21E-07	3.28E-05	2.51E-05	ACTA2/AD	32
BP	GO:00604(	calcium io	34/1638	158/18670	7.27E-07	3.28E-05	2.51E-05	ADCYAP1F	34
BP	GO:00305(	leukocyte (	43/1638	224/18670	7.57E-07	3.38E-05	2.59E-05	CXCR5/CA	43
BP	GO:00550(	striated mu	36/1638	173/18670	8.17E-07	3.62E-05	2.78E-05	ACTC1/AC	36
BP	GO:00226:	extracellul	22/1638	80/18670	9.04E-07	3.97E-05	3.05E-05	A2M/CMA	22
BP	GO:00197:	calcium-m	42/1638	218/18670	9.17E-07	4.00E-05	3.07E-05	AGTR1/AN	42
BP	GO:00302(	intestinal c	9/1638	15/18670	9.26E-07	4.00E-05	3.07E-05	APOA1/AF	9
BP	GO:00024	lymphocyt	59/1638	352/18670	9.55E-07	4.09E-05	3.14E-05	SERPING1/	59

BP	GO:00246	adaptive ir	60/1638	361/18670	1.03E-06	4.39E-05	3.37E-05	SERPING1/	60
BP	GO:00550	sodium ior	17/1638	52/18670	1.11E-06	4.69E-05	3.60E-05	AGTR1/AG	17
BP	GO:00860	cardiac mu	20/1638	69/18670	1.13E-06	4.73E-05	3.63E-05	ANK2/ATP	20
BP	GO:00442	lipid diges	10/1638	19/18670	1.16E-06	4.82E-05	3.69E-05	APOA1/AF	10
BP	GO:00605	respiratory	39/1638	198/18670	1.25E-06	5.14E-05	3.94E-05	ALDH1A3/	39
BP	GO:00303	respiratory	36/1638	176/18670	1.26E-06	5.14E-05	3.94E-05	CRH/CCN2	36
BP	GO:00741	synapse as	36/1638	177/18670	1.45E-06	5.86E-05	4.50E-05	ADD2/AD(	36
BP	GO:00741	axon guida	49/1638	276/18670	1.47E-06	5.91E-05	4.54E-05	CSF1R/EPH	49
BP	GO:00329	collagen m	27/1638	115/18670	1.70E-06	6.78E-05	5.20E-05	COL1A1/C	27
BP	GO:00604	cytosolic c	35/1638	171/18670	1.73E-06	6.85E-05	5.26E-05	ADCYAP1F	35
BP	GO:00352	regulation	31/1638	143/18670	1.87E-06	7.27E-05	5.57E-05	ACTA2/AD	31
BP	GO:00508	regulation	31/1638	143/18670	1.87E-06	7.27E-05	5.57E-05	ACTA2/AD	31
BP	GO:00977	regulation	31/1638	143/18670	1.87E-06	7.27E-05	5.57E-05	ACTA2/AD	31
BP	GO:00860	regulation	14/1638	38/18670	1.94E-06	7.48E-05	5.74E-05	ANK2/CAC	14
BP	GO:00424	hormone r	43/1638	232/18670	2.00E-06	7.64E-05	5.86E-05	ADH1B/AD	43
BP	GO:00161	diterpenoi	26/1638	110/18670	2.29E-06	8.70E-05	6.68E-05	ADH1B/AD	26
BP	GO:19908	response t	24/1638	97/18670	2.35E-06	8.79E-05	6.74E-05	CXCR5/CC	24
BP	GO:19908	cellular res	24/1638	97/18670	2.35E-06	8.79E-05	6.74E-05	CXCR5/CC	24
BP	GO:00300	actin filam	30/1638	138/18670	2.57E-06	9.44E-05	7.24E-05	ACTC1/AC	30
BP	GO:00152	retinoid m	25/1638	104/18670	2.57E-06	9.44E-05	7.24E-05	ADH1B/AD	25
BP	GO:00488	sensory sy	60/1638	371/18670	2.58E-06	9.44E-05	7.24E-05	ALDH1A3/	60
BP	GO:00069	striated mu	35/1638	174/18670	2.63E-06	9.44E-05	7.24E-05	ACTC1/AN	35
BP	GO:00016	urogenital	55/1638	330/18670	2.64E-06	9.44E-05	7.24E-05	ACTA2/AG	55
BP	GO:00487	mesenchyr	41/1638	219/18670	2.64E-06	9.44E-05	7.24E-05	ANXA6/CC	41
BP	GO:00458	negative re	59/1638	363/18670	2.65E-06	9.44E-05	7.24E-05	A2M/AHS(	59
BP	GO:00903	positive re	18/1638	61/18670	2.90E-06	0.000103	7.87E-05	APOH/CD3	18
BP	GO:00071	homophilic	34/1638	168/18670	3.15E-06	0.000111	8.49E-05	CDH9/CDH	34
BP	GO:00901	negative re	28/1638	126/18670	3.49E-06	0.000122	9.35E-05	CAV1/CIDI	28
BP	GO:00860	cardiac mu	17/1638	56/18670	3.54E-06	0.000123	9.42E-05	ANK2/CAC	17
BP	GO:00068	calcium ior	67/1638	434/18670	3.60E-06	0.000123	9.46E-05	ADCYAP1F	67
BP	GO:00506	epithelial c	67/1638	434/18670	3.60E-06	0.000123	9.46E-05	AGTR1/AP	67
BP	GO:00310	actomyosi	38/1638	199/18670	3.72E-06	0.000126	9.65E-05	ACTC1/AC	38
BP	GO:00519	regulation	25/1638	106/18670	3.72E-06	0.000126	9.65E-05	ADGRB1/A	25
BP	GO:00860	cardiac mu	20/1638	74/18670	3.76E-06	0.000126	9.69E-05	ANK2/ATP	20
BP	GO:00988	intestinal li	9/1638	17/18670	3.82E-06	0.000128	9.78E-05	APOA1/AF	9
BP	GO:00610	negative re	12/1638	30/18670	3.86E-06	0.000128	9.82E-05	NKX3-2/C	12
BP	GO:00226	gland mor	27/1638	120/18670	4.04E-06	0.000133	0.000102	AR/CAV1/	27
BP	GO:00717	response t	34/1638	170/18670	4.14E-06	0.000135	0.000103	COMP/NK	34
BP	GO:00717	cellular res	34/1638	170/18670	4.14E-06	0.000135	0.000103	COMP/NK	34
BP	GO:00451	cell fate co	47/1638	270/18670	4.17E-06	0.000135	0.000104	AR/NKX2-	47
BP	GO:00069	phagocyto	59/1638	369/18670	4.50E-06	0.000145	0.000111	AHSG/APC	59
BP	GO:00069	regulation	34/1638	171/18670	4.74E-06	0.000152	0.000116	ANK2/ANX	34
BP	GO:00020	regulation	24/1638	101/18670	5.03E-06	0.00016	0.000123	AGTR2/AN	24
BP	GO:00700	chemokine	22/1638	88/18670	5.10E-06	0.000161	0.000124	CXCR5/CC	22
BP	GO:00487	cardiac mu	42/1638	233/18670	5.44E-06	0.000171	0.000131	ACTC1/AC	42
BP	GO:00343	cell junctio	43/1638	241/18670	5.54E-06	0.000173	0.000133	ACTN2/AN	43
BP	GO:19040	positive re	30/1638	144/18670	6.39E-06	0.000198	0.000152	ACTN2/AP	30
BP	GO:00988	bone grow	15/1638	47/18670	6.64E-06	0.000205	0.000157	ANXA6/CC	15
BP	GO:00347	positive re	38/1638	204/18670	6.83E-06	0.000209	0.000161	ACTN2/AP	38
BP	GO:00030	heart morp	45/1638	259/18670	6.97E-06	0.000211	0.000162	ACTC1/CC	45
BP	GO:00902	regulation	45/1638	259/18670	6.97E-06	0.000211	0.000162	ANK2/ANX	45
BP	GO:01500	visual syst	58/1638	366/18670	7.15E-06	0.000215	0.000165	ALDH1A3/	58
BP	GO:00069	smooth mu	25/1638	110/18670	7.53E-06	0.000226	0.000173	ACTA2/AT	25
BP	GO:00510	positive re	19/1638	71/18670	7.65E-06	0.000228	0.000175	ADCYAP1F	19
BP	GO:00720	renal syste	49/1638	293/18670	8.26E-06	0.000245	0.000188	ACTA2/AG	49
BP	GO:00192	sensory pe	24/1638	104/18670	8.62E-06	0.000254	0.000195	ADCYAP1/	24
BP	GO:00601	positive re	19/1638	72/18670	9.55E-06	0.000278	0.000213	ADCYAP1F	19
BP	GO:00610	regulation	19/1638	72/18670	9.55E-06	0.000278	0.000213	NKX3-2/C	19
BP	GO:00105	positive re	16/1638	54/18670	9.66E-06	0.00028	0.000215	ADCYAP1F	16
BP	GO:00975	calcium ior	29/1638	140/18670	1.02E-05	0.000293	0.000225	APLNR/AN	29
BP	GO:00487	skeletal sys	42/1638	239/18670	1.04E-05	0.000299	0.00023	ANXA6/NK	42
BP	GO:00512	negative re	27/1638	126/18670	1.06E-05	0.000302	0.000232	APLNR/AN	27

BP	GO:200125	regulation	34/1638	178/18670	1.17E-05	0.000333	0.000255	ACTN2/AN	34
BP	GO:003023	myofibril a	19/1638	73/18670	1.19E-05	0.000334	0.000256	ACTC1/AC	19
BP	GO:005123	maintenan	53/1638	330/18670	1.19E-05	0.000334	0.000256	APLNR/AN	53
BP	GO:004856	digestive t	28/1638	134/18670	1.21E-05	0.000338	0.000259	NKX3-2/C	28
BP	GO:003090	forebrain c	59/1638	381/18670	1.23E-05	0.000341	0.000261	ADCYAP1/	59
BP	GO:000672	terpenoid	26/1638	120/18670	1.25E-05	0.000344	0.000264	ADH1B/AD	26
BP	GO:004512	cellular ext	17/1638	61/18670	1.27E-05	0.000349	0.000268	ADD2/CX3	17
BP	GO:005254	regulation	64/1638	425/18670	1.33E-05	0.000364	0.000279	A2M/AHS(	64
BP	GO:190004	regulation	20/1638	80/18670	1.35E-05	0.000368	0.000282	APOH/SER	20
BP	GO:000341	endochon(	14/1638	44/18670	1.40E-05	0.000378	0.00029	ANXA6/CC	14
BP	GO:000268	positive re	27/1638	128/18670	1.44E-05	0.000383	0.000294	CCR7/ELAI	27
BP	GO:005128	regulation	27/1638	128/18670	1.44E-05	0.000383	0.000294	APLNR/AN	27
BP	GO:200014	negative re	55/1638	349/18670	1.44E-05	0.000383	0.000294	AGTR2/AP	55
BP	GO:190303	positive re	19/1638	74/18670	1.47E-05	0.000388	0.000298	APOH/CD:	19
BP	GO:000689	receptor-r	51/1638	316/18670	1.50E-05	0.000397	0.000304	APOA1/AF	51
BP	GO:000170	endoderm	15/1638	50/18670	1.54E-05	0.000405	0.000311	COL4A2/C	15
BP	GO:005196	positive re	18/1638	68/18670	1.55E-05	0.000406	0.000311	ADGRB1/A	18
BP	GO:000341	growth pla	10/1638	24/18670	1.63E-05	0.000425	0.000326	ANXA6/CC	10
BP	GO:003033	negative re	53/1638	334/18670	1.68E-05	0.000435	0.000333	AGTR2/AP	53
BP	GO:000300	regionaliza	55/1638	351/18670	1.70E-05	0.000437	0.000335	AR/C3/NK:	55
BP	GO:006053	cartilage m	11/1638	29/18670	1.79E-05	0.000458	0.000351	COL6A1/C	11
BP	GO:003598	endoderm	14/1638	45/18670	1.87E-05	0.000474	0.000363	COL4A2/C	14
BP	GO:006004	cardiac mu	28/1638	137/18670	1.87E-05	0.000474	0.000363	ACTC1/AN	28
BP	GO:005195	regulation	22/1638	95/18670	1.90E-05	0.00048	0.000368	AGTR2/AT	22
BP	GO:000821	regulation	34/1638	182/18670	1.91E-05	0.000481	0.000369	ACTA2/AD	34
BP	GO:000718	adenylate	39/1638	221/18670	1.93E-05	0.000482	0.00037	ADCY2/AD	39
BP	GO:005076	positive re	69/1638	474/18670	1.96E-05	0.000483	0.00037	ADCYAP1/	69
BP	GO:001052	regulation	23/1638	102/18670	1.97E-05	0.000483	0.00037	ADCYAP1F	23
BP	GO:001583	amine tran	23/1638	102/18670	1.97E-05	0.000483	0.00037	AGTR2/AT	23
BP	GO:005192	positive re	26/1638	123/18670	1.98E-05	0.000483	0.00037	ADCYAP1F	26
BP	GO:007162	granulocyt	26/1638	123/18670	1.98E-05	0.000483	0.00037	CCR7/ITG/	26
BP	GO:000758	excretion	17/1638	63/18670	2.02E-05	0.000492	0.000377	AGTR1/AG	17
BP	GO:000182	kidney dev	46/1638	278/18670	2.04E-05	0.000493	0.000378	ACTA2/AG	46
BP	GO:009869	postsynapt	9/1638	20/18670	2.07E-05	0.000497	0.000381	CBLN1/GA	9
BP	GO:005134	negative re	68/1638	466/18670	2.07E-05	0.000497	0.000381	A2M/ADC'	68
BP	GO:009710	postsynapt	7/1638	12/18670	2.11E-05	0.000503	0.000386	CEL/NRXN	7
BP	GO:190401	positive re	40/1638	230/18670	2.16E-05	0.00051	0.000391	AGTR1/AP	40
BP	GO:002195	central ner	34/1638	183/18670	2.16E-05	0.00051	0.000391	PHOX2A/C	34
BP	GO:005120	sequesterii	27/1638	131/18670	2.23E-05	0.000526	0.000403	APLNR/AN	27
BP	GO:005124	regulation	70/1638	485/18670	2.27E-05	0.000532	0.000408	AXL/CAV1	70
BP	GO:005120	release of	26/1638	124/18670	2.30E-05	0.000536	0.000411	APLNR/AN	26
BP	GO:000722	integrin-m	23/1638	103/18670	2.33E-05	0.00054	0.000414	APOA1/CC	23
BP	GO:006057	morphoge	8/1638	16/18670	2.34E-05	0.00054	0.000414	AR/FGF10/	8
BP	GO:005512	digestive s	29/1638	146/18670	2.35E-05	0.000542	0.000416	NKX3-2/C	29
BP	GO:001993	cyclic-nucl	38/1638	215/18670	2.37E-05	0.000543	0.000416	ADCY2/AD	38
BP	GO:003300	muscle cel	41/1638	239/18670	2.39E-05	0.000543	0.000417	APOD/CNI	41
BP	GO:200125	positive re	18/1638	70/18670	2.39E-05	0.000543	0.000417	ACTN2/AN	18
BP	GO:002260	ovulation c	14/1638	46/18670	2.47E-05	0.000556	0.000426	AFP/ESR1/	14
BP	GO:009917	presynaps	14/1638	46/18670	2.47E-05	0.000556	0.000426	CBLN1/CE	14
BP	GO:005092	positive re	10/1638	25/18670	2.51E-05	0.000563	0.000432	CCR4/S1PI	10
BP	GO:000718	G protein-	43/1638	256/18670	2.59E-05	0.000577	0.000442	ADCY2/AD	43
BP	GO:003051	negative re	15/1638	52/18670	2.60E-05	0.000577	0.000442	DLX1/FBN	15
BP	GO:000341	chondrocy	11/1638	30/18670	2.60E-05	0.000577	0.000442	ANXA6/CC	11
BP	GO:004547	response t	26/1638	125/18670	2.66E-05	0.000587	0.00045	ACTC1/AD	26
BP	GO:003210	positive re	51/1638	323/18670	2.76E-05	0.000606	0.000465	AGTR1/AP	51
BP	GO:004206	gliogenesis	47/1638	290/18670	2.87E-05	0.000628	0.000482	ADCYAP1/	47
BP	GO:007156	cellular res	42/1638	249/18670	2.89E-05	0.000629	0.000483	CAV1/CIDI	42
BP	GO:005092	regulation	38/1638	217/18670	2.93E-05	0.000635	0.000487	CCR4/CCR	38
BP	GO:007251	divalent in	70/1638	489/18670	2.98E-05	0.000644	0.000494	ADCYAP1F	70
BP	GO:006104	regulation	29/1638	148/18670	3.07E-05	0.000661	0.000507	APOH/SER	29
BP	GO:003051	regulation	21/1638	91/18670	3.09E-05	0.000662	0.000508	DLX1/FBN	21
BP	GO:000602	aminoglyca	17/1638	65/18670	3.15E-05	0.000673	0.000516	BGN/VCAI	17

BP	GO:00601	regulation	22/1638	98/18670	3.19E-05	0.000678	0.00052	ADCYAP1F	22
BP	GO:00975	granulocyt	28/1638	141/18670	3.24E-05	0.000687	0.000527	CCR7/IL1R	28
BP	GO:00347	positive re	30/1638	156/18670	3.28E-05	0.000692	0.000531	ACTN2/AP	30
BP	GO:00902	positive re	12/1638	36/18670	3.39E-05	0.000713	0.000547	CD19/CCR	12
BP	GO:00457	positive re	60/1638	403/18670	3.45E-05	0.000722	0.000554	APOA1/CA	60
BP	GO:00708	divalent m	69/1638	483/18670	3.62E-05	0.000754	0.000578	ADCYAP1F	69
BP	GO:00305	BMP signa	30/1638	157/18670	3.72E-05	0.000762	0.000585	COMP/NK	30
BP	GO:00506	regulation	57/1638	378/18670	3.73E-05	0.000762	0.000585	AGTR1/AP	57
BP	GO:00301	positive re	10/1638	26/18670	3.76E-05	0.000762	0.000585	APOH/CD3	10
BP	GO:00509	regulation	10/1638	26/18670	3.76E-05	0.000762	0.000585	CCR4/S1PI	10
BP	GO:00607	prostate gl	10/1638	26/18670	3.76E-05	0.000762	0.000585	AR/ESR1/F	10
BP	GO:00995	synaptic m	10/1638	26/18670	3.76E-05	0.000762	0.000585	CDH9/SPA	10
BP	GO:19000	positive re	10/1638	26/18670	3.76E-05	0.000762	0.000585	APOH/CD3	10
BP	GO:00107	negative re	53/1638	344/18670	3.84E-05	0.000776	0.000595	ADCYAP1/	53
BP	GO:00026	regulation	35/1638	196/18670	3.91E-05	0.000788	0.000604	APOD/CCI	35
BP	GO:00301	regulation	19/1638	79/18670	3.96E-05	0.000792	0.000607	APOH/SER	19
BP	GO:00457	positive re	36/1638	204/18670	3.96E-05	0.000792	0.000607	AGTR1/AP	36
BP	GO:00487	branching	29/1638	150/18670	3.98E-05	0.000792	0.000607	AGTR2/AR	29
BP	GO:00068	cellular so	8/1638	17/18670	4.07E-05	0.000807	0.000619	AGTR2/AT	8
BP	GO:00016	eye develo	55/1638	362/18670	4.11E-05	0.000811	0.000622	ALDH1A3/	55
BP	GO:00456	positive re	56/1638	371/18670	4.23E-05	0.000831	0.000638	ADCYAP1/	56
BP	GO:00017	formation	25/1638	121/18670	4.24E-05	0.000832	0.000638	COL4A2/C	25
BP	GO:00302	bone mine	24/1638	114/18670	4.36E-05	0.000851	0.000653	AHSG/COI	24
BP	GO:00032	cardiac ve	18/1638	73/18670	4.40E-05	0.000856	0.000656	COL11A1/	18
BP	GO:00226	digestive s	22/1638	100/18670	4.43E-05	0.000856	0.000656	APOA1/AF	22
BP	GO:00608	artery deve	22/1638	100/18670	4.43E-05	0.000856	0.000656	APOB/COI	22
BP	GO:00860	membrane	12/1638	37/18670	4.63E-05	0.00089	0.000683	ANK2/ATP	12
BP	GO:00519	regulation	42/1638	254/18670	4.66E-05	0.000893	0.000685	ADCYAP1F	42
BP	GO:00715	response t	42/1638	255/18670	5.11E-05	0.000972	0.000745	CAV1/CIDI	42
BP	GO:00060	glycosamir	16/1638	61/18670	5.15E-05	0.000972	0.000745	BGN/VCAI	16
BP	GO:00341	heterotypi	16/1638	61/18670	5.15E-05	0.000972	0.000745	APOA1/DS	16
BP	GO:00990	presynaps	13/1638	43/18670	5.18E-05	0.000972	0.000745	CBLN1/CE	13
BP	GO:00435	positive re	11/1638	32/18670	5.21E-05	0.000972	0.000745	CD19/CCR	11
BP	GO:00519	positive re	11/1638	32/18670	5.21E-05	0.000972	0.000745	ADCYAP1/	11
BP	GO:00712	cellular res	11/1638	32/18670	5.21E-05	0.000972	0.000745	COL1A1/F	11
BP	GO:00860	cell-cell si	11/1638	32/18670	5.21E-05	0.000972	0.000745	ANK2/CAC	11
BP	GO:00032	cardiac ve	26/1638	130/18670	5.39E-05	0.001002	0.000768	COL11A1/	26
BP	GO:00508	positive re	10/1638	27/18670	5.50E-05	0.001014	0.000778	APOH/CD3	10
BP	GO:00723	blood coa	10/1638	27/18670	5.50E-05	0.001014	0.000778	A2M/APOI	10
BP	GO:00508	positive re	31/1638	168/18670	5.59E-05	0.001027	0.000788	ADCYAP1/	31
BP	GO:00073	pattern sp	64/1638	446/18670	5.98E-05	0.001095	0.00084	AR/NKX3-	64
BP	GO:00508	intestinal a	12/1638	38/18670	6.22E-05	0.001136	0.000871	APOA1/AF	12
BP	GO:19016	response t	34/1638	193/18670	6.65E-05	0.001206	0.000925	ADCY2/AD	34
BP	GO:00603	bone deve	37/1638	217/18670	6.66E-05	0.001206	0.000925	ANXA6/CC	37
BP	GO:00606	regulation	15/1638	56/18670	6.74E-05	0.001206	0.000925	AGTR2/AR	15
BP	GO:00034	chondrocy	8/1638	18/18670	6.77E-05	0.001206	0.000925	COL6A1/C	8
BP	GO:00034	growth pla	8/1638	18/18670	6.77E-05	0.001206	0.000925	COL6A1/C	8
BP	GO:00716	vocalizatio	8/1638	18/18670	6.77E-05	0.001206	0.000925	NRXN3/NI	8
BP	GO:00901	chondrocy	8/1638	18/18670	6.77E-05	0.001206	0.000925	COL6A1/C	8
BP	GO:00512	regulation	19/1638	82/18670	6.84E-05	0.001215	0.000932	APLNR/AN	19
BP	GO:00860	membrane	14/1638	50/18670	6.90E-05	0.001222	0.000937	ANK2/ATP	14
BP	GO:00426	ovulation c	17/1638	69/18670	7.19E-05	0.001267	0.000972	AFP/AXL/E	17
BP	GO:00600	regulation	27/1638	140/18670	7.56E-05	0.001329	0.001019	ADCYAP1/	27
BP	GO:00074	endoderm	18/1638	76/18670	7.77E-05	0.001344	0.001031	COL4A2/C	18
BP	GO:19902	neutrophil	24/1638	118/18670	7.79E-05	0.001344	0.001031	CCR7/IL1R	24
BP	GO:00343	triglyceride	7/1638	14/18670	7.79E-05	0.001344	0.001031	APOA1/AF	7
BP	GO:00363	sodium ior	7/1638	14/18670	7.79E-05	0.001344	0.001031	ATP1A2/A	7
BP	GO:00228	regulation	43/1638	268/18670	7.82E-05	0.001344	0.001031	ACTN2/AN	43
BP	GO:00427	fibrinolysis	10/1638	28/18670	7.89E-05	0.001344	0.001031	APOH/SER	10
BP	GO:00605	prostate gl	10/1638	28/18670	7.89E-05	0.001344	0.001031	AR/ESR1/F	10
BP	GO:00850	extracellul	10/1638	28/18670	7.89E-05	0.001344	0.001031	COL1A2/H	10
BP	GO:00032	cardiac ch	31/1638	171/18670	7.90E-05	0.001344	0.001031	ANK2/COI	31

BP	GO:00860	membrane	9/1638	23/18670	7.90E-05	0.001344	0.001031	ANK2/ATP	9
BP	GO:00324	regulation	42/1638	260/18670	8.06E-05	0.001362	0.001045	ACTN2/AN	42
BP	GO:19030	regulation	32/1638	179/18670	8.06E-05	0.001362	0.001045	APOH/SER	32
BP	GO:00481	regulation	33/1638	187/18670	8.16E-05	0.001375	0.001055	ADGRB1/C	33
BP	GO:00305	neutrophil	22/1638	104/18670	8.28E-05	0.001391	0.001067	CCR7/ITG	22
BP	GO:00107	regulation	20/1638	90/18670	8.35E-05	0.001397	0.001072	COL1A1/E	20
BP	GO:00432	regulation	22/1638	105/18670	9.62E-05	0.001604	0.00123	ACTN2/AD	22
BP	GO:00465	positive re	16/1638	64/18670	9.64E-05	0.001604	0.00123	APOA1/CC	16
BP	GO:00508	regulation	19/1638	84/18670	9.68E-05	0.001606	0.001232	APOH/SER	19
BP	GO:14011	export acr	11/1638	34/18670	9.81E-05	0.001622	0.001244	ATP1A2/A	11
BP	GO:00430	camera-ty	48/1638	314/18670	0.000106	0.001748	0.001341	ALDH1A3/	48
BP	GO:00034	growth pla	8/1638	19/18670	0.000108	0.001773	0.00136	COL6A1/C	8
BP	GO:00440	regulation	12/1638	40/18670	0.000109	0.001778	0.001364	APOA1/AP	12
BP	GO:00512	positive re	12/1638	40/18670	0.000109	0.001778	0.001364	APLNR/CA	12
BP	GO:00512	negative re	56/1638	384/18670	0.00011	0.001782	0.001367	AGTR2/AP	56
BP	GO:00323	regulation	14/1638	52/18670	0.00011	0.001783	0.001368	NKX3-2/C	14
BP	GO:00508	neuromus	14/1638	52/18670	0.00011	0.001783	0.001368	ADCY5/AL	14
BP	GO:00350	positive re	10/1638	29/18670	0.000111	0.001789	0.001372	APOA1/CC	10
BP	GO:00506	positive re	35/1638	206/18670	0.000111	0.001789	0.001372	AGTR1/AP	35
BP	GO:00076	locomotor	34/1638	198/18670	0.000112	0.001794	0.001376	ADCY5/AL	34
BP	GO:00071	heterophili	13/1638	46/18670	0.000112	0.001795	0.001377	CBLN1/ITG	13
BP	GO:00019	endothelia	33/1638	191/18670	0.000124	0.00198	0.001519	AGTR1/AP	33
BP	GO:00032	cardiac ch	25/1638	129/18670	0.000127	0.00201	0.001541	COL11A1/	25
BP	GO:00508	negative re	17/1638	72/18670	0.000127	0.00201	0.001541	BCHE/CBL	17
BP	GO:00026	regulation	64/1638	458/18670	0.000131	0.002067	0.001585	A2M/APO	64
BP	GO:00075	skeletal m	29/1638	160/18670	0.000133	0.00209	0.001603	CASQ1/CA	29
BP	GO:00703	ERK1 and	48/1638	317/18670	0.000134	0.002103	0.001613	ADCYAP1/	48
BP	GO:00703	regulation	46/1638	300/18670	0.000136	0.002125	0.00163	ADCYAP1/	46
BP	GO:00400	negative re	57/1638	396/18670	0.000136	0.002125	0.00163	AGTR2/AP	57
BP	GO:00324	regulation	44/1638	283/18670	0.000137	0.002125	0.00163	ACTN2/AN	44
BP	GO:00019	regulation	31/1638	176/18670	0.000137	0.002125	0.00163	AGTR1/AP	31
BP	GO:00026	regulation	6/1638	11/18670	0.000142	0.002192	0.001681	CCR7/FCG	6
BP	GO:00069	acute-pha	13/1638	47/18670	0.000143	0.002197	0.001685	AHSG/CN	13
BP	GO:00603	cartilage d	13/1638	47/18670	0.000143	0.002197	0.001685	ANXA6/CC	13
BP	GO:00486	regulation	30/1638	169/18670	0.000151	0.002316	0.001776	APOD/CN	30
BP	GO:00618	antimicrob	17/1638	73/18670	0.000152	0.002324	0.001783	DEFB1/DE	17
BP	GO:00073	gastrulatio	32/1638	185/18670	0.000153	0.002329	0.001786	APLNR/AP	32
BP	GO:00458	positive re	27/1638	146/18670	0.000158	0.0024	0.001841	AGTR1/AB	27
BP	GO:00100	glial cell di	36/1638	218/18670	0.000161	0.002438	0.00187	DLX1/DLX	36
BP	GO:00508	regulation	36/1638	218/18670	0.000161	0.002438	0.00187	ADGRB1/A	36
BP	GO:00301	negative re	35/1638	210/18670	0.000164	0.002476	0.001899	CAV1/NKX	35
BP	GO:00605	morphoge	9/1638	25/18670	0.000168	0.002525	0.001936	AR/FGF10/	9
BP	GO:00217	limbic syst	22/1638	109/18670	0.000171	0.002552	0.001957	ALDH1A3/	22
BP	GO:19000	negative re	14/1638	54/18670	0.000171	0.002552	0.001957	APOH/SER	14
BP	GO:00067	isoprenoid	26/1638	139/18670	0.000171	0.002552	0.001957	ADH1B/AD	26
BP	GO:00517	multi-orga	16/1638	67/18670	0.000172	0.002561	0.001965	AVP/CX3C	16
BP	GO:00508	regulation	37/1638	227/18670	0.000173	0.002562	0.001965	ADGRB1/A	37
BP	GO:00034	growth pla	11/1638	36/18670	0.000175	0.002584	0.001982	ANXA6/CC	11
BP	GO:00018	mesoneph	21/1638	102/18670	0.000178	0.002624	0.002013	AGTR2/FG	21
BP	GO:00020	chondrocy	13/1638	48/18670	0.00018	0.002643	0.002027	COL6A1/C	13
BP	GO:00308	prostate gl	13/1638	48/18670	0.00018	0.002643	0.002027	AR/ESR1/F	13
BP	GO:00076	memory	23/1638	117/18670	0.000186	0.002717	0.002084	CNR1/CX3	23
BP	GO:00486	smooth m	30/1638	171/18670	0.000187	0.002729	0.002093	APOD/CN	30
BP	GO:00486	animal org	15/1638	61/18670	0.000193	0.00279	0.00214	AR/NKX3-	15
BP	GO:00018	epithelial t	26/1638	140/18670	0.000193	0.00279	0.00214	COL1A1/E	26
BP	GO:00720	kidney epi	26/1638	140/18670	0.000193	0.00279	0.00214	AGTR2/EP	26
BP	GO:00452	cell-cell ju	28/1638	156/18670	0.000204	0.002934	0.002251	ANK2/CAV	28
BP	GO:00485	embryonic	44/1638	288/18670	0.000204	0.002934	0.002251	ALDH1A3/	44
BP	GO:00336	cell adhesi	16/1638	68/18670	0.000207	0.002968	0.002277	COL16A1/	16
BP	GO:00099	proximal/c	10/1638	31/18670	0.000209	0.002968	0.002277	DLX1/DLX	10
BP	GO:00514	positive re	10/1638	31/18670	0.000209	0.002968	0.002277	AGTR1/CA	10
BP	GO:00991	regulation	10/1638	31/18670	0.000209	0.002968	0.002277	CBLN1/NT	10

BP	GO:00989(	regulation	14/1638	55/18670	0.000211	0.002996	0.002298	ANK2/CAC		14
BP	GO:00604:	muscle tiss	18/1638	82/18670	0.000219	0.003094	0.002373	ACTC1/CC		18
BP	GO:00485	digestive t	13/1638	49/18670	0.000226	0.003187	0.002444	FGF10/FOX		13
BP	GO:00165	negative re	30/1638	173/18670	0.000231	0.003251	0.002493	APOH/AD(		30
BP	GO:00075	respiratory	15/1638	62/18670	0.000234	0.00328	0.002516	PHOX2A/A		15
BP	GO:00600	synapse m	9/1638	26/18670	0.000237	0.003316	0.002544	CX3CR1/N		9
BP	GO:00720(	nephron d	26/1638	142/18670	0.000244	0.003408	0.002614	ACTA2/AG		26
BP	GO:00070	cell-substr	20/1638	97/18670	0.000246	0.003428	0.002629	ACTN2/AP		20
BP	GO:00457	positive re	19/1638	90/18670	0.000253	0.003517	0.002698	GLI3/HGF/		19
BP	GO:00423	vasoconstr	17/1638	76/18670	0.000256	0.003537	0.002713	ACTA2/AG		17
BP	GO:00019	positive re	22/1638	112/18670	0.000257	0.003541	0.002716	AGTR1/AP		22
BP	GO:00604	heart grow	22/1638	112/18670	0.000257	0.003541	0.002716	AGTR2/NK		22
BP	GO:00703	positive re	35/1638	215/18670	0.000262	0.003594	0.002757	ADCYAP1/		35
BP	GO:00024(	dendritic c	6/1638	12/18670	0.000262	0.003594	0.002757	CCR7/FCG		6
BP	GO:00550	cardiac mu	21/1638	105/18670	0.000272	0.003712	0.002847	AGTR2/NK		21
BP	GO:00508(	negative re	33/1638	199/18670	0.000274	0.003732	0.002863	AXL/BPI/C		33
BP	GO:00016(	ameboidal	63/1638	461/18670	0.000275	0.003732	0.002863	AGTR2/AN		63
BP	GO:00101	body morp	13/1638	50/18670	0.000281	0.003812	0.002924	COL1A1/M		13
BP	GO:20001	negative re	30/1638	175/18670	0.000284	0.003839	0.002945	APOH/AD(		30
BP	GO:00301	regulation	52/1638	363/18670	0.000295	0.003961	0.003038	CAV1/COL		52
BP	GO:00104(	mesenchyr	12/1638	44/18670	0.000296	0.003961	0.003038	FGF7/FGFF		12
BP	GO:00617	leukocyte a	12/1638	44/18670	0.000296	0.003961	0.003038	ADD2/CX3		12
BP	GO:00519(	regulation	16/1638	70/18670	0.000296	0.003961	0.003038	ADCYAP1/		16
BP	GO:19035	positive re	56/1638	399/18670	0.0003	0.003992	0.003062	ADCYAP1/		56
BP	GO:00508	endocrine	18/1638	84/18670	0.000301	0.003992	0.003062	AGTR1/AG		18
BP	GO:00977	negative re	18/1638	84/18670	0.000301	0.003992	0.003062	ACTA2/AG		18
BP	GO:00508(	regulation	31/1638	184/18670	0.000311	0.004122	0.003162	CD19/CD2		31
BP	GO:00215	telencepha	39/1638	251/18670	0.000323	0.004269	0.003275	ALDH1A3/		39
BP	GO:00603	bone morp	22/1638	114/18670	0.000334	0.004402	0.003377	ANXA6/CC		22
BP	GO:00456(	positive re	15/1638	64/18670	0.000339	0.004462	0.003422	GLI3/HGF/		15
BP	GO:00065	thyroid ho	7/1638	17/18670	0.000349	0.004563	0.0035	CGA/DIO2		7
BP	GO:00971(	postsynapt	7/1638	17/18670	0.000349	0.004563	0.0035	CBLN1/NT		7
BP	GO:00605	skeletal mu	29/1638	169/18670	0.00035	0.004563	0.0035	CASQ1/CA		29
BP	GO:19044	positive re	16/1638	71/18670	0.000351	0.004576	0.00351	APLNR/AN		16
BP	GO:00359	steroid ho	8/1638	22/18670	0.000359	0.004669	0.003581	AGTR1/AG		8
BP	GO:00605(	epithelial t	47/1638	322/18670	0.000369	0.004777	0.003664	AGTR2/AR		47
BP	GO:00199	cGMP-me	10/1638	33/18670	0.00037	0.004777	0.003664	CD36/GUC		10
BP	GO:00990	postsynapt	10/1638	33/18670	0.00037	0.004777	0.003664	CBLN1/GA		10
BP	GO:00435	ear develo	35/1638	219/18670	0.000374	0.004815	0.003693	ALDH1A3/		35
BP	GO:20000	regulation	39/1638	253/18670	0.00038	0.004877	0.003741	AGTR2/AR		39
BP	GO:00319(	response t	28/1638	162/18670	0.000388	0.004971	0.003813	ADCYAP1/		28
BP	GO:00030	regulation	19/1638	93/18670	0.000394	0.005029	0.003858	ADRB3/AG		19
BP	GO:00031	outflow tra	17/1638	79/18670	0.000415	0.00528	0.00405	NKX2-5/EI		17
BP	GO:00192	transmissic	16/1638	72/18670	0.000415	0.00528	0.00405	AVP/DRD5		16
BP	GO:00487	tissue rem	30/1638	179/18670	0.000422	0.005355	0.004108	AGTR2/AX		30
BP	GO:00351	social beh	13/1638	52/18670	0.000426	0.005367	0.004117	AVP/CX3C		13
BP	GO:00517(	intraspecie	13/1638	52/18670	0.000426	0.005367	0.004117	AVP/CX3C		13
BP	GO:00721	mesenchyr	13/1638	52/18670	0.000426	0.005367	0.004117	ACTA2/AC		13
BP	GO:00901	tissue migr	51/1638	360/18670	0.000438	0.005498	0.004217	ACTA2/AC		51
BP	GO:00080	motor neu	9/1638	28/18670	0.000446	0.005572	0.004274	ERBB2/NTI		9
BP	GO:00301(	regulation	42/1638	281/18670	0.00045	0.005572	0.004274	AHSG/APC		42
BP	GO:00020	regulation	6/1638	13/18670	0.000451	0.005572	0.004274	ATP1A2/PI		6
BP	GO:00028(	regulation	6/1638	13/18670	0.000451	0.005572	0.004274	ADCYAP1/		6
BP	GO:00300(	cellular po	6/1638	13/18670	0.000451	0.005572	0.004274	ATP1A2/A		6
BP	GO:00380(	collagen-a	6/1638	13/18670	0.000451	0.005572	0.004274	COL1A1/C		6
BP	GO:00702(	protein he	6/1638	13/18670	0.000451	0.005572	0.004274	COL1A1/C		6
BP	GO:00604	mammary	12/1638	46/18670	0.000463	0.005679	0.004356	AR/CAV1/		12
BP	GO:00105	positive re	14/1638	59/18670	0.000463	0.005679	0.004356	ADCYAP1F		14
BP	GO:00311(	neuron prc	14/1638	59/18670	0.000463	0.005679	0.004356	APOA1/AF		14
BP	GO:00329	regulation	54/1638	388/18670	0.000468	0.005725	0.004391	ACTN2/AD		54
BP	GO:00905	sensory or	39/1638	256/18670	0.000483	0.005892	0.004519	ALDH1A3/		39
BP	GO:00485(	embryonic	10/1638	34/18670	0.000484	0.005893	0.00452	FGF10/FOX		10

BP	GO:00705(	calcium ioı	17/1638	80/18670	0.000485	0.005893	0.00452	ATP2B4/C/	17
BP	GO:00603!	endochonc	16/1638	73/18670	0.000489	0.005934	0.004552	ANXA6/CC	16
BP	GO:00323:	negative re	8/1638	23/18670	0.000509	0.006142	0.004711	NKX3-2/EI	8
BP	GO:00435;	regulation	8/1638	23/18670	0.000509	0.006142	0.004711	PHOX2A/A	8
BP	GO:00301!	negative re	13/1638	53/18670	0.00052	0.006239	0.004786	APOH/SER	13
BP	GO:00452:	sarcomere	13/1638	53/18670	0.00052	0.006239	0.004786	ACTN2/CA	13
BP	GO:00104(	regulation	29/1638	173/18670	0.000521	0.006239	0.004786	ACTN2/AD	29
BP	GO:00987:	import acr	21/1638	110/18670	0.000525	0.006273	0.004811	ATP1A2/A	21
BP	GO:00017(	neuron mi	27/1638	157/18670	0.00053	0.006327	0.004853	AXL/CCR4/	27
BP	GO:00181(	peptidyl-ty	51/1638	363/18670	0.000532	0.006336	0.004859	ALK/AXL/E	51
BP	GO:00310!	regenerati	32/1638	198/18670	0.000535	0.006356	0.004875	APOA1/AF	32
BP	GO:00486	muscle org	18/1638	88/18670	0.000546	0.006469	0.004962	ACTC1/CC	18
BP	GO:00217(	developme	42/1638	284/18670	0.000562	0.006644	0.005096	AXL/ADGR	42
BP	GO:00217(	hippocamp	17/1638	81/18670	0.000564	0.00666	0.005108	ALK/ATP2I	17
BP	GO:00305;	collagen ca	12/1638	47/18670	0.000572	0.006737	0.005167	COL15A1/	12
BP	GO:00996(	regulation	16/1638	74/18670	0.000574	0.006747	0.005175	ACTN2/CR	16
BP	GO:00510	positive re	58/1638	428/18670	0.000582	0.006821	0.005232	ADCYAP1/	58
BP	GO:00343:	adherens j	25/1638	142/18670	0.000587	0.006862	0.005263	ACTN2/AF	25
BP	GO:00313	positive re	53/1638	383/18670	0.000602	0.007025	0.005388	ADCYAP1/	53
BP	GO:00483:	mesoderm	10/1638	35/18670	0.000625	0.007258	0.005567	FGFR1/FO)	10
BP	GO:00988(	regulation	10/1638	35/18670	0.000625	0.007258	0.005567	AGTR1/AG	10
BP	GO:00072:	neuropept	20/1638	104/18670	0.000639	0.007395	0.005672	ADCYAP1/	20
BP	GO:00324:	positive re	20/1638	104/18670	0.000639	0.007395	0.005672	ACTN2/AN	20
BP	GO:00182:	peptidyl-ty	51/1638	366/18670	0.000644	0.007435	0.005703	ALK/AXL/E	51
BP	GO:00488:	inner ear c	31/1638	192/18670	0.000659	0.00758	0.005814	ALDH1A3/	31
BP	GO:00507(	negative re	43/1638	295/18670	0.00066	0.00758	0.005814	ADCYAP1/	43
BP	GO:00016!	ureteric bu	19/1638	97/18670	0.000683	0.007812	0.005992	AGTR2/FG	19
BP	GO:00322:	regulation	19/1638	97/18670	0.000683	0.007812	0.005992	APOA1/CC	19
BP	GO:00080:	neuron rec	12/1638	48/18670	0.000703	0.008006	0.006141	CNR1/EPH	12
BP	GO:19037;	positive re	12/1638	48/18670	0.000703	0.008006	0.006141	CD19/CCR	12
BP	GO:00034:	chondrocy	8/1638	24/18670	0.000705	0.008015	0.006147	COL6A1/C	8
BP	GO:00302(	glycosamir	27/1638	160/18670	0.000717	0.008135	0.00624	BGN/VCAI	27
BP	GO:19013;	regulation	18/1638	90/18670	0.000723	0.008165	0.006263	ACTN2/AN	18
BP	GO:19030:	negative re	18/1638	90/18670	0.000723	0.008165	0.006263	APOH/SER	18
BP	GO:00509:	induction c	6/1638	14/18670	0.000731	0.008215	0.006301	FGF10/VE(	6
BP	GO:00519:	regulation	6/1638	14/18670	0.000731	0.008215	0.006301	APOH/CPE	6
BP	GO:00075!	hemostasis	48/1638	341/18670	0.000732	0.008215	0.006301	A2M/APOI	48
BP	GO:00104;	regulation	11/1638	42/18670	0.000763	0.008545	0.006554	APOA1/CC	11
BP	GO:00519(	negative re	45/1638	315/18670	0.000773	0.008634	0.006623	ADCYAP1/	45
BP	GO:00721(	mesoneph	19/1638	98/18670	0.000779	0.008665	0.006646	AGTR2/FG	19
BP	GO:00721(	mesoneph	19/1638	98/18670	0.000779	0.008665	0.006646	AGTR2/FG	19
BP	GO:00508:	coagulatio	48/1638	342/18670	0.000781	0.008666	0.006647	A2M/APOI	48
BP	GO:00610	negative re	16/1638	76/18670	0.000783	0.008666	0.006647	APOH/SER	16
BP	GO:00482(	response t	9/1638	30/18670	0.000787	0.008666	0.006647	CRH/NTRK	9
BP	GO:00514!	positive re	9/1638	30/18670	0.000787	0.008666	0.006647	CCR7/DPY	9
BP	GO:19056(	regulation	9/1638	30/18670	0.000787	0.008666	0.006647	CBLN1/NT	9
BP	GO:00328:	glomerulus	14/1638	62/18670	0.00079	0.008675	0.006654	ACTA2/AG	14
BP	GO:00550(	cardiac mu	15/1638	69/18670	0.000795	0.008717	0.006686	ACTC1/CC	15
BP	GO:00309	regulation	10/1638	36/18670	0.000798	0.008729	0.006696	FGF10/ITG	10
BP	GO:00316	regulation	24/1638	137/18670	0.000804	0.008784	0.006737	AVP/CBLN	24
BP	GO:00011(	response t	48/1638	343/18670	0.000833	0.009055	0.006945	APOB/ATP	48
BP	GO:00022:	response t	48/1638	343/18670	0.000833	0.009055	0.006945	APOB/ATP	48
BP	GO:00900!	negative re	29/1638	178/18670	0.000835	0.00906	0.006949	CAV1/NKX	29
BP	GO:00015!	regulation	56/1638	416/18670	0.000842	0.009114	0.00699	AGTR1/AG	56
BP	GO:00060;	aminoglyc	28/1638	170/18670	0.000855	0.00924	0.007087	BGN/VCAI	28
BP	GO:00027!	regulation	65/1638	500/18670	0.000861	0.009285	0.007122	ADCY5/AD	65
BP	GO:00507(	regulation	62/1638	472/18670	0.000863	0.009285	0.007122	ADCY5/AG	62
BP	GO:00550(	monovaler	26/1638	154/18670	0.000885	0.009408	0.007216	AGTR1/AG	26
BP	GO:00600;	canonical \	47/1638	335/18670	0.000888	0.009408	0.007216	CAV1/COL	47
BP	GO:00199:	cAMP-me(	30/1638	187/18670	0.000888	0.009408	0.007216	ADCY2/AD	30
BP	GO:19013	negative re	30/1638	187/18670	0.000888	0.009408	0.007216	APOH/AD(	30
BP	GO:00513{	response t	25/1638	146/18670	0.000892	0.009408	0.007216	ADCYAP1/	25

BP	GO:00343	chylomicro	5/1638	10/18670	0.000894	0.009408	0.007216	APOA1/AF	5
BP	GO:00380	collagen-a	5/1638	10/18670	0.000894	0.009408	0.007216	COL1A1/C	5
BP	GO:00519	negative re	5/1638	10/18670	0.000894	0.009408	0.007216	APOH/CPE	5
BP	GO:00605	prostatic b	5/1638	10/18670	0.000894	0.009408	0.007216	AR/FGF10/	5
BP	GO:00970	dendritic c	5/1638	10/18670	0.000894	0.009408	0.007216	AXL/CCR7	5
BP	GO:20006	regulation	5/1638	10/18670	0.000894	0.009408	0.007216	AXL/CCR7	5
BP	GO:00509	neuromusc	20/1638	107/18670	0.00093	0.009766	0.007491	ADCY5/AL	20
BP	GO:00075	blood coag	47/1638	336/18670	0.000946	0.009923	0.007611	A2M/APOI	47
BP	GO:00082	glucocortic	8/1638	25/18670	0.000958	0.009975	0.007651	APOA1/SE	8
BP	GO:00219	central ner	8/1638	25/18670	0.000958	0.009975	0.007651	CDH11/GL	8
BP	GO:00316	plasminog	8/1638	25/18670	0.000958	0.009975	0.007651	APOH/CPE	8
BP	GO:00312	biomineral	27/1638	163/18670	0.000959	0.009975	0.007651	AHSG/COL	27
BP	GO:00466	female sex	21/1638	115/18670	0.000961	0.009975	0.007651	ADCYAP1/	21
BP	GO:19018	regulation	26/1638	155/18670	0.000976	0.010114	0.007757	NKX2-5/F	26
BP	GO:00160	cell growth	63/1638	484/18670	0.000993	0.010269	0.007876	AGTR1/AG	63
BP	GO:00421	B cell activ	44/1638	310/18670	0.001004	0.010357	0.007944	CXCR5/CD	44
BP	GO:00344	substrate a	19/1638	100/18670	0.001005	0.010357	0.007944	APOA1/AX	19
BP	GO:00083	associative	16/1638	78/18670	0.001052	0.010814	0.008295	ATP1A2/C	16
BP	GO:00332	response t	18/1638	93/18670	0.00108	0.011062	0.008485	BCHE/COL	18
BP	GO:00352	synaptic tr	18/1638	93/18670	0.00108	0.011062	0.008485	ADCYAP1/	18
BP	GO:00508	negative re	13/1638	57/18670	0.001088	0.011125	0.008533	APOH/SER	13
BP	GO:00025	regulation	7/1638	20/18670	0.001099	0.011143	0.008547	CCR7/FCG	7
BP	GO:00860	atrial cardi	7/1638	20/18670	0.001099	0.011143	0.008547	ANK2/CAC	7
BP	GO:00860	atrial cardi	7/1638	20/18670	0.001099	0.011143	0.008547	ANK2/CAC	7
BP	GO:00860	atrial cardi	7/1638	20/18670	0.001099	0.011143	0.008547	ANK2/CAC	7
BP	GO:00357	sodium ior	24/1638	140/18670	0.001101	0.011143	0.008547	ATP1A2/A	24
BP	GO:00424	odontoger	23/1638	132/18670	0.001103	0.011143	0.008547	COL1A1/C	23
BP	GO:00181	protein oxi	6/1638	15/18670	0.001128	0.011356	0.008711	APOA1/AF	6
BP	GO:00468	negative re	6/1638	15/18670	0.001128	0.011356	0.008711	CALCA/IL6	6
BP	GO:00440	regulation	11/1638	44/18670	0.001162	0.011616	0.00891	AGTR1/AG	11
BP	GO:00514	regulation	11/1638	44/18670	0.001162	0.011616	0.00891	CCR7/DPY	11
BP	GO:20004	regulation	11/1638	44/18670	0.001162	0.011616	0.00891	APOD/CD2	11
BP	GO:00486	reproducti	57/1638	431/18670	0.001163	0.011616	0.00891	ADCYAP1/	57
BP	GO:00485	response t	52/1638	385/18670	0.001173	0.011699	0.008974	ADCYAP1/	52
BP	GO:00608	regulation	41/1638	286/18670	0.001206	0.012005	0.009208	CAV1/COL	41
BP	GO:00216	nerve deve	16/1638	79/18670	0.001214	0.012053	0.009245	PHOX2A/C	16
BP	GO:00456	negative re	12/1638	51/18670	0.001253	0.012394	0.009506	GDF10/IGF	12
BP	GO:00482	positive re	12/1638	51/18670	0.001253	0.012394	0.009506	C3/SERPIN	12
BP	GO:00071	activation	10/1638	38/18670	0.001262	0.012467	0.009562	ADCY2/AD	10
BP	GO:00025	monocyte	14/1638	65/18670	0.001292	0.012736	0.009769	CALCA/IL6	14
BP	GO:00108	regulation	9/1638	32/18670	0.001315	0.012922	0.009911	ANK2/ATP	9
BP	GO:00026	positive re	17/1638	87/18670	0.001316	0.012922	0.009911	CCR7/F7/V	17
BP	GO:00508	positive re	24/1638	142/18670	0.001347	0.013208	0.010131	IGHD/IGH	24
BP	GO:00424	ear morph	21/1638	118/18670	0.00135	0.013211	0.010133	ALDH1A3/	21
BP	GO:00487	gland deve	57/1638	434/18670	0.001369	0.013342	0.010234	ADCYAP1/	57
BP	GO:00614	reproducti	57/1638	434/18670	0.001369	0.013342	0.010234	ADCYAP1/	57
BP	GO:00705	calcium ioi	44/1638	315/18670	0.00139	0.013527	0.010376	APLNR/AN	44
BP	GO:00026	negative re	60/1638	463/18670	0.001438	0.013971	0.010716	A2M/ADC	60
BP	GO:00189	phenol-co	19/1638	103/18670	0.00145	0.01406	0.010784	AGTR2/CG	19
BP	GO:19031	regulation	25/1638	151/18670	0.001462	0.014144	0.010848	APLNR/AN	25
BP	GO:00507	regulation	32/1638	210/18670	0.001474	0.014234	0.010918	AGTR2/AP	32
BP	GO:00071	negative re	41/1638	289/18670	0.001476	0.014234	0.010918	APOA1/AF	41
BP	GO:00023	immunogl	30/1638	193/18670	0.001486	0.014285	0.010957	CD22/FCG	30
BP	GO:00726	lymphocyt	20/1638	111/18670	0.001487	0.014285	0.010957	APOD/CCI	20
BP	GO:00101	embryonic	5/1638	11/18670	0.00152	0.014452	0.011085	MAB21L2/	5
BP	GO:00613	positive re	5/1638	11/18670	0.00152	0.014452	0.011085	APOA4/AF	5
BP	GO:00024	acute infla	7/1638	21/18670	0.001525	0.014452	0.011085	ADCYAP1/	7
BP	GO:00358	regulation	7/1638	21/18670	0.001525	0.014452	0.011085	AGTR1/AG	7
BP	GO:00424	thyroid ho	7/1638	21/18670	0.001525	0.014452	0.011085	CGA/DIO2	7
BP	GO:19030	positive re	7/1638	21/18670	0.001525	0.014452	0.011085	CPB2/IL6/I	7
BP	GO:00016	branching	13/1638	59/18670	0.001526	0.014452	0.011085	AGTR2/FG	13
BP	GO:00218	forebrain c	13/1638	59/18670	0.001526	0.014452	0.011085	AXL/GLI3/I	13

BP	GO:00603(	regulation	10/1638	39/18670	0.001566	0.0148	0.011352	ANK2/CAC	10
BP	GO:00341(	homotypic	16/1638	81/18670	0.0016	0.015099	0.011581	COMP/CSI	16
BP	GO:00162(	regulation	25/1638	152/18670	0.001607	0.015133	0.011607	NKX2-5/F(	25
BP	GO:00149(	myotube c	20/1638	112/18670	0.001664	0.015526	0.011909	ADGRB1/A	20
BP	GO:00324:	positive re	20/1638	112/18670	0.001664	0.015526	0.011909	ACTN2/AN	20
BP	GO:00986!	regulation	20/1638	112/18670	0.001664	0.015526	0.011909	CACNB2/C	20
BP	GO:00100!	specificatic	9/1638	33/18670	0.001669	0.015526	0.011909	AR/FGF10/	9
BP	GO:00108:	regulation	9/1638	33/18670	0.001669	0.015526	0.011909	KLF9/FGF7	9
BP	GO:00606(	mammary	9/1638	33/18670	0.001669	0.015526	0.011909	AR/CSF1R/	9
BP	GO:00435	endothelia	39/1638	273/18670	0.00167	0.015526	0.011909	AGTR2/AP	39
BP	GO:00440(	regulation	6/1638	16/18670	0.001672	0.015526	0.011909	ATP1A2/PI	6
BP	GO:00519!	negative re	8/1638	27/18670	0.001678	0.015556	0.011932	AGTR2/CN	8
BP	GO:00600:	roof of mc	17/1638	89/18670	0.001706	0.015782	0.012105	FOXF2/FO:	17
BP	GO:00432	apoptotic (	11/1638	46/18670	0.001717	0.015863	0.012168	AXL/ADGR	11
BP	GO:00486(	negative re	14/1638	67/18670	0.001757	0.016182	0.012412	APOD/CNI	14
BP	GO:00301(	platelet ac	25/1638	153/18670	0.001764	0.016182	0.012412	AXL/COL1.	25
BP	GO:00458(	positive re	25/1638	153/18670	0.001764	0.016182	0.012412	AHSG/APC	25
BP	GO:00510:	actin filam	25/1638	153/18670	0.001764	0.016182	0.012412	ADD2/APC	25
BP	GO:00069	regulation	18/1638	97/18670	0.001782	0.016257	0.012469	ANK2/ATP	18
BP	GO:00487(	oligodendı	18/1638	97/18670	0.001782	0.016257	0.012469	DLX1/DLX:	18
BP	GO:00515!	response t	18/1638	97/18670	0.001782	0.016257	0.012469	ALDH3A1/	18
BP	GO:00725!	reactive ox	40/1638	284/18670	0.001916	0.017451	0.013385	AGTR1/AG	40
BP	GO:00019	postsynapt	10/1638	40/18670	0.001926	0.017515	0.013434	CEL/GLRB/	10
BP	GO:00324!	response t	45/1638	330/18670	0.002006	0.018211	0.013968	APOB/ATP	45
BP	GO:00509:	positive ch	14/1638	68/18670	0.002038	0.018464	0.014162	CCR4/DEFI	14
BP	GO:00215:	spinal corc	19/1638	106/18670	0.002052	0.018563	0.014238	PHOX2A/E	19
BP	GO:00609{	endocrine	11/1638	47/18670	0.002067	0.018593	0.014261	AGTR1/AG	11
BP	GO:00613{	trabecula r	11/1638	47/18670	0.002067	0.018593	0.014261	COL1A1/N	11
BP	GO:00336:	integrin ac	7/1638	22/18670	0.002069	0.018593	0.014261	COL16A1/	7
BP	GO:00026{	regulation	20/1638	114/18670	0.002073	0.018593	0.014261	CCR7/F7/V	20
BP	GO:19038	regulation	21/1638	122/18670	0.002073	0.018593	0.014261	CAV1/CIDI	21
BP	GO:00986!	inorganic (	16/1638	83/18670	0.002086	0.018643	0.0143	ATP1A2/A	16
BP	GO:00995{	inorganic i	16/1638	83/18670	0.002086	0.018643	0.0143	ATP1A2/A	16
BP	GO:00322:	positive re	13/1638	61/18670	0.002101	0.018749	0.014381	APOA1/CC	13
BP	GO:00215	pallium de	27/1638	172/18670	0.002158	0.019222	0.014744	ALK/ATP2I	27
BP	GO:00343(	protein-lip	8/1638	28/18670	0.002171	0.019245	0.014761	AGTR1/AP	8
BP	GO:00343(	plasma lip	8/1638	28/18670	0.002171	0.019245	0.014761	AGTR1/AP	8
BP	GO:00456{	negative re	8/1638	28/18670	0.002171	0.019245	0.014761	DLX1/DLX:	8
BP	GO:00485:	rhythmic p	41/1638	295/18670	0.002178	0.019274	0.014783	AFP/AXL/K	41
BP	GO:00518!	membrane	17/1638	91/18670	0.002189	0.019334	0.014829	ANK2/ATP	17
BP	GO:00488	artery mor	15/1638	76/18670	0.002228	0.019654	0.015075	APOB/COL	15
BP	GO:00486:	regulation	25/1638	156/18670	0.002318	0.020411	0.015655	NKX2-5/F(	25
BP	GO:00075:	myoblast f	10/1638	41/18670	0.00235	0.020544	0.015757	ADGRB1/A	10
BP	GO:00351:	forelimb m	10/1638	41/18670	0.00235	0.020544	0.015757	CACNA1C/	10
BP	GO:00440!	membrane	10/1638	41/18670	0.00235	0.020544	0.015757	CAV1/CEL/	10
BP	GO:19030!	regulation	10/1638	41/18670	0.00235	0.020544	0.015757	AEBP1/CPI	10
BP	GO:00604:	regulation	16/1638	84/18670	0.002371	0.020544	0.015757	NKX2-5/F(	16
BP	GO:00344:	steroid est	6/1638	17/18670	0.002394	0.020544	0.015757	AGTR1/AP	6
BP	GO:00344:	sterol este	6/1638	17/18670	0.002394	0.020544	0.015757	AGTR1/AP	6
BP	GO:00344:	cholestero	6/1638	17/18670	0.002394	0.020544	0.015757	AGTR1/AP	6
BP	GO:00359:	corticoster	6/1638	17/18670	0.002394	0.020544	0.015757	AGTR1/AG	6
BP	GO:00460(	cGMP met	6/1638	17/18670	0.002394	0.020544	0.015757	GUCY1A1/	6
BP	GO:00600{	inhibitory (	6/1638	17/18670	0.002394	0.020544	0.015757	NTSR1/RIN	6
BP	GO:00608!	regulation	6/1638	17/18670	0.002394	0.020544	0.015757	DLX1/DLX:	6
BP	GO:00302(	dermatan :	5/1638	12/18670	0.002419	0.020544	0.015757	BGN/VCAI	5
BP	GO:00337(	phospholip	5/1638	12/18670	0.002419	0.020544	0.015757	APOA1/AF	5
BP	GO:00343	very-low-(	5/1638	12/18670	0.002419	0.020544	0.015757	APOA1/AF	5
BP	GO:00423	keratan su	5/1638	12/18670	0.002419	0.020544	0.015757	LUM/OMD	5
BP	GO:00426!	regulation	5/1638	12/18670	0.002419	0.020544	0.015757	AR/FGF2/F	5
BP	GO:00514{	negative re	5/1638	12/18670	0.002419	0.020544	0.015757	ATP1A2/K(	5
BP	GO:00970!	presynapti	5/1638	12/18670	0.002419	0.020544	0.015757	CEL/CNTN	5
BP	GO:00991!	regulation	5/1638	12/18670	0.002419	0.020544	0.015757	CBLN1/GA	5

BP	GO:00995	trans-syna	5/1638	12/18670	0.002419	0.020544	0.015757	CNR1/F2R	5
BP	GO:01500	regulation	5/1638	12/18670	0.002419	0.020544	0.015757	CBLN1/GA	5
BP	GO:00504	regulation	13/1638	62/18670	0.002449	0.020764	0.015927	AGTR2/CN	13
BP	GO:00550	ventricular	11/1638	48/18670	0.002473	0.020936	0.016058	COL11A1/	11
BP	GO:00550	regulation	18/1638	100/18670	0.002531	0.02134	0.016368	NKX2-5/F	18
BP	GO:00432	negative re	25/1638	157/18670	0.002533	0.02134	0.016368	ACTN2/AD	25
BP	GO:00615	actin filam	25/1638	157/18670	0.002533	0.02134	0.016368	ADD2/APC	25
BP	GO:00486	response t	15/1638	77/18670	0.002548	0.02143	0.016437	APOA1/AP	15
BP	GO:00032	cardiac atr	9/1638	35/18670	0.002604	0.021766	0.016695	ANK2/NKX	9
BP	GO:00194	triglyceride	9/1638	35/18670	0.002604	0.021766	0.016695	APOA1/AP	9
BP	GO:00219	central ner	9/1638	35/18670	0.002604	0.021766	0.016695	CDH11/GL	9
BP	GO:00519	positive re	9/1638	35/18670	0.002604	0.021766	0.016695	AVP/KCNE	9
BP	GO:00485	embryonic	55/1638	428/18670	0.002663	0.022223	0.017046	ALDH1A3/	55
BP	GO:00488	multicellula	61/1638	485/18670	0.002677	0.022306	0.017109	ADCY2/AD	61
BP	GO:00105	regulation	14/1638	70/18670	0.00271	0.022539	0.017288	ADCYAP1F	14
BP	GO:00507	positive re	29/1638	192/18670	0.002733	0.022696	0.017408	CD36/CNT	29
BP	GO:00074	axonal fasc	7/1638	23/18670	0.002752	0.022713	0.017421	CNR1/EPH	7
BP	GO:00358	renal sodiu	7/1638	23/18670	0.002752	0.022713	0.017421	AGTR1/AG	7
BP	GO:00713	cellular res	7/1638	23/18670	0.002752	0.022713	0.017421	TNC/PENK	7
BP	GO:01060	neuron pro	7/1638	23/18670	0.002752	0.022713	0.017421	CNR1/EPH	7
BP	GO:00069	substrate-	8/1638	29/18670	0.002771	0.022727	0.017432	FN1/SNAI2	8
BP	GO:00106	cell comm	8/1638	29/18670	0.002771	0.022727	0.017432	ATP1A2/A	8
BP	GO:00343	protein-co	8/1638	29/18670	0.002771	0.022727	0.017432	AGTR1/AP	8
BP	GO:00026	negative re	27/1638	175/18670	0.002772	0.022727	0.017432	AXL/BPI/C	27
BP	GO:00901	epithelium	47/1638	354/18670	0.002775	0.022727	0.017432	AGTR2/AP	47
BP	GO:00303	embryonic	21/1638	125/18670	0.00281	0.022937	0.017593	CACNA1C	21
BP	GO:00351	embryonic	21/1638	125/18670	0.00281	0.022937	0.017593	CACNA1C	21
BP	GO:00465	developme	18/1638	101/18670	0.002833	0.023094	0.017714	ADCYAP1/	18
BP	GO:20004	regulation	13/1638	63/18670	0.002843	0.023131	0.017742	APOD/CD2	13
BP	GO:00219	pituitary gl	10/1638	42/18670	0.002846	0.023131	0.017742	ADCYAP1/	10
BP	GO:00720	nephron e	19/1638	109/18670	0.002852	0.023137	0.017747	AGTR2/FG	19
BP	GO:00350	cardiocyte	26/1638	167/18670	0.002894	0.023449	0.017986	ACTC1/AC	26
BP	GO:00550	regulation	15/1638	78/18670	0.002904	0.023453	0.017989	NKX2-5/F	15
BP	GO:00720	nephron m	15/1638	78/18670	0.002904	0.023453	0.017989	AGTR2/FG	15
BP	GO:00220	telencepha	12/1638	56/18670	0.002928	0.023614	0.018113	GLI3/POU	12
BP	GO:00030	regulation	11/1638	49/18670	0.002941	0.023642	0.018134	ADRB3/AG	11
BP	GO:00107	regulation	11/1638	49/18670	0.002941	0.023642	0.018134	CCN2/F2/F	11
BP	GO:00518	positive re	27/1638	176/18670	0.003008	0.024146	0.018521	AXL/CD19	27
BP	GO:00466	response t	22/1638	134/18670	0.003034	0.024316	0.018651	ALDH3A1/	22
BP	GO:00321	negative re	48/1638	365/18670	0.00305	0.02441	0.018723	ADCYAP1/	48
BP	GO:00510	negative re	34/1638	238/18670	0.003188	0.025474	0.019539	AGTR2/AP	34
BP	GO:00148	release of	9/1638	36/18670	0.003206	0.025482	0.019545	ANK2/ATP	9
BP	GO:00717	membrane	9/1638	36/18670	0.003206	0.025482	0.019545	CAV1/CEL	9
BP	GO:20003	regulation	9/1638	36/18670	0.003206	0.025482	0.019545	ACTN2/CR	9
BP	GO:00508	positive re	51/1638	394/18670	0.003208	0.025482	0.019545	AXL/CAV1	51
BP	GO:00504	catecholan	13/1638	64/18670	0.003287	0.026065	0.019993	AGTR2/CN	13
BP	GO:00067	glucocortic	6/1638	18/18670	0.003327	0.026307	0.020178	CRH/CYP1	6
BP	GO:00485	embryonic	6/1638	18/18670	0.003327	0.026307	0.020178	FGF10/FOX	6
BP	GO:00108	positive re	10/1638	43/18670	0.003423	0.026821	0.020572	ADCYAP1F	10
BP	GO:00312	regulation	10/1638	43/18670	0.003423	0.026821	0.020572	CACNA1C	10
BP	GO:00464	neutral lipi	10/1638	43/18670	0.003423	0.026821	0.020572	APOA1/AP	10
BP	GO:00464	acylglycero	10/1638	43/18670	0.003423	0.026821	0.020572	APOA1/AP	10
BP	GO:00702	sarcoplasm	10/1638	43/18670	0.003423	0.026821	0.020572	ANK2/ATP	10
BP	GO:19905	potassium	10/1638	43/18670	0.003423	0.026821	0.020572	ATP1A2/A	10
BP	GO:00604	lung morp	11/1638	50/18670	0.003477	0.027207	0.020868	FGF7/FGF1	11
BP	GO:00032	cardiac atr	8/1638	30/18670	0.003492	0.027286	0.020929	NKX2-5/C	8
BP	GO:00482	regulation	18/1638	103/18670	0.003528	0.027522	0.02111	APOC3/C3	18
BP	GO:00305	adult beha	23/1638	144/18670	0.003538	0.027563	0.021141	ALK/ATP1	23
BP	GO:00028	regulation	7/1638	24/18670	0.003595	0.027758	0.021291	ADCYAP1/	7
BP	GO:00108	regulation	7/1638	24/18670	0.003595	0.027758	0.021291	ANK2/ATP	7
BP	GO:00215	spinal corc	7/1638	24/18670	0.003595	0.027758	0.021291	EVX1/GLI2	7
BP	GO:00215	subpallium	7/1638	24/18670	0.003595	0.027758	0.021291	ALDH1A3/	7

BP	GO:00440(	regulation	7/1638	24/18670	0.003595	0.027758	0.021291	AGTR1/AG	7
BP	GO:00603	trabecula f	7/1638	24/18670	0.003595	0.027758	0.021291	COL1A1/N	7
BP	GO:00506(	cytokine se	34/1638	240/18670	0.003649	0.02785	0.021361	AGTR2/AP	34
BP	GO:00104!	negative re	5/1638	13/18670	0.003649	0.02785	0.021361	SFRP2/SO!	5
BP	GO:00108	positive re	5/1638	13/18670	0.003649	0.02785	0.021361	FGF7/FGF1	5
BP	GO:00108	regulation	5/1638	13/18670	0.003649	0.02785	0.021361	AGTR1/AP	5
BP	GO:00302(	dermatan :	5/1638	13/18670	0.003649	0.02785	0.021361	BGN/VCAI	5
BP	GO:00509(	detection (	5/1638	13/18670	0.003649	0.02785	0.021361	HTR2A/KC	5
BP	GO:00510(	negative re	5/1638	13/18670	0.003649	0.02785	0.021361	ATP2B4/C/	5
BP	GO:00606(	lateral spro	5/1638	13/18670	0.003649	0.02785	0.021361	AR/FGF10/	5
BP	GO:00486(	neuron fat	13/1638	65/18670	0.003785	0.028766	0.022064	DLX1/DLX:	13
BP	GO:00606	ureteric bu	13/1638	65/18670	0.003785	0.028766	0.022064	AGTR2/FG	13
BP	GO:19010	regulation	13/1638	65/18670	0.003785	0.028766	0.022064	ACTN2/AN	13
BP	GO:00487	appendage	27/1638	179/18670	0.003819	0.028937	0.022195	CACNA1C/	27
BP	GO:00601	limb devel	27/1638	179/18670	0.003819	0.028937	0.022195	CACNA1C/	27
BP	GO:00170	regulation	20/1638	120/18670	0.003837	0.028999	0.022243	CAV1/CIDI	20
BP	GO:00024	productior	39/1638	286/18670	0.003838	0.028999	0.022243	APOA1/AF	39
BP	GO:00715	dopaminei	9/1638	37/18670	0.003913	0.029405	0.022555	PHOX2A/V	9
BP	GO:19035	release of :	9/1638	37/18670	0.003913	0.029405	0.022555	ANK2/ATP	9
BP	GO:20004(	positive re	9/1638	37/18670	0.003913	0.029405	0.022555	S100A7/C(	9
BP	GO:00085	female gor	17/1638	96/18670	0.003914	0.029405	0.022555	ADCYAP1/	17
BP	GO:00106	epithelial c	46/1638	351/18670	0.003921	0.029415	0.022562	AGTR2/AP	46
BP	GO:00192	regulation	12/1638	58/18670	0.003974	0.029727	0.022801	AGTR1/AT	12
BP	GO:00359(	aorta deve	12/1638	58/18670	0.003974	0.029727	0.022801	COL3A1/LI	12
BP	GO:00311(	animal org	14/1638	73/18670	0.00405	0.030209	0.023171	APOA1/AF	14
BP	GO:00335!	multicellul	14/1638	73/18670	0.00405	0.030209	0.023171	ADCYAP1/	14
BP	GO:00072	dopamine	10/1638	44/18670	0.004087	0.030286	0.02323	ADCY5/AL	10
BP	GO:00380	vascular er	10/1638	44/18670	0.004087	0.030286	0.02323	DCN/VEGI	10
BP	GO:00716	regulation	10/1638	44/18670	0.004087	0.030286	0.02323	CCR7/RAR	10
BP	GO:00311(	axon reger	11/1638	51/18670	0.004089	0.030286	0.02323	APOA1/AF	11
BP	GO:00451(	intermedia	11/1638	51/18670	0.004089	0.030286	0.02323	DES/GFAP.	11
BP	GO:19053	regulation	27/1638	180/18670	0.004127	0.030526	0.023414	AGTR2/AR	27
BP	GO:00991	postsynap:	25/1638	163/18670	0.004209	0.031068	0.02383	CBLN1/CEI	25
BP	GO:00329!	regulation	45/1638	343/18670	0.004212	0.031068	0.02383	ACTN2/AD	45
BP	GO:00070	cell-substr	15/1638	81/18670	0.004226	0.031082	0.023841	ACTN2/AP	15
BP	GO:00480	focal adhe	15/1638	81/18670	0.004226	0.031082	0.023841	ACTN2/AP	15
BP	GO:00550	cardiac mu	16/1638	89/18670	0.004327	0.031782	0.024377	ACTC1/AC	16
BP	GO:00218	forebrain (	13/1638	66/18670	0.004343	0.031782	0.024377	CSF1R/DL	13
BP	GO:00721	mesoneph	13/1638	66/18670	0.004343	0.031782	0.024377	AGTR2/FG	13
BP	GO:00346!	response t	8/1638	31/18670	0.004351	0.031782	0.024377	APOB/CCI	8
BP	GO:00971(	postsynap	8/1638	31/18670	0.004351	0.031782	0.024377	CBLN1/NT	8
BP	GO:00075	sex differe	37/1638	270/18670	0.004398	0.032079	0.024605	ADCYAP1/	37
BP	GO:00322	myelin ass	6/1638	19/18670	0.004506	0.032733	0.025107	ANK2/PMI	6
BP	GO:00609	cell migrat	6/1638	19/18670	0.004506	0.032733	0.025107	PDGFRB/S	6
BP	GO:20008	regulation	6/1638	19/18670	0.004506	0.032733	0.025107	AGTR1/AG	6
BP	GO:00705	platelet ag	12/1638	59/18670	0.004599	0.033329	0.025564	COMP/CSI	12
BP	GO:00720	nephron tu	14/1638	74/18670	0.004601	0.033329	0.025564	AGTR2/FG	14
BP	GO:00071	transformi	29/1638	199/18670	0.004619	0.033379	0.025602	CAV1/CIDI	29
BP	GO:19048(	excitatory :	7/1638	25/18670	0.00462	0.033379	0.025602	CBLN1/NT	7
BP	GO:00860(	ventricular	9/1638	38/18670	0.004735	0.034117	0.026169	ANK2/CAC	9
BP	GO:19027	apoptotic		9/1638	38/18670	0.004735	0.034117	0.026169	CRYAB/NK	9
BP	GO:00071	adenylate	22/1638	139/18670	0.004778	0.034379	0.02637	ADCY2/AD	22
BP	GO:00468	bone remo	16/1638	90/18670	0.004844	0.034608	0.026545	CALCA/CS	16
BP	GO:00716	mononucle	16/1638	90/18670	0.004844	0.034608	0.026545	CALCA/IL6	16
BP	GO:00484	autonomic	10/1638	45/18670	0.00485	0.034608	0.026545	PHOX2A/E	10
BP	GO:00519	synaptic tr	10/1638	45/18670	0.00485	0.034608	0.026545	CNR1/CNI	10
BP	GO:19002	regulation	10/1638	45/18670	0.00485	0.034608	0.026545	ADCYAP1I	10
BP	GO:00466	response t	43/1638	327/18670	0.00486	0.034608	0.026545	ACTC1/AD	43
BP	GO:00300	contractile	17/1638	98/18670	0.004862	0.034608	0.026545	APOA1/C(	17
BP	GO:00431	stress fiber	17/1638	98/18670	0.004862	0.034608	0.026545	APOA1/C(	17
BP	GO:00109	positive re	38/1638	281/18670	0.00492	0.034967	0.02682	ADCYAP1/	38
BP	GO:00351(	appendage	23/1638	148/18670	0.005	0.035395	0.027149	CACNA1C/	23

BP	GO:00351(	limb morp	23/1638	148/18670	0.005	0.035395	0.027149	CACNA1C/	23
BP	GO:00515(	response t	23/1638	148/18670	0.005	0.035395	0.027149	APOBEC1/	23
BP	GO:00712(	cellular res	30/1638	209/18670	0.005011	0.035424	0.027171	APOB/AKR	30
BP	GO:00068(	sodium ior	31/1638	218/18670	0.005043	0.035604	0.027309	ATP1A2/A	31
BP	GO:00032(	ventricular	14/1638	75/18670	0.00521	0.036689	0.028141	NKX2-5/C	14
BP	GO:00215(	diencepha	14/1638	75/18670	0.00521	0.036689	0.028141	ADCYAP1/	14
BP	GO:00102(	establishm	5/1638	14/18670	0.00527	0.036813	0.028236	ATP1A2/A	5
BP	GO:00343(	cell junctio	5/1638	14/18670	0.00527	0.036813	0.028236	CSF1R/F2F	5
BP	GO:00353(	regulation	5/1638	14/18670	0.00527	0.036813	0.028236	FABP5/LEF	5
BP	GO:00482(	behavioral	5/1638	14/18670	0.00527	0.036813	0.028236	NTRK1/TH	5
BP	GO:00860(	bundle of	5/1638	14/18670	0.00527	0.036813	0.028236	CACNA2D	5
BP	GO:00971(	GABAergic	5/1638	14/18670	0.00527	0.036813	0.028236	DLX1/DLX	5
BP	GO:00322(	regulation	8/1638	32/18670	0.005363	0.037265	0.028583	CNR1/CNF	8
BP	GO:00459(	positive re	8/1638	32/18670	0.005363	0.037265	0.028583	AVP/AVPR	8
BP	GO:00603(	face morpl	8/1638	32/18670	0.005363	0.037265	0.028583	COL1A1/M	8
BP	GO:00650(	protein-lip	8/1638	32/18670	0.005363	0.037265	0.028583	APOA1/AF	8
BP	GO:00099(	anterior/p	31/1638	219/18670	0.005395	0.037435	0.028714	FOXF1/FO	31
BP	GO:00017(	cell fate sp	17/1638	99/18670	0.005403	0.037443	0.028719	AR/EVX1/F	17
BP	GO:00140(	response t	23/1638	149/18670	0.005435	0.037615	0.028852	ALDH3A1/	23
BP	GO:00094(	response t	39/1638	292/18670	0.005463	0.037758	0.028961	AGTR2/AL	39
BP	GO:00329(	collagen b	11/1638	53/18670	0.005568	0.038385	0.029442	COL1A1/C	11
BP	GO:00456(	regulation	11/1638	53/18670	0.005568	0.038385	0.029442	MYF6/RIP(	11
BP	GO:00480(	inositol lipi	27/1638	184/18670	0.005577	0.038397	0.029451	CSF1R/DC	27
BP	GO:00070(	cell-cell ju	20/1638	124/18670	0.0056	0.038456	0.029496	ANK2/CAV	20
BP	GO:00140(	regulation	20/1638	124/18670	0.0056	0.038456	0.029496	ADCYAP1/	20
BP	GO:00108(	negative re	13/1638	68/18670	0.005658	0.038755	0.029726	APOD/CO	13
BP	GO:00359(	cellular res	13/1638	68/18670	0.005658	0.038755	0.029726	DCN/VEGF	13
BP	GO:00300(	muscle fila	9/1638	39/18670	0.005686	0.038845	0.029795	ACTC1/AC	9
BP	GO:00332(	actin-myo:	9/1638	39/18670	0.005686	0.038845	0.029795	ACTC1/AC	9
BP	GO:00507(	regulation	35/1638	256/18670	0.005696	0.038865	0.02981	CAV1/CD3	35
BP	GO:00509(	negative c	10/1638	46/18670	0.005721	0.03893	0.02986	APOA1/EP	10
BP	GO:00513(	regulation	10/1638	46/18670	0.005721	0.03893	0.02986	CACNA1C/	10
BP	GO:19035(	negative re	30/1638	211/18670	0.005748	0.039065	0.029963	AGTR2/AP	30
BP	GO:00105(	regulation	32/1638	229/18670	0.005769	0.039127	0.030011	AGTR2/AP	32
BP	GO:00024(	immune re	58/1638	473/18670	0.005772	0.039127	0.030011	BLK/CD19/	58
BP	GO:19010(	positive re	7/1638	26/18670	0.005851	0.039615	0.030385	ACTN2/AN	7
BP	GO:00305(	regulation	14/1638	76/18670	0.005884	0.039733	0.030476	AHSG/CO	14
BP	GO:00720(	nephron e	14/1638	76/18670	0.005884	0.039733	0.030476	AGTR2/FG	14
BP	GO:00995(	chemical s	18/1638	108/18670	0.005898	0.039777	0.03051	CBLN1/CH	18
BP	GO:00076(	copulation	6/1638	20/18670	0.005966	0.040082	0.030743	AVP/CNR1	6
BP	GO:00341(	negative re	6/1638	20/18670	0.005966	0.040082	0.030743	CALCA/IL6	6
BP	GO:00487(	smooth mu	6/1638	20/18670	0.005966	0.040082	0.030743	COL3A1/M	6
BP	GO:00514(	regulation	15/1638	84/18670	0.006001	0.040264	0.030883	APOA1/CC	15
BP	GO:00717(	tumor nec	25/1638	168/18670	0.006237	0.041799	0.032061	AXL/BPI/C	25
BP	GO:00511(	regulation	19/1638	117/18670	0.006331	0.042371	0.0325	ADGRB1/N	19
BP	GO:00301(	proteoglyc	13/1638	69/18670	0.006425	0.042899	0.032905	BGN/VCAN	13
BP	GO:00323(	response t	21/1638	134/18670	0.006446	0.042899	0.032905	ADCYAP1F	21
BP	GO:00509(	regulation	11/1638	54/18670	0.00645	0.042899	0.032905	ABCD2/AP	11
BP	GO:00610(	cell differe	11/1638	54/18670	0.00645	0.042899	0.032905	ACTA2/FO	11
BP	GO:00702(	protein trir	11/1638	54/18670	0.00645	0.042899	0.032905	COL1A1/C	11
BP	GO:00164(	peptide hc	8/1638	33/18670	0.006545	0.043366	0.033262	CGA/CMA	8
BP	GO:00351(	embryonic	8/1638	33/18670	0.006545	0.043366	0.033262	CACNA1C/	8
BP	GO:00519(	regulation	8/1638	33/18670	0.006545	0.043366	0.033262	ATP1A2/A	8
BP	GO:00447(	multi-mult	31/1638	222/18670	0.006575	0.043495	0.033361	ADCYAP1/	31
BP	GO:00326(	regulation	24/1638	160/18670	0.006581	0.043495	0.033361	AXL/BPI/C	24
BP	GO:00508(	defense re	17/1638	101/18670	0.00663	0.043762	0.033567	TNFSF8/CI	17
BP	GO:00487(	embryonic	20/1638	126/18670	0.006704	0.044019	0.033764	NKX3-2/C	20
BP	GO:00720(	nephron tu	16/1638	93/18670	0.006705	0.044019	0.033764	AGTR2/FG	16
BP	GO:00488(	genitalia d	10/1638	47/18670	0.006708	0.044019	0.033764	AR/AXL/ES	10
BP	GO:19031(	regulation	10/1638	47/18670	0.006708	0.044019	0.033764	ANK2/ATP	10
BP	GO:00343(	primary alc	15/1638	85/18670	0.006711	0.044019	0.033764	ADH1B/AD	15
BP	GO:00019(	regulation	9/1638	40/18670	0.006778	0.04424	0.033933	AGTR1/AG	9

BP	GO:00076:	mating	9/1638	40/18670	0.006778	0.04424	0.033933	AVP/CNR1	9
BP	GO:00091	cyclic nucl	9/1638	40/18670	0.006778	0.04424	0.033933	ADCY2/AD	9
BP	GO:00508	defense re	9/1638	40/18670	0.006778	0.04424	0.033933	CTSG/ELAI	9
BP	GO:00018	negative re	39/1638	296/18670	0.006841	0.044601	0.03421	ABCD2/AP	39
BP	GO:00108	regulation	12/1638	62/18670	0.006955	0.045174	0.03465	ADGRB1/N	12
BP	GO:00316	killing of c	12/1638	62/18670	0.006955	0.045174	0.03465	CTSG/DEFI	12
BP	GO:00443	disruption	12/1638	62/18670	0.006955	0.045174	0.03465	CTSG/DEFI	12
BP	GO:00192	regulation	51/1638	410/18670	0.007036	0.04564	0.035007	AGTR1/AB	51
BP	GO:00512	positive re	43/1638	334/18670	0.00705	0.04568	0.035038	AXL/CAV1	43
BP	GO:00060	glycosamir	18/1638	110/18670	0.007152	0.046283	0.0355	BGN/VCAI	18
BP	GO:00603	innervatior	7/1638	27/18670	0.007312	0.047041	0.036082	GABRA5/I	7
BP	GO:00170	protein nit	5/1638	15/18670	0.007341	0.047041	0.036082	ATP2B4/DI	5
BP	GO:00181	peptidyl-c	5/1638	15/18670	0.007341	0.047041	0.036082	ATP2B4/DI	5
BP	GO:00336	negative re	5/1638	15/18670	0.007341	0.047041	0.036082	AGTR2/CN	5
BP	GO:00506	dermatan	5/1638	15/18670	0.007341	0.047041	0.036082	BGN/VCAI	5
BP	GO:00605	ventral spi	5/1638	15/18670	0.007341	0.047041	0.036082	EVX1/GLI2	5
BP	GO:00605	cell fate cc	5/1638	15/18670	0.007341	0.047041	0.036082	EVX1/GLI2	5
BP	GO:19018	regulation	5/1638	15/18670	0.007341	0.047041	0.036082	CACNA2D	5
BP	GO:00032	ventricular	11/1638	55/18670	0.007439	0.047334	0.036306	COL11A1/	11
BP	GO:00435	regulation	11/1638	55/18670	0.007439	0.047334	0.036306	CD19/CCR	11
BP	GO:00613	renal tubul	14/1638	78/18670	0.007439	0.047334	0.036306	AGTR2/FG	14
BP	GO:00060	proteoglyc	16/1638	94/18670	0.00744	0.047334	0.036306	BGN/COL1	16
BP	GO:00609	kidney mo	16/1638	94/18670	0.00744	0.047334	0.036306	AGTR2/FG	16
BP	GO:00701	regulation	16/1638	94/18670	0.00744	0.047334	0.036306	AHSG/COI	16
BP	GO:00459	positive re	36/1638	270/18670	0.007577	0.048148	0.03693	AVP/ERBB	36
BP	GO:00019	regulation	19/1638	119/18670	0.0076	0.048235	0.036997	APOD/CD	19
BP	GO:00109	regulation	60/1638	499/18670	0.007642	0.048441	0.037155	ADCYAP1/	60
BP	GO:01100	regulation	35/1638	261/18670	0.007678	0.048611	0.037286	ACTN2/AD	35
BP	GO:00307	ovulation	6/1638	21/18670	0.007742	0.048901	0.037508	AFP/INHB	6
BP	GO:00484	sympathet	6/1638	21/18670	0.007742	0.048901	0.037508	PHOX2A/N	6
BP	GO:00101	potassium	10/1638	48/18670	0.007822	0.049114	0.037671	ATP1A2/A	10
BP	GO:00163	calcium-d	10/1638	48/18670	0.007822	0.049114	0.037671	CDH9/CDH	10
BP	GO:00432	positive re	10/1638	48/18670	0.007822	0.049114	0.037671	ACTN2/AN	10
BP	GO:00468	regulation	10/1638	48/18670	0.007822	0.049114	0.037671	CALCA/CS	10
BP	GO:00603	face devel	10/1638	48/18670	0.007822	0.049114	0.037671	ALDH1A3/	10
BP	GO:00480	embryonic	8/1638	34/18670	0.007914	0.049368	0.037866	ALDH1A3/	8
BP	GO:00316	positive re	12/1638	63/18670	0.007923	0.049368	0.037866	HGF/NTSR	12
BP	GO:00224	regulation	50/1638	403/18670	0.007931	0.049368	0.037866	APOA1/CA	50
BP	GO:00456	negative re	31/1638	225/18670	0.007963	0.049368	0.037866	DLX1/DLX	31
BP	GO:00425	retinol me	9/1638	41/18670	0.008023	0.049368	0.037866	ADH1B/AD	9
BP	GO:00432	negative re	9/1638	41/18670	0.008023	0.049368	0.037866	ACTN2/AD	9
BP	GO:00482	lung alveo	9/1638	41/18670	0.008023	0.049368	0.037866	FGF10/FO	9
BP	GO:00902	regulation	9/1638	41/18670	0.008023	0.049368	0.037866	CRH/GCG/	9
BP	GO:19013	positive re	9/1638	41/18670	0.008023	0.049368	0.037866	ACTN2/AN	9
BP	GO:00017	establishm	4/1638	10/18670	0.00805	0.049368	0.037866	CCR7/CCL	4
BP	GO:00353	sterol imp	4/1638	10/18670	0.00805	0.049368	0.037866	APOA1/AP	4
BP	GO:00445	relaxation	4/1638	10/18670	0.00805	0.049368	0.037866	GUCY1A1/	4
BP	GO:00510	positive re	4/1638	10/18670	0.00805	0.049368	0.037866	APOA4/AP	4
BP	GO:00603	bone trabe	4/1638	10/18670	0.00805	0.049368	0.037866	COL1A1/M	4
BP	GO:00700	glucagon	4/1638	10/18670	0.00805	0.049368	0.037866	CRH/IL6/LI	4
BP	GO:00705	cholestero	4/1638	10/18670	0.00805	0.049368	0.037866	APOA1/AP	4
BP	GO:00971	presynapti	4/1638	10/18670	0.00805	0.049368	0.037866	CEL/NLGN	4
BP	GO:00989	retrograde	4/1638	10/18670	0.00805	0.049368	0.037866	CNR1/FAB	4
BP	GO:20004	regulation	4/1638	10/18670	0.00805	0.049368	0.037866	C3/C4A/C	4
BP	GO:20008	positive re	4/1638	10/18670	0.00805	0.049368	0.037866	CRH/GALR	4
BP	GO:00071	leukocyte	43/1638	337/18670	0.008216	0.050287	0.038571	ADD2/APC	43
BP	GO:00550	cardiac cel	16/1638	95/18670	0.008238	0.050287	0.038571	ACTC1/AC	16
BP	GO:00613	renal tubul	16/1638	95/18670	0.008238	0.050287	0.038571	AGTR2/FG	16
BP	GO:01100	regulation	16/1638	95/18670	0.008238	0.050287	0.038571	APOA1/CC	16
BP	GO:00326	tumor nec	24/1638	163/18670	0.008268	0.050355	0.038623	AXL/BPI/C	24
BP	GO:19035	regulation	24/1638	163/18670	0.008268	0.050355	0.038623	AXL/BPI/C	24
BP	GO:00030	renal syste	19/1638	120/18670	0.008308	0.050508	0.03874	ADCY2/AD	19

BP	GO:00140(	positive re	15/1638	87/18670	0.008332	0.050508	0.03874	DCN/F2/F2	15
BP	GO:00458	positive re	15/1638	87/18670	0.008332	0.050508	0.03874	FGF2/FGFF	15
BP	GO:00486	positive re	15/1638	87/18670	0.008332	0.050508	0.03874	FGF2/FGFF	15
BP	GO:00605(	developme	32/1638	235/18670	0.008412	0.050938	0.03907	S1PR1/EPH	32
BP	GO:00480	phosphatic	26/1638	181/18670	0.008476	0.051207	0.039276	CSF1R/DC	26
BP	GO:00511	regulation	26/1638	181/18670	0.008476	0.051207	0.039276	ADGRB1/N	26
BP	GO:00007(	syncytium	11/1638	56/18670	0.008541	0.051483	0.039488	ADGRB1/A	11
BP	GO:01402	cell-cell fu	11/1638	56/18670	0.008541	0.051483	0.039488	ADGRB1/A	11
BP	GO:19035	mucopolys	18/1638	112/18670	0.008614	0.05186	0.039777	BGN/VCAI	18
BP	GO:00148	muscle cel	17/1638	104/18670	0.008884	0.053425	0.040978	IGF1/IGFBI	17
BP	GO:00435	regulation	12/1638	64/18670	0.008993	0.053604	0.041116	CD19/CCR	12
BP	GO:00465(	glycerolipi	12/1638	64/18670	0.008993	0.053604	0.041116	APOA1/AF	12
BP	GO:00482	lymphocyt	12/1638	64/18670	0.008993	0.053604	0.041116	S100A7/C(	12
BP	GO:19004	regulation	12/1638	64/18670	0.008993	0.053604	0.041116	ACTN2/CR	12
BP	GO:19036	positive re	12/1638	64/18670	0.008993	0.053604	0.041116	AGTR1/AP	12
BP	GO:00026	regulation	7/1638	28/18670	0.009026	0.053604	0.041116	ELANE/IL1	7
BP	GO:00031	aortic valv	7/1638	28/18670	0.009026	0.053604	0.041116	ELN/SLIT3,	7
BP	GO:00336	regulation	7/1638	28/18670	0.009026	0.053604	0.041116	CCN1/NPF	7
BP	GO:00343	plasma lip	7/1638	28/18670	0.009026	0.053604	0.041116	APOA1/AF	7
BP	GO:00457(	regulation	7/1638	28/18670	0.009026	0.053604	0.041116	CACNA1C,	7
BP	GO:20004(	positive re	7/1638	28/18670	0.009026	0.053604	0.041116	RELN/RGS	7
BP	GO:00718	protein-lip	10/1638	49/18670	0.009074	0.053832	0.04129	AGTR1/AP	10
BP	GO:00480	vascular er	16/1638	96/18670	0.009103	0.053943	0.041375	AXL/FGF1(	16
BP	GO:00511	positive re	13/1638	72/18670	0.009231	0.054639	0.041909	ADGRB1/I(	13
BP	GO:19018(	positive re	15/1638	88/18670	0.009251	0.054639	0.041909	FGF2/FGFF	15
BP	GO:19018	regulation	15/1638	88/18670	0.009251	0.054639	0.041909	APOD/CA\	15
BP	GO:00996	cardiac mu	9/1638	42/18670	0.009435	0.055658	0.042691	ANK2/CAC	9
BP	GO:01101	negative re	8/1638	35/18670	0.009488	0.055909	0.042884	CPB2/FOX	8
BP	GO:00075	response t	30/1638	219/18670	0.009662	0.056874	0.043623	ALDH3A1/	30
BP	GO:00020	sprouting	26/1638	183/18670	0.009735	0.057238	0.043903	AGTR1/AP	26
BP	GO:00066	triglyceride	17/1638	105/18670	0.009758	0.057252	0.043914	APOA1/AF	17
BP	GO:19011	carbohydr	27/1638	192/18670	0.009759	0.057252	0.043914	APOBEC1/	27
BP	GO:00091	cyclic nucl	6/1638	22/18670	0.009869	0.057351	0.043989	ADCY2/AD	6
BP	GO:00215	spinal corc	6/1638	22/18670	0.009869	0.057351	0.043989	EVX1/GLI2	6
BP	GO:00312	positive re	6/1638	22/18670	0.009869	0.057351	0.043989	CACNA1C,	6
BP	GO:00456(	positive re	6/1638	22/18670	0.009869	0.057351	0.043989	MYF6/RIP(	6
BP	GO:00526	cyclic purir	6/1638	22/18670	0.009869	0.057351	0.043989	ADCY2/AD	6
BP	GO:00017	neutrophil	5/1638	16/18670	0.009917	0.057351	0.043989	AXL/IL6/IT	5
BP	GO:00069	compleme	5/1638	16/18670	0.009917	0.057351	0.043989	C3/C7/CR	5
BP	GO:00430	negative re	5/1638	16/18670	0.009917	0.057351	0.043989	BPI/FCGR2	5
BP	GO:00506	dermatan	5/1638	16/18670	0.009917	0.057351	0.043989	BGN/VCAI	5
BP	GO:00512	sequesteri	5/1638	16/18670	0.009917	0.057351	0.043989	LCN2/S10(	5
BP	GO:00705	dendrite se	5/1638	16/18670	0.009917	0.057351	0.043989	CNTN2/PA	5
BP	GO:19051	regulation	5/1638	16/18670	0.009917	0.057351	0.043989	C3/CD36/I	5
BP	GO:20008	regulation	5/1638	16/18670	0.009917	0.057351	0.043989	AGTR1/AG	5
BP	GO:00069	regulation	12/1638	65/18670	0.010172	0.058693	0.045019	ATP1A2/C,	12
BP	GO:00726	T cell migr	12/1638	65/18670	0.010172	0.058693	0.045019	APOD/S1P	12
BP	GO:00071	adenylate	15/1638	89/18670	0.01025	0.058837	0.045129	ADCY2/AD	15
BP	GO:00326(	chemokine	15/1638	89/18670	0.01025	0.058837	0.045129	ADCYAP1/	15
BP	GO:00464	positive re	15/1638	89/18670	0.01025	0.058837	0.045129	CSF1R/CYI	15
BP	GO:19010	regulation	15/1638	89/18670	0.01025	0.058837	0.045129	ANK2/ATP	15
BP	GO:00468	positive re	20/1638	131/18670	0.010252	0.058837	0.045129	ADCYAP1/	20
BP	GO:00006(	superoxide	13/1638	73/18670	0.010354	0.05922	0.045423	APOA4/CD	13
BP	GO:00436	response t	13/1638	73/18670	0.010354	0.05922	0.045423	APOA1/AF	13
BP	GO:19035	positive re	13/1638	73/18670	0.010354	0.05922	0.045423	AVP/AVPR	13
BP	GO:00026	positive re	47/1638	380/18670	0.010364	0.05922	0.045423	AXL/CAV1	47
BP	GO:00107	positive re	10/1638	50/18670	0.010473	0.059715	0.045803	COL1A1/IL	10
BP	GO:00451(	intermedia	10/1638	50/18670	0.010473	0.059715	0.045803	DES/GFAP,	10
BP	GO:00350	regulation	21/1638	140/18670	0.010546	0.060066	0.046072	APOA1/AF	21
BP	GO:19029(	regulation	44/1638	352/18670	0.010744	0.061124	0.046883	ACTN2/AD	44
BP	GO:00485	eye morph	22/1638	149/18670	0.010772	0.061222	0.046958	ALDH1A3/	22
BP	GO:00030	respiratory	7/1638	29/18670	0.011016	0.061988	0.047546	ATP1A2/N	7

BP	GO:00108	regulation	7/1638	29/18670	0.011016	0.061988	0.047546	ANK2/ATP	7
BP	GO:00322	negative re	7/1638	29/18670	0.011016	0.061988	0.047546	S1PR1/MY	7
BP	GO:00508	regulation	7/1638	29/18670	0.011016	0.061988	0.047546	BLK/CD19/	7
BP	GO:00550	potassium	7/1638	29/18670	0.011016	0.061988	0.047546	ATP1A2/A	7
BP	GO:00604	epithelial t	7/1638	29/18670	0.011016	0.061988	0.047546	FGF10/FO	7
BP	GO:00019	negative re	9/1638	43/18670	0.011026	0.061988	0.047546	APOD/MY	9
BP	GO:00217	cerebral co	9/1638	43/18670	0.011026	0.061988	0.047546	GLI3/POU	9
BP	GO:00436	keratinocy	9/1638	43/18670	0.011026	0.061988	0.047546	KLF9/FGF7	9
BP	GO:00860	regulation	9/1638	43/18670	0.011026	0.061988	0.047546	ANK2/ATP	9
BP	GO:00424	inner ear n	16/1638	98/18670	0.011048	0.061988	0.047546	ALDH1A3/	16
BP	GO:00508	regulation	16/1638	98/18670	0.011048	0.061988	0.047546	ATP2B4/C/	16
BP	GO:00072	Rho protei	28/1638	203/18670	0.011088	0.062006	0.04756	AGTR1/AP	28
BP	GO:00425	myelinatio	20/1638	132/18670	0.011117	0.062006	0.04756	ABCD2/AN	20
BP	GO:00455	regulation	20/1638	132/18670	0.011117	0.062006	0.04756	RUNX1T1/	20
BP	GO:00459	regulation	20/1638	132/18670	0.011117	0.062006	0.04756	APOA1/AF	20
BP	GO:00069	syncytium	11/1638	58/18670	0.011122	0.062006	0.04756	ADGRB1/A	11
BP	GO:00713	cellular res	11/1638	58/18670	0.011122	0.062006	0.04756	ADCYAP1/	11
BP	GO:00060	aminoglyc	18/1638	115/18670	0.011246	0.062632	0.04804	BGN/VCAN	18
BP	GO:00104	regulation	8/1638	36/18670	0.011283	0.06264	0.048046	AR/FGF2/F	8
BP	GO:19026	regulation	8/1638	36/18670	0.011283	0.06264	0.048046	CCR7/IL1R	8
BP	GO:00016	metanephi	15/1638	90/18670	0.011331	0.06264	0.048046	AGTR2/FBI	15
BP	GO:00099	dorsal/ven	15/1638	90/18670	0.011331	0.06264	0.048046	EVX1/GLI1	15
BP	GO:00140	neural cres	15/1638	90/18670	0.011331	0.06264	0.048046	ANXA6/ED	15
BP	GO:00343	adherens j	15/1638	90/18670	0.011331	0.06264	0.048046	ACTN2/AP	15
BP	GO:00424	odontoger	15/1638	90/18670	0.011331	0.06264	0.048046	DLX1/DLX	15
BP	GO:19048	cranial ske	12/1638	66/18670	0.011467	0.063326	0.048572	DLX2/FOX	12
BP	GO:00302	negative re	14/1638	82/18670	0.01152	0.063485	0.048694	AHSG/GDI	14
BP	GO:00551	regulation	14/1638	82/18670	0.01152	0.063485	0.048694	ANK2/ATP	14
BP	GO:00316	cellular res	13/1638	74/18670	0.01158	0.063751	0.048899	COL1A1/F	13
BP	GO:00017	establishm	4/1638	11/18670	0.011781	0.063794	0.048931	CCR7/CCL	4
BP	GO:00061	cGMP bios	4/1638	11/18670	0.011781	0.063794	0.048931	GUCY1A1/	4
BP	GO:00215	ventral spi	4/1638	11/18670	0.011781	0.063794	0.048931	EVX1/GLI2	4
BP	GO:00218	cerebral co	4/1638	11/18670	0.011781	0.063794	0.048931	DLX1/DLX	4
BP	GO:00322	regulation	4/1638	11/18670	0.011781	0.063794	0.048931	CRH/INHB	4
BP	GO:00336	negative re	4/1638	11/18670	0.011781	0.063794	0.048931	CYP1B1/SE	4
BP	GO:00359	glucocortic	4/1638	11/18670	0.011781	0.063794	0.048931	CRH/GALR	4
BP	GO:00452	cell-cell ju	4/1638	11/18670	0.011781	0.063794	0.048931	CSF1R/F2R	4
BP	GO:00603	cardiac mu	4/1638	11/18670	0.011781	0.063794	0.048931	ISL1/GREM	4
BP	GO:00605	cell fate sp	4/1638	11/18670	0.011781	0.063794	0.048931	EVX1/GLI2	4
BP	GO:00608	establishm	4/1638	11/18670	0.011781	0.063794	0.048931	TTPA/RECI	4
BP	GO:00725	fibroblast a	4/1638	11/18670	0.011781	0.063794	0.048931	LEP/PDGFI	4
BP	GO:00988	synapse pr	4/1638	11/18670	0.011781	0.063794	0.048931	C3/CX3CR	4
BP	GO:00991	regulation	4/1638	11/18670	0.011781	0.063794	0.048931	CBLN1/NT	4
BP	GO:19025	regulation	4/1638	11/18670	0.011781	0.063794	0.048931	FCGR2B/IT	4
BP	GO:19059	regulation	4/1638	11/18670	0.011781	0.063794	0.048931	FGFR1/SFR	4
BP	GO:00015	regulation	44/1638	354/18670	0.011804	0.063849	0.048973	AGTR2/AT	44
BP	GO:00019	cell killing	24/1638	168/18670	0.011854	0.064054	0.04913	C3/CD1C/	24
BP	GO:00508	antigen re	40/1638	316/18670	0.011912	0.064303	0.049322	BLK/CD19/	40
BP	GO:00100	response t	45/1638	364/18670	0.012021	0.064585	0.049538	APBB1/AP	45
BP	GO:00215	cranial ner	10/1638	51/18670	0.012031	0.064585	0.049538	PHOX2A/C	10
BP	GO:00509	regulation	10/1638	51/18670	0.012031	0.064585	0.049538	AGTR2/AT	10
BP	GO:00514	positive re	10/1638	51/18670	0.012031	0.064585	0.049538	APOA1/CC	10
BP	GO:00973	response t	10/1638	51/18670	0.012031	0.064585	0.049538	CCR7/CNR	10
BP	GO:00072	smoothen	20/1638	133/18670	0.012038	0.064585	0.049538	EVC/FGF1	20
BP	GO:00451	developme	30/1638	223/18670	0.012317	0.066018	0.050637	ADCYAP1/	30
BP	GO:00095	detection	6/1638	23/18670	0.012382	0.066027	0.050644	C4B/TREM	6
BP	GO:00305	male genit	6/1638	23/18670	0.012382	0.066027	0.050644	AR/FGF10/	6
BP	GO:00451	intermedia	6/1638	23/18670	0.012382	0.066027	0.050644	DES/GFAP	6
BP	GO:00513	positive re	6/1638	23/18670	0.012382	0.066027	0.050644	CACNA1C/	6
BP	GO:20000	regulation	6/1638	23/18670	0.012382	0.066027	0.050644	SFRP1/SFR	6
BP	GO:00230	signal rele	55/1638	462/18670	0.012439	0.066266	0.050827	ADCY5/AD	55
BP	GO:00149	smooth mu	15/1638	91/18670	0.012499	0.066519	0.051021	IGF1/IGFBI	15

BP	GO:00468	filopodium	11/1638	59/18670	0.012617	0.067004	0.051393	CCR7/DPY	11
BP	GO:00977	positive re	11/1638	59/18670	0.012617	0.067004	0.051393	ADCYAP1/	11
BP	GO:19033	regulation	23/1638	160/18670	0.012628	0.067004	0.051393	CACNB2/C	23
BP	GO:19038	negative re	14/1638	83/18670	0.012771	0.067693	0.051922	CAV1/CIDI	14
BP	GO:19046	glucose tra	17/1638	108/18670	0.012798	0.067697	0.051925	C3/EDNRA	17
BP	GO:00031	endocardia	9/1638	44/18670	0.01281	0.067697	0.051925	FOXF1/ISL	9
BP	GO:00329	regulation	9/1638	44/18670	0.01281	0.067697	0.051925	CCN2/F2/	9
BP	GO:00600	cardiac mu	12/1638	67/18670	0.012885	0.068025	0.052177	NKX2-5/F	12
BP	GO:00017	mesoderm	13/1638	75/18670	0.012916	0.06805	0.052195	FGFR1/FO	13
BP	GO:00074	peripheral	13/1638	75/18670	0.012916	0.06805	0.052195	ADGRB1/E	13
BP	GO:00072	ensheathm	20/1638	134/18670	0.013019	0.06829	0.05238	ABCD2/AN	20
BP	GO:00083	axon ensh	20/1638	134/18670	0.013019	0.06829	0.05238	ABCD2/AN	20
BP	GO:00026	positive re	5/1638	17/18670	0.013052	0.06829	0.05238	ELANE/IL1	5
BP	GO:00215	ventral spi	5/1638	17/18670	0.013052	0.06829	0.05238	EVX1/GLI2	5
BP	GO:00359	chondrocy	5/1638	17/18670	0.013052	0.06829	0.05238	COMP/CC	5
BP	GO:00361	phosphatic	5/1638	17/18670	0.013052	0.06829	0.05238	PLA2G1B/	5
BP	GO:19049	midbrain c	5/1638	17/18670	0.013052	0.06829	0.05238	LMX1A/SF	5
BP	GO:00900	regulation	7/1638	30/18670	0.013305	0.069146	0.053037	CCR7/CCL	7
BP	GO:00600	excitatory	16/1638	100/18670	0.013307	0.069146	0.053037	CBLN1/CH	16
BP	GO:20010	regulation	16/1638	100/18670	0.013307	0.069146	0.053037	ATP1A2/C	16
BP	GO:00031	regulation	8/1638	37/18670	0.013316	0.069146	0.053037	AR/FGF10/	8
BP	GO:00310	embryonic	8/1638	37/18670	0.013316	0.069146	0.053037	ALDH1A3/	8
BP	GO:00459	positive re	8/1638	37/18670	0.013316	0.069146	0.053037	ABCD2/AP	8
BP	GO:00603	head morp	8/1638	37/18670	0.013316	0.069146	0.053037	COL1A1/M	8
BP	GO:00486	regulation	43/1638	347/18670	0.013321	0.069146	0.053037	AR/NKX2-	43
BP	GO:00226	regulation	57/1638	484/18670	0.013634	0.070703	0.054231	APOA1/AD	57
BP	GO:00096	response t	10/1638	52/18670	0.013756	0.071004	0.054462	CTSG/ELA	10
BP	GO:00604	positive re	10/1638	52/18670	0.013756	0.071004	0.054462	FGF2/FGF	10
BP	GO:19000	regulation	10/1638	52/18670	0.013756	0.071004	0.054462	APOA1/FB	10
BP	GO:20001	positive re	10/1638	52/18670	0.013756	0.071004	0.054462	CX3CR1/F	10
BP	GO:19048	positive re	15/1638	92/18670	0.013759	0.071004	0.054462	CSF1R/CY	15
BP	GO:00511	positive re	17/1638	109/18670	0.013962	0.071979	0.055209	ADGRB1/I	17
BP	GO:00149	regulation	14/1638	84/18670	0.014125	0.072679	0.055746	IGF1/IGFB	14
BP	GO:00454	myoblast c	14/1638	84/18670	0.014125	0.072679	0.055746	IGF1/ISL1/	14
BP	GO:00518	regulation	11/1638	60/18670	0.01426	0.073229	0.056168	APOD/CO	11
BP	GO:00901	regulation	11/1638	60/18670	0.01426	0.073229	0.056168	APOD/CO	11
BP	GO:00140	negative re	9/1638	45/18670	0.0148	0.075857	0.058184	ADCYAP1/	9
BP	GO:00718	plasma lip	9/1638	45/18670	0.0148	0.075857	0.058184	AGTR1/AP	9
BP	GO:00084	gonad dev	29/1638	217/18670	0.01489	0.076244	0.058481	ADCYAP1/	29
BP	GO:00487	embryonic	15/1638	93/18670	0.015116	0.077323	0.059309	COL11A1/	15
BP	GO:00106	regulation	6/1638	24/18670	0.015311	0.078017	0.059841	APOD/F7/	6
BP	GO:00509	positive re	6/1638	24/18670	0.015311	0.078017	0.059841	ABCD2/AP	6
BP	GO:00860	cell comm	6/1638	24/18670	0.015311	0.078017	0.059841	ATP1A2/A	6
BP	GO:20010	regulation	6/1638	24/18670	0.015311	0.078017	0.059841	FGF2/FGF	6
BP	GO:00224	negative re	25/1638	181/18670	0.015487	0.078801	0.060442	APOA1/DT	25
BP	GO:00107	positive re	22/1638	154/18670	0.015494	0.078801	0.060442	APOA1/FG	22
BP	GO:00140	mesenchy	14/1638	85/18670	0.015587	0.079113	0.060681	ANXA6/ED	14
BP	GO:00507	positive re	14/1638	85/18670	0.015587	0.079113	0.060681	FN1/MAP1	14
BP	GO:19010	positive re	8/1638	38/18670	0.015604	0.079113	0.060681	ANK2/CAC	8
BP	GO:00100	response t	10/1638	53/18670	0.015661	0.079113	0.060681	APOBEC1/	10
BP	GO:00218	forebrain r	10/1638	53/18670	0.015661	0.079113	0.060681	CSF1R/DL	10
BP	GO:00316	zymogen a	10/1638	53/18670	0.015661	0.079113	0.060681	APOH/C1	10
BP	GO:19055	macropha	10/1638	53/18670	0.015661	0.079113	0.060681	CX3CR1/C	10
BP	GO:00099	hormone t	40/1638	322/18670	0.015906	0.079456	0.060944	ADCY5/AD	40
BP	GO:20003	positive re	16/1638	102/18670	0.015911	0.079456	0.060944	AGTR1/AG	16
BP	GO:00026	positive re	7/1638	31/18670	0.015917	0.079456	0.060944	C3/CCR7/	7
BP	GO:00034	axis elong	7/1638	31/18670	0.015917	0.079456	0.060944	ESR1/FGF1	7
BP	GO:00107	regulation	7/1638	31/18670	0.015917	0.079456	0.060944	FGF2/HAS	7
BP	GO:00336	osteoblast	7/1638	31/18670	0.015917	0.079456	0.060944	CCN1/NP	7
BP	GO:00443	wound hea	7/1638	31/18670	0.015917	0.079456	0.060944	COL5A1/F	7
BP	GO:00602	long-term	7/1638	31/18670	0.015917	0.079456	0.060944	CBLN1/DR	7
BP	GO:00860	membrane	7/1638	31/18670	0.015917	0.079456	0.060944	CACNA2D	7

BP	GO:00905(	epiboly inv	7/1638	31/18670	0.015917	0.079456	0.060944	COL5A1/FI	7
BP	GO:009962	ventricular	7/1638	31/18670	0.015917	0.079456	0.060944	ANK2/CAC	7
BP	GO:20004(	positive re	7/1638	31/18670	0.015917	0.079456	0.060944	S100A7/C(	7
BP	GO:004833	mesoderm	13/1638	77/18670	0.015939	0.079456	0.060944	FGFR1/FO)	13
BP	GO:005193	catecholan	13/1638	77/18670	0.015939	0.079456	0.060944	AGTR2/CN	13
BP	GO:004273	embryonic	11/1638	61/18670	0.016059	0.079899	0.061284	GLI2/GLI3/	11
BP	GO:007138	cellular res	11/1638	61/18670	0.016059	0.079899	0.061284	ADCYAP1/	11
BP	GO:001059	positive re	19/1638	128/18670	0.01608	0.079931	0.061308	FGF2/FGFI	19
BP	GO:00713(	cellular res	12/1638	69/18670	0.01612	0.080054	0.061403	COL1A1/E	12
BP	GO:00026	negative re	4/1638	12/18670	0.016463	0.081373	0.062414	ADCYAP1/	4
BP	GO:00028(	negative re	4/1638	12/18670	0.016463	0.081373	0.062414	ADCYAP1/	4
BP	GO:000292	negative re	4/1638	12/18670	0.016463	0.081373	0.062414	A2M/SERP	4
BP	GO:00860(	AV node c	4/1638	12/18670	0.016463	0.081373	0.062414	CACNA1C/	4
BP	GO:00995(	regulation	4/1638	12/18670	0.016463	0.081373	0.062414	GRIA1/ITPI	4
BP	GO:00016	temperatu	24/1638	173/18670	0.016595	0.081947	0.062855	ADCYAP1/	24
BP	GO:005189	negative re	5/1638	18/18670	0.016792	0.08277	0.063486	APOD/THE	5
BP	GO:009709	craniofacia	5/1638	18/18670	0.016792	0.08277	0.063486	GLI3/MMP	5
BP	GO:004688	regulation	34/1638	266/18670	0.016845	0.08295	0.063624	ADCY5/AD	34
BP	GO:00096	response t	28/1638	210/18670	0.016975	0.083369	0.063945	ATP1A2/C(	28
BP	GO:000243	inflammat	9/1638	46/18670	0.017009	0.083369	0.063945	ADCYAP1/	9
BP	GO:001973	antibacteri	9/1638	46/18670	0.017009	0.083369	0.063945	CTSG/DEFI	9
BP	GO:003362	regulation	9/1638	46/18670	0.017009	0.083369	0.063945	CYP1B1/F(	9
BP	GO:00457	negative re	9/1638	46/18670	0.017009	0.083369	0.063945	ADRB3/AG	9
BP	GO:006029	long-term	14/1638	86/18670	0.017162	0.084042	0.064462	CRH/CX3C	14
BP	GO:19021(	negative re	16/1638	103/18670	0.017353	0.084761	0.065014	DTX1/ERBI	16
BP	GO:000663	acylglycerc	19/1638	129/18670	0.017357	0.084761	0.065014	APOA1/AF	19
BP	GO:003475	cellular ho	19/1638	129/18670	0.017357	0.084761	0.065014	ADH1B/AD	19
BP	GO:001082	regulation	13/1638	78/18670	0.017639	0.085979	0.065948	C3/EDNRA	13
BP	GO:002195	central ner	13/1638	78/18670	0.017639	0.085979	0.065948	CDH11/GL	13
BP	GO:007169	anatomica	23/1638	165/18670	0.017774	0.086558	0.066392	ADGRB3/C	23
BP	GO:003272	negative re	12/1638	70/18670	0.01795	0.086869	0.06663	AXL/BPI/C	12
BP	GO:00507(	positive re	12/1638	70/18670	0.01795	0.086869	0.06663	AHSG/APC	12
BP	GO:006039	SMAD pro	12/1638	70/18670	0.01795	0.086869	0.06663	AFP/BMP3	12
BP	GO:00032	cardiac sep	17/1638	112/18670	0.017954	0.086869	0.06663	ANK2/NK	17
BP	GO:00086	hexose tra	17/1638	112/18670	0.017954	0.086869	0.06663	C3/EDNRA	17
BP	GO:001715	regulation	17/1638	112/18670	0.017954	0.086869	0.06663	CACNB2/C	17
BP	GO:004859	camera-ty	17/1638	112/18670	0.017954	0.086869	0.06663	ALDH1A3/	17
BP	GO:005502	positive re	11/1638	62/18670	0.018022	0.087121	0.066823	FGF2/FGFI	11
BP	GO:00107(	fibroblast	8/1638	39/18670	0.018163	0.087561	0.067161	FGF2/HAS	8
BP	GO:00381	neurotropl	8/1638	39/18670	0.018163	0.087561	0.067161	AGTR2/ND	8
BP	GO:005193	regulation	8/1638	39/18670	0.018163	0.087561	0.067161	F2R/FABP5	8
BP	GO:00019	lymph ves	6/1638	25/18670	0.018686	0.089634	0.068751	FOXC2/PR	6
BP	GO:00020	positive re	6/1638	25/18670	0.018686	0.089634	0.068751	FGFR1/FO)	6
BP	GO:00310(	hair follicle	6/1638	25/18670	0.018686	0.089634	0.068751	FGF7/FGF1	6
BP	GO:00722(	cell differe	6/1638	25/18670	0.018686	0.089634	0.068751	POU3F3/T	6
BP	GO:009002	regulation	6/1638	25/18670	0.018686	0.089634	0.068751	SERPINE1/	6
BP	GO:004863	positive re	25/1638	184/18670	0.018703	0.089634	0.068751	FGF2/FGFI	25
BP	GO:000663	neutral lipi	19/1638	130/18670	0.018712	0.089634	0.068751	APOA1/AF	19
BP	GO:00019	response t	7/1638	32/18670	0.018872	0.08977	0.068855	CNR2/DRD	7
BP	GO:00031	aortic valv	7/1638	32/18670	0.018872	0.08977	0.068855	ELN/SLIT3/	7
BP	GO:00076	long-term	7/1638	32/18670	0.018872	0.08977	0.068855	GRIA1/NF/	7
BP	GO:00425	retinoic ac	7/1638	32/18670	0.018872	0.08977	0.068855	ADH1B/AD	7
BP	GO:006138	heart trabe	7/1638	32/18670	0.018872	0.08977	0.068855	NKX2-5/S	7
BP	GO:00905(	epiboly	7/1638	32/18670	0.018872	0.08977	0.068855	COL5A1/FI	7
BP	GO:009962	regulation	7/1638	32/18670	0.018872	0.08977	0.068855	ANK2/CAC	7
BP	GO:000243	immune re	20/1638	139/18670	0.018911	0.08977	0.068855	FCGR2B/IG	20
BP	GO:003809	Fc-gamma	20/1638	139/18670	0.018911	0.08977	0.068855	FCGR2B/IG	20
BP	GO:005158	regulation	20/1638	139/18670	0.018911	0.08977	0.068855	ATP1A2/C/	20
BP	GO:000279	negative re	21/1638	148/18670	0.019012	0.090169	0.069161	APOA1/AF	21
BP	GO:190595	regulation	22/1638	157/18670	0.01903	0.090174	0.069166	AGTR1/AG	22
BP	GO:004662	regulation	17/1638	113/18670	0.019463	0.092107	0.070648	NKX2-5/F(	17
BP	GO:00015	vasculoger	13/1638	79/18670	0.019473	0.092107	0.070648	APLNR/CA	13

BP	GO:000279	positive re	36/1638	288/18670	0.019536	0.092323	0.070813	ADCYAP1/	36
BP	GO:00995(	synaptic ve	26/1638	194/18670	0.019639	0.092725	0.071122	AMPH/CA(	26
BP	GO:00327ᕵ	positive re	15/1638	96/18670	0.019809	0.093445	0.071674	ADCYAP1/	15
BP	GO:00425	positive re	12/1638	71/18670	0.019932	0.093944	0.072057	CSF1R/FLT	12
BP	GO:00431	receptor cl	10/1638	55/18670	0.020046	0.094311	0.072339	FLNA/GLR	10
BP	GO:00442	sulfur com	10/1638	55/18670	0.020046	0.094311	0.072339	BGN/CDO	10
BP	GO:00354	protein loc	11/1638	63/18670	0.020159	0.094756	0.07268	GRIN2A/M	11
BP	GO:19029(	positive re	27/1638	204/18670	0.020509	0.096319	0.073878	ACTN2/AF	27
BP	GO:00070(	plasma me	16/1638	105/18670	0.020535	0.096354	0.073905	ABCD2/AN	16
BP	GO:00508	defense re	14/1638	88/18670	0.020673	0.096914	0.074335	ADGRB1/B	14
BP	GO:20003	regulation	26/1638	195/18670	0.020833	0.097579	0.074845	AGTR1/AG	26
BP	GO:00454	fat cell diff	29/1638	223/18670	0.020901	0.097812	0.075024	RUNX1T1/	29
BP	GO:00198	cytolysis	8/1638	40/18670	0.021009	0.098143	0.075278	C7/F2/GZN	8
BP	GO:00519	regulation	8/1638	40/18670	0.021009	0.098143	0.075278	F2R/FABP5	8
BP	GO:00157	monosacch	17/1638	114/18670	0.021068	0.09816	0.075291	C3/EDNRA	17
BP	GO:00017	mesoderm	5/1638	19/18670	0.021181	0.09816	0.075291	FGFR1/FO>	5
BP	GO:00025	chronic inf	5/1638	19/18670	0.021181	0.09816	0.075291	S100A8/C(	5
BP	GO:00029	positive re	5/1638	19/18670	0.021181	0.09816	0.075291	C3/CCR7/F	5
BP	GO:00309	positive re	5/1638	19/18670	0.021181	0.09816	0.075291	FGF10/ITG	5
BP	GO:00484	parasympa	5/1638	19/18670	0.021181	0.09816	0.075291	PHOX2A/N	5
BP	GO:00985	detection (	5/1638	19/18670	0.021181	0.09816	0.075291	C4B/TREM	5
BP	GO:00989	regulation	5/1638	19/18670	0.021181	0.09816	0.075291	CACNA1C/	5
BP	GO:19017	positive re	5/1638	19/18670	0.021181	0.09816	0.075291	ADGRB1/N	5
CC	GO:00620	collagen-c	158/1730	406/19717	3.36E-63	1.80E-60	1.34E-60	A2M/AEBF	158
CC	GO:00444	extracellula	27/1730	51/19717	6.91E-16	1.33E-13	9.88E-14	COL1A1/C	27
CC	GO:00057	endoplasm	74/1730	309/19717	7.43E-16	1.33E-13	9.88E-14	AFP/AHSG	74
CC	GO:00444	contractile	58/1730	221/19717	1.45E-14	1.95E-12	1.45E-12	ACTA2/AC	58
CC	GO:00432	contractile	59/1730	234/19717	5.88E-14	6.30E-12	4.69E-12	ACTA2/AC	59
CC	GO:00300	sarcomere	54/1730	204/19717	8.03E-14	6.89E-12	5.13E-12	ACTC1/AC	54
CC	GO:00055	collagen tr	33/1730	87/19717	9.52E-14	6.89E-12	5.13E-12	COL1A1/C	33
CC	GO:00300	myofibril	57/1730	224/19717	1.03E-13	6.89E-12	5.13E-12	ACTC1/AC	57
CC	GO:00198	immunogl	46/1730	159/19717	1.71E-13	1.02E-11	7.59E-12	CD79A/CD	46
CC	GO:00098	external si	81/1730	393/19717	2.38E-13	1.28E-11	9.51E-12	CXCR5/CA	81
CC	GO:00056	basement	34/1730	95/19717	2.92E-13	1.42E-11	1.06E-11	COL4A2/C	34
CC	GO:00970	synaptic m	86/1730	432/19717	3.32E-13	1.48E-11	1.10E-11	ACTN2/AN	86
CC	GO:00725	blood micr	41/1730	147/19717	1.29E-11	5.30E-10	3.95E-10	A2M/ACT(	41
CC	GO:00316	I band	40/1730	143/19717	2.07E-11	7.91E-10	5.89E-10	ACTC1/AC	40
CC	GO:00300	Z disc	38/1730	132/19717	2.54E-11	9.08E-10	6.76E-10	ACTN2/AN	38
CC	GO:00423	sarcolemm	37/1730	136/19717	2.77E-10	9.29E-09	6.92E-09	ANK2/ATP	37
CC	GO:00015	cornified e	24/1730	65/19717	4.27E-10	1.31E-08	9.73E-09	CSTA/DSC	24
CC	GO:00452	postsynapt	64/1730	323/19717	4.39E-10	1.31E-08	9.73E-09	ACTN2/AN	64
CC	GO:00310	platelet alp	28/1730	91/19717	1.97E-09	5.54E-08	4.13E-08	A2M/ACTN	28
CC	GO:00986	complex o	12/1730	19/19717	5.64E-09	1.51E-07	1.12E-07	COL1A1/C	12
CC	GO:00430	neuronal c	83/1730	497/19717	7.54E-09	1.92E-07	1.43E-07	ADCYAP1/	83
CC	GO:00988(	plasma me	56/1730	295/19717	2.60E-08	6.12E-07	4.56E-07	CD79A/CD	56
CC	GO:00989	glutamate	63/1730	349/19717	2.63E-08	6.12E-07	4.56E-07	ACTC1/AC	63
CC	GO:00310	platelet alp	22/1730	67/19717	2.78E-08	6.21E-07	4.62E-07	A2M/ACTN	22
CC	GO:00421(	T cell recep	32/1730	127/19717	3.30E-08	7.07E-07	5.26E-07	TRAV6/TRI	32
CC	GO:00992	intrinsic co	37/1730	164/19717	6.74E-08	1.39E-06	1.03E-06	ATP2B4/CI	37
CC	GO:00425	immunogl	22/1730	72/19717	1.19E-07	2.37E-06	1.76E-06	IGHD/IGH(	22
CC	GO:19024	transmem	57/1730	324/19717	2.93E-07	5.60E-06	4.17E-06	ATP1A2/A	57
CC	GO:00432(	perikaryon	31/1730	134/19717	4.24E-07	7.84E-06	5.84E-06	ADCYAP1/	31
CC	GO:00347(	cation cha	43/1730	220/19717	4.60E-07	8.21E-06	6.11E-06	CACNA1C/	43
CC	GO:00058	intermedia	42/1730	214/19717	5.55E-07	9.59E-06	7.14E-06	DES/GFAP/	42
CC	GO:00451	membrane	55/1730	315/19717	6.03E-07	1.01E-05	7.52E-06	ADCY2/AD	55
CC	GO:19903	transporte	57/1730	332/19717	6.68E-07	1.05E-05	7.85E-06	ATP1A2/A	57
CC	GO:00988	membrane	55/1730	316/19717	6.69E-07	1.05E-05	7.85E-06	ADCY2/AD	55
CC	GO:00059	cell-cell ju	71/1730	459/19717	1.76E-06	2.67E-05	1.99E-05	ADCYAP1F	71
CC	GO:00989	intrinsic co	28/1730	122/19717	1.79E-06	2.67E-05	1.99E-05	CDH9/CDH	28
CC	GO:00448	plasma me	26/1730	109/19717	1.92E-06	2.78E-05	2.07E-05	ADCYAP1F	26
CC	GO:00985	membrane	55/1730	328/19717	2.21E-06	3.12E-05	2.32E-05	ADCY2/AD	55
CC	GO:00996	integral co	32/1730	152/19717	2.54E-06	3.49E-05	2.59E-05	ATP2B4/CI	32

CC	GO:00451:	intermedia	45/1730	251/19717	3.00E-06	4.02E-05	2.99E-05	ADCY5/DE	45
CC	GO:00057!	Golgi lume	24/1730	102/19717	6.07E-06	7.94E-05	5.91E-05	BGN/CGA/	24
CC	GO:00989{	GABA-erg	19/1730	71/19717	7.68E-06	9.80E-05	7.30E-05	ATP2B3/CI	19
CC	GO:00347(	ion channe	50/1730	301/19717	8.23E-06	0.000103	7.64E-05	CACNA1C/	50
CC	GO:00427:	presynapti	32/1730	161/19717	9.06E-06	0.000108	8.07E-05	APBB1/ATI	32
CC	GO:00055{	fibrillar col	7/1730	11/19717	9.51E-06	0.000108	8.07E-05	COL1A1/C	7
CC	GO:00322{	symmetric	7/1730	11/19717	9.51E-06	0.000108	8.07E-05	CHRM2/NT	7
CC	GO:00986	banded co	7/1730	11/19717	9.51E-06	0.000108	8.07E-05	COL1A1/C	7
CC	GO:00312:	anchored (	33/1730	170/19717	1.11E-05	0.000124	9.24E-05	ART4/CA4/	33
CC	GO:00347	secretory g	52/1730	321/19717	1.14E-05	0.000124	9.24E-05	A2M/ACTN	52
CC	GO:00147(	intercalate	15/1730	50/19717	1.55E-05	0.000166	0.000124	ANK2/ATP	15
CC	GO:00056:	interstitial	7/1730	12/19717	2.11E-05	0.000222	0.000165	ECM2/TNC	7
CC	GO:00990!	integral co	25/1730	117/19717	2.35E-05	0.000241	0.000179	CDH9/CDH	25
CC	GO:00602(	cytoplasmi	53/1730	338/19717	2.38E-05	0.000241	0.000179	A2M/ACTN	53
CC	GO:00319{	vesicle lum	53/1730	339/19717	2.59E-05	0.000254	0.000189	A2M/ACTN	53
CC	GO:00303:	T-tubule	15/1730	52/19717	2.61E-05	0.000254	0.000189	ANK2/ATP	15
CC	GO:00426	actomyosi	19/1730	79/19717	3.97E-05	0.00038	0.000283	ACTC1/LPI	19
CC	GO:00426:	chylomicro	7/1730	13/19717	4.22E-05	0.000397	0.000295	APOA1/AF	7
CC	GO:00059(	caveola	19/1730	80/19717	4.78E-05	0.000431	0.000321	ADCYAP1F	19
CC	GO:00017:	stress fiber	17/1730	67/19717	4.82E-05	0.000431	0.000321	LPP/MYLK/	17
CC	GO:00975:	contractile	17/1730	67/19717	4.82E-05	0.000431	0.000321	LPP/MYLK/	17
CC	GO:00995	postsynap	53/1730	348/19717	5.33E-05	0.000468	0.000348	ACTN2/AD	53
CC	GO:00989{	neuron to	53/1730	350/19717	6.22E-05	0.000538	0.0004	ACTN2/AD	53
CC	GO:00324:	actin filam	18/1730	75/19717	6.48E-05	0.000551	0.00041	CRYAB/LPI	18
CC	GO:00300!	cell-substr	60/1730	412/19717	6.67E-05	0.000559	0.000416	ACTC1/AC	60
CC	GO:00988{	intrinsic co	19/1730	82/19717	6.87E-05	0.000566	0.000421	ATP2B4/CI	19
CC	GO:00347(	potassium	21/1730	96/19717	7.18E-05	0.000583	0.000434	DPP6/GRIK	21
CC	GO:00347(	sodium ch	9/1730	23/19717	7.91E-05	0.000633	0.000471	GRIK3/GRI	9
CC	GO:00322	asymmetri	50/1730	328/19717	8.36E-05	0.000659	0.00049	ACTN2/AD	50
CC	GO:00059:	cell-substr	59/1730	408/19717	9.33E-05	0.000725	0.000539	ACTC1/AC	59
CC	GO:00442!	cell-cell cc	17/1730	71/19717	0.000106	0.00081	0.000603	ANK2/ATP	17
CC	GO:00430:	costamere	8/1730	19/19717	0.000108	0.000816	0.000607	ANK2/DMI	8
CC	GO:00059:	focal adhe	58/1730	405/19717	0.000139	0.001035	0.00077	ACTC1/AC	58
CC	GO:00165:	sarcoplasn	18/1730	80/19717	0.000158	0.001158	0.000862	CACNA2D	18
CC	GO:00451	apical part	55/1730	384/19717	0.000204	0.001474	0.001097	ANK2/ATP	55
CC	GO:00083(	integrin co	10/1730	31/19717	0.000209	0.001476	0.001099	ITGA1/ITG/	10
CC	GO:00057	vacuolar lu	30/1730	172/19717	0.000209	0.001476	0.001099	APOB/BGN	30
CC	GO:00140(	postsynap	48/1730	324/19717	0.000229	0.001592	0.001185	ACTN2/AD	48
CC	GO:00163:	apical plas	47/1730	318/19717	0.000279	0.001919	0.001428	ANK2/ATP	47
CC	GO:00332(	axon part	54/1730	382/19717	0.000322	0.002184	0.001625	ADCYAP1/	54
CC	GO:00451	basal part	13/1730	51/19717	0.000348	0.002303	0.001714	ERBB2/FAI	13
CC	GO:00431!	dendritic s	29/1730	169/19717	0.000351	0.002303	0.001714	ACTN2/AP	29
CC	GO:00165:	sarcoplasn	16/1730	71/19717	0.000352	0.002303	0.001714	CACNA2D	16
CC	GO:00443(	neuron spi	29/1730	171/19717	0.00043	0.002759	0.002054	ACTN2/AP	29
CC	GO:00996:	postsynap	20/1730	101/19717	0.000432	0.002759	0.002054	ACTN2/CD	20
CC	GO:00466!	anchored (	14/1730	59/19717	0.000464	0.002926	0.002178	CA4/CNTN	14
CC	GO:00080	voltage-ga	18/1730	87/19717	0.000474	0.002952	0.002197	DPP6/KCN	18
CC	GO:00986:	protein co	10/1730	34/19717	0.000485	0.002986	0.002222	ITGA1/ITG/	10
CC	GO:00450!	keratin fila	19/1730	95/19717	0.000523	0.003185	0.00237	KRT4/KRT!	19
CC	GO:00443(	main axon	15/1730	68/19717	0.000678	0.004081	0.003038	ANK2/APB	15
CC	GO:00989	intrinsic co	7/1730	19/19717	0.000773	0.004606	0.003428	ATP2B4/CI	7
CC	GO:00487{	presynapti	9/1730	30/19717	0.000788	0.004642	0.003455	ATP2B4/CI	9
CC	GO:00431!	dendritic s	10/1730	36/19717	0.000799	0.004654	0.003464	FLNA/HTR	10
CC	GO:00015:	microfibril	5/1730	10/19717	0.000894	0.005154	0.003836	FBN1/LTBI	5
CC	GO:00989	intrinsic co	16/1730	77/19717	0.000911	0.005194	0.003866	CDH10/EP	16
CC	GO:00312!	cell projec	48/1730	345/19717	0.000953	0.005374	0.004	CA4/CA9/(	48
CC	GO:00343(	very-low-(	7/1730	20/19717	0.0011	0.00608	0.004526	APOA1/AF	7
CC	GO:00343{	triglyceride	7/1730	20/19717	0.0011	0.00608	0.004526	APOA1/AF	7
CC	GO:00905:	cation-trar	6/1730	15/19717	0.001129	0.006176	0.004597	ATP1A2/A	6
CC	GO:00432(	lysosomal	18/1730	95/19717	0.001397	0.007566	0.005632	APOB/BGN	18
CC	GO:00990!	integral co	15/1730	73/19717	0.001466	0.007857	0.005848	ATP2B4/CI	15
CC	GO:00428:	platelet de	7/1730	21/19717	0.001527	0.008101	0.00603	APOH/ITIH	7

CC	GO:00301	filopodium	19/1730	104/19717	0.001636	0.008596	0.006398	ACTA2/AC		19
CC	GO:00990	integral co	15/1730	74/19717	0.001692	0.008806	0.006555	CDH10/EP		15
CC	GO:00312	leading ed	27/1730	170/19717	0.001823	0.00936	0.006966	AMPH/CSI		27
CC	GO:00986	Schaffer co	16/1730	82/19717	0.001834	0.00936	0.006966	CDH11/DC		16
CC	GO:01500	distal axon	40/1730	285/19717	0.002056	0.010398	0.007739	ADCYAP1/		40
CC	GO:00442	cell body r	8/1730	28/19717	0.002173	0.010887	0.008103	CD22/CX3		8
CC	GO:00310	platelet alp	6/1730	17/19717	0.002396	0.01189	0.008849	CD36/ITGE		6
CC	GO:00991	anchored	5/1730	12/19717	0.00242	0.011902	0.008859	CNTN1/CN		5
CC	GO:00487	presynapti	14/1730	71/19717	0.003114	0.015172	0.011293	ATP2B4/CI		14
CC	GO:00315	filopodium	6/1730	18/19717	0.003329	0.016077	0.011966	DMD/GAP		6
CC	GO:00600	excitatory	11/1730	50/19717	0.003482	0.016662	0.012402	CBLN1/DG		11
CC	GO:00987	presynaps	61/1730	491/19717	0.003569	0.016929	0.0126	ADCYAP1/		61
CC	GO:00988	postsynap	14/1730	74/19717	0.004608	0.021667	0.016127	ACTN2/EP		14
CC	GO:00015	voltage-ga	5/1730	14/19717	0.005273	0.024364	0.018134	SCN1B/SC		5
CC	GO:00310	platelet de	5/1730	14/19717	0.005273	0.024364	0.018134	APOH/ITIH		5
CC	GO:00330	sarcoplasn	9/1730	39/19717	0.005692	0.026076	0.019408	CASQ1/CA		9
CC	GO:00343	high-dens	7/1730	26/19717	0.005856	0.0266	0.019799	APOA1/AF		7
CC	GO:00059	cell-cell ac	19/1730	117/19717	0.006345	0.028578	0.021271	CDH9/CDH		19
CC	GO:00325	dendrite m	9/1730	40/19717	0.006785	0.030304	0.022556	GABRA4/G		9
CC	GO:00328	neuronal c	7/1730	27/19717	0.007318	0.032006	0.023822	CD22/CX3		7
CC	GO:00332	node of Ra	5/1730	15/19717	0.007345	0.032006	0.023822	MYOC/SCI		5
CC	GO:00990	integral co	5/1730	15/19717	0.007345	0.032006	0.023822	ATP2B4/CI		5
CC	GO:00985	ATPase de	6/1730	21/19717	0.007747	0.033488	0.024925	ATP1A2/A		6
CC	GO:00988	actin-base	29/1730	208/19717	0.008576	0.036774	0.027371	ACTA2/AC		29
CC	GO:00432	myelin she	10/1730	49/19717	0.009084	0.038643	0.028762	ERBB2/GN		10
CC	GO:00325	neuron pro	11/1730	57/19717	0.009778	0.041267	0.030716	GABRA4/G		11
CC	GO:00312	lamellipod	6/1730	22/19717	0.009876	0.041355	0.03078	CSPG4/FA		6
CC	GO:00163	catenin co	7/1730	29/19717	0.011024	0.045804	0.034092	CDH9/CDH		7
CC	GO:00600	inhibitory	5/1730	17/19717	0.013059	0.053842	0.040075	NLGN4Y/S		5
CC	GO:00304	growth co	24/1730	171/19717	0.01458	0.059655	0.044402	APBB1/CN		24
CC	GO:00991	intrinsic co	10/1730	53/19717	0.015676	0.063655	0.047379	EPHA7/GR		10
CC	GO:00058	sodium:po	4/1730	12/19717	0.016469	0.065877	0.049032	ATP1A2/A		4
CC	GO:00332	paranode	4/1730	12/19717	0.016469	0.065877	0.049032	KCNA1/CN		4
CC	GO:00443	neuron pro	20/1730	138/19717	0.017626	0.069981	0.052087	ADCYAP1/		20
CC	GO:00355	specific gra	11/1730	62/19717	0.018041	0.071104	0.052923	BPI/ELANE		11
CC	GO:00300	desmosom	6/1730	25/19717	0.018697	0.073152	0.054447	DSC2/DSG		6
CC	GO:00304	site of pola	24/1730	176/19717	0.020134	0.078201	0.058206	APBB1/CN		24
CC	GO:00160	dystrophin	5/1730	19/19717	0.021191	0.080555	0.059957	DMD/PGM		5
CC	GO:00313	anchored	5/1730	19/19717	0.021191	0.080555	0.059957	CA4/FOLR		5
CC	GO:00906	glycoprote	5/1730	19/19717	0.021191	0.080555	0.059957	DMD/PGM		5
CC	GO:00325	dendritic s	4/1730	13/19717	0.022166	0.08367	0.062276	GRIA1/PAL		4
CC	GO:00099	basal plasr	7/1730	34/19717	0.025909	0.097114	0.072282	ERBB2/ITG		7
MF	GO:00052	extracellul	84/1526	163/17697	3.98E-46	3.44E-43	2.82E-43	AEBP1/BGI		84
MF	GO:00055	glycosamir	78/1526	229/17697	1.43E-27	6.19E-25	5.08E-25	ANXA6/AF		78
MF	GO:00082	heparin bi	59/1526	169/17697	9.06E-22	2.61E-19	2.14E-19	APOB/APC		59
MF	GO:00480	receptor li	97/1526	482/17697	1.42E-15	3.06E-13	2.51E-13	ADCYAP1/		97
MF	GO:19016	sulfur com	63/1526	250/17697	3.53E-15	6.09E-13	5.00E-13	ANXA6/AF		63
MF	GO:00300	extracellul	23/1526	41/17697	1.25E-14	1.79E-12	1.47E-12	COL1A1/C		23
MF	GO:00611	peptidase	51/1526	219/17697	3.61E-11	4.45E-09	3.65E-09	A2M/AHS		51
MF	GO:00304	peptidase	45/1526	182/17697	6.17E-11	6.18E-09	5.07E-09	A2M/AHS		45
MF	GO:00051	integrin bi	37/1526	132/17697	6.44E-11	6.18E-09	5.07E-09	ACTN2/CC		37
MF	GO:00055	collagen b	25/1526	67/17697	9.67E-11	8.35E-09	6.85E-09	AEBP1/CO		25
MF	GO:00048	endopepti	43/1526	175/17697	2.03E-10	1.58E-08	1.30E-08	A2M/AHS		43
MF	GO:00611	endopepti	44/1526	182/17697	2.20E-10	1.58E-08	1.30E-08	A2M/AHS		44
MF	GO:00198	growth fac	36/1526	137/17697	8.34E-10	5.53E-08	4.54E-08	A2M/CD3		36
MF	GO:00085	G protein-	36/1526	146/17697	5.38E-09	3.32E-07	2.72E-07	ADCYAP1		36
MF	GO:00048	serine-typ	27/1526	94/17697	1.35E-08	7.79E-07	6.39E-07	A2M/SERP		27
MF	GO:00016	peptide re	36/1526	152/17697	1.68E-08	9.08E-07	7.46E-07	ADCYAP1		36
MF	GO:00199	cytokine bi	32/1526	128/17697	2.63E-08	1.34E-06	1.10E-06	A2M/CXC		32
MF	GO:00051	cytokine a	45/1526	220/17697	3.67E-08	1.76E-06	1.45E-06	BMP3/TNF		45
MF	GO:00300	extracellul	12/1526	22/17697	4.62E-08	2.10E-06	1.72E-06	BGN/HAPL		12
MF	GO:00047	transmem	20/1526	62/17697	1.23E-07	5.30E-06	4.35E-06	ALK/AXL/		20

MF	GO:000382	antigen bi	35/1526	160/17697	2.17E-07	8.92E-06	7.32E-06	CD1C/CD1	35
MF	GO:000166	G protein-	51/1526	280/17697	2.35E-07	9.22E-06	7.57E-06	ADCYAP1/	51
MF	GO:003498	immunogl	22/1526	76/17697	2.52E-07	9.47E-06	7.77E-06	FLNA/IGHI	22
MF	GO:000526	cation cha	55/1526	319/17697	5.12E-07	1.83E-05	1.50E-05	ANXA6/CA	55
MF	GO:001919	transmeml	22/1526	79/17697	5.30E-07	1.83E-05	1.50E-05	ALK/AXL/C	22
MF	GO:005084	extracellula	18/1526	57/17697	7.46E-07	2.48E-05	2.03E-05	BGN/COL1	18
MF	GO:000196	fibronectin	12/1526	27/17697	8.25E-07	2.64E-05	2.16E-05	CCN2/CTS	12
MF	GO:000521	ion channe	66/1526	416/17697	8.84E-07	2.72E-05	2.24E-05	ANXA6/CA	66
MF	GO:000485	enzyme inl	61/1526	375/17697	9.76E-07	2.91E-05	2.39E-05	A2M/AHS	61
MF	GO:001507	potassium	33/1526	159/17697	1.67E-06	4.81E-05	3.95E-05	ATP1A2/A	33
MF	GO:002283	ion gated	55/1526	334/17697	2.25E-06	6.26E-05	5.14E-05	ANXA6/CA	55
MF	GO:002283	substrate-	66/1526	428/17697	2.43E-06	6.55E-05	5.37E-05	ANXA6/CA	66
MF	GO:004687	metal ion t	67/1526	438/17697	2.71E-06	7.09E-05	5.82E-05	ANXA6/AT	67
MF	GO:000517	hormone a	27/1526	122/17697	4.05E-06	0.000103	8.45E-05	ADCYAP1/	27
MF	GO:002283	gated char	55/1526	343/17697	5.12E-06	0.000126	0.000104	ANXA6/CA	55
MF	GO:001526	channel ac	68/1526	456/17697	5.58E-06	0.000134	0.00011	ANXA6/CA	68
MF	GO:002280	passive tra	68/1526	457/17697	6.02E-06	0.00014	0.000115	ANXA6/CA	68
MF	GO:000524	voltage-ga	35/1526	197/17697	3.04E-05	0.000672	0.000552	CACNA1C	35
MF	GO:002283	voltage-ga	35/1526	197/17697	3.04E-05	0.000672	0.000552	CACNA1C	35
MF	GO:000526	potassium	25/1526	123/17697	4.23E-05	0.000913	0.00075	GRIK3/GRI	25
MF	GO:001995	chemokine	11/1526	32/17697	4.44E-05	0.000934	0.000767	A2M/CXCF	11
MF	GO:005083	cell adhesi	69/1526	499/17697	5.87E-05	0.001205	0.00099	ACTN2/CA	69
MF	GO:000552	insulin-like	10/1526	28/17697	6.80E-05	0.001365	0.001121	CCN2/IGFI	10
MF	GO:000510	frizzled bir	12/1526	39/17697	6.99E-05	0.00137	0.001125	MYOC/RO	12
MF	GO:000823	serine-typ	32/1526	182/17697	8.03E-05	0.001539	0.001263	C1R/C1S/C	32
MF	GO:000425	serine-typ	29/1526	160/17697	9.78E-05	0.001834	0.001505	C1R/C1S/C	29
MF	GO:001527	outward re	7/1526	15/17697	0.000121	0.002204	0.00181	KCNA3/KC	7
MF	GO:001717	serine hyd	32/1526	186/17697	0.000123	0.002204	0.00181	C1R/C1S/C	32
MF	GO:000524	voltage-ga	19/1526	87/17697	0.000126	0.002219	0.001822	KCNA1/KC	19
MF	GO:004840	platelet-de	6/1526	11/17697	0.000129	0.002219	0.001822	COL1A1/C	6
MF	GO:000808	growth fac	29/1526	163/17697	0.000137	0.002321	0.001906	BMP3/CCN	29
MF	GO:000549	steroid bin	20/1526	95/17697	0.000145	0.002404	0.001974	ANXA6/AF	20
MF	GO:004339	proteoglyc	11/1526	36/17697	0.00015	0.002438	0.002002	COL5A1/C	11
MF	GO:002284	voltage-ga	26/1526	142/17697	0.000185	0.002959	0.002429	CACNA1C	26
MF	GO:001714	Wnt-prote	11/1526	37/17697	0.000197	0.003085	0.002533	AXL/FRZB/	11
MF	GO:001507	monovaler	54/1526	382/17697	0.000206	0.003178	0.002609	ATP1A2/A	54
MF	GO:000818	neuropept	13/1526	50/17697	0.000237	0.003584	0.002942	GALR1/GR	13
MF	GO:000823	metallope	30/1526	181/17697	0.000382	0.005565	0.004569	AEBP1/CP	30
MF	GO:009863	cell adhesi	14/1526	59/17697	0.000388	0.005565	0.004569	DSC2/ITGA	14
MF	GO:000518	neuropept	9/1526	28/17697	0.000392	0.005565	0.004569	ADCYAP1/	9
MF	GO:000520	structural	20/1526	102/17697	0.000393	0.005565	0.004569	ADD2/ANI	20
MF	GO:000817	adenylate	6/1526	13/17697	0.00041	0.005712	0.004689	ADCY2/AD	6
MF	GO:000422	metalloen	20/1526	103/17697	0.000449	0.006155	0.005052	FAP/MEP1	20
MF	GO:000527	sodium ch	11/1526	41/17697	0.000527	0.007107	0.005834	GRIK3/GRI	11
MF	GO:001995	C-C chem	8/1526	24/17697	0.000628	0.008332	0.00684	CXCR5/CC	8
MF	GO:000495	prostaglan	5/1526	10/17697	0.000825	0.010644	0.008737	PTGDR/PT	5
MF	GO:004317	alcohol bir	17/1526	85/17697	0.000826	0.010644	0.008737	ADH7/ANX	17
MF	GO:000512	cytokine re	41/1526	286/17697	0.000861	0.010922	0.008965	BMP3/TNF	41
MF	GO:001508	sodium ior	25/1526	149/17697	0.00094	0.011762	0.009656	ATP1A2/A	25
MF	GO:000495	icosanoid	6/1526	15/17697	0.001029	0.01251	0.010269	PTGDR/PT	6
MF	GO:007049	oligosacch	6/1526	15/17697	0.001029	0.01251	0.010269	REG3A/SEI	6
MF	GO:003024	carbohydra	39/1526	271/17697	0.001054	0.012638	0.010374	C4B/CD22	39
MF	GO:000163	G protein-	8/1526	26/17697	0.001141	0.013127	0.010776	CXCR5/CC	8
MF	GO:000495	chemokine	8/1526	26/17697	0.001141	0.013127	0.010776	CXCR5/CC	8
MF	GO:007032	lipoprotei	8/1526	26/17697	0.001141	0.013127	0.010776	APOA1/AF	8
MF	GO:004237	chemokine	14/1526	66/17697	0.001277	0.014506	0.011907	DEFB1/DEI	14
MF	GO:000184	compleme	7/1526	21/17697	0.001377	0.014971	0.012289	C4A/C4B/C	7
MF	GO:000495	prostanoid	5/1526	11/17697	0.001405	0.014971	0.012289	PTGDR/PT	5
MF	GO:000539	sodium:po	5/1526	11/17697	0.001405	0.014971	0.012289	ATP1A2/A	5
MF	GO:000855	potassium	5/1526	11/17697	0.001405	0.014971	0.012289	ATP1A2/A	5
MF	GO:009749	structural r	5/1526	11/17697	0.001405	0.014971	0.012289	ELN/FBLN2	5
MF	GO:007181	lipoprotei	9/1526	33/17697	0.001477	0.015326	0.012581	APOA1/AF	9

MF	GO:00718:	protein-lip	9/1526	33/17697	0.001477	0.015326	0.012581	APOA1/AF	9
MF	GO:00083(	structural (	11/1526	46/17697	0.001492	0.015326	0.012581	ACTN2/CS	11
MF	GO:00081!	metalloen(	6/1526	16/17697	0.001527	0.015326	0.012581	COL4A3/N	6
MF	GO:00301(	low-densit	6/1526	16/17697	0.001527	0.015326	0.012581	CD36/CRP	6
MF	GO:00152	ligand-gat	23/1526	138/17697	0.001615	0.015836	0.012999	ANXA6/CH	23
MF	GO:00228:	ligand-gat	23/1526	138/17697	0.001615	0.015836	0.012999	ANXA6/CH	23
MF	GO:00041	endopepti(	55/1526	427/17697	0.001726	0.016734	0.013737	C1R/C1S/(	55
MF	GO:0428:	peptidogly	6/1526	17/17697	0.00219	0.020995	0.017234	REG3A/TR	6
MF	GO:00041{	metallocar	8/1526	29/17697	0.002483	0.02337	0.019184	AEBP1/CP/	8
MF	GO:00164!	C-C chem(	7/1526	23/17697	0.002491	0.02337	0.019184	CXCR5/CC	7
MF	GO:00154{	cholestero	11/1526	49/17697	0.002566	0.02381	0.019545	ANXA6/AF	11
MF	GO:00480:	CCR chem	10/1526	43/17697	0.003016	0.027686	0.022727	DEFB1/DEI	10
MF	GO:00020:	protease b	21/1526	128/17697	0.003059	0.027788	0.02281	A2M/COL:	21
MF	GO:00012:	DNA-bind	55/1526	439/17697	0.003168	0.028479	0.023378	AR/NKX2-	55
MF	GO:00051	vascular er	5/1526	13/17697	0.003381	0.029774	0.024441	VEGFD/IT(	5
MF	GO:0420‹	neurexin fa	5/1526	13/17697	0.003381	0.029774	0.024441	CEL/CPE/N	5
MF	GO:00017{	phosphatic	12/1526	58/17697	0.003448	0.030047	0.024665	ANXA6/AX	12
MF	GO:00513!	alpha-acti	9/1526	37/17697	0.003482	0.030047	0.024665	CACNA1C/	9
MF	GO:0050‹	scavenger	11/1526	51/17697	0.003578	0.030568	0.025093	CD36/TMF	11
MF	GO:00428(	actinin bin	10/1526	46/17697	0.005062	0.042826	0.035155	CACNA1C/	10
MF	GO:00047:	protein tyr	21/1526	134/17697	0.005298	0.044391	0.03644	ALK/AXL/E	21
MF	GO:0052‹	voltage-ga	6/1526	20/17697	0.005478	0.045027	0.036962	PKD2/SCN	6
MF	GO:00083:	signaling p	6/1526	20/17697	0.005478	0.045027	0.036962	CD36/FCN	6
MF	GO:00991(	ion channe	19/1526	118/17697	0.005781	0.047066	0.038636	CAV1/DPP	19
MF	GO:00156(	ATPase act	8/1526	33/17697	0.005898	0.047574	0.039052	ATP1A2/A	8
MF	GO:0162‹	channel re	22/1526	144/17697	0.005965	0.047665	0.039127	ATP2B4/C/	22
MF	GO:00425(	hormone b	17/1526	102/17697	0.006179	0.048924	0.040161	ADCYAP1F	17
MF	GO:0018‹	opsonin bi	5/1526	15/17697	0.00682	0.053022	0.043525	C4A/CR1/(	5
MF	GO:00051(	platelet-de	5/1526	15/17697	0.00682	0.053022	0.043525	VEGFD/IL1	5
MF	GO:0055‹	hyaluronic	6/1526	21/17697	0.007119	0.054067	0.044383	HAPLN1/V	6
MF	GO:00381{	pattern rec	6/1526	21/17697	0.007119	0.054067	0.044383	CD36/FCN	6
MF	GO:00055(	fatty acid b	8/1526	34/17697	0.007142	0.054067	0.044383	FABP4/FAI	8
MF	GO:00055:	calmodulin	28/1526	200/17697	0.007283	0.054653	0.044864	ADD2/AEB	28
MF	GO:00329:	sterol bind	11/1526	56/17697	0.007526	0.055992	0.045963	ANXA6/AF	11
MF	GO:00048!	cytokine re	16/1526	96/17697	0.007755	0.057198	0.046953	CXCR5/CC	16
MF	GO:00080(	chemokine	10/1526	49/17697	0.008062	0.058517	0.048036	CCL7/CCL:	10
MF	GO:00150{	calcium ior	21/1526	139/17697	0.008069	0.058517	0.048036	ANXA6/AT	21
MF	GO:00990!	ligand-gat	17/1526	105/17697	0.00827	0.059473	0.048821	CHRNA3/(	17
MF	GO:00037	actin bindi	52/1526	431/17697	0.00843	0.060121	0.049353	ACTN2/AD	52
MF	GO:00055(	retinoid bi	8/1526	35/17697	0.008574	0.060648	0.049785	ADH7/FAB	8
MF	GO:00504:	transformi	6/1526	22/17697	0.009087	0.063187	0.051869	CD36/LRR(	6
MF	GO:0166‹	oxidoredu(	5/1526	16/17697	0.009225	0.063187	0.051869	LOX/LOXL	5
MF	GO:00302{	structural (	5/1526	16/17697	0.009225	0.063187	0.051869	PI3/PKP1/!	5
MF	GO:00474!	calcium-de	5/1526	16/17697	0.009225	0.063187	0.051869	PLA2G1B/I	5
MF	GO:00986:	cell-cell ac	10/1526	50/17697	0.009318	0.063322	0.05198	DSC2/JUP/	10
MF	GO:00048(	cysteine-ty	11/1526	58/17697	0.009827	0.066257	0.05439	AHSG/AVF	11
MF	GO:0198‹	isoprenoid	8/1526	36/17697	0.010209	0.068299	0.056065	ADH7/FAB	8
MF	GO:00708!	growth fac	20/1526	134/17697	0.010896	0.072332	0.059376	MS4A1/EF	20
MF	GO:00361:	BMP bindi	4/1526	11/17697	0.011089	0.073055	0.05997	COMP/SO	4
MF	GO:00513	muscle alp	6/1526	23/17697	0.011415	0.07463	0.061262	PKD2/PDL	6
MF	GO:00150:	coreceptor	9/1526	44/17697	0.011509	0.074681	0.061304	CCR8/ITGE	9
MF	GO:00198(	immunogl(	6/1526	24/17697	0.014134	0.089836	0.073745	MS4A1/CD	6
MF	GO:01200:	intermemb	6/1526	24/17697	0.014134	0.089836	0.073745	APOA1/AF	6
MF	GO:00420!	chemoattr	8/1526	38/17697	0.014157	0.089836	0.073745	DEFB4A/F(	8
MF	GO:00041:	3',5'-cyclic	4/1526	12/17697	0.015515	0.095179	0.07813	PDE2A/PD	4
MF	GO:00161	superoxide	4/1526	12/17697	0.015515	0.095179	0.07813	NOX1/NO	4
MF	GO:00319!	insulin-like	4/1526	12/17697	0.015515	0.095179	0.07813	IGFBP4/IGI	4
MF	GO:00336!	sialic acid	4/1526	12/17697	0.015515	0.095179	0.07813	CD22/FCN	4
MF	GO:00171:	cholestero	5/1526	18/17697	0.015661	0.095179	0.07813	APOA1/AF	5
MF	GO:01200:	intermemb	5/1526	18/17697	0.015661	0.095179	0.07813	APOA1/AF	5
