# Supplementary material for: Identification of GGT5 as a Novel Prognostic Biomarker for Gastric Cancer and its Correlation With Immune Cell Infiltration
Source: Front Genet. 2022 Mar 18;13:810292. doi: 10.3389/fgene.2022.810292 (PMC8971189; doi:10.3389/fgene.2022.810292)
Supplement: Supplementary file 4 [file DataSheet9.PDF]

ONTOLOGY	ID	Description	GeneRatio	BgRatio	pvalue	p.adjust	qvalue	geneID	Count
BP	GO:0003012	muscle systen	10/35	465/18670	8.7636E-09	1.038E-05	7.7327E-06	ACTG2/CAV1/	10
BP	GO:0006936	muscle contra	9/35	360/18670	1.5131E-08	1.038E-05	7.7327E-06	ACTG2/CAV1/	9
BP	GO:0090257	regulation of ı	6/35	259/18670	7.79E-06	0.00356265	0.00265408	CAV1/CNN1/I	6
BP	GO:0006937	regulation of ı	5/35	171/18670	1.5797E-05	0.00541844	0.0040366	CAV1/CNN1/F	5
BP	GO:0048009	insulin-like gro	3/35	38/18670	4.8674E-05	0.01186383	0.00883826	GHR/IGF1/IGF	3
BP	GO:0006939	smooth muscl	4/35	110/18670	5.1883E-05	0.01186383	0.00883826	CAV1/CNN1/I	4
BP	GO:0033002	muscle cell pr	5/35	239/18670	7.8216E-05	0.01533035	0.01142072	APOD/CNN1/	5
BP	GO:0060537	muscle tissue	6/35	408/18670	9.9601E-05	0.01559663	0.0116191	CAV1/IGF1/M	6
BP	GO:0007517	muscle organ	6/35	410/18670	0.00010231	0.01559663	0.0116191	CAV1/FHL1/I(	6
BP	GO:0050673	epithelial cell	6/35	434/18670	0.00013965	0.0184722	0.01376132	CAV1/IGF1/IG	6
BP	GO:2000242	negative regu	3/35	55/18670	0.0001481	0.0184722	0.01376132	IGF1/SERPINF	3
BP	GO:0043568	positive regula	2/35	13/18670	0.00026287	0.02606983	0.01942136	IGF1/IGFBP4	2
BP	GO:0048662	negative regu	3/35	67/18670	0.0002663	0.02606983	0.01942136	APOD/CNN1/(	3
BP	GO:0048660	regulation of :	4/35	169/18670	0.00027253	0.02606983	0.01942136	APOD/CNN1/	4
BP	GO:0048659	smooth muscl	4/35	171/18670	0.00028502	0.02606983	0.01942136	APOD/CNN1/	4
BP	GO:0048639	positive regula	4/35	184/18670	0.0003765	0.03071708	0.02288344	GHR/IGF1/CX(	4
BP	GO:0006957	complement a	2/35	16/18670	0.00040299	0.03071708	0.02288344	C7/CFD	2
BP	GO:0086103	G protein-cou	2/35	16/18670	0.00040299	0.03071708	0.02288344	CAV1/PLN	2
BP	GO:0042509	regulation of t	3/35	83/18670	0.00050038	0.03613295	0.02691814	CAV1/GHR/IG	3
BP	GO:0007260	tyrosine phos	3/35	86/18670	0.0005552	0.03808699	0.02837384	CAV1/GHR/IG	3
BP	GO:0050678	regulation of (	5/35	378/18670	0.00065235	0.04262026	0.03175101	CAV1/IGF1/SE	5
BP	GO:0014706	striated muscl	5/35	390/18670	0.00075111	0.04684206	0.03489615	CAV1/IGF1/M	5
BP	GO:0010959	regulation of ı	5/35	394/18670	0.0007864	0.04691055	0.03494717	CAV1/FHL1/M	5
BP	GO:0048738	cardiac muscl	4/35	233/18670	0.00091351	0.05222236	0.03890434	IGF1/MYH11/	4
BP	GO:0043567	regulation of i	2/35	26/18670	0.00107867	0.05919753	0.04410066	IGF1/IGFBP4	2
BP	GO:0002026	regulation of t	2/35	27/18670	0.0011636	0.06123284	0.04561692	CAV1/PLN	2
BP	GO:0051924	regulation of (	4/35	254/18670	0.0012574	0.06123284	0.04561692	CAV1/MYLK/F	4
BP	GO:0030282	bone minerali	3/35	114/18670	0.00125858	0.06123284	0.04561692	IGF1/MGP/AS	3
BP	GO:0050730	regulation of		4/35	256/18670	0.00129428	0.06123284	0.04561692	CAV1/GHR/IG	4
BP	GO:0051928	positive regula	3/35	123/18670	0.00156577	0.06966174	0.05189623	CAV1/MYLK/(	3
BP	GO:0045927	positive regula	4/35	270/18670	0.00157399	0.06966174	0.05189623	GHR/IGF1/CX(	4
BP	GO:0043270	positive regula	4/35	275/18670	0.00168337	0.07217433	0.05376805	CAV1/FHL1/M	4
BP	GO:0050727	regulation of i	5/35	485/18670	0.00197648	0.08217361	0.06121726	APOD/C7/FAE	5
BP	GO:0046425	regulation of .	3/35	137/18670	0.00213056	0.08597421	0.06404861	CAV1/GHR/IG	3
BP	GO:1904892	regulation of :	3/35	146/18670	0.00255262	0.09728321	0.07247353	CAV1/GHR/IG	3
BP	GO:2000241	regulation of ı	3/35	146/18670	0.00255262	0.09728321	0.07247353	IGF1/SERPINF	3
BP	GO:0090279	regulation of (	2/35	41/18670	0.00267402	0.09915542	0.07386828	PLN/CXCL12	2
CC	GO:0062023	collagen-conta	8/35	406/19717	4.3634E-07	4.7561E-05	3.4907E-05	MGP/OGN/SE	8
CC	GO:0016459	myosin compl	3/35	65/19717	0.00020755	0.01131162	0.00830211	ACTG2/MYH1	3
CC	GO:0005859	muscle myosi	2/35	18/19717	0.00046007	0.01507534	0.01106447	MYH11/MYL9	2
CC	GO:0016460	myosin II com	2/35	22/19717	0.00069153	0.01507534	0.01106447	MYH11/MYL9	2
CC	GO:0032982	myosin filame	2/35	22/19717	0.00069153	0.01507534	0.01106447	ACTG2/MYH1	2
CC	GO:0005925	focal adhesior	4/35	405/19717	0.00555249	0.05500892	0.04037352	CAV1/CNN1/F	4
CC	GO:0005924	cell-substrate	4/35	408/19717	0.00569804	0.05500892	0.04037352	CAV1/CNN1/F	4
CC	GO:0030055	cell-substrate	4/35	412/19717	0.00589606	0.05500892	0.04037352	CAV1/CNN1/F	4
CC	GO:0001725	stress fiber	2/35	67/19717	0.00629595	0.05500892	0.04037352	MYLK/MYL9	2
CC	GO:0031093	platelet alpha	2/35	67/19717	0.00629595	0.05500892	0.04037352	CFD/IGF1	2
CC	GO:0097517	contractile act	2/35	67/19717	0.00629595	0.05500892	0.04037352	MYLK/MYL9	2
CC	GO:0044449	contractile fib	3/35	221/19717	0.0069811	0.05500892	0.04037352	MYH11/MYL9	3
CC	GO:0016529	sarcoplasmic ı	2/35	71/19717	0.00704486	0.05500892	0.04037352	PLN/THBS4	2
CC	GO:0032432	actin filament	2/35	75/19717	0.0078322	0.05500892	0.04037352	MYLK/MYL9	2
CC	GO:0043292	contractile fib	3/35	234/19717	0.00816425	0.05500892	0.04037352	MYH11/MYL9	3
CC	GO:0042641	actomyosin	2/35	79/19717	0.00865741	0.05500892	0.04037352	MYLK/MYL9	2
CC	GO:0016528	sarcoplasm	2/35	80/19717	0.00886956	0.05500892	0.04037352	PLN/THBS4	2
CC	GO:0005811	lipid droplet	2/35	81/19717	0.00908404	0.05500892	0.04037352	CAV1/FABP4	2
CC	GO:0031091	platelet alpha	2/35	91/19717	0.01135484	0.06514094	0.04780986	CFD/IGF1	2
CC	GO:0005604	basement me	2/35	95/19717	0.01232615	0.0671775	0.04930459	SERPINF1/THI	2
CC	GO:0042470	melanosome	2/35	106/19717	0.0151772	0.07519612	0.05518981	MYH11/SERPI	2
CC	GO:0048770	pigment granu	2/35	106/19717	0.0151772	0.07519612	0.05518981	MYH11/SERPI	2
MF	GO:0008307	structural con	3/34	46/17697	9.2952E-05	0.00933559	0.00636931	MYH11/MYL9	3
MF	GO:0005201	extracellular r	4/34	163/17697	0.00025932	0.00933559	0.00636931	MGP/OGN/SF	4
MF	GO:0008083	growth factor	4/34	163/17697	0.00025932	0.00933559	0.00636931	IGF1/OGN/CX	4
MF	GO:0030021	extracellular r	2/34	22/17697	0.00080791	0.02181356	0.01488255	OGN/ASPN	2
MF	GO:0044325	ion channel bi	3/34	124/17697	0.00171451	0.03162462	0.02157625	CAV1/FHL1/B.	3
MF	GO:0005319	lipid transport	3/34	131/17697	0.00200578	0.03162462	0.02157625	APOD/FABP4/	3
MF	GO:0005178	integrin bindi	3/34	132/17697	0.00204974	0.03162462	0.02157625	IGF1/CXCL12/	3
MF	GO:0060590	ATPase regula	2/34	40/17697	0.00266959	0.03603946	0.02458833	PLN/BAG2	2
MF	GO:0015485	cholesterol bi	2/34	49/17697	0.00398166	0.04777988	0.03259836	APOD/CAV1	2

MF	GO:0032934	sterol binding	2/34	56/17697	0.00517047	0.05584108	0.0380982	APOD/CAV1	2
MF	GO:0005516	calmodulin bi	3/34	200/17697	0.00657535	0.06455795	0.04404539	CNN1/MYH11	3
MF	GO:0005518	collagen bindi	2/34	67/17697	0.0073261	0.06593487	0.0449848	SPARCL1/ASP	2
MF	GO:0003779	actin binding	4/34	431/17697	0.00902993	0.07501789	0.0511818	CNN1/MYH11	4
MF	GO:0051117	ATPase bindir	2/34	80/17697	0.01030893	0.07952607	0.05425755	CAV1/PLN	2
MF	GO:0043178	alcohol bindin	2/34	85/17697	0.01157707	0.0833549	0.05686982	APOD/CAV1	2
MF	GO:0048018	receptor ligan	4/34	482/17697	0.01320552	0.08913725	0.06081489	IGF1/OGN/CX	4
MF	GO:0005496	steroid bindin	2/34	95/17697	0.0143075	0.09089473	0.06201395	APOD/CAV1	2
MF	GO:0051087	chaperone bir	2/34	102/17697	0.01636839	0.09821032	0.06700509	BAG2/HSPB6	2
MF	GO:0032395	MHC class II r	1/34	10/17697	0.01905185	0.09858116	0.0672581	HLA-DQA1	1
MF	GO:0005324	long-chain fat	1/34	11/17697	0.02093754	0.09858116	0.0672581	FABP4	1
MF	GO:0017166	vinculin bindir	1/34	11/17697	0.02093754	0.09858116	0.0672581	SYNM	1
MF	GO:0045236	CXCR chemok	1/34	11/17697	0.02093754	0.09858116	0.0672581	CXCL12	1
MF	GO:0099106	ion channel re	2/34	118/17697	0.0215214	0.09858116	0.0672581	CAV1/PLN	2
MF	GO:0031994	insulin-like gr	1/34	12/17697	0.02281971	0.09858116	0.0672581	IGFBP4	1
MF	GO:0032036	myosin heavy	1/34	12/17697	0.02281971	0.09858116	0.0672581	MYL9	1
MF	GO:0008179	adenylate cyc	1/34	13/17697	0.02469837	0.09896349	0.06751895	AKAP12	1
MF	GO:0019955	cytokine bindi	2/34	128/17697	0.0250408	0.09896349	0.06751895	ACKR1/GHR	2
MF	GO:0036041	long-chain fat	1/34	14/17697	0.02657353	0.09896349	0.06751895	FABP4	1
MF	GO:0050998	nitric-oxide sy	1/34	14/17697	0.02657353	0.09896349	0.06751895	CAV1	1
MF	GO:0019838	growth factor	2/34	137/17697	0.0283954	0.09909936	0.06761165	GHR/IGFBP4	2
MF	GO:0019215	intermediate	1/34	15/17697	0.02844519	0.09909936	0.06761165	SYNM	1

ONTOLOGY	ID	Descriptior	GeneRatio	BgRatio	pvalue	p.adjust	qvalue	geneID	Count
KEGG	hsa04510	Focal adhe:	5/22	201/8076	0.000169	0.016941	0.015515	CAV1/IGF1	5
KEGG	hsa04270	Vascular sn	4/22	135/8076	0.000432	0.021619	0.019798	ACTG2/MY	4
KEGG	hsa05150	Staphyloco	3/22	96/8076	0.002128	0.064433	0.059007	CFD/HLA-D	3
KEGG	hsa04810	Regulation	4/22	218/8076	0.002577	0.064433	0.059007	MYH11/MY	4
