# Supplementary figures and images for: Identification of GGT5 as a Novel Prognostic Biomarker for Gastric Cancer and its Correlation With Immune Cell Infiltration
Source: Front Genet. 2022 Mar 18;13:810292. doi: 10.3389/fgene.2022.810292 (PMC8971189; doi:10.3389/fgene.2022.810292)

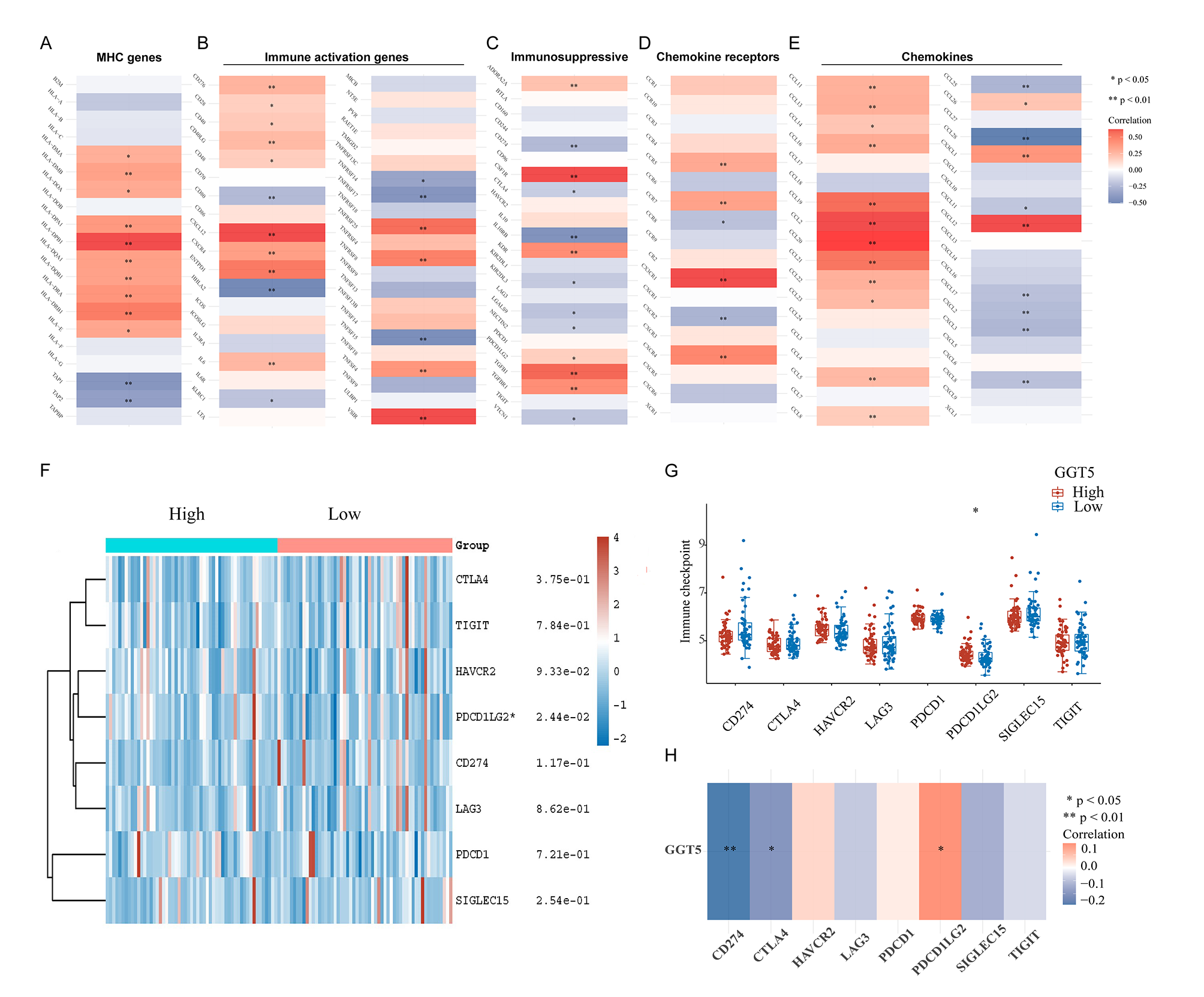

Supplement: Supplementary file 5 [file Image3.TIF]

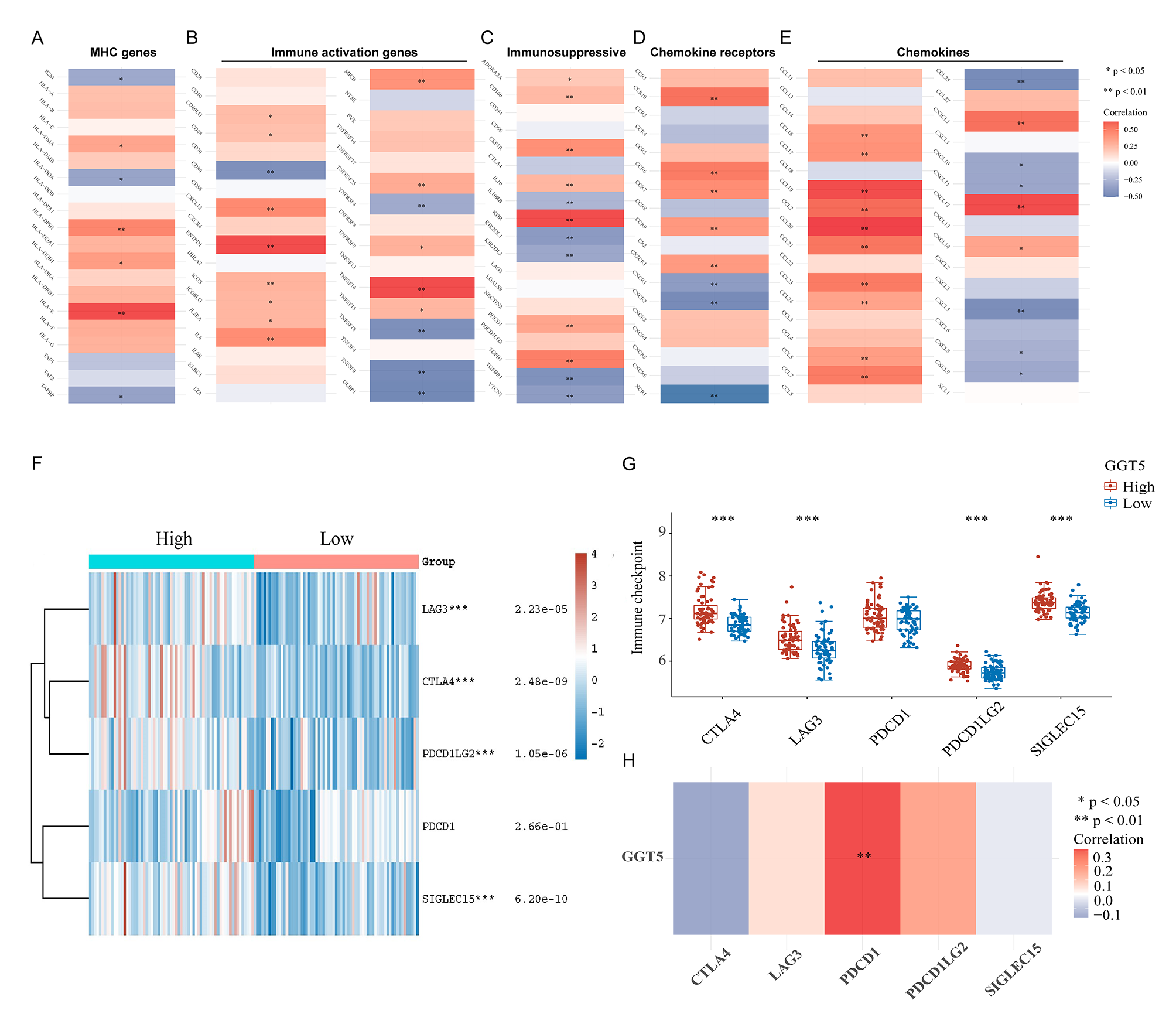

Supplement: Supplementary file 6 [file Image4.TIF]

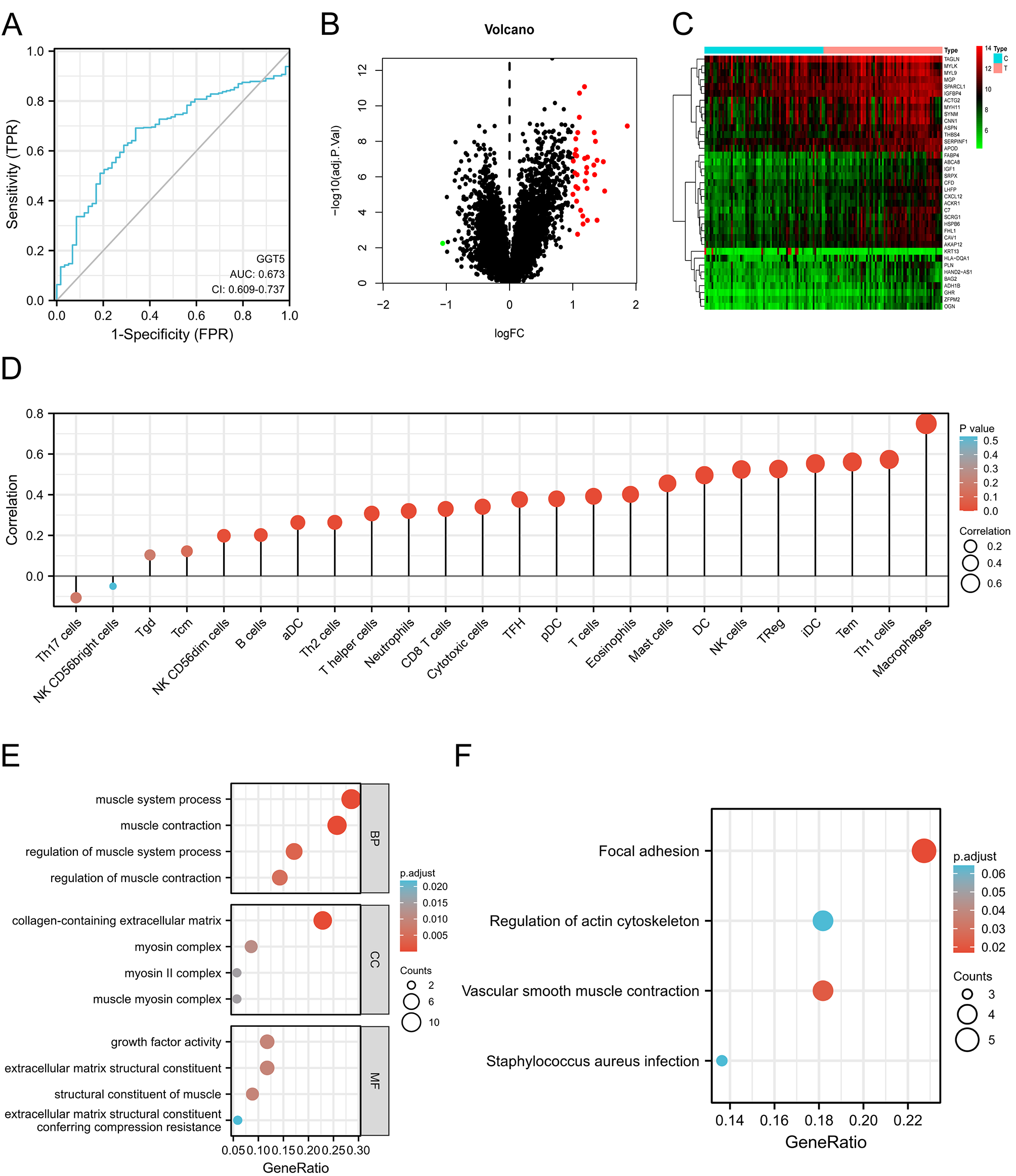

Supplement: Supplementary file 7 [file Image2.TIF]

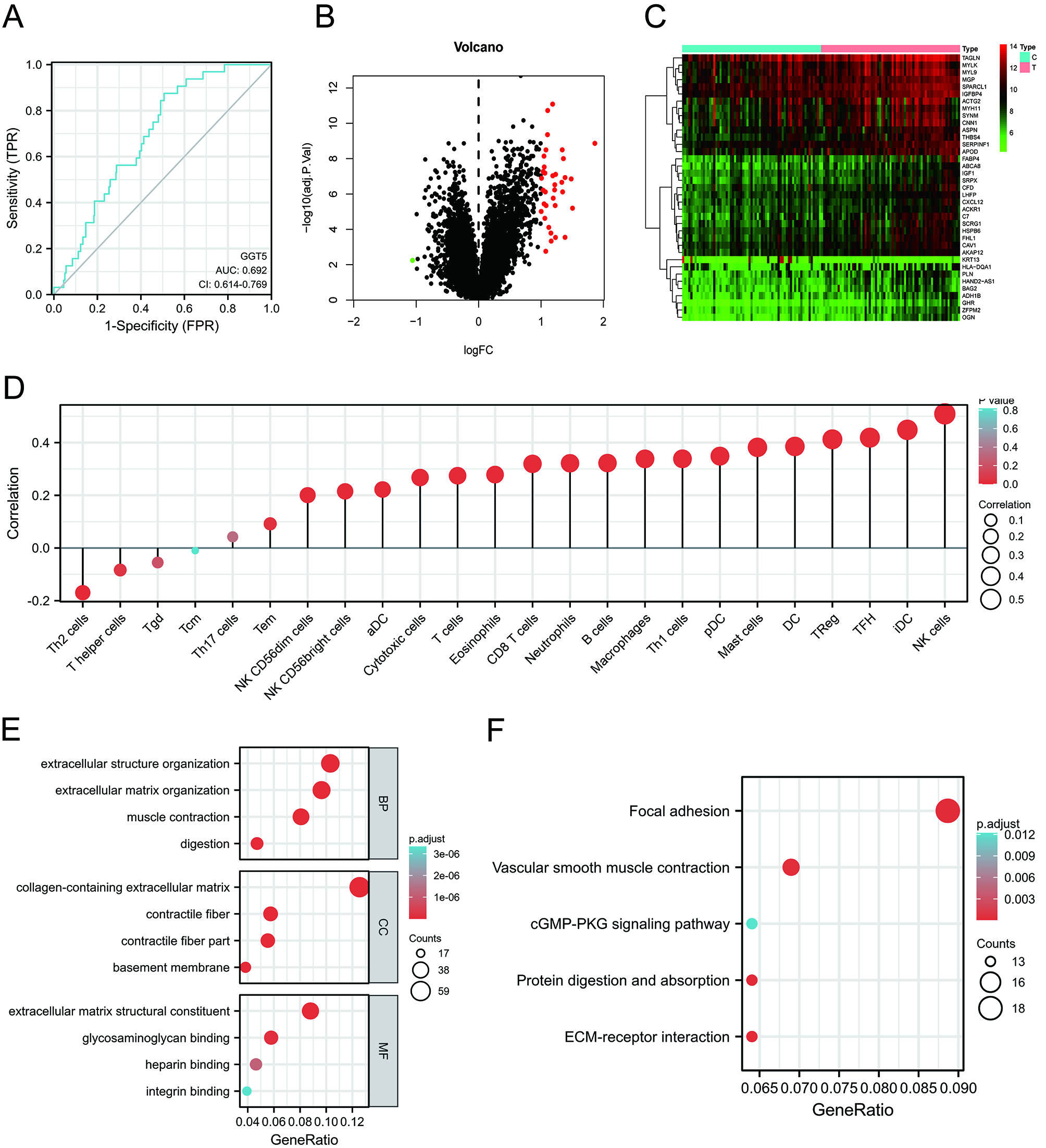

Supplement: Supplementary file 8 [file Image1.TIF]
