# Supplementary material for: Identification of GGT5 as a Novel Prognostic Biomarker for Gastric Cancer and its Correlation With Immune Cell Infiltration
Source: Front Genet. 2022 Mar 18;13:810292. doi: 10.3389/fgene.2022.810292 (PMC8971189; doi:10.3389/fgene.2022.810292)
Supplement: Supplementary file 9 [file DataSheet3.PDF]

Gene ID	log2FoldC	padj
HSPB6	3.221353	1.25E-49
TAC1	2.772858	2.85E-18
CLCA4	-3.1966	8.69E-27
IGF1	2.447032	9.06E-49
ATP1A2	3.097978	5.83E-46
NRXN3	2.200437	6.50E-32
FHL1	2.279499	4.60E-39
MYOC	2.681321	8.80E-19
EPHA3	2.001722	6.53E-51
MAGEC2	-2.02545	0.000564
METTL24	2.102524	3.69E-30
CHRDL2	2.371003	9.52E-34
PSD	2.305871	4.85E-45
CCN5	2.019397	6.26E-28
NGFR	2.625363	5.78E-37
GLP2R	2.088955	3.79E-36
MYLK	2.642098	3.18E-60
PYGM	2.318704	4.48E-51
PAGE1	-2.1018	3.50E-10
ABCC9	2.080793	1.60E-56
FGF10	2.275975	1.23E-33
WSCD2	2.037699	3.88E-19
TACR2	3.032566	1.56E-41
PPP1R12B	2.019524	1.54E-34
DCX	2.548185	9.82E-27
ACTN2	2.65331	6.68E-31
TNS1	2.062435	4.27E-49
CHRNA3	2.389477	3.36E-28
AFP	-2.18635	1.16E-11
PKP1	-2.11473	1.23E-15
TMPRSS1[illegible]	-2.48431	5.57E-12
GNAO1	2.212044	7.58E-37
TMEM40	-2.18301	1.52E-19
APOH	-2.13061	2.03E-15
CCDC80	2.372225	1.25E-64
CMA1	2.191651	8.05E-20
CRTAC1	2.05267	5.85E-24
ASB2	2.02606	6.27E-33
MYL9	2.400126	2.14E-53
CHRDL1	2.643507	4.21E-35
KLHL4	2.022793	8.31E-44
HTR2A	2.008699	7.67E-46
MLNR	2.406522	4.72E-23
SFRP1	2.708767	2.84E-39
FCER2	2.072921	2.06E-18
HAS1	2.043749	2.07E-26
UPK1A	-2.37018	1.44E-24
VIPR2	2.288848	1.90E-40
SFRP4	2.251203	1.93E-35
MEOX2	2.50191	2.53E-51
PRUNE2	2.230013	4.97E-27
OGN	3.000433	6.03E-46
SGCA	2.200407	1.91E-36
CWH43	-2.38627	2.72E-09
NKX3-2	2.244882	2.30E-29
NRXN2	2.181168	3.19E-45
KCNA1	3.241184	4.26E-28

MGP	2.297905	6.47E-59
C7	3.298909	1.53E-54
THBS4	3.551044	4.04E-69
BCHE	2.061629	2.41E-23
GCG	2.649926	5.96E-10
SMYD1	3.723945	1.50E-31
SLC5A7	2.174052	3.42E-17
ANGPTL1	2.982745	1.54E-57
PRG4	2.231786	4.44E-40
PTCH2	2.289311	2.41E-67
TTR	-2.36183	8.20E-16
CASQ2	3.078214	1.30E-46
ECRG4	2.522569	1.42E-23
TNN	2.184798	2.81E-35
POPDC2	2.307743	5.30E-50
CPXM2	2.23851	1.77E-46
LDB3	2.887972	5.77E-49
PLP1	2.141761	1.08E-18
NRK	2.440816	5.91E-36
PTGIS	2.471158	2.39E-52
TGM3	-2.30623	1.33E-23
TMEM35A	2.323765	1.21E-32
OMD	2.545298	7.30E-48
FLNC	3.089714	3.18E-52
CDO1	2.365682	2.78E-56
KCNA5	2.045933	1.52E-37
CNN1	3.290167	1.29E-49
DPP6	2.557848	3.50E-27
AOC3	2.147843	5.57E-54
BARX1	2.043301	9.43E-29
CRP	-2.52165	4.26E-15
MYH11	3.33369	4.59E-48
CHIA	-2.40484	1.55E-08
ADAMTS8	2.113576	6.04E-30
EPHA7	2.079052	1.98E-20
SCN7A	2.387576	2.89E-28
IL36A	-3.68979	1.60E-16
IL36RN	-2.27627	1.33E-12
CLCA2	-2.91397	3.37E-23
CYP1B1	2.470675	3.42E-58
CILP	2.758557	2.90E-45
PCDH10	3.012095	3.09E-39
SHISAL1	2.07846	3.63E-29
KERA	2.592132	9.22E-38
RHCG	-2.7738	2.08E-21
MYOCD	2.220298	1.73E-32
ZNF750	-2.52321	4.32E-30
CASQ1	2.23047	9.31E-31
CRNN	-5.94036	1.26E-22
S100A7	-4.50978	3.77E-33
REG3G	-2.92818	1.13E-14
COL8A1	2.194852	3.10E-78
BOC	2.397967	1.52E-66
SLIT2	2.348008	3.75E-59
SFRP2	2.551291	7.76E-38
KCNMB1	2.028793	7.53E-31
VIP	3.233553	7.18E-38
TAGLN	2.219183	3.79E-42

JPH2	2.362486	1.52E-35
CNKSR2	2.547039	1.08E-38
TMPRSS11	-2.37685	4.07E-15
ABI3BP	2.047893	1.22E-37
ABCA9	2.083225	1.14E-50
PGM5	2.773727	8.17E-48
KCNMA1	2.502016	7.80E-39
CLDN17	-2.76517	7.88E-05
GDF6	2.158654	1.49E-31
KCNB1	2.224273	1.19E-22
CPA2	-2.87989	2.84E-20
PAGE5	-2.1432	1.02E-09
APOA2	-2.068	6.47E-09
SPRR2G	-2.45757	8.41E-09
SCGB3A1	-2.24359	1.82E-16
SYNC	2.377979	3.96E-58
CADM3	2.375361	2.37E-31
ACTG2	3.08078	5.00E-38
C1QTNF7	2.013359	6.07E-35
LCE3D	-2.0648	0.000209
IVL	-3.5472	2.30E-14
SPRR3	-2.6437	1.23E-10
SPRR2D	-2.86974	4.85E-14
TAFA4	2.479734	4.68E-15
IGFN1	2.295442	1.46E-21
LMOD1	2.768514	2.64E-49
TCF23	2.226323	2.88E-24
SCRG1	2.552657	1.93E-30
HAND2	3.33949	2.84E-48
ASB5	2.834283	1.59E-18
CARTPT	3.678359	1.97E-28
PI16	2.376475	3.10E-26
FNDC1	2.249875	3.23E-48
SVEP1	2.13291	3.36E-66
FAT3	2.449342	1.74E-37
PDZRN4	3.017139	3.23E-48
LMO1	3.261899	1.82E-31
PLIN1	2.174693	1.85E-28
GREM1	2.000451	1.12E-41
RBFOX3	2.340906	5.87E-23
PLIN4	3.179703	6.37E-51
KRT24	-2.39409	5.17E-10
TNXB	2.118101	2.34E-43
CTRB1	-2.5928	1.93E-14
VCX3A	-2.75964	3.82E-09
RSPO1	2.387213	5.17E-41
HSPB3	2.2404	3.95E-12
CRCT1	-2.71759	1.49E-10
GKN1	-2.54301	8.19E-10
RNF150	2.077832	1.86E-39
FABP4	3.130543	3.06E-49
KRT78	-2.96586	1.30E-20
KRT6C	-4.94493	8.58E-39
KRT4	-4.22466	7.74E-27
CEL	-2.10987	3.15E-14
PLA2G1B	-2.37318	5.78E-22
KCNK3	2.080671	2.21E-27
KRT13	-4.16745	3.54E-28

FGG	-3.13957	2.86E-16
FGA	-2.71755	2.52E-17
FGB	-2.06493	2.88E-09
ANGPTL7	2.199042	3.21E-33
REG3A	-2.01018	4.47E-08
SYNPO2	3.185689	6.32E-50
CCL19	2.257518	2.36E-26
MRGPRF	2.257868	2.40E-47
BNC2	2.063965	1.59E-60
HSPB7	2.681723	2.47E-36
DIRC1	2.125817	2.28E-20
PODN	2.222955	6.57E-77
MIR1-1HC	2.694267	5.96E-35
MIR1-1HC	2.125602	1.44E-11
DES	3.199057	1.08E-32
WFDC5	-2.01125	8.57E-13
PRIMA1	2.195907	4.46E-24
VCX2	-2.45768	0.000614
SHISA3	2.037712	1.03E-22
CALML3	-3.21146	9.29E-23
CALML5	-2.85948	6.34E-13
DYNAP	-2.65123	4.92E-10
FOXE1	-2.00298	2.58E-11
RTL3	2.580337	3.47E-36
SSC5D	2.057531	2.24E-51
GAS1	2.256142	9.52E-55
AGTR2	3.007778	1.20E-15
CHRM2	2.907568	2.55E-26
ADIPOQ	4.357219	5.94E-36
PENK	2.875538	1.58E-30
SAGE1	-2.27757	1.60E-08
MAB21L2	2.137986	2.52E-22
TMEM252	2.818202	2.67E-37
SLC2A4	2.116373	1.35E-33
RGMA	2.24874	9.89E-34
SYNM	3.088124	1.24E-42
NKX2-5	2.12591	9.60E-15
CTNNA3	2.046046	1.03E-17
GBP6	-2.07291	4.59E-18
GKN2	-2.45465	5.37E-10
S100A7A	-3.128	3.36E-15
TRARG1	3.403698	5.82E-37
FLRT2	2.172275	1.31E-67
NOTUM	-3.09812	2.54E-31
MORN5	2.886716	1.73E-24
TMPRSS11	-4.02916	4.36E-17
LCE3E	-2.85596	9.61E-07
KRT5	-3.82431	3.36E-32
KRT14	-3.53641	1.14E-26
TMPRSS11	-3.22371	5.41E-17
SLC18A3	2.756662	7.72E-19
LIN28B	-2.45546	1.25E-07
COL14A1	2.479929	7.23E-84
C10orf99	-2.11138	3.30E-07
KRTDAP	-3.06524	1.66E-21
ADRB3	2.139044	4.96E-20
PRELP	2.405835	2.25E-44
SBSN	-2.96575	1.49E-21

CGB5	2.14747	1.06E-09
KIAA0408	2.137717	1.67E-19
ADH1B	2.435462	2.52E-28
FAM180B	2.050247	3.19E-21
SPRR2B	-4.77819	1.73E-09
DACT3	2.094318	1.04E-44
FABP12	-2.57021	1.08E-11
SERPINB13	-3.11919	6.34E-14
TMPRSS11	-2.67665	3.81E-11
PLN	2.298633	6.21E-31
ITGBL1	2.339763	2.37E-51
SPRR2E	-4.97935	1.11E-19
RHOXF2B	-2.01693	0.001681
NHSL2	2.010133	2.57E-56
MUC21	-4.44021	1.07E-32
KRT6A	-2.45265	2.75E-12
AC016813	2.178517	5.41E-17
MT-TI	-2.26002	6.21E-11
MT-TR	-2.22795	2.34E-09
KRTAP3-1	-2.25243	7.71E-07
SP9	-3.08824	3.94E-12
RN7SKP26	2.060913	3.24E-16
AC005165	2.293541	1.65E-27
AL513217.	2.569563	1.09E-25
MTND1P2	-2.04811	1.58E-11
AC010907	2.021035	8.79E-10
PGM5-AS	2.972357	9.17E-32
KRT16P6	-2.02978	9.07E-10
AC093787	2.579702	6.06E-13
SPRR2C	-4.29203	1.36E-10
AC016710	-2.0438	0.021484
AC233976	2.035523	3.46E-16
PGA4	-2.22617	0.003013
AC006007	2.905244	1.39E-09
MTND1P4	-2.10033	1.19E-07
MIR205HG	-2.4582	3.18E-08
TRBV26OR	2.36084	4.09E-14
AC007099	-2.05636	5.91E-11
LINC0288	2.585625	3.02E-46
LINC0070	2.514975	7.07E-40
AL356867.	-2.95345	1.19E-06
AC053503	2.726381	2.52E-20
LINC0128	-2.06501	9.90E-13
LINC0188	-2.28394	0.000261
BNIP3P2	-2.18817	0.002284
NPAP1P4	2.344022	7.18E-10
HAND2-A	3.495499	2.02E-52
AC092691	2.066922	2.16E-11
INMT	2.131208	2.68E-65
SPRR2A	-2.82598	3.67E-10
AC121764	-2.0166	0.002866
LINC0121	-2.34427	5.27E-12
SPRR2F	-3.14745	6.44E-15
LINC0211	-2.02586	1.45E-06
AC008808	2.556203	1.69E-21
CARMN	2.675432	3.42E-59
AC026780	2.217617	6.10E-12
TMPRSS11	-2.02116	1.47E-06

BX510359	-3.24619	1.03E-05
FTLP10	-2.29113	2.47E-08
AC012055	2.435298	4.39E-10
LINC0210(	2.249216	1.13E-27
AC022559	-2.23453	7.71E-12
C8orf88	2.247579	5.17E-36
AC044893	-2.04913	5.50E-06
AP001107	2.076773	2.69E-38
AP005018	2.17891	1.10E-27
INMT-MIN	2.504876	1.36E-29
LINC0148(	2.133926	2.13E-16
AC068875	-2.18808	4.29E-05
AC055874	2.444088	8.83E-10
FRRS1L	2.617738	2.58E-30
SERTM2	2.098953	9.70E-26
RARRES2F	-2.08008	0.000142
MUC22	-2.41109	1.52E-13
AP003071	2.227926	1.04E-34
AP003071	2.205407	2.09E-38
MTATP6P:	-2.22559	1.94E-14
AF001548	2.79416	5.43E-41
LINC0256:	-2.28894	4.29E-06
MTND2P1	-2.31539	1.45E-19
MTND1P1	-2.12623	1.94E-19
AC090283	-2.10971	0.002839
MIR4728	-2.29455	7.09E-21
AP000894	2.150422	1.04E-21
GDF10	2.563629	2.88E-36
AC079466	-2.15143	5.70E-11
AC002398	2.508656	2.56E-22
AC005180	2.189369	3.28E-26
AC008878	2.136903	3.43E-15
CT45A1	-2.47199	5.38E-05
AC009102	2.421355	6.18E-28
AC026336	-2.08239	1.53E-08
PGM5P3-/	2.250389	2.34E-32
AC078925	2.019212	4.39E-11
C8orf87	2.128055	3.75E-09
AP000892	2.090533	2.55E-37
AC053503	3.157058	6.62E-28
AF001548	2.715781	2.26E-31
