# Supplementary material for: Identification of GGT5 as a Novel Prognostic Biomarker for Gastric Cancer and its Correlation With Immune Cell Infiltration
Source: Front Genet. 2022 Mar 18;13:810292. doi: 10.3389/fgene.2022.810292 (PMC8971189; doi:10.3389/fgene.2022.810292)
Supplement: Supplementary file 10 [file DataSheet1.PDF]

sample_id	gene	status	expr
TCGA-BR-645	GGT5	Normal	2.54744888
TCGA-BR-645	GGT5	Normal	3.31362876
TCGA-BR-645	GGT5	Normal	3.47997413
TCGA-BR-680	GGT5	Normal	1.50284521
TCGA-BR-770	GGT5	Normal	3.58926743
TCGA-BR-770	GGT5	Normal	4.76246223
TCGA-BR-771	GGT5	Normal	2.9210454
TCGA-BR-771	GGT5	Normal	3.55177692
TCGA-BR-771	GGT5	Normal	2.91081479
TCGA-BR-785	GGT5	Normal	2.52068178
TCGA-BR-806	GGT5	Normal	3.05449532
TCGA-CG-572	GGT5	Normal	3.52250119
TCGA-CG-572	GGT5	Normal	2.13117133
TCGA-CG-572	GGT5	Normal	2.87288939
TCGA-CG-572	GGT5	Normal	2.83643318
TCGA-CG-573	GGT5	Normal	3.73856736
TCGA-CG-573	GGT5	Normal	3.27575105
TCGA-CG-573	GGT5	Normal	3.02876624
TCGA-FP-773	GGT5	Normal	4.22600922
TCGA-FP-782	GGT5	Normal	3.36071305
TCGA-HU-823	GGT5	Normal	2.13129289
TCGA-HU-A4	GGT5	Normal	3.13689177
TCGA-HU-A4	GGT5	Normal	3.79753189
TCGA-HU-A4	GGT5	Normal	2.41972527
TCGA-HU-A4	GGT5	Normal	3.92723235
TCGA-HU-A4	GGT5	Normal	4.9319384
TCGA-HU-A4	GGT5	Normal	3.05375495
TCGA-IN-780	GGT5	Normal	2.69571017
TCGA-IN-866	GGT5	Normal	0.85142052
TCGA-IN-AB1	GGT5	Normal	2.22065933
TCGA-IN-AB1	GGT5	Normal	2.60138709
TCGA-IP-796	GGT5	Normal	2.6916622
TCGA-3M-AB	GGT5	Tumor	2.49979194
TCGA-3M-AB	GGT5	Tumor	4.98523954
TCGA-B7-581	GGT5	Tumor	3.63958785
TCGA-B7-A5T	GGT5	Tumor	5.18052493
TCGA-B7-A5T	GGT5	Tumor	3.39435562
TCGA-B7-A5T	GGT5	Tumor	4.74733095
TCGA-B7-A5T	GGT5	Tumor	5.15380823
TCGA-BR-418	GGT5	Tumor	5.66025555
TCGA-BR-419	GGT5	Tumor	3.77334035
TCGA-BR-420	GGT5	Tumor	4.57306699
TCGA-BR-425	GGT5	Tumor	2.78196128
TCGA-BR-425	GGT5	Tumor	5.31502522
TCGA-BR-425	GGT5	Tumor	4.04608514
TCGA-BR-426	GGT5	Tumor	3.00710906
TCGA-BR-427	GGT5	Tumor	4.76065455
TCGA-BR-428	GGT5	Tumor	2.25980915
TCGA-BR-429	GGT5	Tumor	1.41209201

TCGA-BR-435	GGT5	Tumor	4.35194944
TCGA-BR-436	GGT5	Tumor	4.31132869
TCGA-BR-436	GGT5	Tumor	4.25758542
TCGA-BR-436	GGT5	Tumor	2.96092112
TCGA-BR-436	GGT5	Tumor	4.87284813
TCGA-BR-436	GGT5	Tumor	3.84566254
TCGA-BR-436	GGT5	Tumor	3.5601182
TCGA-BR-437	GGT5	Tumor	4.53952921
TCGA-BR-437	GGT5	Tumor	1.83032445
TCGA-BR-645	GGT5	Tumor	4.18854044
TCGA-BR-645	GGT5	Tumor	5.49448359
TCGA-BR-645	GGT5	Tumor	4.36124611
TCGA-BR-645	GGT5	Tumor	4.66411039
TCGA-BR-645	GGT5	Tumor	5.79798911
TCGA-BR-645	GGT5	Tumor	6.71642256
TCGA-BR-645	GGT5	Tumor	5.69518466
TCGA-BR-656	GGT5	Tumor	5.41863389
TCGA-BR-656	GGT5	Tumor	5.75532807
TCGA-BR-656	GGT5	Tumor	4.74392595
TCGA-BR-656	GGT5	Tumor	4.84992449
TCGA-BR-670	GGT5	Tumor	6.53240076
TCGA-BR-670	GGT5	Tumor	4.05833228
TCGA-BR-670	GGT5	Tumor	8.07754401
TCGA-BR-671	GGT5	Tumor	3.29403491
TCGA-BR-680	GGT5	Tumor	3.57308962
TCGA-BR-680	GGT5	Tumor	4.21633458
TCGA-BR-680	GGT5	Tumor	5.37539996
TCGA-BR-685	GGT5	Tumor	4.98330362
TCGA-BR-719	GGT5	Tumor	6.4799617
TCGA-BR-719	GGT5	Tumor	3.47000534
TCGA-BR-770	GGT5	Tumor	4.09780175
TCGA-BR-770	GGT5	Tumor	4.35274827
TCGA-BR-771	GGT5	Tumor	4.10858432
TCGA-BR-771	GGT5	Tumor	4.47773291
TCGA-BR-771	GGT5	Tumor	3.91977903
TCGA-BR-772	GGT5	Tumor	4.67180103
TCGA-BR-772	GGT5	Tumor	4.30320589
TCGA-BR-785	GGT5	Tumor	4.33579154
TCGA-BR-790	GGT5	Tumor	5.46987781
TCGA-BR-795	GGT5	Tumor	6.43479757
TCGA-BR-795	GGT5	Tumor	5.23029557
TCGA-BR-795	GGT5	Tumor	5.76400084
TCGA-BR-805	GGT5	Tumor	5.41433925
TCGA-BR-805	GGT5	Tumor	5.09481658
TCGA-BR-806	GGT5	Tumor	4.53587821
TCGA-BR-807	GGT5	Tumor	3.1864303
TCGA-BR-808	GGT5	Tumor	5.70610299
TCGA-BR-808	GGT5	Tumor	5.09201874
TCGA-BR-828	GGT5	Tumor	5.26803021
TCGA-BR-828	GGT5	Tumor	3.99850022

TCGA-BR-828	GGT5	Tumor	4.71381297
TCGA-BR-829	GGT5	Tumor	6.03077388
TCGA-BR-829	GGT5	Tumor	2.63805117
TCGA-BR-829	GGT5	Tumor	4.37069923
TCGA-BR-829	GGT5	Tumor	4.65429747
TCGA-BR-836	GGT5	Tumor	3.51996134
TCGA-BR-836	GGT5	Tumor	5.89536256
TCGA-BR-836	GGT5	Tumor	5.51816732
TCGA-BR-836	GGT5	Tumor	5.64271469
TCGA-BR-836	GGT5	Tumor	5.65613898
TCGA-BR-836	GGT5	Tumor	3.3459757
TCGA-BR-836	GGT5	Tumor	4.14937144
TCGA-BR-837	GGT5	Tumor	4.41797634
TCGA-BR-837	GGT5	Tumor	2.78765119
TCGA-BR-837	GGT5	Tumor	4.79579045
TCGA-BR-838	GGT5	Tumor	5.34047691
TCGA-BR-838	GGT5	Tumor	5.0880678
TCGA-BR-838	GGT5	Tumor	4.43568359
TCGA-BR-838	GGT5	Tumor	6.11023017
TCGA-BR-848	GGT5	Tumor	3.70552366
TCGA-BR-848	GGT5	Tumor	4.71874854
TCGA-BR-848	GGT5	Tumor	4.47914076
TCGA-BR-848	GGT5	Tumor	4.0710639
TCGA-BR-848	GGT5	Tumor	2.96463449
TCGA-BR-858	GGT5	Tumor	4.66917645
TCGA-BR-858	GGT5	Tumor	2.47423293
TCGA-BR-859	GGT5	Tumor	5.30177729
TCGA-BR-859	GGT5	Tumor	4.04735489
TCGA-BR-859	GGT5	Tumor	5.851141
TCGA-BR-867	GGT5	Tumor	1.35056045
TCGA-BR-867	GGT5	Tumor	5.7908987
TCGA-BR-867	GGT5	Tumor	4.5745146
TCGA-BR-867	GGT5	Tumor	4.50717293
TCGA-BR-868	GGT5	Tumor	3.49202863
TCGA-BR-868	GGT5	Tumor	4.72447538
TCGA-BR-868	GGT5	Tumor	4.90347718
TCGA-BR-868	GGT5	Tumor	5.28946257
TCGA-BR-868	GGT5	Tumor	4.31039422
TCGA-BR-869	GGT5	Tumor	4.17230469
TCGA-BR-A44	GGT5	Tumor	4.11861465
TCGA-BR-A44	GGT5	Tumor	3.95226267
TCGA-BR-A4C	GGT5	Tumor	2.23452046
TCGA-BR-A4C	GGT5	Tumor	3.33670428
TCGA-BR-A4I	GGT5	Tumor	4.62140753
TCGA-BR-A4J	GGT5	Tumor	3.63790315
TCGA-BR-A4J	GGT5	Tumor	4.82969573
TCGA-BR-A4J	GGT5	Tumor	4.48520844
TCGA-BR-A4J	GGT5	Tumor	4.52520146
TCGA-BR-A4J	GGT5	Tumor	4.12802926
TCGA-BR-A4J	GGT5	Tumor	4.22696313

TCGA-BR-A4F	GGT5	Tumor	3.31414934
TCGA-BR-A4C	GGT5	Tumor	2.07687499
TCGA-CD-579	GGT5	Tumor	5.3110893
TCGA-CD-579	GGT5	Tumor	4.28843054
TCGA-CD-580	GGT5	Tumor	3.32556469
TCGA-CD-580	GGT5	Tumor	3.9105886
TCGA-CD-580	GGT5	Tumor	5.9689753
TCGA-CD-580	GGT5	Tumor	4.41262377
TCGA-CD-581	GGT5	Tumor	6.53491267
TCGA-CD-852	GGT5	Tumor	5.9778762
TCGA-CD-852	GGT5	Tumor	4.29290745
TCGA-CD-852	GGT5	Tumor	4.70399491
TCGA-CD-852	GGT5	Tumor	3.38395551
TCGA-CD-852	GGT5	Tumor	3.89004529
TCGA-CD-852	GGT5	Tumor	4.78526966
TCGA-CD-853	GGT5	Tumor	5.41585832
TCGA-CD-853	GGT5	Tumor	3.96562079
TCGA-CD-853	GGT5	Tumor	4.72081831
TCGA-CD-853	GGT5	Tumor	4.4008811
TCGA-CD-853	GGT5	Tumor	3.80297575
TCGA-CD-853	GGT5	Tumor	3.95353708
TCGA-CD-A48	GGT5	Tumor	3.79631473
TCGA-CD-A48	GGT5	Tumor	3.66964927
TCGA-CD-A48	GGT5	Tumor	5.20638468
TCGA-CD-A48	GGT5	Tumor	2.85859487
TCGA-CD-A48	GGT5	Tumor	4.1136902
TCGA-CD-A4I	GGT5	Tumor	3.09805454
TCGA-CD-A4I	GGT5	Tumor	3.22966547
TCGA-CG-430	GGT5	Tumor	3.93088062
TCGA-CG-430	GGT5	Tumor	3.44129246
TCGA-CG-430	GGT5	Tumor	4.47191141
TCGA-CG-430	GGT5	Tumor	3.4879834
TCGA-CG-443	GGT5	Tumor	1.85263549
TCGA-CG-443	GGT5	Tumor	4.66978098
TCGA-CG-443	GGT5	Tumor	3.51267565
TCGA-CG-444	GGT5	Tumor	2.90868585
TCGA-CG-444	GGT5	Tumor	5.12637341
TCGA-CG-444	GGT5	Tumor	2.82174834
TCGA-CG-444	GGT5	Tumor	3.06105248
TCGA-CG-444	GGT5	Tumor	3.55678255
TCGA-CG-446	GGT5	Tumor	3.11797473
TCGA-CG-446	GGT5	Tumor	5.96935403
TCGA-CG-446	GGT5	Tumor	3.40851242
TCGA-CG-446	GGT5	Tumor	2.10085131
TCGA-CG-446	GGT5	Tumor	1.9063971
TCGA-CG-447	GGT5	Tumor	4.78766854
TCGA-CG-447	GGT5	Tumor	4.73999978
TCGA-CG-447	GGT5	Tumor	3.37380056
TCGA-CG-571	GGT5	Tumor	3.1812865
TCGA-CG-571	GGT5	Tumor	3.64260879

TCGA-CG-571	GGT5	Tumor	3.5735983
TCGA-CG-571	GGT5	Tumor	5.40564782
TCGA-CG-572	GGT5	Tumor	5.0895233
TCGA-CG-572	GGT5	Tumor	3.83951425
TCGA-CG-572	GGT5	Tumor	4.14982552
TCGA-CG-572	GGT5	Tumor	3.36793093
TCGA-CG-572	GGT5	Tumor	4.61607419
TCGA-CG-572	GGT5	Tumor	2.71933978
TCGA-CG-572	GGT5	Tumor	2.23572862
TCGA-CG-573	GGT5	Tumor	3.19154465
TCGA-CG-573	GGT5	Tumor	3.80662297
TCGA-D7-557	GGT5	Tumor	4.26404473
TCGA-D7-557	GGT5	Tumor	5.22633814
TCGA-D7-651	GGT5	Tumor	4.14119411
TCGA-D7-652	GGT5	Tumor	5.79544547
TCGA-D7-652	GGT5	Tumor	5.06033595
TCGA-D7-652	GGT5	Tumor	6.24841966
TCGA-D7-652	GGT5	Tumor	5.70513898
TCGA-D7-652	GGT5	Tumor	4.99114356
TCGA-D7-652	GGT5	Tumor	3.38046917
TCGA-D7-652	GGT5	Tumor	3.77323931
TCGA-D7-652	GGT5	Tumor	3.01576762
TCGA-D7-681	GGT5	Tumor	3.95202141
TCGA-D7-681	GGT5	Tumor	5.35439766
TCGA-D7-682	GGT5	Tumor	3.44714386
TCGA-D7-857	GGT5	Tumor	4.65844531
TCGA-D7-857	GGT5	Tumor	5.27102879
TCGA-D7-857	GGT5	Tumor	3.13526402
TCGA-D7-857	GGT5	Tumor	6.18239902
TCGA-D7-857	GGT5	Tumor	4.2035634
TCGA-D7-857	GGT5	Tumor	4.07022345
TCGA-D7-857	GGT5	Tumor	4.82978417
TCGA-D7-857	GGT5	Tumor	5.72973342
TCGA-D7-A4Y	GGT5	Tumor	4.79038917
TCGA-D7-A4Y	GGT5	Tumor	3.33509597
TCGA-D7-A4Z	GGT5	Tumor	4.30510341
TCGA-D7-A6E	GGT5	Tumor	3.12581937
TCGA-D7-A6E	GGT5	Tumor	3.14927991
TCGA-D7-A6E	GGT5	Tumor	4.6266131
TCGA-D7-A6E	GGT5	Tumor	3.82050204
TCGA-D7-A6F	GGT5	Tumor	3.44083426
TCGA-D7-A6F	GGT5	Tumor	3.20577457
TCGA-D7-A74	GGT5	Tumor	4.79745107
TCGA-D7-A74	GGT5	Tumor	5.46354621
TCGA-D7-A74	GGT5	Tumor	2.1994425
TCGA-EQ-812	GGT5	Tumor	4.50852219
TCGA-F1-617	GGT5	Tumor	2.6417252
TCGA-F1-687	GGT5	Tumor	4.29758296
TCGA-F1-687	GGT5	Tumor	3.40716352
TCGA-F1-A44	GGT5	Tumor	4.73759472

TCGA-F1-A72	GGT5	Tumor	4.23464416
TCGA-FP-773	GGT5	Tumor	3.44804262
TCGA-FP-782	GGT5	Tumor	4.55089172
TCGA-FP-791	GGT5	Tumor	5.30459899
TCGA-FP-799	GGT5	Tumor	5.31365398
TCGA-FP-809	GGT5	Tumor	4.29859491
TCGA-FP-820	GGT5	Tumor	5.80247168
TCGA-FP-821	GGT5	Tumor	6.14648106
TCGA-FP-821	GGT5	Tumor	2.94801295
TCGA-FP-863	GGT5	Tumor	4.61079755
TCGA-FP-A4B	GGT5	Tumor	5.64567626
TCGA-FP-A8C	GGT5	Tumor	3.26605851
TCGA-FP-A9T	GGT5	Tumor	3.16186464
TCGA-HF-713	GGT5	Tumor	3.78071176
TCGA-HF-713	GGT5	Tumor	3.82914543
TCGA-HF-713	GGT5	Tumor	4.2085595
TCGA-HF-713	GGT5	Tumor	3.41740557
TCGA-HF-A5N	GGT5	Tumor	2.01340633
TCGA-HJ-759	GGT5	Tumor	3.74044723
TCGA-HU-823	GGT5	Tumor	3.56460465
TCGA-HU-824	GGT5	Tumor	1.07503792
TCGA-HU-824	GGT5	Tumor	3.61961572
TCGA-HU-86(	GGT5	Tumor	3.65423544
TCGA-HU-86(	GGT5	Tumor	4.66164756
TCGA-HU-86(	GGT5	Tumor	4.1387929
TCGA-HU-861	GGT5	Tumor	4.08802224
TCGA-HU-A4(	GGT5	Tumor	3.22976011
TCGA-HU-A4(	GGT5	Tumor	3.04904785
TCGA-HU-A4(	GGT5	Tumor	3.09629829
TCGA-HU-A4(	GGT5	Tumor	0.9464546
TCGA-HU-A4(	GGT5	Tumor	3.35400215
TCGA-HU-A4(	GGT5	Tumor	2.22390241
TCGA-HU-A4(	GGT5	Tumor	2.76842441
TCGA-HU-A4(	GGT5	Tumor	2.20649037
TCGA-HU-A4(	GGT5	Tumor	4.52612658
TCGA-HU-A4(	GGT5	Tumor	2.97588932
TCGA-HU-A4(	GGT5	Tumor	3.53606625
TCGA-HU-A4(	GGT5	Tumor	2.95924245
TCGA-HU-A4(	GGT5	Tumor	2.67990728
TCGA-HU-A4(	GGT5	Tumor	2.46396047
TCGA-HU-A4(	GGT5	Tumor	5.09692542
TCGA-HU-A4I	GGT5	Tumor	2.71741258
TCGA-HU-A4I	GGT5	Tumor	3.08183383
TCGA-HU-A4I	GGT5	Tumor	2.91035521
TCGA-HU-A4I	GGT5	Tumor	3.43295991
TCGA-HU-A4I	GGT5	Tumor	2.58983471
TCGA-HU-A4I	GGT5	Tumor	3.33375598
TCGA-HU-A4I	GGT5	Tumor	1.7439122
TCGA-HU-A4I	GGT5	Tumor	3.45803115
TCGA-HU-A4I	GGT5	Tumor	3.52151004

TCGA-IN-780(	GGT5	Tumor	3.78412865
TCGA-IN-780:	GGT5	Tumor	5.73675742
TCGA-IN-846:	GGT5	Tumor	3.45078797
TCGA-IN-866:	GGT5	Tumor	3.74499327
TCGA-IN-A6R	GGT5	Tumor	2.62793265
TCGA-IN-A6R	GGT5	Tumor	1.82414375
TCGA-IN-A6R	GGT5	Tumor	3.19318726
TCGA-IN-A6R	GGT5	Tumor	2.63926771
TCGA-IN-A6R	GGT5	Tumor	2.14531592
TCGA-IN-A6R	GGT5	Tumor	3.30658
TCGA-IN-A6R	GGT5	Tumor	2.37604115
TCGA-IN-A7N	GGT5	Tumor	3.06315548
TCGA-IN-A7N	GGT5	Tumor	4.0896974
TCGA-IN-A7N	GGT5	Tumor	4.40723626
TCGA-IN-AB1	GGT5	Tumor	2.01014664
TCGA-IN-AB1	GGT5	Tumor	3.65780736
TCGA-IP-796:	GGT5	Tumor	4.38327783
TCGA-KB-A6F	GGT5	Tumor	3.15556744
TCGA-KB-A93	GGT5	Tumor	6.52213467
TCGA-KB-A93	GGT5	Tumor	1.80455734
TCGA-KB-A93	GGT5	Tumor	3.89334557
TCGA-MX-A5	GGT5	Tumor	6.28067167
TCGA-MX-A5	GGT5	Tumor	4.51053521
TCGA-MX-A6	GGT5	Tumor	5.63098443
TCGA-MX-A6	GGT5	Tumor	3.84395993
TCGA-R5-A7(	GGT5	Tumor	4.04759789
TCGA-R5-A7Z	GGT5	Tumor	2.0035716
TCGA-R5-A7Z	GGT5	Tumor	3.07963793
TCGA-R5-A7Z	GGT5	Tumor	1.88901504
TCGA-R5-A7Z	GGT5	Tumor	1.70204287
TCGA-R5-A8(	GGT5	Tumor	3.32252522
TCGA-RD-A7E	GGT5	Tumor	4.42397106
TCGA-RD-A7E	GGT5	Tumor	2.5525249
TCGA-RD-A7E	GGT5	Tumor	5.08458053
TCGA-RD-A7(	GGT5	Tumor	3.31944887
TCGA-RD-A8I	GGT5	Tumor	3.4733952
TCGA-RD-A8I	GGT5	Tumor	4.33372749
TCGA-RD-A8I	GGT5	Tumor	3.81671448
TCGA-RD-A8I	GGT5	Tumor	4.18123401
TCGA-RD-A8I	GGT5	Tumor	4.4515775
TCGA-RD-A8I	GGT5	Tumor	3.50367781
TCGA-RD-A8I	GGT5	Tumor	5.60533981
TCGA-RD-A8I	GGT5	Tumor	3.88421785
TCGA-RD-A8I	GGT5	Tumor	5.77855162
TCGA-RD-A8I	GGT5	Tumor	4.39766515
TCGA-SW-A7I	GGT5	Tumor	3.41174537
TCGA-SW-A7I	GGT5	Tumor	3.53789089
TCGA-VQ-A8I	GGT5	Tumor	2.72693272
TCGA-VQ-A8I	GGT5	Tumor	2.96093104
TCGA-VQ-A8I	GGT5	Tumor	1.4882535

TCGA-VQ-A8I	GGT5	Tumor	3.22433243
TCGA-VQ-A8I	GGT5	Tumor	2.48219558
TCGA-VQ-A8I	GGT5	Tumor	4.86628489
TCGA-VQ-A8I	GGT5	Tumor	3.15200509
TCGA-VQ-A8I	GGT5	Tumor	3.60967876
TCGA-VQ-A8I	GGT5	Tumor	2.67976037
TCGA-VQ-A8I	GGT5	Tumor	3.60334906
TCGA-VQ-A8I	GGT5	Tumor	4.39154416
TCGA-VQ-A8I	GGT5	Tumor	3.75043102
TCGA-VQ-A8I	GGT5	Tumor	3.04817208
TCGA-VQ-A8I	GGT5	Tumor	4.80859765
TCGA-VQ-A8I	GGT5	Tumor	4.66091471
TCGA-VQ-A8I	GGT5	Tumor	3.57873687
TCGA-VQ-A8I	GGT5	Tumor	4.78313055
TCGA-VQ-A8I	GGT5	Tumor	2.21211969
TCGA-VQ-A8I	GGT5	Tumor	2.69083818
TCGA-VQ-A8I	GGT5	Tumor	3.73282592
TCGA-VQ-A8I	GGT5	Tumor	3.75955273
TCGA-VQ-A8I	GGT5	Tumor	3.78685963
TCGA-VQ-A8I	GGT5	Tumor	4.45976242
TCGA-VQ-A8I	GGT5	Tumor	6.71461619
TCGA-VQ-A8I	GGT5	Tumor	2.60154822
TCGA-VQ-A8I	GGT5	Tumor	2.40401058
TCGA-VQ-A9:	GGT5	Tumor	5.30420276
TCGA-VQ-A9:	GGT5	Tumor	4.33297845
TCGA-VQ-A9:	GGT5	Tumor	2.6161546
TCGA-VQ-A9:	GGT5	Tumor	3.40368259
TCGA-VQ-A9:	GGT5	Tumor	2.71427419
TCGA-VQ-A9:	GGT5	Tumor	4.79033135
TCGA-VQ-A9:	GGT5	Tumor	3.14398338
TCGA-VQ-A9:	GGT5	Tumor	2.92790191
TCGA-VQ-A9:	GGT5	Tumor	2.81876039
TCGA-VQ-A9:	GGT5	Tumor	1.45152352
TCGA-VQ-A9:	GGT5	Tumor	5.12648571
TCGA-VQ-A9:	GGT5	Tumor	2.17616198
TCGA-VQ-A9:	GGT5	Tumor	4.56268108
TCGA-VQ-A9:	GGT5	Tumor	4.28159395
TCGA-VQ-A9:	GGT5	Tumor	3.75455107
TCGA-VQ-A9:	GGT5	Tumor	2.60609033
TCGA-VQ-A9:	GGT5	Tumor	3.25882683
TCGA-VQ-A9:	GGT5	Tumor	4.50518735
TCGA-VQ-A9:	GGT5	Tumor	4.35610844
TCGA-VQ-A9‹	GGT5	Tumor	3.48750881
TCGA-VQ-A9‹	GGT5	Tumor	4.96587874
TCGA-VQ-A9‹	GGT5	Tumor	4.87120921
TCGA-VQ-A9‹	GGT5	Tumor	3.96333672
TCGA-VQ-A9‹	GGT5	Tumor	5.20408619
TCGA-VQ-AA(	GGT5	Tumor	5.12691668
TCGA-VQ-AA(	GGT5	Tumor	3.35538478
TCGA-VQ-AA(	GGT5	Tumor	2.38937868

TCGA-VQ-AA(	GGT5	Tumor	3.78701358
TCGA-VQ-AA(	GGT5	Tumor	2.56569837
TCGA-VQ-AA(	GGT5	Tumor	3.8734811
TCGA-VQ-AA(	GGT5	Tumor	3.27710877
TCGA-VQ-AA(	GGT5	Tumor	4.24256678
TCGA-VQ-AA(	GGT5	Tumor	4.63779973
TCGA-ZA-A8F	GGT5	Tumor	5.04450648
TCGA-ZQ-A9(	GGT5	Tumor	4.91049342

rownames(datas)	Type	values
GSM1308392	G1(n=21)	2.772183
GSM1308393	G1(n=21)	2.791138
GSM1308394	G1(n=21)	2.811305
GSM1308395	G1(n=21)	2.888706
GSM1308396	G1(n=21)	2.766308
GSM1308397	G1(n=21)	2.820558
GSM1308398	G1(n=21)	2.807148
GSM1308399	G1(n=21)	2.771545
GSM1308400	G1(n=21)	2.764021
GSM1308401	G1(n=21)	2.780918
GSM1308402	G1(n=21)	2.745655
GSM1308403	G1(n=21)	2.808753
GSM1308404	G1(n=21)	2.800974
GSM1308405	G1(n=21)	2.760406
GSM1308406	G1(n=21)	2.776453
GSM1308407	G1(n=21)	2.782413
GSM1308408	G1(n=21)	2.830849
GSM1308409	G1(n=21)	2.807246
GSM1308410	G1(n=21)	2.782291
GSM1308411	G1(n=21)	2.791711
GSM1308412	G1(n=21)	2.808158
GSM1308413	G2(n=111)	3.078238
GSM1308414	G2(n=111)	3.129191
GSM1308415	G2(n=111)	3.01339
GSM1308416	G2(n=111)	2.88831
GSM1308417	G2(n=111)	3.141141
GSM1308418	G2(n=111)	2.974197
GSM1308419	G2(n=111)	3.110772
GSM1308420	G2(n=111)	3.27546
GSM1308421	G2(n=111)	2.934896
GSM1308422	G2(n=111)	2.859757
GSM1308423	G2(n=111)	3.222442
GSM1308424	G2(n=111)	3.238416
GSM1308425	G2(n=111)	2.878138
GSM1308426	G2(n=111)	3.014167
GSM1308427	G2(n=111)	3.053354
GSM1308428	G2(n=111)	3.339927
GSM1308429	G2(n=111)	2.975641
GSM1308430	G2(n=111)	3.065754
GSM1308431	G2(n=111)	2.779452
GSM1308432	G2(n=111)	3.107381
GSM1308433	G2(n=111)	2.900454
GSM1308434	G2(n=111)	3.023843
GSM1308435	G2(n=111)	3.009765
GSM1308436	G2(n=111)	3.088467
GSM1308437	G2(n=111)	3.000796
GSM1308438	G2(n=111)	3.176287
GSM1308439	G2(n=111)	2.975768
GSM1308440	G2(n=111)	2.981025

GSM1308441	G2(n=111)	3.147403
GSM1308442	G2(n=111)	3.108378
GSM1308443	G2(n=111)	3.102497
GSM1308444	G2(n=111)	3.183476
GSM1308445	G2(n=111)	3.194925
GSM1308446	G2(n=111)	3.143676
GSM1308447	G2(n=111)	3.010806
GSM1308448	G2(n=111)	3.24503
GSM1308449	G2(n=111)	2.790693
GSM1308450	G2(n=111)	2.928545
GSM1308451	G2(n=111)	3.166878
GSM1308452	G2(n=111)	3.206542
GSM1308453	G2(n=111)	3.250446
GSM1308454	G2(n=111)	3.014885
GSM1308455	G2(n=111)	2.987863
GSM1308456	G2(n=111)	3.008514
GSM1308457	G2(n=111)	2.950501
GSM1308458	G2(n=111)	3.180397
GSM1308459	G2(n=111)	2.97921
GSM1308460	G2(n=111)	3.018065
GSM1308461	G2(n=111)	2.994487
GSM1308462	G2(n=111)	3.034992
GSM1308463	G2(n=111)	2.833335
GSM1308464	G2(n=111)	3.044852
GSM1308465	G2(n=111)	3.107807
GSM1308466	G2(n=111)	3.040796
GSM1308467	G2(n=111)	2.990451
GSM1308468	G2(n=111)	3.180035
GSM1308469	G2(n=111)	2.989559
GSM1308470	G2(n=111)	2.85021
GSM1308471	G2(n=111)	2.867292
GSM1308472	G2(n=111)	3.000614
GSM1308473	G2(n=111)	3.106901
GSM1308474	G2(n=111)	2.800756
GSM1308475	G2(n=111)	2.890496
GSM1308476	G2(n=111)	2.90391
GSM1308477	G2(n=111)	2.958504
GSM1308478	G2(n=111)	2.850155
GSM1308479	G2(n=111)	2.872125
GSM1308480	G2(n=111)	2.855515
GSM1308481	G2(n=111)	3.058415
GSM1308482	G2(n=111)	3.155372
GSM1308483	G2(n=111)	3.033426
GSM1308484	G2(n=111)	3.170302
GSM1308485	G2(n=111)	2.849094
GSM1308486	G2(n=111)	2.843181
GSM1308487	G2(n=111)	2.879899
GSM1308488	G2(n=111)	3.035391
GSM1308489	G2(n=111)	2.72863
GSM1308490	G2(n=111)	2.801107

GSM1308491	G2(n=111)	2.915428
GSM1308492	G2(n=111)	3.051426
GSM1308493	G2(n=111)	2.861403
GSM1308494	G2(n=111)	3.094681
GSM1308495	G2(n=111)	3.051607
GSM1308496	G2(n=111)	2.807383
GSM1308497	G2(n=111)	3.076984
GSM1308498	G2(n=111)	2.875152
GSM1308499	G2(n=111)	2.867665
GSM1308500	G2(n=111)	2.733251
GSM1308501	G2(n=111)	2.900402
GSM1308502	G2(n=111)	2.920619
GSM1308503	G2(n=111)	3.016419
GSM1308504	G2(n=111)	3.049021
GSM1308505	G2(n=111)	2.883216
GSM1308506	G2(n=111)	2.931765
GSM1308507	G2(n=111)	3.119891
GSM1308508	G2(n=111)	2.937371
GSM1308509	G2(n=111)	2.92788
GSM1308510	G2(n=111)	2.97972
GSM1308511	G2(n=111)	3.001644
GSM1308512	G2(n=111)	2.801374
GSM1308513	G2(n=111)	2.831356
GSM1308514	G2(n=111)	2.857404
GSM1308515	G2(n=111)	3.121367
GSM1308516	G2(n=111)	3.088912
GSM1308517	G2(n=111)	2.908496
GSM1308518	G2(n=111)	3.232141
GSM1308519	G2(n=111)	2.958383
GSM1308520	G2(n=111)	2.865125
GSM1308521	G2(n=111)	2.698996
GSM1308522	G2(n=111)	3.063777
GSM1308523	G2(n=111)	2.859081

rownames	Type	values
GSM2758(	G1(n=9)	3.177602
GSM2758(	G1(n=9)	3.211945
GSM2758(	G1(n=9)	3.376595
GSM2758(	G1(n=9)	3.217593
GSM2758(	G1(n=9)	3.330263
GSM2758(	G1(n=9)	3.144597
GSM2758(	G1(n=9)	3.222092
GSM2758(	G1(n=9)	3.22351
GSM2758(	G1(n=9)	3.212334
GSM2758(	G2(n=10)	3.385859
GSM2758(	G2(n=10)	3.259025
GSM2758(	G2(n=10)	3.412791
GSM2758(	G2(n=10)	3.525898
GSM2758(	G2(n=10)	3.29717
GSM2758(	G2(n=10)	3.15181
GSM2758(	G2(n=10)	3.48559
GSM2758(	G2(n=10)	3.483107
GSM2758(	G2(n=10)	3.359289
GSM2758(	G2(n=10)	3.42122

rownames	Type	values
GSM72346	G1(n=134)	3.04264
GSM72346	G1(n=134)	3.008095
GSM72346	G1(n=134)	2.990578
GSM72347	G1(n=134)	3.048667
GSM72347	G1(n=134)	3.078809
GSM72347	G1(n=134)	3.038479
GSM72347	G1(n=134)	3.141914
GSM72347	G1(n=134)	3.087573
GSM72348	G1(n=134)	3.071126
GSM72348	G1(n=134)	3.052027
GSM72348	G1(n=134)	3.070404
GSM72348	G1(n=134)	3.069916
GSM72348	G1(n=134)	3.077645
GSM72349	G1(n=134)	3.036987
GSM72349	G1(n=134)	3.09736
GSM72349	G1(n=134)	3.101656
GSM72349	G1(n=134)	3.061349
GSM72349	G1(n=134)	2.989649
GSM72350	G1(n=134)	3.063823
GSM72350	G1(n=134)	3.045928
GSM72350	G1(n=134)	3.147966
GSM72350	G1(n=134)	3.065202
GSM72350	G1(n=134)	3.107683
GSM72351	G1(n=134)	3.075599
GSM72351	G1(n=134)	3.024957
GSM72351	G1(n=134)	3.025829
GSM72351	G1(n=134)	3.119003
GSM72351	G1(n=134)	3.053756
GSM72352	G1(n=134)	3.033018
GSM72352	G1(n=134)	3.045122
GSM72352	G1(n=134)	3.02866
GSM72352	G1(n=134)	3.049251
GSM72352	G1(n=134)	3.028463
GSM72353	G1(n=134)	3.014202
GSM72353	G1(n=134)	3.048414
GSM72353	G1(n=134)	3.115094
GSM72353	G1(n=134)	3.073014
GSM72353	G1(n=134)	3.107182
GSM72354	G1(n=134)	3.11039
GSM72354	G1(n=134)	3.000914
GSM72354	G1(n=134)	2.986811
GSM72354	G1(n=134)	3.069068
GSM72354	G1(n=134)	3.063327
GSM72355	G1(n=134)	3.036388
GSM72355	G1(n=134)	2.997931
GSM72355	G1(n=134)	3.053953
GSM72355	G1(n=134)	3.005274
GSM72355	G1(n=134)	3.082445
GSM72356	G1(n=134)	3.113348

GSM72356	G1(n=134)	3.069122
GSM72356	G1(n=134)	3.033365
GSM72356	G1(n=134)	3.099856
GSM72356	G1(n=134)	3.08541
GSM72357	G1(n=134)	2.975456
GSM72357	G1(n=134)	3.071567
GSM72357	G1(n=134)	3.114215
GSM72357	G1(n=134)	3.074826
GSM72357	G1(n=134)	3.063463
GSM72358	G1(n=134)	3.080257
GSM72358	G1(n=134)	3.052133
GSM72358	G1(n=134)	3.075996
GSM72358	G1(n=134)	3.06629
GSM72358	G1(n=134)	3.121535
GSM72359	G1(n=134)	3.092246
GSM72359	G1(n=134)	3.085459
GSM72359	G1(n=134)	3.012509
GSM72359	G1(n=134)	3.097937
GSM72359	G1(n=134)	3.041608
GSM72360	G1(n=134)	3.107829
GSM72360	G1(n=134)	3.072108
GSM72360	G1(n=134)	3.126949
GSM72360	G1(n=134)	3.034564
GSM72360	G1(n=134)	3.091183
GSM72361	G1(n=134)	3.126729
GSM72361	G1(n=134)	3.165437
GSM72361	G1(n=134)	3.2329
GSM72361	G1(n=134)	3.11039
GSM72361	G1(n=134)	3.148618
GSM72362	G1(n=134)	3.171551
GSM72362	G1(n=134)	3.194827
GSM72362	G1(n=134)	3.252153
GSM72362	G1(n=134)	3.141028
GSM72362	G1(n=134)	3.192518
GSM72363	G1(n=134)	3.201897
GSM72363	G1(n=134)	3.167506
GSM72363	G1(n=134)	3.161513
GSM72363	G1(n=134)	3.184709
GSM72363	G1(n=134)	3.045973
GSM72364	G1(n=134)	3.124135
GSM72364	G1(n=134)	3.175736
GSM72364	G1(n=134)	3.121203
GSM72364	G1(n=134)	3.170125
GSM72364	G1(n=134)	3.189383
GSM72365	G1(n=134)	3.119204
GSM72365	G1(n=134)	3.106858
GSM72365	G1(n=134)	3.115698
GSM72365	G1(n=134)	3.17537
GSM72365	G1(n=134)	3.139936
GSM72366	G1(n=134)	3.150174

GSM72366	G1(n=134)	3.139791
GSM72366	G1(n=134)	3.15138
GSM72366	G1(n=134)	3.131565
GSM72366	G1(n=134)	3.182832
GSM72367	G1(n=134)	3.149369
GSM72367	G1(n=134)	3.182324
GSM72367	G1(n=134)	3.208157
GSM72367	G1(n=134)	3.146549
GSM72367	G1(n=134)	3.152606
GSM72368	G1(n=134)	3.142134
GSM72368	G1(n=134)	3.131565
GSM72368	G1(n=134)	3.169486
GSM72368	G1(n=134)	3.185988
GSM72368	G1(n=134)	3.13331
GSM72369	G1(n=134)	3.180874
GSM72369	G1(n=134)	3.235579
GSM72369	G1(n=134)	3.10432
GSM72369	G1(n=134)	3.116682
GSM72369	G1(n=134)	3.128466
GSM72370	G1(n=134)	3.091632
GSM72370	G1(n=134)	3.134369
GSM72370	G1(n=134)	3.102773
GSM72370	G1(n=134)	3.178481
GSM72370	G1(n=134)	3.171428
GSM72371	G1(n=134)	3.129717
GSM72371	G1(n=134)	3.121475
GSM72371	G1(n=134)	3.260166
GSM72371	G1(n=134)	3.173124
GSM72371	G1(n=134)	3.189551
GSM72372	G1(n=134)	3.10522
GSM72372	G1(n=134)	3.15605
GSM72372	G1(n=134)	3.130944
GSM72372	G1(n=134)	3.080398
GSM72372	G1(n=134)	3.171601
GSM72373	G1(n=134)	3.051228
GSM72346	G2(n=134)	3.116359
GSM72346	G2(n=134)	3.216982
GSM72346	G2(n=134)	3.056747
GSM72347	G2(n=134)	3.166459
GSM72347	G2(n=134)	3.23458
GSM72347	G2(n=134)	3.14032
GSM72347	G2(n=134)	3.156331
GSM72347	G2(n=134)	3.211574
GSM72348	G2(n=134)	3.162615
GSM72348	G2(n=134)	3.017938
GSM72348	G2(n=134)	3.002476
GSM72348	G2(n=134)	3.112983
GSM72348	G2(n=134)	3.032948
GSM72349	G2(n=134)	3.094357
GSM72349	G2(n=134)	3.170552

GSM72349	G2(n=134)	3.044145
GSM72349	G2(n=134)	3.070572
GSM72349	G2(n=134)	3.097005
GSM72350	G2(n=134)	3.101071
GSM72350	G2(n=134)	3.016497
GSM72350	G2(n=134)	3.26796
GSM72350	G2(n=134)	3.214752
GSM72350	G2(n=134)	3.161529
GSM72351	G2(n=134)	3.147183
GSM72351	G2(n=134)	3.169257
GSM72351	G2(n=134)	3.088619
GSM72351	G2(n=134)	3.076642
GSM72351	G2(n=134)	3.002919
GSM72352	G2(n=134)	2.940607
GSM72352	G2(n=134)	3.166986
GSM72352	G2(n=134)	3.048117
GSM72352	G2(n=134)	2.994469
GSM72352	G2(n=134)	2.947202
GSM72353	G2(n=134)	3.111945
GSM72353	G2(n=134)	3.173025
GSM72353	G2(n=134)	3.024371
GSM72353	G2(n=134)	3.049258
GSM72353	G2(n=134)	3.111067
GSM72354	G2(n=134)	3.148505
GSM72354	G2(n=134)	3.049258
GSM72354	G2(n=134)	2.950009
GSM72354	G2(n=134)	2.959016
GSM72354	G2(n=134)	3.073849
GSM72355	G2(n=134)	3.097123
GSM72355	G2(n=134)	3.023443
GSM72355	G2(n=134)	3.161777
GSM72355	G2(n=134)	3.178354
GSM72355	G2(n=134)	3.209638
GSM72356	G2(n=134)	3.147415
GSM72356	G2(n=134)	3.112216
GSM72356	G2(n=134)	3.093788
GSM72356	G2(n=134)	3.138493
GSM72356	G2(n=134)	3.136416
GSM72357	G2(n=134)	3.084074
GSM72357	G2(n=134)	2.99966
GSM72357	G2(n=134)	3.050391
GSM72357	G2(n=134)	3.02178
GSM72357	G2(n=134)	3.208898
GSM72358	G2(n=134)	3.072643
GSM72358	G2(n=134)	3.067582
GSM72358	G2(n=134)	3.252375
GSM72358	G2(n=134)	3.021117
GSM72358	G2(n=134)	2.976331
GSM72359	G2(n=134)	3.012344
GSM72359	G2(n=134)	3.142483

GSM72359	G2(n=134)	3.044034
GSM72359	G2(n=134)	3.00286
GSM72359	G2(n=134)	3.099677
GSM72360	G2(n=134)	3.080086
GSM72360	G2(n=134)	3.106074
GSM72360	G2(n=134)	3.238064
GSM72360	G2(n=134)	3.200413
GSM72360	G2(n=134)	3.240265
GSM72361	G2(n=134)	3.388525
GSM72361	G2(n=134)	3.268499
GSM72361	G2(n=134)	3.109192
GSM72361	G2(n=134)	3.255859
GSM72361	G2(n=134)	3.186756
GSM72362	G2(n=134)	3.181948
GSM72362	G2(n=134)	3.208591
GSM72362	G2(n=134)	3.140063
GSM72362	G2(n=134)	3.168747
GSM72362	G2(n=134)	3.156493
GSM72363	G2(n=134)	3.159182
GSM72363	G2(n=134)	3.113099
GSM72363	G2(n=134)	3.159861
GSM72363	G2(n=134)	3.091698
GSM72363	G2(n=134)	3.115152
GSM72364	G2(n=134)	3.22975
GSM72364	G2(n=134)	3.262678
GSM72364	G2(n=134)	3.068693
GSM72364	G2(n=134)	3.19604
GSM72364	G2(n=134)	3.113598
GSM72365	G2(n=134)	3.116572
GSM72365	G2(n=134)	3.226846
GSM72365	G2(n=134)	3.173081
GSM72365	G2(n=134)	3.094789
GSM72365	G2(n=134)	3.186279
GSM72366	G2(n=134)	3.112436
GSM72366	G2(n=134)	3.153498
GSM72366	G2(n=134)	3.124783
GSM72366	G2(n=134)	3.233047
GSM72366	G2(n=134)	3.157474
GSM72367	G2(n=134)	3.297193
GSM72367	G2(n=134)	3.366701
GSM72367	G2(n=134)	3.232422
GSM72367	G2(n=134)	3.155343
GSM72367	G2(n=134)	3.248857
GSM72368	G2(n=134)	3.115754
GSM72368	G2(n=134)	3.36021
GSM72368	G2(n=134)	3.144768
GSM72368	G2(n=134)	3.174225
GSM72368	G2(n=134)	3.099348
GSM72369	G2(n=134)	3.191956
GSM72369	G2(n=134)	3.216982

GSM72369	G2(n=134)	3.172019
GSM72369	G2(n=134)	3.248501
GSM72369	G2(n=134)	3.130678
GSM72370	G2(n=134)	3.178749
GSM72370	G2(n=134)	3.202954
GSM72370	G2(n=134)	3.1398
GSM72370	G2(n=134)	3.239381
GSM72370	G2(n=134)	3.084267
GSM72371	G2(n=134)	3.153719
GSM72371	G2(n=134)	3.192563
GSM72371	G2(n=134)	3.119609
GSM72371	G2(n=134)	3.064072
GSM72371	G2(n=134)	3.164566
GSM72372	G2(n=134)	3.279616
GSM72372	G2(n=134)	3.165093
GSM72372	G2(n=134)	2.990711
GSM72372	G2(n=134)	3.162256
GSM72372	G2(n=134)	3.078589
GSM72373	G2(n=134)	3.171581
