# Supplementary material for: Identification of GGT5 as a Novel Prognostic Biomarker for Gastric Cancer and its Correlation With Immune Cell Infiltration
Source: Front Genet. 2022 Mar 18;13:810292. doi: 10.3389/fgene.2022.810292 (PMC8971189; doi:10.3389/fgene.2022.810292)
Supplement: Supplementary file 11 [file DataSheet5.PDF]

id	logFC	AveExpr	t	P.Value	adj.P.Val	B
LHFP	1.185985	8.507689	9.076701	1.22E-15	8.26E-12	24.96871
IGFBP4	1.106637	11.06425	8.858585	4.22E-15	1.90E-11	23.77751
SERPINF1	1.104116	10.11929	8.146277	2.28E-13	4.40E-10	19.94046
C7	1.862704	8.500797	7.797814	1.55E-12	1.37E-09	18.0998
OGN	1.340078	6.112473	7.537455	6.35E-12	3.18E-09	16.74338
AKAP12	1.079239	8.899066	7.529399	6.63E-12	3.20E-09	16.70169
SPARCL1	1.047957	11.23183	7.340953	1.82E-11	7.21E-09	15.73152
THBS4	1.358784	9.310396	7.227591	3.33E-11	9.99E-09	15.15275
CAV1	1.051439	9.13132	6.948084	1.45E-10	2.96E-08	13.74245
GHR	1.034007	5.611111	6.799549	3.12E-10	5.35E-08	13.00329
ACKR1	1.052355	8.203983	6.73615	4.33E-10	6.43E-08	12.69009
IGF1	1.06313	7.846967	6.730117	4.47E-10	6.56E-08	12.66036
MGP	1.233813	10.69967	6.680454	5.77E-10	7.79E-08	12.41611
HSPB6	1.18785	8.727778	6.631406	7.41E-10	9.27E-08	12.17575
ABCA8	1.385149	7.40578	6.565911	1.03E-09	1.17E-07	11.85616
ZFPM2	1.008379	6.389284	6.542352	1.17E-09	1.28E-07	11.74159
MYL9	1.483904	10.83467	6.513677	1.35E-09	1.40E-07	11.60242
APOD	1.333096	9.877613	6.4085	2.29E-09	2.18E-07	11.09463
SRPX	1.220835	7.806361	6.325184	3.48E-09	3.01E-07	10.69544
TAGLN	1.217853	12.1299	6.153869	8.13E-09	5.87E-07	9.8834
CXCL12	1.033218	8.303304	6.142227	8.60E-09	6.08E-07	9.828655
HAND2-A	1.069754	6.85291	6.082035	1.16E-08	7.58E-07	9.546533
SCRG1	1.346183	8.464157	6.079526	1.17E-08	7.60E-07	9.534805
FHL1	1.195577	8.846921	5.857116	3.44E-08	1.73E-06	8.506249
ASPN	1.049128	9.606568	5.665167	8.55E-08	3.63E-06	7.636608
BAG2	1.081375	6.675612	5.604924	1.13E-07	4.49E-06	7.36727
PLN	1.226664	7.169225	5.604576	1.14E-07	4.49E-06	7.365717
CNN1	1.504765	10.04043	5.512162	1.75E-07	6.36E-06	6.955996
ADH1B	1.003015	6.587583	5.390447	3.06E-07	9.73E-06	6.422868
CFD	1.061417	8.577999	5.142094	9.38E-07	2.37E-05	5.358845
MYLK	1.124582	10.57258	4.788092	4.38E-06	7.74E-05	3.900649
MYH11	1.158658	9.623552	4.564718	1.12E-05	0.000163	3.018126
ACTG2	1.384534	10.22797	4.389505	2.28E-05	0.000282	2.347348
SYNM	1.232309	9.854284	4.384424	2.33E-05	0.000287	2.328186
FABP4	1.163039	7.666486	4.230431	4.29E-05	0.00046	1.755323
HLA-DQA	1.076437	6.909446	3.776766	0.000238	0.001737	0.160171
KRT13	-1.05827	5.399121	-3.33569	0.0011	0.005674	-1.24984
