# Supplementary material for: Identification of GGT5 as a Novel Prognostic Biomarker for Gastric Cancer and its Correlation With Immune Cell Infiltration
Source: Front Genet. 2022 Mar 18;13:810292. doi: 10.3389/fgene.2022.810292 (PMC8971189; doi:10.3389/fgene.2022.810292)
Supplement: Supplementary file 12 [file DataSheet8.PDF]

ONTOLOGY	ID	Description	GeneRatio	BgRatio	pvalue	p.adjust	qvalue	geneID	Count
BP	GO:0030198	extracellular r	43/446	368/18670	6.0179E-18	1.2879E-14	1.0874E-14	AEBP1/BGN/(	43
BP	GO:0043062	extracellular s	46/446	422/18670	6.2856E-18	1.2879E-14	1.0874E-14	AEBP1/AGTR1	46
BP	GO:0006936	muscle contra	36/446	360/18670	4.0472E-13	5.5284E-10	4.6677E-10	ACTA2/ACTC1	36
BP	GO:0007586	digestion	21/446	139/18670	1.6442E-11	1.6845E-08	1.4223E-08	AQP1/FABP1/	21
BP	GO:0003012	muscle systen	37/446	465/18670	1.6372E-10	1.3419E-07	1.133E-07	ACTA2/ACTC1	37
BP	GO:0031589	cell-substrate	29/446	354/18670	8.6761E-09	5.9258E-06	5.0032E-06	APOD/COL16/	29
BP	GO:0034330	cell junction o	25/446	290/18670	3.4698E-08	2.0313E-05	1.7151E-05	APOD/CAV1/(	25
BP	GO:0010810	regulation of (	21/446	215/18670	5.2591E-08	2.694E-05	2.2746E-05	APOD/COL16/	21
BP	GO:0001655	urogenital sys	26/446	330/18670	1.1274E-07	5.1336E-05	4.3343E-05	ACTA2/AGTR1	26
BP	GO:1903779	regulation of (	12/446	73/18670	1.4656E-07	5.7266E-05	4.835E-05	ATP1A2/ATP2	12
BP	GO:0006937	regulation of r	18/446	171/18670	1.5371E-07	5.7266E-05	4.835E-05	ATP1A2/CASC	18
BP	GO:0072001	renal system (	24/446	293/18670	1.7042E-07	5.8199E-05	4.9138E-05	ACTA2/AGTR1	24
BP	GO:0010811	positive regul	15/446	121/18670	1.989E-07	6.2701E-05	5.2939E-05	COL16A1/FBL	15
BP	GO:0007178	transmembra	26/446	349/18670	3.3659E-07	8.8122E-05	7.4403E-05	CAV1/CDH5/F	26
BP	GO:0034329	cell junction a	21/446	241/18670	3.6539E-07	8.8122E-05	7.4403E-05	APOD/CAV1/(	21
BP	GO:0090092	regulation of t	21/446	241/18670	3.6539E-07	8.8122E-05	7.4403E-05	CAV1/CDH5/F	21
BP	GO:0006939	smooth muscl	14/446	110/18670	3.6556E-07	8.8122E-05	7.4403E-05	ACTA2/ATP1A	14
BP	GO:0061337	cardiac condu	16/446	146/18670	4.3533E-07	9.9109E-05	8.3679E-05	ATP1A2/ATP2	16
BP	GO:0090287	regulation of (	23/446	292/18670	6.1524E-07	0.0001327	0.00011204	CAV1/CDH5/F	23
BP	GO:0003018	vascular proce	17/446	173/18670	9.048E-07	0.00018539	0.00015653	ACTA2/AGTR1	17
BP	GO:0002685	regulation of l	18/446	196/18670	1.1714E-06	0.00022859	0.000193	APOD/F2RL1/	18
BP	GO:0090101	negative regu	14/446	126/18670	1.9377E-06	0.00035638	0.0003009	CAV1/FBN1/L	14
BP	GO:0002686	negative regu	9/446	49/18670	2.057E-06	0.00035638	0.0003009	APOD/CCL2/C	9
BP	GO:0035637	multicellular (	18/446	204/18670	2.0872E-06	0.00035638	0.0003009	ATP1A2/ATP2	18
BP	GO:0055123	digestive syst	15/446	146/18670	2.2674E-06	0.00037167	0.00031381	KLF5/CCNB1/	15
BP	GO:0048565	digestive tract	14/446	134/18670	4.0399E-06	0.00062491	0.00052762	KLF5/CCNB1/	14
BP	GO:0060047	heart contract	21/446	280/18670	4.1173E-06	0.00062491	0.00052762	ACTC1/ATP1A	21
BP	GO:0090257	regulation of r	20/446	259/18670	4.4978E-06	0.00065829	0.0005558	ATP1A2/ATP2	20
BP	GO:0007229	integrin-medi	12/446	103/18670	6.3865E-06	0.00090249	0.00076198	CDH17/COL16	12
BP	GO:0071466	cellular respo	16/446	180/18670	6.9785E-06	0.00091768	0.00077481	NAT1/AOC1/	16
BP	GO:0003015	heart process	21/446	290/18670	7.0989E-06	0.00091768	0.00077481	ACTC1/ATP1A	21
BP	GO:0019730	antimicrobial	13/446	122/18670	7.1659E-06	0.00091768	0.00077481	CTSG/DEFA5/	13
BP	GO:0033622	integrin activa	6/446	22/18670	9.6875E-06	0.00120301	0.00101572	CDH17/COL16	6
BP	GO:1903522	regulation of l	21/446	297/18670	1.0229E-05	0.00121045	0.001022	AGTR1/ATP1A	21
BP	GO:0008016	regulation of l	19/446	251/18670	1.0338E-05	0.00121045	0.001022	ATP1A2/ATP2	19
BP	GO:0030510	regulation of l	11/446	91/18670	1.085E-05	0.0012236	0.00103311	CDH5/FBN1/(	11
BP	GO:0090288	negative regu	15/446	166/18670	1.1048E-05	0.0012236	0.00103311	CAV1/FBN1/L	15
BP	GO:2000050	regulation of r	6/446	23/18670	1.2843E-05	0.00131986	0.00111438	SFRP1/SFRP2/	6
BP	GO:0001822	kidney develo	20/446	278/18670	1.2883E-05	0.00131986	0.00111438	ACTA2/AGTR1	20
BP	GO:0060485	mesenchyme	20/446	278/18670	1.2883E-05	0.00131986	0.00111438	ACTA2/ACTC1	20
BP	GO:0045785	positive regul	25/446	403/18670	1.4649E-05	0.00146418	0.00123623	CAV1/COL16A	25
BP	GO:0045216	cell-cell juncti	14/446	156/18670	2.3343E-05	0.00227758	0.00192299	CAV1/CDH1/(	14
BP	GO:0022600	digestive syst	11/446	100/18670	2.679E-05	0.00255312	0.00215564	AQP1/FABP1/	11
BP	GO:0003081	regulation of s	6/446	26/18670	2.7564E-05	0.00256406	0.00216487	AGTR1/CES1/	6
BP	GO:0050900	leukocyte mig	28/446	499/18670	2.8471E-05	0.00256406	0.00216487	APOB/APOD/(	28
BP	GO:0001101	response to a	22/446	343/18670	2.8782E-05	0.00256406	0.00216487	APOB/AQP1/	22
BP	GO:0007160	cell-matrix ad	17/446	225/18670	3.0747E-05	0.00268089	0.00226351	APOD/COL16/	17
BP	GO:0035296	regulation of t	13/446	143/18670	3.9704E-05	0.00325417	0.00274754	ACTA2/AGTR1	13
BP	GO:0050880	regulation of l	13/446	143/18670	3.9704E-05	0.00325417	0.00274754	ACTA2/AGTR1	13
BP	GO:0097746	regulation of l	13/446	143/18670	3.9704E-05	0.00325417	0.00274754	ACTA2/AGTR1	13
BP	GO:0035150	regulation of t	13/446	144/18670	4.2715E-05	0.00341356	0.00288212	ACTA2/AGTR1	13
BP	GO:0085029	extracellular r	6/446	28/18670	4.3315E-05	0.00341356	0.00288212	GPM6B/MFAI	6
BP	GO:0048871	multicellular (	27/446	485/18670	4.5256E-05	0.00343789	0.00290266	AQP1/AZGP1/	27
BP	GO:0042445	hormone met	17/446	232/18670	4.5302E-05	0.00343789	0.00290266	CES1/CMA1/(	17
BP	GO:0097305	response to al	17/446	233/18670	4.7815E-05	0.00356262	0.00300797	ACTC1/APOBI	17
BP	GO:0060537	muscle tissue	24/446	408/18670	5.0767E-05	0.00371506	0.00313668	ACTC1/KLF5/(	24
BP	GO:0086065	cell communi	8/446	56/18670	5.1777E-05	0.00372248	0.00314294	ATP1A2/CASC	8
BP	GO:0010669	epithelial stru	6/446	29/18670	5.3521E-05	0.00378153	0.0031928	CXADR/MUC6	6
BP	GO:0045778	positive regul	10/446	90/18670	5.685E-05	0.00392056	0.00331018	GPM6B/IGF1/	10
BP	GO:0042692	muscle cell dil	23/446	385/18670	5.7402E-05	0.00392056	0.00331018	ACTC1/KLF5/(	23
BP	GO:0030239	myofibril asse	9/446	73/18670	5.8592E-05	0.00393621	0.0033234	ACTC1/CASQ2	9
BP	GO:0086004	regulation of (	7/446	43/18670	6.4868E-05	0.0042876	0.00362008	ATP1A2/CAV1	7
BP	GO:0001991	regulation of s	5/446	19/18670	6.7056E-05	0.00436185	0.00368277	CES1/CMA1/(	5
BP	GO:0006941	striated muscl	14/446	174/18670	7.7784E-05	0.00498062	0.00420521	ACTC1/ATP1A	14
BP	GO:1901342	regulation of v	24/446	422/18670	8.5879E-05	0.00536997	0.00453394	AGTR1/ANXA	24
BP	GO:0051146	striated muscl	19/446	293/18670	8.6486E-05	0.00536997	0.00453394	ACTC1/KLF5/(	19
BP	GO:1903011	negative regu	4/446	11/18670	9.2801E-05	0.00567607	0.00479239	FBN1/LTF/GR	4
BP	GO:0034113	heterotypic ce	8/446	61/18670	9.6725E-05	0.00579715	0.00489462	CXADR/DSC2/	8

BP	GO:0030856	regulation of (	13/446	156/18670	9.7609E-05	0.00579715	0.00489462	CAV1/CDH5/S	13
BP	GO:0030509	BMP signaling	13/446	157/18670	0.00010416	0.00604908	0.00510732	CDH5/FBN1/C	13
BP	GO:0070252	actin-mediate	11/446	116/18670	0.00010569	0.00604908	0.00510732	ACTC1/ATP1A	11
BP	GO:0098742	cell-cell adhes	18/446	273/18670	0.00010705	0.00604908	0.00510732	ANXA3/CDH1,	18
BP	GO:0006942	regulation of s	10/446	97/18670	0.00010776	0.00604908	0.00510732	ATP1A2/CASC	10
BP	GO:0030277	maintenance	5/446	21/18670	0.00011281	0.00617165	0.00521081	MUC6/TFF1/T	5
BP	GO:0010951	negative regu	17/446	250/18670	0.000114	0.00617165	0.00521081	AQP1/SERPIN	17
BP	GO:0030278	regulation of (	15/446	203/18670	0.00011446	0.00617165	0.00521081	GPM6B/IGF1/	15
BP	GO:1903115	regulation of a	7/446	47/18670	0.00011661	0.00620616	0.00523995	ATP1A2/CAV1	7
BP	GO:0002002	regulation of a	4/446	12/18670	0.00013658	0.00701631	0.00592397	CES1/CMA1/C	4
BP	GO:0002003	angiotensin m	4/446	12/18670	0.00013658	0.00701631	0.00592397	CES1/CMA1/C	4
BP	GO:0098901	regulation of (	6/446	34/18670	0.00013697	0.00701631	0.00592397	CAV1/CXADR/	6
BP	GO:0006959	humoral immu	21/446	356/18670	0.00014266	0.00721772	0.00609402	SERPING1/C1	21
BP	GO:0055117	regulation of (	9/446	82/18670	0.00014639	0.00731583	0.00617686	ATP1A2/CASC	9
BP	GO:0002548	monocyte che	8/446	65/18670	0.00015255	0.00736998	0.00622258	CCL2/CCL19/C	8
BP	GO:0006940	regulation of s	8/446	65/18670	0.00015255	0.00736998	0.00622258	ATP1A2/CAV1	8
BP	GO:0048546	digestive tract	7/446	49/18670	0.00015287	0.00736998	0.00622258	FOXF1/SFRP1,	7
BP	GO:0032970	regulation of a	22/446	388/18670	0.00017538	0.00830602	0.00701289	ATP1A2/CAV1	22
BP	GO:0050886	endocrine pro	9/446	84/18670	0.00017634	0.00830602	0.00701289	AGTR1/CES1/	9
BP	GO:0014706	striated muscl	22/446	390/18670	0.00018857	0.00878113	0.00741404	ACTC1/KLF5/C	22
BP	GO:0048146	positive regul	7/446	51/18670	0.00019775	0.00910529	0.00768772	AQP1/CCNB1,	7
BP	GO:0010466	negative regu	17/446	262/18670	0.00020002	0.00910778	0.00768983	AQP1/SERPIN	17
BP	GO:0006805	xenobiotic me	11/446	125/18670	0.00020586	0.00927037	0.0078271	NAT1/AOC1/E	11
BP	GO:1903010	regulation of l	5/446	24/18670	0.00022211	0.00986475	0.00832895	FBN1/LTF/GR	5
BP	GO:0030514	negative regu	7/446	52/18670	0.00022387	0.00986475	0.00832895	FBN1/SFRP1/S	7
BP	GO:0071772	response to B	13/446	170/18670	0.00023063	0.00992759	0.008382	CDH5/FBN1/C	13
BP	GO:0071773	cellular respo	13/446	170/18670	0.00023063	0.00992759	0.008382	CDH5/FBN1/C	13
BP	GO:0086003	cardiac muscl	8/446	69/18670	0.00023256	0.00992759	0.008382	ATP1A2/CAV1	8
BP	GO:0060348	bone develop	15/446	217/18670	0.00023812	0.01006008	0.00849387	COL6A2/COL6	15
BP	GO:0001503	ossification	22/446	398/18670	0.00025048	0.0104743	0.0088436	GPM6B/TNC/	22
BP	GO:0090025	regulation of r	5/446	25/18670	0.00027223	0.01121818	0.00947167	CXCL12/SLIT2,	5
BP	GO:0055002	striated muscl	13/446	173/18670	0.00027375	0.01121818	0.00947167	ACTC1/CASQ2	13
BP	GO:0061005	cell differentia	7/446	54/18670	0.00028445	0.01154145	0.00974461	ACTA2/PROM	7
BP	GO:2000146	negative regu	20/446	349/18670	0.00029817	0.01197928	0.01011428	APOD/CDH1/(	20
BP	GO:0031099	regeneration	14/446	198/18670	0.00030249	0.01203501	0.01016133	ANXA3/APOD	14
BP	GO:0031032	actomyosin st	14/446	199/18670	0.00031845	0.01246286	0.01052257	ACTC1/CASQ2	14
BP	GO:0098900	regulation of a	7/446	55/18670	0.00031933	0.01246286	0.01052257	CAV1/CXADR/	7
BP	GO:0071560	cellular respo	16/446	249/18670	0.00034061	0.01316825	0.01111814	CAV1/CDH5/C	16
BP	GO:0001990	regulation of s	6/446	40/18670	0.00034639	0.01326629	0.01120091	AGTR1/CES1/	6
BP	GO:0046851	negative regu	4/446	15/18670	0.00035577	0.01337554	0.01129316	SFRP1/GREM1	4
BP	GO:2000095	regulation of V	4/446	15/18670	0.00035577	0.01337554	0.01129316	SFRP1/SFRP2/	4
BP	GO:0016485	protein proce	19/446	328/18670	0.00036706	0.01367468	0.01154573	AEBP1/SERPIN	19
BP	GO:0007517	muscle organ	22/446	410/18670	0.00037689	0.01382676	0.01167413	ACTC1/KLF5/C	22
BP	GO:0086001	cardiac muscl	8/446	74/18670	0.00037789	0.01382676	0.01167413	ATP1A2/CAV1	8
BP	GO:0045765	regulation of a	21/446	383/18670	0.00038321	0.01389727	0.01173367	AGTR1/ANXA3	21
BP	GO:0060326	cell chemotax	18/446	304/18670	0.00040107	0.01434885	0.01211494	AGTR1/CXADI	18
BP	GO:0002688	regulation of l	10/446	114/18670	0.00040713	0.01434885	0.01211494	F2RL1/CCL2/C	10
BP	GO:0030282	bone minerali	10/446	114/18670	0.00040713	0.01434885	0.01211494	GPM6B/IGF1/	10
BP	GO:0048545	response to st	21/446	385/18670	0.00041043	0.01434885	0.01211494	ANXA3/AQP1,	21
BP	GO:0070167	regulation of l	9/446	94/18670	0.00041317	0.01434885	0.01211494	GPM6B/CCN1	9
BP	GO:0010927	cellular comp	10/446	115/18670	0.0004366	0.01503502	0.01269428	ACTC1/CASQ2	10
BP	GO:0071559	response to tr	16/446	255/18670	0.00044304	0.01512875	0.01277342	CAV1/CDH5/C	16
BP	GO:0008217	regulation of l	13/446	182/18670	0.0004467	0.01512875	0.01277342	ACTA2/AGTR1	13
BP	GO:0030500	regulation of l	8/446	76/18670	0.00045358	0.01516833	0.01280684	GPM6B/CCN1	8
BP	GO:0060048	cardiac muscl	11/446	137/18670	0.00045545	0.01516833	0.01280684	ACTC1/ATP1A	11
BP	GO:0030336	negative regu	19/446	334/18670	0.00045897	0.01516833	0.01280684	APOD/CDH1/(	19
BP	GO:0033688	regulation of (	5/446	28/18670	0.00047473	0.01556367	0.01314063	CCN1/LTF/SFF	5
BP	GO:0030048	actin filament	11/446	138/18670	0.0004845	0.01575793	0.01330465	ACTC1/ATP1A	11
BP	GO:0045861	negative regu	20/446	363/18670	0.00049306	0.01583613	0.01337067	AQP1/SERPIN	20
BP	GO:0031102	neuron projec	7/446	59/18670	0.00049464	0.01583613	0.01337067	APOD/TNC/M	7
BP	GO:0007044	cell-substrate	9/446	97/18670	0.00052135	0.0165621	0.01398362	APOD/COL16/	9
BP	GO:0097529	myeloid leuko	14/446	210/18670	0.00054735	0.0171802	0.01450549	CXADR/ITGA1	14
BP	GO:0055001	muscle cell de	13/446	186/18670	0.0005492	0.0171802	0.01450549	ACTC1/CASQ2	13
BP	GO:0052547	regulation of p	23/446	452/18670	0.00058582	0.01797591	0.01517732	AQP1/SERPIN	23
BP	GO:0035850	epithelial cell	6/446	44/18670	0.00058779	0.01797591	0.01517732	ACTA2/PROM	6
BP	GO:0071622	regulation of g	6/446	44/18670	0.00058779	0.01797591	0.01517732	CCL19/THBS4,	6
BP	GO:0006721	terpenoid me	10/446	120/18670	0.00061182	0.01857211	0.0156807	APOB/CYP1B1	10
BP	GO:0033002	muscle cell pr	15/446	239/18670	0.00066043	0.01990037	0.01680217	APOD/CCNB1	15
BP	GO:0009410	response to xe	17/446	292/18670	0.00069643	0.02071663	0.01749135	NAT1/AOC1/A	17

BP	GO:0034109	homotypic cel	8/446	81/18670	0.00069763	0.02071663	0.01749135	CSRP1/CXADR	8
BP	GO:0071621	granulocyte cl	10/446	123/18670	0.00074235	0.02177603	0.01838581	CXADR/ITGA1	10
BP	GO:0007157	heterophilic c	6/446	46/18670	0.00074925	0.02177603	0.01838581	CXADR/ITGA5	6
BP	GO:0019731	antibacterial h	6/446	46/18670	0.00074925	0.02177603	0.01838581	CTSG/DEFA5/	6
BP	GO:0048660	regulation of s	12/446	169/18670	0.0007724	0.02194473	0.01852825	APOD/CNN1/	12
BP	GO:0033687	osteoblast pro	5/446	31/18670	0.00077383	0.02194473	0.01852825	CCN1/LTF/SFF	5
BP	GO:0034694	response to p	5/446	31/18670	0.00077383	0.02194473	0.01852825	APOB/TNC/CC	5
BP	GO:1901654	response to ke	13/446	193/18670	0.00077647	0.02194473	0.01852825	AQP1/CA9/CA	13
BP	GO:0050673	epithelial cell	22/446	434/18670	0.00080516	0.022518	0.01901227	AGTR1/CAV1/	22
BP	GO:0002683	negative regu	23/446	463/18670	0.00081169	0.022518	0.01901227	APOD/SERPIN	23
BP	GO:0045669	positive regula	7/446	64/18670	0.00081324	0.022518	0.01901227	IGF1/CCN1/LT	7
BP	GO:0048145	regulation of f	8/446	83/18670	0.00082085	0.02257603	0.01906127	AQP1/CCNB1/	8
BP	GO:0048659	smooth muscl	12/446	171/18670	0.00085651	0.02339989	0.01975686	APOD/CNN1/	12
BP	GO:0001523	retinoid meta	9/446	104/18670	0.00086589	0.02349951	0.01984097	APOB/CYP1B1	9
BP	GO:0048144	fibroblast pro	8/446	84/18670	0.00088869	0.02395954	0.02022939	AQP1/CCNB1/	8
BP	GO:0061448	connective tis	16/446	273/18670	0.00092341	0.02473295	0.02088238	ACTA2/COL6A	16
BP	GO:0098911	regulation of v	4/446	19/18670	0.00093653	0.02476057	0.0209057	CAV1/DSC2/D	4
BP	GO:2000696	regulation of e	4/446	19/18670	0.00093653	0.02476057	0.0209057	PROM1/ADIPO	4
BP	GO:0051271	negative regu	20/446	384/18670	0.0009897	0.02599865	0.02195103	APOD/CDH1/C	20
BP	GO:0030595	leukocyte che	14/446	224/18670	0.00102877	0.02662121	0.02247667	CXADR/F2RL1	14
BP	GO:0007179	transforming g	13/446	199/18670	0.00102982	0.02662121	0.02247667	CAV1/CDH5/F	13
BP	GO:0016486	peptide horm	5/446	33/18670	0.00103938	0.02662121	0.02247667	CES1/CMA1/C	5
BP	GO:1901890	positive regula	5/446	33/18670	0.00103938	0.02662121	0.02247667	CAV1/COL16A	5
BP	GO:0003044	regulation of s	6/446	49/18670	0.00105323	0.02680838	0.0226347	AGTR1/CES1/	6
BP	GO:0030858	positive regula	7/446	67/18670	0.00107088	0.02692312	0.02273158	CDH5/SFN/SF	7
BP	GO:0032387	negative regu	7/446	67/18670	0.00107088	0.02692312	0.02273158	APOD/CRYAB/	7
BP	GO:0034104	negative regu	4/446	20/18670	0.00114874	0.02853061	0.02408881	SFRP1/GREM1	4
BP	GO:0043931	ossification in	4/446	20/18670	0.00114874	0.02853061	0.02408881	IGF1/LTF/GRE	4
BP	GO:0001894	tissue homeos	14/446	227/18670	0.00116863	0.02884976	0.02435827	AZGP1/CA2/C	14
BP	GO:0003382	epithelial cell	5/446	34/18670	0.00119497	0.02932332	0.0247581	CLDN3/RAB25	5
BP	GO:1901888	regulation of c	8/446	88/18670	0.00120638	0.02935409	0.02478407	APOD/CAV1/C	8
BP	GO:0001676	long-chain fat	9/446	109/18670	0.00121055	0.02935409	0.02478407	CYP1B1/CYP2	9
BP	GO:0016101	diterpenoid m	9/446	110/18670	0.00129122	0.03112603	0.02628015	APOB/CYP1B1	9
BP	GO:0010959	regulation of m	20/446	394/18670	0.00134863	0.03223666	0.02721788	ATP1A2/ATP2	20
BP	GO:0001508	action potenti	10/446	133/18670	0.00135303	0.03223666	0.02721788	ATP1A2/CAV1	10
BP	GO:0071674	mononuclear	8/446	90/18670	0.00139603	0.03282556	0.02771509	CCL2/CCL19/C	8
BP	GO:0040013	negative regu	20/446	396/18670	0.00143239	0.03282556	0.02771509	APOD/CDH1/C	20
BP	GO:0010749	regulation of n	3/446	10/18670	0.00143382	0.03282556	0.02771509	ATP2B4/GUCY	3
BP	GO:0033690	positive regula	3/446	10/18670	0.00143382	0.03282556	0.02771509	CCN1/LTF/TM	3
BP	GO:0035672	oligopeptide t	3/446	10/18670	0.00143382	0.03282556	0.02771509	CA2/CDH17/S	3
BP	GO:0038063	collagen-activ	3/446	10/18670	0.00143382	0.03282556	0.02771509	COL4A1/COL4	3
BP	GO:2000425	regulation of a	3/446	10/18670	0.00143382	0.03282556	0.02771509	C3/C4B/CCL2	3
BP	GO:0055078	sodium ion ho	6/446	52/18670	0.00144393	0.03287356	0.02775562	AGTR1/ATP1A	6
BP	GO:0002064	epithelial cell	13/446	207/18670	0.001472	0.03332734	0.02813875	ACTA2/KLF5/C	13
BP	GO:0002385	mucosal immu	5/446	36/18670	0.00155691	0.03505609	0.02959836	DEFA5/LTF/PI	5
BP	GO:0002237	response to m	18/446	343/18670	0.00160278	0.03589179	0.03030395	APOB/C4B/CT	18
BP	GO:0044342	type B pancre	4/446	22/18670	0.00167013	0.03699558	0.03123589	IGFBP4/REG1A	4
BP	GO:0071379	cellular respo	4/446	22/18670	0.00167013	0.03699558	0.03123589	APOB/TNC/SF	4
BP	GO:0030199	collagen fibril	6/446	54/18670	0.00175995	0.03877567	0.03273885	AEBP1/CYP1B	6
BP	GO:0006720	isoprenoid me	10/446	139/18670	0.00188316	0.04112217	0.03472004	APOB/CYP1B1	10
BP	GO:0048705	skeletal syster	14/446	239/18670	0.00189783	0.04112217	0.03472004	COL6A2/COL6	14
BP	GO:0006182	cGMP biosynt	3/446	11/18670	0.0019367	0.04112217	0.03472004	GUCY1A1/GU	3
BP	GO:0006681	galactosylcera	3/446	11/18670	0.0019367	0.04112217	0.03472004	UGT8/GBA3/F	3
BP	GO:0006857	oligopeptide t	3/446	11/18670	0.0019367	0.04112217	0.03472004	CA2/CDH17/S	3
BP	GO:0014820	tonic smooth	3/446	11/18670	0.0019367	0.04112217	0.03472004	EDNRA/MYLK/	3
BP	GO:2001204	regulation of o	3/446	11/18670	0.0019367	0.04112217	0.03472004	FBN1/LTF/CLD	3
BP	GO:0050678	regulation of e	19/446	378/18670	0.00196925	0.04126006	0.03483646	AGTR1/CAV1/	19
BP	GO:0070977	bone maturat	4/446	23/18670	0.00198398	0.04126006	0.03483646	IGF1/LTF/GRE	4
BP	GO:0048009	insulin-like gro	5/446	38/18670	0.00199353	0.04126006	0.03483646	GHR/IGF1/IGF	5
BP	GO:0086005	ventricular ca	5/446	38/18670	0.00199353	0.04126006	0.03483646	CAV1/DSC2/D	5
BP	GO:1904037	positive regula	5/446	38/18670	0.00199353	0.04126006	0.03483646	CCL2/SFRP4/A	5
BP	GO:0097530	granulocyte m	10/446	141/18670	0.00209315	0.04310423	0.03639352	CXADR/ITGA1	10
BP	GO:0086002	cardiac muscle	6/446	56/18670	0.00212581	0.04315317	0.03643484	CAV1/DSC2/D	6
BP	GO:0090183	regulation of k	6/446	56/18670	0.00212581	0.04315317	0.03643484	PDGFRB/PROI	6
BP	GO:1902903	regulation of s	18/446	352/18670	0.00212712	0.04315317	0.03643484	AEBP1/CDH5/	18
BP	GO:0034332	adherens junc	10/446	142/18670	0.002205	0.04439537	0.03748365	APOD/CDH1/C	10
BP	GO:0045927	positive regula	15/446	270/18670	0.00222502	0.04439537	0.03748365	CCNB1/FN1/G	15
BP	GO:0042310	vasoconstricti	7/446	76/18670	0.00224146	0.04439537	0.03748365	ACTA2/AGTR1	7
BP	GO:0048844	artery morpho	7/446	76/18670	0.00224146	0.04439537	0.03748365	APOB/FOXF1/	7

BP	GO:0002251	organ or tissu	5/446	39/18670	0.00224252	0.04439537	0.03748365	DEFA5/LTF/PI	5
BP	GO:0046677	response to a	17/446	327/18670	0.00235641	0.04630026	0.03909197	AOC1/ACTC1/	17
BP	GO:0017015	regulation of t	9/446	120/18670	0.00236134	0.04630026	0.03909197	CAV1/FBN1/L	9
BP	GO:0014831	gastro-intestir	3/446	12/18670	0.00253673	0.04871408	0.04112999	HTR2B/TACR2	3
BP	GO:0019374	galactolipid m	3/446	12/18670	0.00253673	0.04871408	0.04112999	UGT8/GBA3/I	3
BP	GO:0035630	bone minerali	3/446	12/18670	0.00253673	0.04871408	0.04112999	IGF1/LTF/GRE	3
BP	GO:0042340	keratan sulfat	3/446	12/18670	0.00253673	0.04871408	0.04112999	OMD/OGN/PI	3
BP	GO:0001558	regulation of	20/446	416/18670	0.00254388	0.04871408	0.04112999	AGTR1/CRYAE	20
BP	GO:0001666	response to h	18/446	359/18670	0.00262854	0.05010122	0.04230118	AQP1/CA9/CA	18
BP	GO:1903844	regulation of	9/446	122/18670	0.00264214	0.05012726	0.04232316	CAV1/FBN1/L	9
BP	GO:0060840	artery develo	8/446	100/18670	0.00272759	0.05107568	0.04312393	APOB/FOXF1/	8
BP	GO:1904385	cellular respo	4/446	25/18670	0.00272952	0.05107568	0.04312393	AGTR1/CA2/C	4
BP	GO:2000353	positive regul	4/446	25/18670	0.00272952	0.05107568	0.04312393	CCL2/AKR1C3	4
BP	GO:0097755	positive regul	6/446	59/18670	0.00277857	0.05175712	0.04369928	F2RL1/GUCY1	6
BP	GO:0001570	vasculogenesi	7/446	79/18670	0.0027964	0.0518202	0.04375254	CAV1/FOXF1/	7
BP	GO:0090279	regulation of	5/446	41/18670	0.00280724	0.0518202	0.04375254	PDGFRB/PLN/	5
BP	GO:0002027	regulation of l	8/446	101/18670	0.00290194	0.05332808	0.04502566	CASQ2/CAV1/	8
BP	GO:0007043	cell-cell juncti	9/446	124/18670	0.00294876	0.05394651	0.04554781	CAV1/CDH1/C	9
BP	GO:0061041	regulation of	10/446	148/18670	0.00298119	0.05429743	0.0458441	SERPING1/CA	10
BP	GO:0010038	response to m	18/446	364/18670	0.00304427	0.05520091	0.04660692	AOC1/APOBE	18
BP	GO:0045471	response to e	9/446	125/18670	0.00311225	0.05601786	0.04729668	ACTC1/APOBI	9
BP	GO:0070482	response to o	19/446	394/18670	0.0031182	0.05601786	0.04729668	AQP1/CA9/CA	19
BP	GO:0016049	cell growth	22/446	484/18670	0.00315197	0.05601786	0.04729668	AGTR1/CRYAE	22
BP	GO:0043567	regulation of i	4/446	26/18670	0.00316571	0.05601786	0.04729668	IGF1/IGFBP4/	4
BP	GO:0071711	basement me	4/446	26/18670	0.00316571	0.05601786	0.04729668	CMA1/COL4A	4
BP	GO:0030512	negative regu	7/446	81/18670	0.00322108	0.05601786	0.04729668	CAV1/FBN1/L	7
BP	GO:0010642	negative regu	3/446	13/18670	0.00323969	0.05601786	0.04729668	APOD/ADIPO	3
BP	GO:0038065	collagen-activ	3/446	13/18670	0.00323969	0.05601786	0.04729668	COL4A1/COL4	3
BP	GO:0061469	regulation of t	3/446	13/18670	0.00323969	0.05601786	0.04729668	REG1A/SFRP1	3
BP	GO:0070208	protein heter	3/446	13/18670	0.00323969	0.05601786	0.04729668	COL6A2/ADIP	3
BP	GO:0072182	regulation of	3/446	13/18670	0.00323969	0.05601786	0.04729668	PROM1/FAT4,	3
BP	GO:0045667	regulation of	9/446	126/18670	0.00328281	0.05618087	0.04743431	IGF1/CCN1/LT	9
BP	GO:0006027	glycosaminog	6/446	61/18670	0.00329024	0.05618087	0.04743431	BGN/HMMR/	6
BP	GO:0045123	cellular extrav	6/446	61/18670	0.00329024	0.05618087	0.04743431	ITGA1/CCL2/C	6
BP	GO:0051604	protein matur	19/446	397/18670	0.00338633	0.05758159	0.04861696	AEBP1/SERPIN	19
BP	GO:0014812	muscle cell mi	8/446	104/18670	0.00347752	0.05840529	0.04931242	IGF1/PDGFRB	8
BP	GO:0019233	sensory perce	8/446	104/18670	0.00347752	0.05840529	0.04931242	NDN/CCL2/CX	8
BP	GO:0030593	neutrophil ch	8/446	104/18670	0.00347752	0.05840529	0.04931242	CXADR/ITGA1	8
BP	GO:0072593	reactive oxyg	15/446	284/18670	0.00358745	0.05930885	0.05007531	AGTR1/ATP2E	15
BP	GO:0001933	negative regu	20/446	429/18670	0.00360048	0.05930885	0.05007531	CAV1/CCNB1/	20
BP	GO:0036293	response to d	18/446	370/18670	0.00361406	0.05930885	0.05007531	AQP1/CA9/CA	18
BP	GO:0002576	platelet degra	9/446	128/18670	0.00364595	0.05930885	0.05007531	SERPING1/CFI	9
BP	GO:0002687	positive regul	9/446	128/18670	0.00364595	0.05930885	0.05007531	F2RL1/CCL19/	9
BP	GO:0002026	regulation of t	4/446	27/18670	0.0036471	0.05930885	0.05007531	ATP1A2/ATP2	4
BP	GO:0045671	negative regu	4/446	27/18670	0.0036471	0.05930885	0.05007531	FBN1/LTF/SFF	4
BP	GO:0048799	animal organ	4/446	27/18670	0.0036471	0.05930885	0.05007531	IGF1/LTF/GRE	4
BP	GO:0006641	triglyceride m	8/446	105/18670	0.00368783	0.05935333	0.05011286	APOB/APOBE	8
BP	GO:1904018	positive regul	13/446	230/18670	0.00368851	0.05935333	0.05011286	AGTR1/ANXA	13
BP	GO:1903845	negative regu	7/446	83/18670	0.00369329	0.05935333	0.05011286	CAV1/FBN1/L	7
BP	GO:0003416	endochondral	5/446	44/18670	0.00383749	0.06095361	0.051464	COL6A2/COL6	5
BP	GO:0010463	mesenchymal	5/446	44/18670	0.00383749	0.06095361	0.051464	FOXF1/PRRX1	5
BP	GO:0044331	cell-cell adhes	5/446	44/18670	0.00383749	0.06095361	0.051464	CDH1/CDH5/C	5
BP	GO:0007052	mitotic spindl	8/446	106/18670	0.00390785	0.06169894	0.0520933	CCNB1/EML1,	8
BP	GO:1903034	regulation of	11/446	179/18670	0.00391898	0.06169894	0.0520933	SERPING1/CA	11
BP	GO:0003007	heart morpho	14/446	259/18670	0.00392958	0.06169894	0.0520933	ACTC1/CPE/D	14
BP	GO:0097756	negative regu	7/446	84/18670	0.00394825	0.06175549	0.05214105	ACTA2/AGTR1	7
BP	GO:0003231	cardiac ventri	9/446	130/18670	0.00403987	0.06193974	0.05229661	CPE/DSP/FOX	9
BP	GO:0003214	cardiac left ve	3/446	14/18670	0.00405072	0.06193974	0.05229661	CPE/FOXF1/SI	3
BP	GO:0035791	platelet-deriv	3/446	14/18670	0.00405072	0.06193974	0.05229661	PDGFRL/PDGI	3
BP	GO:0038166	angiotensin-a	3/446	14/18670	0.00405072	0.06193974	0.05229661	AGTR1/CA2/C	3
BP	GO:0042574	retinal metab	3/446	14/18670	0.00405072	0.06193974	0.05229661	CYP1B1/AKR1	3
BP	GO:0086069	bundle of His	3/446	14/18670	0.00405072	0.06193974	0.05229661	DSC2/DSG2/D	3
BP	GO:0048738	cardiac muscl	13/446	233/18670	0.00411406	0.06267444	0.05291692	ACTC1/CCNB1	13
BP	GO:1990776	response to a	4/446	28/18670	0.00417583	0.06337985	0.05351251	AGTR1/CA2/C	4
BP	GO:0048255	mRNA stabiliz	5/446	45/18670	0.00423408	0.06402684	0.05405878	APOBEC1/DA	5
BP	GO:0007204	positive regul	16/446	319/18670	0.00442058	0.0666012	0.05623234	AGTR1/ATP1A	16
BP	GO:0061138	morphogenes	11/446	182/18670	0.00444069	0.06665916	0.05628128	COL4A1/EDNI	11
BP	GO:0070542	response to fa	7/446	86/18670	0.00449798	0.06727273	0.05679933	APOB/CCNB1,	7
BP	GO:0006026	aminoglycan	6/446	65/18670	0.00451989	0.06735451	0.05686838	BGN/HMMR/	6

BP	GO:0051216	cartilage deve	12/446	209/18670	0.00457109	0.06762576	0.05709739	COL6A2/COL6	12
BP	GO:0071229	cellular respo	12/446	209/18670	0.00457109	0.06762576	0.05709739	APOB/AQP1/(	12
BP	GO:0071675	regulation of	5/446	46/18670	0.00465906	0.06867929	0.05798691	CXCL12/SLIT2	5
BP	GO:0007263	nitric oxide m	4/446	29/18670	0.00475397	0.0695777	0.05874545	ATP2B4/GUC	4
BP	GO:1900181	negative regu	4/446	29/18670	0.00475397	0.0695777	0.05874545	APOD/DCLK1/	4
BP	GO:0055074	calcium ion ho	21/446	471/18670	0.00481853	0.07027167	0.05933138	AGTR1/ATP1A	21
BP	GO:0072160	nephron tubu	3/446	15/18670	0.00497446	0.07203296	0.06081846	PROM1/FAT4	3
BP	GO:2000402	negative regu	3/446	15/18670	0.00497446	0.07203296	0.06081846	APOD/CCL2/A	3
BP	GO:0051181	cofactor trans	5/446	47/18670	0.0051135	0.07352669	0.06207964	CBLIF/LCN2/T	5
BP	GO:0098868	bone growth	5/446	47/18670	0.0051135	0.07352669	0.06207964	COL6A2/COL6	5
BP	GO:0006631	fatty acid met	18/446	383/18670	0.00515531	0.07383274	0.06233804	C3/CAV1/CES	18
BP	GO:0050921	positive regul	9/446	135/18670	0.00517081	0.07383274	0.06233804	F2RL1/PDGFR	9
BP	GO:0019748	secondary me	6/446	67/18670	0.00524808	0.07441735	0.06283163	CYP1B1/FMO	6
BP	GO:0048662	negative regu	6/446	67/18670	0.00524808	0.07441735	0.06283163	APOD/CNN1/	6
BP	GO:0090022	regulation of	4/446	30/18670	0.00538352	0.07607471	0.06423096	CCL19/THBS4	4
BP	GO:0097306	cellular respo	7/446	89/18670	0.00542843	0.07644567	0.06454417	CDH1/HMGCS	7
BP	GO:0002762	negative regu	5/446	48/18670	0.00559844	0.07830172	0.06611127	FBN1/LTF/SFF	5
BP	GO:0046850	regulation of	5/446	48/18670	0.00559844	0.07830172	0.06611127	CA2/SFRP1/G	5
BP	GO:0033627	cell adhesion	6/446	68/18670	0.00564276	0.07843588	0.06622453	COL16A1/CYP	6
BP	GO:0031960	response to co	10/446	162/18670	0.00564631	0.07843588	0.06622453	ANXA3/AQP1	10
BP	GO:0031214	biomineral tis	10/446	163/18670	0.00589119	0.08156108	0.06886318	GPM6B/IGF1/	10
BP	GO:0006957	complement a	3/446	16/18670	0.006015	0.08226383	0.06945653	C3/C7/CFD	3
BP	GO:0086103	G protein-cou	3/446	16/18670	0.006015	0.08226383	0.06945653	ATP2B4/CAV1	3
BP	GO:0060349	bone morpho	8/446	114/18670	0.00605303	0.08226383	0.06945653	COL6A2/COL6	8
BP	GO:0044319	wound healin	4/446	31/18670	0.00606644	0.08226383	0.06945653	FLNA/CCN1/I	4
BP	GO:0090075	relaxation of	4/446	31/18670	0.00606644	0.08226383	0.06945653	ATP1A2/GUC	4
BP	GO:0090505	epiboly involv	4/446	31/18670	0.00606644	0.08226383	0.06945653	FLNA/CCN1/I	4
BP	GO:0032496	response to li	16/446	330/18670	0.00608246	0.08226383	0.06945653	APOB/CTSG/D	16
BP	GO:0045638	negative regu	7/446	91/18670	0.00612427	0.08238938	0.06956253	FBN1/LTF/ME	7
BP	GO:0050920	regulation of	12/446	217/18670	0.00613196	0.08238938	0.06956253	F2RL1/PDGFR	12
BP	GO:0090130	tissue migrati	17/446	360/18670	0.00616856	0.08261036	0.06974911	ACTA2/ACTC1	17
BP	GO:0043687	post-translati	17/446	361/18670	0.00633559	0.08453556	0.07137458	APOB/C3/FBN	17
BP	GO:0019722	calcium-medi	12/446	218/18670	0.00635358	0.08453556	0.07137458	AGTR1/ATP1A	12
BP	GO:2000117	negative regu	7/446	92/18670	0.00649615	0.0861528	0.07274004	AQP1/CRYAB/	7
BP	GO:0007584	response to n	12/446	219/18670	0.00658148	0.08663706	0.07314891	BCHE/HMGCS	12
BP	GO:0048762	mesenchymal	12/446	219/18670	0.00658148	0.08663706	0.07314891	EDNRA/FN1/F	12
BP	GO:0032873	negative regu	5/446	50/18670	0.00666403	0.08663706	0.07314891	F2RL1/SFRP1/	5
BP	GO:0043489	RNA stabilizat	5/446	50/18670	0.00666403	0.08663706	0.07314891	APOBEC1/DA	5
BP	GO:0070303	negative regu	5/446	50/18670	0.00666403	0.08663706	0.07314891	F2RL1/SFRP1/	5
BP	GO:0086009	membrane re	5/446	50/18670	0.00666403	0.08663706	0.07314891	ATP1A2/CASC	5
BP	GO:0018108	peptidyl-tyros	17/446	363/18670	0.00668065	0.08663706	0.07314891	CAV1/FRK/GH	17
BP	GO:0090504	epiboly	4/446	32/18670	0.00680459	0.08796601	0.07427096	FLNA/CCN1/I	4
BP	GO:0052548	regulation of	19/446	425/18670	0.00694439	0.08949098	0.07555851	AQP1/SERPIN	19
BP	GO:0006883	cellular sodiu	3/446	17/18670	0.00717595	0.09020562	0.0761619	ATP1A2/C7/T	3
BP	GO:0031268	pseudopodiu	3/446	17/18670	0.00717595	0.09020562	0.0761619	F2RL1/CDC42	3
BP	GO:0035930	corticosteroid	3/446	17/18670	0.00717595	0.09020562	0.0761619	AGTR1/TAC1/	3
BP	GO:0035988	chondrocyte p	3/446	17/18670	0.00717595	0.09020562	0.0761619	LTF/DDR2/SIX	3
BP	GO:0046068	cGMP metabo	3/446	17/18670	0.00717595	0.09020562	0.0761619	GUCY1A1/GU	3
BP	GO:0061298	retina vascula	3/446	17/18670	0.00717595	0.09020562	0.0761619	COL4A1/CYP1	3
BP	GO:0061318	renal filtratior	3/446	17/18670	0.00717595	0.09020562	0.0761619	PROM1/ADIP	3
BP	GO:0072112	glomerular vis	3/446	17/18670	0.00717595	0.09020562	0.0761619	PROM1/ADIP	3
BP	GO:0018212	peptidyl-tyros	17/446	366/18670	0.00722664	0.09026482	0.07621188	CAV1/FRK/GH	17
BP	GO:0031103	axon regenera	5/446	51/18670	0.00724674	0.09026482	0.07621188	APOD/TNC/M	5
BP	GO:0045103	intermediate	5/446	51/18670	0.00724674	0.09026482	0.07621188	DES/DSP/SYN	5
BP	GO:2000377	regulation of	11/446	195/18670	0.00737155	0.09154129	0.07728962	AGTR1/ATP2E	11
BP	GO:1990266	neutrophil mi	8/446	118/18670	0.00741393	0.09178933	0.07749904	CXADR/ITGA1	8
BP	GO:0030307	positive regul	10/446	169/18670	0.00753912	0.09305816	0.07857034	FN1/SFN/IGF1	10
BP	GO:0001763	morphogenes	11/446	196/18670	0.00764781	0.09411624	0.07946369	COL4A1/EDNF	11
BP	GO:0001952	regulation of	8/446	119/18670	0.00778753	0.09534586	0.08050187	APOD/COL16	8
BP	GO:0035690	cellular respo	17/446	369/18670	0.00780809	0.09534586	0.08050187	AOC1/AQP1/	17
BP	GO:0007051	spindle organi	10/446	170/18670	0.00784527	0.09534586	0.08050187	CCNB1/EML1	10
BP	GO:0072132	mesenchyme	5/446	52/18670	0.00786406	0.09534586	0.08050187	ACTA2/ACTC1	5
BP	GO:1902373	negative regu	5/446	52/18670	0.00786406	0.09534586	0.08050187	APOBEC1/DA	5
BP	GO:0007015	actin filament	18/446	400/18670	0.00794509	0.09604421	0.0810915	ACTC1/CFL2/	18
BP	GO:0003205	cardiac chaml	10/446	171/18670	0.00816092	0.09824527	0.08294989	CPE/DSP/FOX	10
BP	GO:0003014	renal system	8/446	120/18670	0.00817512	0.09824527	0.08294989	AGTR1/AQP1	8
BP	GO:1904062	regulation of	16/446	342/18670	0.00843793	0.09934351	0.08387715	ANXA3/ATP1A	16
BP	GO:0003414	chondrocyte r	3/446	18/18670	0.00846044	0.09934351	0.08387715	COL6A2/COL6	3
BP	GO:0003429	growth plate	3/446	18/18670	0.00846044	0.09934351	0.08387715	COL6A2/COL6	3

BP	GO:0036035	osteoclast dev	3/446	18/18670	0.00846044	0.09934351	0.08387715	FBN1/LTF/CLD	3
BP	GO:0048557	embryonic dig	3/446	18/18670	0.00846044	0.09934351	0.08387715	FOXF1/SIX2/R	3
BP	GO:0072311	glomerular ep	3/446	18/18670	0.00846044	0.09934351	0.08387715	PROM1/ADIP(	3
BP	GO:0090026	positive regul	3/446	18/18670	0.00846044	0.09934351	0.08387715	CXCL12/S100/	3
BP	GO:0090171	chondrocyte r	3/446	18/18670	0.00846044	0.09934351	0.08387715	COL6A2/COL6	3
BP	GO:0051384	response to gl	9/446	146/18670	0.00851363	0.09943753	0.08395653	ANXA3/AQP1,	9
BP	GO:0050732	negative regu	5/446	53/18670	0.00851698	0.09943753	0.08395653	CAV1/SFRP1/!	5
BP	GO:1990868	response to cl	7/446	97/18670	0.00861232	0.09998098	0.08441537	REG1A/CCL2/	7
BP	GO:1990869	cellular respo	7/446	97/18670	0.00861232	0.09998098	0.08441537	REG1A/CCL2/	7
CC	GO:0062023	collagen-cont	59/470	406/19717	2.119E-29	8.031E-27	6.647E-27	AEBP1/AZGP1	59
CC	GO:0005604	basement me	18/470	95/19717	9.1668E-12	1.7371E-09	1.4377E-09	COL4A1/COL4	18
CC	GO:0043292	contractile fib	27/470	234/19717	1.3867E-11	1.7519E-09	1.45E-09	ACTA2/ACTC1	27
CC	GO:0044449	contractile fib	26/470	221/19717	2.1476E-11	2.0349E-09	1.6842E-09	ACTA2/ACTC1	26
CC	GO:0030016	myofibril	25/470	224/19717	1.6462E-10	1.2478E-08	1.0328E-08	ACTC1/ATP2B	25
CC	GO:0031674	I band	20/470	143/19717	2.0513E-10	1.2957E-08	1.0724E-08	ACTC1/ATP2B	20
CC	GO:0030018	Z disc	18/470	132/19717	2.5158E-09	1.3621E-07	1.1274E-07	ATP2B4/CASC	18
CC	GO:0030017	sarcomere	22/470	204/19717	3.9819E-09	1.8864E-07	1.5613E-07	ACTC1/ATP2B	22
CC	GO:0044420	extracellular r	11/470	51/19717	2.5327E-08	1.0665E-06	8.8273E-07	COL4A1/COL4	11
CC	GO:0005924	cell-substrate	30/470	408/19717	5.4213E-08	2.0547E-06	1.7006E-06	ACTC1/CAV1/	30
CC	GO:0030055	cell-substrate	30/470	412/19717	6.725E-08	2.3171E-06	1.9178E-06	ACTC1/CAV1/	30
CC	GO:0005788	endoplasmic r	25/470	309/19717	1.1684E-07	3.6902E-06	3.0542E-06	APOB/BCHE/(	25
CC	GO:0005925	focal adhesior	29/470	405/19717	1.5946E-07	4.6489E-06	3.8477E-06	ACTC1/CAV1/	29
CC	GO:0005911	cell-cell juncti	31/470	459/19717	2.1771E-07	5.8936E-06	4.8779E-06	AOC1/ATP1A2	31
CC	GO:0042383	sarcolemma	15/470	136/19717	9.0153E-07	2.2779E-05	1.8853E-05	AQP1/ATP1A2	15
CC	GO:0043296	apical junctior	15/470	143/19717	1.712E-06	4.0552E-05	3.3564E-05	AOC1/CDH1/(	15
CC	GO:0045177	apical part of	26/470	384/19717	1.9999E-06	4.4585E-05	3.6902E-05	AQP1/CA2/DS	26
CC	GO:0016324	apical plasma	23/470	318/19717	2.6063E-06	5.4877E-05	4.542E-05	AQP1/DSG2/F	23
CC	GO:0043034	costamere	6/470	19/19717	3.7037E-06	7.3878E-05	6.1146E-05	FLNC/PGM5/!	6
CC	GO:0005581	collagen trime	11/470	87/19717	6.885E-06	0.00013047	0.00010799	COL4A1/COL4	11
CC	GO:0005923	bicellular tigh	13/470	123/19717	7.7082E-06	0.00013912	0.00011514	AOC1/CDH5/(	13
CC	GO:0070160	tight junction	13/470	128/19717	1.193E-05	0.00020552	0.0001701	AOC1/CDH5/(	13
CC	GO:0005775	vacuolar lume	14/470	172/19717	6.747E-05	0.00111179	0.00092019	APOB/BGN/C	14
CC	GO:0032432	actin filament	9/470	75/19717	7.1698E-05	0.00113222	0.0009371	CRYAB/MYLK/	9
CC	GO:0005913	cell-cell adher	11/470	117/19717	0.00011249	0.00167315	0.0013848	CDH1/CDH5/(	11
CC	GO:0005901	caveola	9/470	80/19717	0.0001192	0.00167315	0.0013848	ATP1A2/ATP2	9
CC	GO:0016528	sarcoplasm	9/470	80/19717	0.0001192	0.00167315	0.0013848	CASQ2/DMPK	9
CC	GO:0005796	Golgi lumen	10/470	102/19717	0.00016187	0.00219103	0.00181343	BGN/DEFA5/H	10
CC	GO:0014704	intercalated d	7/470	50/19717	0.00017216	0.00225	0.00186224	ATP1A2/DES/	7
CC	GO:0045121	membrane ra	19/470	315/19717	0.00021681	0.00273905	0.00226701	ATP1A2/ATP2	19
CC	GO:0098857	membrane mi	19/470	316/19717	0.00022571	0.00275949	0.00228393	ATP1A2/ATP2	19
CC	GO:0030057	desmosome	5/470	25/19717	0.00026979	0.00312578	0.00258709	DSC2/DSG2/D	5
CC	GO:0044853	plasma memb	10/470	109/19717	0.00027948	0.00312578	0.00258709	ATP1A2/ATP2	10
CC	GO:0044291	cell-cell conta	8/470	71/19717	0.00028041	0.00312578	0.00258709	ATP1A2/DES/	8
CC	GO:0098589	membrane re	19/470	328/19717	0.0003602	0.00390049	0.00322829	ATP1A2/ATP2	19
CC	GO:0043202	lysosomal lum	9/470	95/19717	0.00044116	0.00464448	0.00384406	APOB/BGN/G	9
CC	GO:0060205	cytoplasmic v	19/470	338/19717	0.00052097	0.0053364	0.00441674	AOC1/APOB/!	19
CC	GO:0031983	vesicle lumen	19/470	339/19717	0.00054002	0.00538603	0.00445782	AOC1/APOB/!	19
CC	GO:0042641	actomyosin	8/470	79/19717	0.00058269	0.00566259	0.00468672	ACTC1/MYLK/	8
CC	GO:0034774	secretory grar	18/470	321/19717	0.00074527	0.00691329	0.00572187	AOC1/SERPIN	18
CC	GO:0016327	apicolateral p	4/470	18/19717	0.00074788	0.00691329	0.00572187	CLDN3/CXADI	4
CC	GO:0008305	integrin comp	5/470	31/19717	0.00076706	0.00692179	0.00572891	ITGA1/ITGA5/	5
CC	GO:0005902	microvillus	8/470	83/19717	0.0008111	0.0069881	0.00578379	CA2/CA9/CBL	8
CC	GO:0072562	blood micropa	11/470	147/19717	0.00081128	0.0069881	0.00578379	ACTC1/ACTG2	11
CC	GO:0043209	myelin sheath	6/470	49/19717	0.00104287	0.00854164	0.0070696	CA2/PLP1/TU	6
CC	GO:0001725	stress fiber	7/470	67/19717	0.00105925	0.00854164	0.0070696	MYLK/PGM5/	7
CC	GO:0097517	contractile act	7/470	67/19717	0.00105925	0.00854164	0.0070696	MYLK/PGM5/	7
CC	GO:0098636	protein compl	5/470	34/19717	0.00118462	0.00935359	0.00774162	ITGA1/ITGA5/	5
CC	GO:0005884	actin filament	9/470	111/19717	0.00135895	0.01051105	0.00869961	ACTC1/DPYSL	9
CC	GO:0001527	microfibril	3/470	10/19717	0.0014254	0.01080456	0.00894254	FBN1/LTBP1/I	3
CC	GO:0031091	platelet alpha	8/470	91/19717	0.00148188	0.01088351	0.00900788	SERPING1/CFI	8
CC	GO:0016529	sarcoplasmic	7/470	71/19717	0.00149325	0.01088351	0.00900788	CASQ2/DMPK	7
CC	GO:0016328	lateral plasma	6/470	57/19717	0.00230659	0.01649429	0.01365171	CDH1/CLDN3/	6
CC	GO:0005614	interstitial ma	3/470	12/19717	0.00252202	0.01770083	0.01465032	TNC/SMOC2/(	3
CC	GO:0035580	specific granu	6/470	62/19717	0.00353722	0.02437467	0.02017401	AOC1/FRK/LC	6
CC	GO:0042588	zymogen gran	3/470	14/19717	0.00402748	0.02677921	0.02216416	DMBT1/RAB2	3
CC	GO:0043218	compact myel	3/470	14/19717	0.00402748	0.02677921	0.02216416	MPDZ/PLLP/J/	3
CC	GO:0098858	actin-based ce	12/470	208/19717	0.00433946	0.0283561	0.02346929	ACTA2/ACTC1	12
CC	GO:0090533	cation-transp	3/470	15/19717	0.00494608	0.03177227	0.02629673	ATP1A2/FXYD	3
CC	GO:0031093	platelet alpha	6/470	67/19717	0.00519938	0.03284273	0.0271827	SERPING1/CFI	6

CC	GO:0031941	filamentous a	4/470	32/19717	0.00675705	0.04198234	0.03474722	DPYSL3/MYO:	4
CC	GO:0045178	basal part of c	5/470	51/19717	0.00718789	0.0439389	0.0363666	AQP1/ITGA1/	5
CC	GO:0051233	spindle midzo	4/470	34/19717	0.00839525	0.05050476	0.04180091	CDC6/EML1/C	4
CC	GO:0071682	endocytic vesi	3/470	20/19717	0.01134716	0.06719645	0.05561601	APOB/LTF/SP/	3
CC	GO:0098533	ATPase deper	3/470	21/19717	0.01300797	0.07584649	0.06277532	ATP1A2/FXYD	3
CC	GO:0033017	sarcoplasmic	4/470	39/19717	0.013568	0.07791321	0.06448587	CASQ2/DMPK	4
CC	GO:0016323	basolateral pl	11/470	217/19717	0.0153412	0.08678084	0.07182528	AQP1/ATP2B4	11
CC	GO:0043025	neuronal cell	20/470	497/19717	0.01641163	0.0914707	0.0757069	ANXA3/APOB,	20
CC	GO:0031528	microvillus me	3/470	23/19717	0.01672467	0.09186448	0.07603282	CA9/S100P/PI	3
MF	GO:0005201	extracellular r	38/432	163/17697	1.3485E-26	7.727E-24	6.8703E-24	AEBP1/BGN/C	38
MF	GO:0005539	glycosaminog	25/432	229/17697	4.1398E-10	1.1861E-07	1.0546E-07	AOC1/APOB/I	25
MF	GO:0008201	heparin bindi	20/432	169/17697	5.9377E-09	1.1341E-06	1.0084E-06	AOC1/APOB/C	20
MF	GO:0005178	integrin bindi	17/432	132/17697	2.3279E-08	3.3347E-06	2.965E-06	CDH17/COL16	17
MF	GO:0005518	collagen bindi	11/432	67/17697	6.0685E-07	6.9545E-05	6.1835E-05	AEBP1/FN1/IT	11
MF	GO:1901681	sulfur compou	21/432	250/17697	9.358E-07	8.9369E-05	7.9461E-05	AOC1/APOB/C	21
MF	GO:0097493	structural mol	5/432	11/17697	3.4662E-06	0.00028373	0.00025227	ELN/FBLN2/FI	5
MF	GO:0050839	cell adhesion	30/432	499/17697	5.7827E-06	0.00041419	0.00036827	CALD1/CDH1/	30
MF	GO:0003779	actin binding	27/432	431/17697	8.1338E-06	0.00051682	0.00045952	CALD1/CFL2/C	27
MF	GO:0050840	extracellular r	9/432	57/17697	9.0196E-06	0.00051682	0.00045952	BGN/ELN/FBL	9
MF	GO:0030020	extracellular r	7/432	41/17697	5.3964E-05	0.00281101	0.00249937	COL4A1/COL4	7
MF	GO:0098631	cell adhesion	8/432	59/17697	8.8058E-05	0.00420475	0.00373858	CXADR/DSC2/	8
MF	GO:0061134	peptidase reg	16/432	219/17697	9.9744E-05	0.00439641	0.00390899	SERPING1/C3,	16
MF	GO:0030021	extracellular r	5/432	22/17697	0.00015822	0.00647565	0.00575772	BGN/HSPG2/C	5
MF	GO:0086080	protein bindir	4/432	13/17697	0.00021018	0.00802904	0.0071389	CXADR/DSC2/	4
MF	GO:0001664	G protein-cou	17/432	280/17697	0.00054995	0.01969501	0.0175115	AGTR1/C3/FLI	17
MF	GO:0004181	metallocarbo:	5/432	29/17697	0.00061991	0.02089457	0.01857807	AEBP1/CPE/C	5
MF	GO:0048306	calcium-deper	7/432	61/17697	0.0006888	0.02192673	0.01949579	ANXA3/CASQ:	7
MF	GO:0016247	channel regul	11/432	144/17697	0.00082485	0.02399342	0.02133336	ATP2B4/CAV1	11
MF	GO:0008307	structural con	6/432	46/17697	0.00083747	0.02399342	0.02133336	CSRP1/MYH1:	6
MF	GO:0042379	chemokine re	7/432	66/17697	0.00110786	0.03022864	0.02687731	CCL2/CCL19/C	7
MF	GO:0098632	cell-cell adhes	6/432	50/17697	0.001309	0.03409343	0.03031362	CXADR/DSC2/	6
MF	GO:0019911	structural con	3/432	10/17697	0.00152545	0.03800362	0.0337903	PLP1/PLLP/M.	3
MF	GO:0017080	sodium chann	5/432	36/17697	0.00171082	0.04017694	0.03572267	ATP2B4/FXYD	5
MF	GO:0061135	endopeptidas	12/432	182/17697	0.00175292	0.04017694	0.03572267	SERPING1/C3,	12
MF	GO:0005324	long-chain fat	3/432	11/17697	0.00205968	0.04474311	0.03978261	FABP4/FABP1	3
MF	GO:0004867	serine-type er	8/432	94/17697	0.00210831	0.04474311	0.03978261	SERPING1/CO	8
MF	GO:0016504	peptidase acti	5/432	38/17697	0.00218879	0.04479197	0.03982605	CAV1/FBLN1/	5
MF	GO:0099106	ion channel re	9/432	118/17697	0.00242915	0.04799658	0.04267539	CAV1/FLNA/G	9
MF	GO:0005172	vascular endo	3/432	13/17697	0.00344276	0.05919216	0.05262975	CDH5/ITGA5/	3
MF	GO:0008494	translation ac	3/432	13/17697	0.00344276	0.05919216	0.05262975	DAZ1/DAZ2/D	3
MF	GO:0016725	oxidoreductas	3/432	13/17697	0.00344276	0.05919216	0.05262975	CYP3A5/RRM:	3
MF	GO:0005200	structural con	8/432	102/17697	0.00351094	0.05919216	0.05262975	DES/DSP/PLS:	8
MF	GO:0051015	actin filament	12/432	198/17697	0.00351227	0.05919216	0.05262975	CFL2/FLNA/M	12
MF	GO:0004866	endopeptidas	11/432	175/17697	0.00387356	0.06267601	0.05572736	SERPING1/C3,	11
MF	GO:0001968	fibronectin bi	4/432	27/17697	0.00393776	0.06267601	0.05572736	FBLN1/IGFBP(	4
MF	GO:0015926	glucosidase a	3/432	14/17697	0.00430298	0.06663805	0.05925015	SI/MGAM/GB	3
MF	GO:0005520	insulin-like gr	4/432	28/17697	0.00450682	0.06795805	0.0604238	IGFBP4/IGFBF	4
MF	GO:0004180	carboxypeptic	5/432	45/17697	0.00463534	0.06810383	0.06055342	AEBP1/CPE/C	5
MF	GO:0042805	actinin bindin	5/432	46/17697	0.00509849	0.07038875	0.06258502	CSRP1/PROM	5
MF	GO:0008236	serine-type pe	11/432	182/17697	0.00519545	0.07038875	0.06258502	C1R/C1S/CM/	11
MF	GO:0030414	peptidase inh	11/432	182/17697	0.00519545	0.07038875	0.06258502	SERPING1/C3,	11
MF	GO:0070492	oligosaccharic	3/432	15/17697	0.00528223	0.07038875	0.06258502	REG3A/REG1/	3
MF	GO:0004252	serine-type er	10/432	160/17697	0.00599401	0.07763866	0.06903116	C1R/C1S/CM/	10
MF	GO:0017171	serine hydrola	11/432	186/17697	0.00609728	0.07763866	0.06903116	C1R/C1S/CM/	11
MF	GO:0019838	growth factor	9/432	137/17697	0.00650893	0.0810786	0.07208973	COL4A1/LRRC	9
MF	GO:0008009	chemokine ac	5/432	49/17697	0.00668345	0.08148121	0.0724477	CCL2/CCL19/C	5

ONTOLOGY	ID	Descriptior	GeneRatio	BgRatio	pvalue	p.adjust	qvalue	geneID	Count
KEGG	hsa04512	ECM-recep	13/203	88/8076	2.49E-07	5.36E-05	4.75E-05	COL4A1/CC	13
KEGG	hsa04974	Protein dig	13/203	103/8076	1.6E-06	0.000172	0.000152	ATP1A2/CC	13
KEGG	hsa04510	Focal adhe:	18/203	201/8076	2.7E-06	0.000194	0.000172	CAV1/COL4	18
KEGG	hsa04270	Vascular sn	14/203	135/8076	6.74E-06	0.000362	0.000321	ACTA2/ACT	14
KEGG	hsa04022	cGMP-PKG	13/203	167/8076	0.000284	0.012208	0.010818	AGTR1/ATF	13
KEGG	hsa03320	PPAR signa	8/203	78/8076	0.00072	0.025808	0.02287	FABP4/FAB	8
KEGG	hsa04670	Leukocyte	9/203	114/8076	0.002228	0.055617	0.049286	CDH5/CLDI	9
KEGG	hsa04614	Renin-angi	4/203	23/8076	0.002358	0.055617	0.049286	AGTR1/CM	4
KEGG	hsa04964	Proximal tu	4/203	23/8076	0.002358	0.055617	0.049286	AQP1/ATP1	4
KEGG	hsa05150	Staphyloco	8/203	96/8076	0.002773	0.055617	0.049286	C1R/C1S/C	8
KEGG	hsa05133	Pertussis	7/203	76/8076	0.002882	0.055617	0.049286	SERPING1/	7
KEGG	hsa05412	Arrhythmo	7/203	77/8076	0.003104	0.055617	0.049286	DES/DSC2/	7
KEGG	hsa04610	Compleme	7/203	85/8076	0.005385	0.089066	0.078928	SERPING1/	7
