# Supplementary material for: Identification of GGT5 as a Novel Prognostic Biomarker for Gastric Cancer and its Correlation With Immune Cell Infiltration
Source: Front Genet. 2022 Mar 18;13:810292. doi: 10.3389/fgene.2022.810292 (PMC8971189; doi:10.3389/fgene.2022.810292)
Supplement: Supplementary file 13 [file DataSheet10.PDF]

ID	Description	setSize	enrichmentSc	NES	pvalue	p.adjust	qvalues	rank	leading_edge
REACTOME_(	REACTOME_(	459	0.55527566	2.53838135	0.0014245	0.01459531	0.00876805	5347	tags=44%, list=15%, signal=38%
REACTOME_S	REACTOME_S	459	0.37277965	1.70412099	0.0014245	0.01459531	0.00876805	6575	tags=31%, list=18%, signal=25%
WP_VEGFAVI	WP_VEGFAVI	431	0.39066106	1.77905281	0.00142857	0.01459531	0.00876805	5764	tags=23%, list=16%, signal=19%
REACTOME_I	REACTOME_I	409	0.51416387	2.32411493	0.00145773	0.01459531	0.00876805	6304	tags=36%, list=17%, signal=30%
REACTOME_(	REACTOME_(	401	0.44624401	2.01182511	0.00146199	0.01459531	0.00876805	5521	tags=35%, list=15%, signal=30%
KEGG_PATHV	KEGG_PATHV	325	0.49606396	2.19721913	0.00149477	0.01459531	0.00876805	5088	tags=28%, list=14%, signal=24%
REACTOME_(	REACTOME_(	327	0.56097684	2.48762683	0.00149477	0.01459531	0.00876805	5504	tags=48%, list=15%, signal=41%
REACTOME_I	REACTOME_I	327	0.34945193	1.54962903	0.00149477	0.01459531	0.00876805	5374	tags=22%, list=15%, signal=19%
NABA_SECRE	NABA_SECRE	342	0.45448835	2.02041322	0.0015015	0.01459531	0.00876805	5521	tags=41%, list=15%, signal=35%
WP_PI3KAKT	WP_PI3KAKT	339	0.52843088	2.34662609	0.0015015	0.01459531	0.00876805	6015	tags=37%, list=16%, signal=31%
REACTOME_I	REACTOME_I	300	0.65383056	2.86587731	0.00151976	0.01459531	0.00876805	4561	tags=56%, list=12%, signal=50%
REACTOME_I	REACTOME_I	305	0.63389364	2.78561619	0.00151976	0.01459531	0.00876805	5459	tags=49%, list=15%, signal=42%
WP_FOCAL_/	WP_FOCAL_/	303	0.57860982	2.54093496	0.00151976	0.01459531	0.00876805	5442	tags=38%, list=15%, signal=33%
KEGG_MAPK_	KEGG_MAPK_	267	0.45303573	1.96605878	0.00152905	0.01459531	0.00876805	5952	tags=25%, list=16%, signal=22%
REACTOME_I	REACTOME_I	292	0.35264015	1.54007633	0.00153139	0.01459531	0.00876805	5374	tags=23%, list=15%, signal=20%
KEGG_NEUR(	KEGG_NEUR(	270	0.54292353	2.35618058	0.00153374	0.01459531	0.00876805	6000	tags=47%, list=16%, signal=40%
KEGG_CYTOK	KEGG_CYTOK	263	0.57240122	2.47722975	0.0015361	0.01459531	0.00876805	6729	tags=54%, list=18%, signal=45%
NABA_CORE_	NABA_CORE_	274	0.72347988	3.1444332	0.00154083	0.01459531	0.00876805	4076	tags=59%, list=11%, signal=53%
WP_IL18_SIG	WP_IL18_SIG	273	0.46728559	2.02914882	0.00154083	0.01459531	0.00876805	6826	tags=34%, list=19%, signal=28%
REACTOME_I	REACTOME_I	205	0.67794211	2.86621153	0.00154321	0.01459531	0.00876805	4939	tags=46%, list=13%, signal=40%
REACTOME_I	REACTOME_I	205	0.44604154	1.88577961	0.00154321	0.01459531	0.00876805	6424	tags=33%, list=17%, signal=27%
REACTOME_T	REACTOME_T	269	0.44708101	1.93947267	0.00154321	0.01459531	0.00876805	6304	tags=31%, list=17%, signal=26%
REACTOME_I	REACTOME_I	259	0.46933753	2.02215048	0.0015456	0.01459531	0.00876805	4564	tags=31%, list=12%, signal=27%
NABA_ECM_I	NABA_ECM_I	238	0.4052582	1.73593617	0.00155039	0.01459531	0.00876805	5091	tags=39%, list=14%, signal=34%
REACTOME_(	REACTOME_(	215	0.51462161	2.18367229	0.00155039	0.01459531	0.00876805	4623	tags=34%, list=13%, signal=30%
REACTOME_/	REACTOME_/	222	0.66636358	2.83019156	0.00155521	0.01459531	0.00876805	5459	tags=57%, list=15%, signal=49%
WP_GPCRS_(	WP_GPCRS_(	256	0.57814612	2.48618352	0.00155521	0.01459531	0.00876805	5347	tags=44%, list=15%, signal=38%
KEGG_REGUI	KEGG_REGUI	213	0.52821343	2.23618991	0.00155763	0.01459531	0.00876805	6017	tags=34%, list=16%, signal=29%
WP_CIRCADI/	WP_CIRCADI/	200	0.38086461	1.60291298	0.00157233	0.01459531	0.00876805	4170	tags=18%, list=11%, signal=16%
REACTOME_I	REACTOME_I	245	0.41746751	1.78241515	0.00158983	0.01459531	0.00876805	4730	tags=27%, list=13%, signal=23%
KEGG_FOCAL	KEGG_FOCAL	199	0.6735092	2.82398548	0.00159744	0.01459531	0.00876805	5099	tags=45%, list=14%, signal=39%
WP_MAPK_S	WP_MAPK_S	246	0.46303437	1.97733725	0.00160256	0.01459531	0.00876805	6312	tags=26%, list=17%, signal=22%
WP_FOCAL_/	WP_FOCAL_/	198	0.65205178	2.73174113	0.00160514	0.01459531	0.00876805	5099	tags=42%, list=14%, signal=37%
WP_RAS_SIG	WP_RAS_SIG	184	0.40911386	1.70941691	0.00161031	0.01459531	0.00876805	5374	tags=28%, list=15%, signal=24%
REACTOME_(	REACTOME_(	194	0.62749128	2.62391425	0.00162075	0.01459531	0.00876805	5635	tags=56%, list=15%, signal=47%
KEGG_CHEM	KEGG_CHEM	188	0.59896686	2.49668038	0.00162866	0.01459531	0.00876805	5686	tags=42%, list=15%, signal=36%
NABA_ECM_(	NABA_ECM_(	195	0.70762909	2.95773183	0.00162866	0.01459531	0.00876805	3972	tags=55%, list=11%, signal=50%
REACTOME_I	REACTOME_I	195	0.55872551	2.33534807	0.00162866	0.01459531	0.00876805	5504	tags=49%, list=15%, signal=42%
KEGG_CALCIU	KEGG_CALCIU	177	0.58981372	2.44551553	0.00163132	0.01459531	0.00876805	5185	tags=44%, list=14%, signal=38%
REACTOME_I	REACTOME_I	186	0.57488241	2.39585826	0.00163399	0.01459531	0.00876805	5571	tags=39%, list=15%, signal=33%
REACTOME_I	REACTOME_I	186	0.78263121	3.26166432	0.00163399	0.01459531	0.00876805	5633	tags=79%, list=15%, signal=67%
KEGG_TIGHT_	KEGG_TIGHT_	131	0.43069693	1.72346452	0.00164204	0.01459531	0.00876805	4907	tags=21%, list=13%, signal=19%
REACTOME_I	REACTOME_I	131	0.47072655	1.8836459	0.00164204	0.01459531	0.00876805	3798	tags=33%, list=10%, signal=30%
REACTOME_I	REACTOME_I	140	0.63491665	2.56549531	0.00164474	0.01459531	0.00876805	4272	tags=53%, list=12%, signal=47%
KEGG_CELL_/	KEGG_CELL_/	130	0.66442975	2.6535046	0.00164745	0.01459531	0.00876805	5996	tags=59%, list=16%, signal=50%
WP_ADIPOGI	WP_ADIPOGI	130	0.59738623	2.38575576	0.00164745	0.01459531	0.00876805	6877	tags=44%, list=19%, signal=36%
KEGG_AXON_	KEGG_AXON_	129	0.54295078	2.16421795	0.00165289	0.01459531	0.00876805	6250	tags=37%, list=17%, signal=31%
PID_PDGFRB_	PID_PDGFRB_	129	0.46396249	1.84936826	0.00165289	0.01459531	0.00876805	6180	tags=25%, list=17%, signal=21%
REACTOME_/	REACTOME_/	133	0.57707606	2.31321177	0.00165289	0.01459531	0.00876805	5340	tags=44%, list=14%, signal=37%
REACTOME_I	REACTOME_I	139	0.47904175	1.92926701	0.00165289	0.01459531	0.00876805	7000	tags=39%, list=19%, signal=32%
WP_EBOLA_\	WP_EBOLA_\	129	0.54720327	2.18116851	0.00165289	0.01459531	0.00876805	6186	tags=34%, list=17%, signal=28%
KEGG_NATUI	KEGG_NATUI	132	0.46170083	1.84865283	0.00165563	0.01459531	0.00876805	7363	tags=40%, list=20%, signal=32%
REACTOME_(	REACTOME_(	137	0.66349802	2.66813596	0.00165837	0.01459531	0.00876805	4009	tags=42%, list=11%, signal=38%
REACTOME_S	REACTOME_S	166	0.62061217	2.54817464	0.00166113	0.01459531	0.00876805	5571	tags=43%, list=15%, signal=37%
REACTOME_I	REACTOME_I	143	0.70061763	2.82786373	0.00166667	0.01459531	0.00876805	5571	tags=56%, list=15%, signal=48%
REACTOME_S	REACTOME_S	134	0.42114539	1.6856032	0.00166667	0.01459531	0.00876805	6180	tags=27%, list=17%, signal=22%
REACTOME_(	REACTOME_(	124	0.53600426	2.12286262	0.00166945	0.01459531	0.00876805	6641	tags=40%, list=18%, signal=33%
REACTOME_I	REACTOME_I	142	0.53319243	2.14678098	0.00167504	0.01459531	0.00876805	4232	tags=33%, list=11%, signal=29%
REACTOME_I	REACTOME_I	136	0.61331758	2.45792696	0.00167504	0.01459531	0.00876805	5459	tags=43%, list=15%, signal=37%
WP_BRAINDI	WP_BRAINDI	144	0.50718911	2.0481457	0.00167504	0.01459531	0.00876805	5571	tags=26%, list=15%, signal=22%
WP_REGULAT	WP_REGULAT	142	0.47371216	1.90729686	0.00167504	0.01459531	0.00876805	8367	tags=38%, list=23%, signal=30%
WP_SUDDEN	WP_SUDDEN	160	0.44530376	1.81914043	0.00167504	0.01459531	0.00876805	4544	tags=26%, list=12%, signal=23%
WP_ECTODEI	WP_ECTODEI	141	0.49739755	2.00048036	0.00167785	0.01459531	0.00876805	5164	tags=29%, list=14%, signal=25%
WP_BREAST_	WP_BREAST_	154	0.41817723	1.7019018	0.00168067	0.01459531	0.00876805	5571	tags=24%, list=15%, signal=20%
WP_EPITHELI	WP_EPITHELI	159	0.44119074	1.80028654	0.00168067	0.01459531	0.00876805	4961	tags=30%, list=13%, signal=26%
REACTOME_I	REACTOME_I	121	0.55825098	2.20084797	0.0016835	0.01459531	0.00876805	5613	tags=34%, list=15%, signal=29%
WP_CHEMOI	WP_CHEMOI	164	0.60685001	2.48211657	0.0016835	0.01459531	0.00876805	5571	tags=41%, list=15%, signal=35%
WP_HIPPOM	WP_HIPPOM	121	0.49762037	1.96181792	0.0016835	0.01459531	0.00876805	5635	tags=38%, list=15%, signal=32%
WP_MYOMET	WP_MYOMET	156	0.57461106	2.34168743	0.0016835	0.01459531	0.00876805	5185	tags=36%, list=14%, signal=31%
KEGG_JAK_ST	KEGG_JAK_ST	155	0.4709381	1.91776929	0.00168634	0.01459531	0.00876805	7511	tags=42%, list=20%, signal=34%
REACTOME_S	REACTOME_S	106	0.51322306	1.97217771	0.00169205	0.01459531	0.00876805	5571	tags=31%, list=15%, signal=27%
WP_OSTEOB	WP_OSTEOB	118	0.5411063	2.12292588	0.00169205	0.01459531	0.00876805	6113	tags=37%, list=17%, signal=31%
WP_SPINAL_	WP_SPINAL_	118	0.58332675	2.28856967	0.00169205	0.01459531	0.00876805	6035	tags=42%, list=16%, signal=35%

KEGG_T_CELI	KEGG_T_CELI	108	0.50877083	1.96449141	0.00169492	0.01459531	0.00876805	8869	tags=44%, list=24%, signal=34%
REACTOME_I	REACTOME_I	108	0.53431156	2.06311058	0.00169492	0.01459531	0.00876805	5236	tags=31%, list=14%, signal=26%
REACTOME_I	REACTOME_I	116	0.73726178	2.8809991	0.00169492	0.01459531	0.00876805	5459	tags=62%, list=15%, signal=53%
WP_ESC_PLU	WP_ESC_PLU	116	0.49492035	1.93400109	0.00169492	0.01459531	0.00876805	5343	tags=27%, list=14%, signal=23%
KEGG_WNT_	KEGG_WNT_	150	0.42567369	1.72501441	0.00170068	0.01459531	0.00876805	5185	tags=21%, list=14%, signal=18%
REACTOME_I	REACTOME_I	111	0.56441575	2.18440563	0.00170068	0.01459531	0.00876805	6996	tags=51%, list=19%, signal=42%
REACTOME_S	REACTOME_S	150	0.40963091	1.66000212	0.00170068	0.01459531	0.00876805	4110	tags=17%, list=11%, signal=15%
WP_CALCIUN	WP_CALCIUN	150	0.58671311	2.37761596	0.00170068	0.01459531	0.00876805	4828	tags=36%, list=13%, signal=31%
WP_REGULAT	WP_REGULAT	150	0.48746327	1.97541257	0.00170068	0.01459531	0.00876805	5952	tags=31%, list=16%, signal=26%
WP_SENESCE	WP_SENESCE	105	0.50157838	1.92235511	0.00170648	0.01459531	0.00876805	3932	tags=23%, list=11%, signal=20%
WP_WNT_SI(	WP_WNT_SI(	113	0.47615174	1.84385349	0.00170648	0.01459531	0.00876805	5091	tags=27%, list=14%, signal=23%
KEGG_LEUKC	KEGG_LEUKC	115	0.5514595	2.1487153	0.0017094	0.01459531	0.00876805	5571	tags=41%, list=15%, signal=35%
REACTOME_(	REACTOME_(	72	0.62983895	2.26159761	0.0017094	0.01459531	0.00876805	6186	tags=46%, list=17%, signal=38%
WP_TOLLLIKE	WP_TOLLLIKE	103	0.49844394	1.90697703	0.0017094	0.01459531	0.00876805	6751	tags=34%, list=18%, signal=28%
REACTOME_I	REACTOME_I	69	0.82578693	2.92583915	0.00171821	0.01459531	0.00876805	5161	tags=88%, list=14%, signal=76%
REACTOME_S	REACTOME_S	69	0.71591854	2.53656532	0.00171821	0.01459531	0.00876805	5161	tags=75%, list=14%, signal=65%
KEGG_HEMA	KEGG_HEMA	84	0.70010775	2.58755641	0.00172117	0.01459531	0.00876805	6985	tags=69%, list=19%, signal=56%
KEGG_VASCU	KEGG_VASCU	114	0.58680711	2.27556225	0.00172117	0.01459531	0.00876805	4110	tags=35%, list=11%, signal=31%
REACTOME_(	REACTOME_(	114	0.66399288	2.57487871	0.00172117	0.01459531	0.00876805	5459	tags=70%, list=15%, signal=60%
REACTOME_(	REACTOME_(	71	0.77236387	2.75611972	0.00172117	0.01459531	0.00876805	5161	tags=85%, list=14%, signal=73%
REACTOME_I	REACTOME_I	84	0.66855041	2.47092236	0.00172117	0.01459531	0.00876805	5120	tags=70%, list=14%, signal=61%
REACTOME_I	REACTOME_I	71	0.80233138	2.86305645	0.00172117	0.01459531	0.00876805	5571	tags=82%, list=15%, signal=69%
REACTOME_T	REACTOME_T	84	0.50527649	1.86747173	0.00172117	0.01459531	0.00876805	2937	tags=14%, list=8%, signal=13%
WP_EGFR_TY	WP_EGFR_TY	84	0.5174584	1.91249532	0.00172117	0.01459531	0.00876805	4373	tags=27%, list=12%, signal=24%
KEGG_ARRHY	KEGG_ARRHY	74	0.67758686	2.43744579	0.00172414	0.01459531	0.00876805	4976	tags=47%, list=13%, signal=41%
KEGG_VIRAL_	KEGG_VIRAL_	68	0.58610735	2.06769354	0.00172414	0.01459531	0.00876805	6958	tags=49%, list=19%, signal=39%
PID_AVB3_IN	PID_AVB3_IN	74	0.65948683	2.37233554	0.00172414	0.01459531	0.00876805	5120	tags=53%, list=14%, signal=45%
WP_ARRHYTI	WP_ARRHYTI	74	0.67758686	2.43744579	0.00172414	0.01459531	0.00876805	4976	tags=47%, list=13%, signal=41%
KEGG_ADIPO	KEGG_ADIPO	67	0.55108222	1.94070624	0.00172712	0.01459531	0.00876805	4105	tags=22%, list=11%, signal=20%
KEGG_TOLL_	KEGG_TOLL_	102	0.51447839	1.95773011	0.00172712	0.01459531	0.00876805	6751	tags=35%, list=18%, signal=29%
REACTOME_(	REACTOME_(	67	0.73138663	2.57567119	0.00172712	0.01459531	0.00876805	5120	tags=69%, list=14%, signal=59%
REACTOME_I	REACTOME_I	102	0.57134368	2.174118	0.00172712	0.01459531	0.00876805	6553	tags=49%, list=18%, signal=40%
WP_INTEGRI	WP_INTEGRI	102	0.584752	2.2251403	0.00172712	0.01459531	0.00876805	5403	tags=34%, list=15%, signal=29%
KEGG_B_CEL	KEGG_B_CEL	75	0.5373734	1.93466959	0.0017301	0.01459531	0.00876805	7363	tags=36%, list=20%, signal=29%
REACTOME_/	REACTOME_/	86	0.80918924	3.00737459	0.0017301	0.01459531	0.00876805	5571	tags=78%, list=15%, signal=66%
REACTOME_I	REACTOME_I	86	0.78156535	2.90470962	0.0017301	0.01459531	0.00876805	5516	tags=74%, list=15%, signal=63%
WP_PEPTIDE	WP_PEPTIDE	75	0.70708463	2.54566963	0.0017301	0.01459531	0.00876805	4673	tags=61%, list=13%, signal=54%
WP_VIRAL_A	WP_VIRAL_A	86	0.55456407	2.06105296	0.0017301	0.01459531	0.00876805	6575	tags=40%, list=18%, signal=33%
KEGG_TGF_B	KEGG_TGF_B	85	0.55227111	2.04109033	0.0017331	0.01459531	0.00876805	3244	tags=26%, list=9%, signal=24%
PID_CXCR4_F	PID_CXCR4_F	100	0.66256399	2.51533956	0.0017331	0.01459531	0.00876805	5923	tags=42%, list=16%, signal=35%
REACTOME_I	REACTOME_I	95	0.79343896	2.99558996	0.0017331	0.01459531	0.00876805	5185	tags=75%, list=14%, signal=64%
REACTOME_I	REACTOME_I	85	0.63297028	2.33933932	0.0017331	0.01459531	0.00876805	4478	tags=42%, list=12%, signal=37%
KEGG_LEISHI	KEGG_LEISHI	70	0.66420992	2.35575532	0.00173611	0.01459531	0.00876805	6958	tags=51%, list=19%, signal=42%
PID_AP1_PAT	PID_AP1_PAT	70	0.55369972	1.96380845	0.00173611	0.01459531	0.00876805	6019	tags=41%, list=16%, signal=35%
PID_LYSOPH(	PID_LYSOPH(	65	0.6043951	2.11836048	0.00173913	0.01459531	0.00876805	8170	tags=46%, list=22%, signal=36%
REACTOME_I	REACTOME_I	87	0.76376475	2.83787424	0.00173913	0.01459531	0.00876805	5459	tags=69%, list=15%, signal=59%
REACTOME_I	REACTOME_I	87	0.60953358	2.26480685	0.00173913	0.01459531	0.00876805	4250	tags=33%, list=12%, signal=30%
WP_GPCRS_(	WP_GPCRS_(	92	0.51402687	1.93385046	0.00173913	0.01459531	0.00876805	4340	tags=37%, list=12%, signal=33%
WP_ONCOST	WP_ONCOST	65	0.54623646	1.91451872	0.00173913	0.01459531	0.00876805	6874	tags=32%, list=19%, signal=26%
WP_B_CELL_	WP_B_CELL_	97	0.5389031	2.04043698	0.00174216	0.01459531	0.00876805	7513	tags=38%, list=20%, signal=30%
WP_HUMAN_	WP_HUMAN_	97	0.49937676	1.89077925	0.00174216	0.01459531	0.00876805	4816	tags=45%, list=13%, signal=40%
REACTOME_I	REACTOME_I	98	0.693158	2.61871068	0.0017452	0.01459531	0.00876805	5161	tags=70%, list=14%, signal=61%
WP_MICROR	WP_MICROR	98	0.5379658	2.03240354	0.0017452	0.01459531	0.00876805	5704	tags=31%, list=15%, signal=26%
WP_PATHWA	WP_PATHWA	98	0.48463969	1.83094062	0.0017452	0.01459531	0.00876805	5357	tags=33%, list=15%, signal=28%
WP_PHOSPH	WP_PHOSPH	53	0.66682431	2.24986381	0.0017452	0.01459531	0.00876805	4110	tags=47%, list=11%, signal=42%
PID_INTEGRI	PID_INTEGRI	66	0.65929545	2.31227185	0.00174825	0.01459531	0.00876805	3692	tags=62%, list=10%, signal=56%
REACTOME_I	REACTOME_I	79	0.76880143	2.78668658	0.00174825	0.01459531	0.00876805	5161	tags=84%, list=14%, signal=72%
REACTOME_I	REACTOME_I	78	0.54343407	1.9604309	0.00174825	0.01459531	0.00876805	5236	tags=32%, list=14%, signal=28%
REACTOME_I	REACTOME_I	82	0.78967994	2.89029481	0.00174825	0.01459531	0.00876805	5571	tags=79%, list=15%, signal=67%
ST_INTEGRIN	ST_INTEGRIN	82	0.56388185	2.06385486	0.00174825	0.01459531	0.00876805	3350	tags=26%, list=9%, signal=23%
WP_REGULAT	WP_REGULAT	78	0.60722124	2.19054224	0.00174825	0.01459531	0.00876805	4587	tags=40%, list=12%, signal=35%
KEGG_DILATI	KEGG_DILATI	90	0.70961113	2.64823138	0.00175131	0.01459531	0.00876805	4561	tags=46%, list=12%, signal=40%
KEGG_GAP_J	KEGG_GAP_J	90	0.53935924	2.01286029	0.00175131	0.01459531	0.00876805	5236	tags=31%, list=14%, signal=27%
REACTOME_(	REACTOME_(	50	0.62583649	2.0975605	0.00175131	0.01459531	0.00876805	6641	tags=52%, list=18%, signal=43%
REACTOME_(	REACTOME_(	90	0.72005276	2.68719899	0.00175131	0.01459531	0.00876805	5120	tags=63%, list=14%, signal=55%
REACTOME_I	REACTOME_I	50	0.59477293	1.99344754	0.00175131	0.01459531	0.00876805	6057	tags=42%, list=16%, signal=35%
REACTOME_I	REACTOME_I	59	0.69010293	2.37447576	0.00175131	0.01459531	0.00876805	4561	tags=61%, list=12%, signal=54%
REACTOME_(	REACTOME_(	90	0.5330344	1.98925632	0.00175131	0.01459531	0.00876805	5236	tags=33%, list=14%, signal=29%
REACTOME_I	REACTOME_I	90	0.49420467	1.84434579	0.00175131	0.01459531	0.00876805	4838	tags=31%, list=13%, signal=27%
WP_ALLOGR/	WP_ALLOGR/	89	0.65163724	2.43057788	0.00175131	0.01459531	0.00876805	6015	tags=53%, list=16%, signal=44%
WP_TCELL_A	WP_TCELL_A	90	0.56496534	2.10842092	0.00175131	0.01459531	0.00876805	7059	tags=40%, list=19%, signal=32%
WP_TGFBET/	WP_TGFBET/	59	0.60765276	2.09078485	0.00175131	0.01459531	0.00876805	7401	tags=49%, list=20%, signal=39%
KEGG_ECM_I	KEGG_ECM_I	83	0.7460345	2.74164753	0.00175439	0.01459531	0.00876805	4976	tags=65%, list=13%, signal=56%
KEGG_HYPER	KEGG_HYPER	83	0.71106586	2.61313914	0.00175439	0.01459531	0.00876805	4561	tags=45%, list=12%, signal=39%

PID_IL12_2P/	PID_IL12_2P/	62	0.63222668	2.1971459	0.00175439	0.01459531	0.00876805	8606	tags=56%, list=23%, signal=43%
WP_TCELL_A	WP_TCELL_A	62	0.56019167	1.9468062	0.00175439	0.01459531	0.00876805	8471	tags=50%, list=23%, signal=39%
KEGG_HEDGI	KEGG_HEDGI	56	0.57200288	1.95221037	0.00175747	0.01459531	0.00876805	4079	tags=30%, list=11%, signal=27%
PID_TXA2PAT	PID_TXA2PAT	56	0.63310722	2.16075571	0.00175747	0.01459531	0.00876805	6098	tags=46%, list=17%, signal=39%
REACTOME_/	REACTOME_/	61	0.74219368	2.57137587	0.00175747	0.01459531	0.00876805	4272	tags=64%, list=12%, signal=57%
REACTOME_(	REACTOME_(	61	0.83462642	2.89161479	0.00175747	0.01459531	0.00876805	5161	tags=90%, list=14%, signal=78%
REACTOME_I	REACTOME_I	76	0.73089062	2.62417708	0.00175747	0.01459531	0.00876805	4260	tags=64%, list=12%, signal=57%
REACTOME_I	REACTOME_I	56	0.58430535	1.99419793	0.00175747	0.01459531	0.00876805	4250	tags=34%, list=12%, signal=30%
WP_HEMATC	WP_HEMATC	61	0.55462406	1.92152932	0.00175747	0.01459531	0.00876805	6379	tags=48%, list=17%, signal=39%
WP_TYROBP_	WP_TYROBP_	61	0.66054267	2.28849089	0.00175747	0.01459531	0.00876805	6239	tags=62%, list=17%, signal=52%
PID_SHP2_P/	PID_SHP2_P/	57	0.58127223	1.98753337	0.00176056	0.01459531	0.00876805	8058	tags=49%, list=22%, signal=38%
REACTOME_(	REACTOME_(	57	0.66326564	2.26789192	0.00176056	0.01459531	0.00876805	5686	tags=68%, list=15%, signal=58%
REACTOME_(	REACTOME_(	94	0.63535185	2.38794617	0.00176056	0.01459531	0.00876805	4336	tags=44%, list=12%, signal=39%
PID_ARF6_TF	PID_ARF6_TF	49	0.67715062	2.25977515	0.00176367	0.01459531	0.00876805	4976	tags=41%, list=13%, signal=35%
PID_FCER1_F	PID_FCER1_F	60	0.54249412	1.87046812	0.00176367	0.01459531	0.00876805	7363	tags=42%, list=20%, signal=33%
WP_IL3_SIGN	WP_IL3_SIGN	49	0.55773178	1.8612527	0.00176367	0.01459531	0.00876805	8495	tags=47%, list=23%, signal=36%
KEGG_BASAL	KEGG_BASAL	55	0.58335242	1.97394891	0.00176678	0.01459531	0.00876805	4079	tags=35%, list=11%, signal=31%
REACTOME_(	REACTOME_(	48	0.58358854	1.93849951	0.00176678	0.01459531	0.00876805	8320	tags=56%, list=23%, signal=44%
REACTOME_(	REACTOME_(	54	0.62026675	2.09648339	0.00176678	0.01459531	0.00876805	5236	tags=39%, list=14%, signal=33%
REACTOME_I	REACTOME_I	54	0.6857531	2.31782531	0.00176678	0.01459531	0.00876805	2999	tags=43%, list=8%, signal=39%
REACTOME_S	REACTOME_S	54	0.53671529	1.81408192	0.00176678	0.01459531	0.00876805	3597	tags=26%, list=10%, signal=23%
WP_DIFFERE	WP_DIFFERE	48	0.65305634	2.1692499	0.00176678	0.01459531	0.00876805	4438	tags=50%, list=12%, signal=44%
WP_G_PROT	WP_G_PROT	93	0.59871916	2.2445509	0.00176678	0.01459531	0.00876805	5236	tags=35%, list=14%, signal=31%
WP_IL4_SIGN	WP_IL4_SIGN	54	0.58304216	1.97066537	0.00176678	0.01459531	0.00876805	9009	tags=50%, list=24%, signal=38%
WP_TGFBET/	WP_TGFBET/	55	0.57317075	1.93949616	0.00176678	0.01459531	0.00876805	7401	tags=45%, list=20%, signal=36%
PID_BCR_5P/	PID_BCR_5P/	63	0.5446101	1.89275401	0.00177305	0.01459531	0.00876805	9009	tags=43%, list=24%, signal=32%
PID_ENDOTH	PID_ENDOTH	63	0.64295633	2.23454943	0.00177305	0.01459531	0.00876805	5952	tags=40%, list=16%, signal=33%
PID_IL4_2PAT	PID_IL4_2PAT	64	0.65236343	2.2683695	0.00177305	0.01459531	0.00876805	6877	tags=53%, list=19%, signal=43%
PID_TCR_PAT	PID_TCR_PAT	63	0.64531729	2.24275475	0.00177305	0.01459531	0.00876805	7059	tags=46%, list=19%, signal=37%
REACTOME_(	REACTOME_(	64	0.73549166	2.55741932	0.00177305	0.01459531	0.00876805	4202	tags=66%, list=11%, signal=58%
REACTOME_I	REACTOME_I	63	0.69799402	2.42582905	0.00177305	0.01459531	0.00876805	3409	tags=43%, list=9%, signal=39%
REACTOME_S	REACTOME_S	64	0.64377731	2.2385142	0.00177305	0.01459531	0.00876805	5880	tags=47%, list=16%, signal=39%
WP_ENDOCH	WP_ENDOCH	64	0.68564008	2.3840776	0.00177305	0.01459531	0.00876805	6113	tags=61%, list=17%, signal=51%
WP_ENDOCH	WP_ENDOCH	64	0.68564008	2.3840776	0.00177305	0.01459531	0.00876805	6113	tags=61%, list=17%, signal=51%
WP_LUNG_FI	WP_LUNG_FI	63	0.63590215	2.21003312	0.00177305	0.01459531	0.00876805	7004	tags=59%, list=19%, signal=48%
REACTOME_S	REACTOME_S	58	0.64244625	2.19687719	0.0017762	0.01459531	0.00876805	5879	tags=48%, list=16%, signal=41%
BIOCARTA_N	BIOCARTA_N	51	0.64230807	2.14107131	0.00177936	0.01459531	0.00876805	5571	tags=39%, list=15%, signal=33%
KEGG_MTOR	KEGG_MTOR	52	0.54112372	1.81154687	0.00178571	0.01459531	0.00876805	4697	tags=25%, list=13%, signal=22%
PID_CD8_TCF	PID_CD8_TCF	52	0.62740246	2.10038651	0.00178571	0.01459531	0.00876805	6462	tags=40%, list=18%, signal=33%
WP_NETRINU	WP_NETRINU	52	0.63650266	2.13085171	0.00178571	0.01459531	0.00876805	7348	tags=44%, list=20%, signal=35%
KEGG_ALLOG	KEGG_ALLOG	35	0.70088946	2.20472058	0.00181488	0.01459531	0.00876805	8358	tags=74%, list=23%, signal=57%
KEGG_PRIM/	KEGG_PRIM/	35	0.63534938	1.99855744	0.00181488	0.01459531	0.00876805	5782	tags=51%, list=16%, signal=43%
NABA_PROTE	NABA_PROTE	35	0.76313945	2.40053439	0.00181488	0.01459531	0.00876805	4057	tags=63%, list=11%, signal=56%
SIG_REGULAT	SIG_REGULAT	35	0.69325278	2.1806986	0.00181488	0.01459531	0.00876805	2226	tags=20%, list=6%, signal=19%
KEGG_INTEST	KEGG_INTEST	46	0.69947874	2.2915323	0.00182149	0.01459531	0.00876805	6015	tags=67%, list=16%, signal=56%
PID_SYNDEC/	PID_SYNDEC/	46	0.74007114	2.42451534	0.00182149	0.01459531	0.00876805	3409	tags=61%, list=9%, signal=55%
SIG_BCR_SIG	SIG_BCR_SIG	46	0.62359333	2.04292739	0.00182149	0.01459531	0.00876805	7363	tags=41%, list=20%, signal=33%
PID_AMB2_N	PID_AMB2_N	41	0.64532864	2.07392059	0.00182815	0.01459531	0.00876805	5091	tags=51%, list=14%, signal=44%
REACTOME_I	REACTOME_I	41	0.63590818	2.04364566	0.00182815	0.01459531	0.00876805	4110	tags=34%, list=11%, signal=30%
REACTOME_I	REACTOME_I	41	0.74912465	2.40749433	0.00182815	0.01459531	0.00876805	4075	tags=44%, list=11%, signal=39%
REACTOME_I	REACTOME_I	41	0.63736384	2.04832377	0.00182815	0.01459531	0.00876805	4260	tags=39%, list=12%, signal=35%
REACTOME_I	REACTOME_I	38	0.78342469	2.49509253	0.00182815	0.01459531	0.00876805	2846	tags=63%, list=8%, signal=58%
REACTOME_(	REACTOME_(	38	0.75977284	2.41976488	0.00182815	0.01459531	0.00876805	4730	tags=71%, list=13%, signal=62%
REACTOME_S	REACTOME_S	38	0.77108785	2.45580152	0.00182815	0.01459531	0.00876805	3819	tags=50%, list=10%, signal=45%
WP_HEDGEH	WP_HEDGEH	43	0.68613053	2.21341731	0.00182815	0.01459531	0.00876805	3823	tags=35%, list=10%, signal=31%
WP_NEURAL_	WP_NEURAL_	43	0.61576206	1.98641271	0.00182815	0.01459531	0.00876805	3297	tags=30%, list=9%, signal=28%
WP_STRIATE	WP_STRIATE	38	0.66430201	2.11570431	0.00182815	0.01459531	0.00876805	2292	tags=34%, list=6%, signal=32%
BIOCARTA_IN	BIOCARTA_IN	34	0.63270216	1.98379106	0.00183486	0.01459531	0.00876805	4976	tags=35%, list=13%, signal=31%
PID_IL8_CXCI	PID_IL8_CXCI	34	0.62801151	1.96908389	0.00183486	0.01459531	0.00876805	4529	tags=35%, list=12%, signal=31%
REACTOME_I	REACTOME_I	45	0.78481179	2.54684321	0.00183486	0.01459531	0.00876805	3422	tags=67%, list=9%, signal=61%
REACTOME_I	REACTOME_I	45	0.60857596	1.97492897	0.00183486	0.01459531	0.00876805	6575	tags=60%, list=18%, signal=49%
REACTOME_I	REACTOME_I	34	0.64207801	2.01318837	0.00183486	0.01459531	0.00876805	4750	tags=35%, list=13%, signal=31%
WP_HEART_I	WP_HEART_I	45	0.59620573	1.93478555	0.00183486	0.01459531	0.00876805	6489	tags=47%, list=18%, signal=38%
KEGG_GRAFT	KEGG_GRAFT	37	0.71373703	2.25333739	0.00183824	0.01459531	0.00876805	7324	tags=70%, list=20%, signal=56%
NABA_BASEN	NABA_BASEN	40	0.82443663	2.63978465	0.00183824	0.01459531	0.00876805	4501	tags=72%, list=12%, signal=64%
PID_ER_NON	PID_ER_NON	40	0.65694118	2.10347672	0.00183824	0.01459531	0.00876805	5571	tags=38%, list=15%, signal=32%
REACTOME_(	REACTOME_(	37	0.64265669	2.02892985	0.00183824	0.01459531	0.00876805	4110	tags=35%, list=11%, signal=31%
REACTOME_(	REACTOME_(	37	0.77917939	2.45994531	0.00183824	0.01459531	0.00876805	7059	tags=84%, list=19%, signal=68%
WP_MICROG	WP_MICROG	40	0.66332359	2.1239127	0.00183824	0.01459531	0.00876805	4564	tags=52%, list=12%, signal=46%
WP_MIRNA_	WP_MIRNA_	40	0.68361921	2.18889775	0.00183824	0.01459531	0.00876805	3891	tags=52%, list=11%, signal=47%
WP_NEOVAS	WP_NEOVAS	37	0.74537551	2.35322316	0.00183824	0.01459531	0.00876805	5430	tags=49%, list=15%, signal=42%
WP_NEURAL_	WP_NEURAL_	40	0.63432724	2.03106855	0.00183824	0.01459531	0.00876805	3297	tags=32%, list=9%, signal=30%
BIOCARTA_T(	BIOCARTA_T(	44	0.62183482	2.01233576	0.00184162	0.01459531	0.00876805	9009	tags=55%, list=24%, signal=41%
NABA_COLLA	NABA_COLLA	44	0.7650708	2.47586541	0.00184162	0.01459531	0.00876805	2788	tags=68%, list=8%, signal=63%

REACTOME_(	REACTOME_(	44	0.7650708	2.47586541	0.00184162	0.01459531	0.00876805	2788	tags=68%, list=8%, signal=63%
REACTOME_I	REACTOME_I	44	0.62538073	2.02381077	0.00184162	0.01459531	0.00876805	9009	tags=66%, list=24%, signal=50%
REACTOME_(	REACTOME_(	33	0.68803875	2.14624568	0.00184502	0.01459531	0.00876805	4110	tags=39%, list=11%, signal=35%
REACTOME_(	REACTOME_(	33	0.72742657	2.26911078	0.00184502	0.01459531	0.00876805	3546	tags=45%, list=10%, signal=41%
REACTOME_I	REACTOME_I	31	0.68496044	2.10716295	0.00184502	0.01459531	0.00876805	3872	tags=39%, list=11%, signal=35%
WP_ENDOTH	WP_ENDOTH	33	0.7020329	2.18989858	0.00184502	0.01459531	0.00876805	6098	tags=45%, list=17%, signal=38%
PID_IL12_ST/	PID_IL12_ST/	32	0.70286914	2.17083466	0.00184843	0.01459531	0.00876805	7401	tags=62%, list=20%, signal=50%
PID_SYNDEC/	PID_SYNDEC/	32	0.6972987	2.15363016	0.00184843	0.01459531	0.00876805	2892	tags=44%, list=8%, signal=40%
REACTOME_(	REACTOME_(	32	0.68290734	2.10918196	0.00184843	0.01459531	0.00876805	3592	tags=41%, list=10%, signal=37%
WP_PURINEF	WP_PURINEF	32	0.68272082	2.1086059	0.00184843	0.01459531	0.00876805	5236	tags=56%, list=14%, signal=48%
REACTOME_(	REACTOME_(	42	0.62862612	2.00984776	0.00185874	0.01459531	0.00876805	3546	tags=31%, list=10%, signal=28%
REACTOME_I	REACTOME_I	42	0.74229137	2.37325911	0.00185874	0.01459531	0.00876805	3409	tags=57%, list=9%, signal=52%
REACTOME_I	REACTOME_I	42	0.67755264	2.16627597	0.00185874	0.01459531	0.00876805	5534	tags=43%, list=15%, signal=36%
REACTOME_\	REACTOME_\	42	0.66215464	2.11704537	0.00185874	0.01459531	0.00876805	6553	tags=62%, list=18%, signal=51%
REACTOME_/	REACTOME_/	30	0.6746881	2.05678548	0.0018622	0.01459531	0.00876805	3611	tags=40%, list=10%, signal=36%
REACTOME_I	REACTOME_I	30	0.79873208	2.43493332	0.0018622	0.01459531	0.00876805	4976	tags=70%, list=13%, signal=61%
REACTOME_I	REACTOME_I	30	0.72756124	2.21796916	0.0018622	0.01459531	0.00876805	4976	tags=57%, list=13%, signal=49%
WP_INFLAMI	WP_INFLAMI	30	0.69665274	2.12374465	0.0018622	0.01459531	0.00876805	6996	tags=83%, list=19%, signal=68%
WP_MATRIX_	WP_MATRIX_	30	0.74654228	2.27583282	0.0018622	0.01459531	0.00876805	4035	tags=60%, list=11%, signal=53%
PID_S1P_S1P	PID_S1P_S1P	29	0.74721824	2.26045133	0.00186916	0.01459531	0.00876805	5952	tags=55%, list=16%, signal=46%
WP_SELECTI\	WP_SELECTI\	29	0.77556555	2.34620634	0.00186916	0.01459531	0.00876805	6517	tags=76%, list=18%, signal=62%
BIOCARTA_N	BIOCARTA_N	26	0.77648689	2.29956329	0.00187266	0.01459531	0.00876805	6517	tags=77%, list=18%, signal=63%
PID_INTEGRI	PID_INTEGRI	26	0.79010995	2.33990794	0.00187266	0.01459531	0.00876805	5403	tags=69%, list=15%, signal=59%
REACTOME_I	REACTOME_I	26	0.79409984	2.35172398	0.00187266	0.01459531	0.00876805	6906	tags=81%, list=19%, signal=66%
KEGG_ASTHN	KEGG_ASTHN	28	0.72721637	2.18141421	0.00188324	0.01459531	0.00876805	6958	tags=75%, list=19%, signal=61%
PID_IL8_CXCI	PID_IL8_CXCI	28	0.694562	2.08346165	0.00188324	0.01459531	0.00876805	4529	tags=36%, list=12%, signal=31%
REACTOME_I	REACTOME_I	28	0.67293618	2.01859115	0.00188324	0.01459531	0.00876805	4750	tags=43%, list=13%, signal=37%
REACTOME_I	REACTOME_I	28	0.69667414	2.08979739	0.00188324	0.01459531	0.00876805	4828	tags=50%, list=13%, signal=43%
REACTOME_S	REACTOME_S	27	0.71518189	2.12676997	0.00190476	0.01459531	0.00876805	2781	tags=56%, list=8%, signal=51%
WP_CANCER_	WP_CANCER_	23	0.72992456	2.09826307	0.00190476	0.01459531	0.00876805	7363	tags=61%, list=20%, signal=49%
WP_GDNFRE	WP_GDNFRE	23	0.74628535	2.1452943	0.00190476	0.01459531	0.00876805	4986	tags=57%, list=14%, signal=49%
PID_LYMPH_	PID_LYMPH_	25	0.73606943	2.15039512	0.00191939	0.01459531	0.00876805	5571	tags=48%, list=15%, signal=41%
WP_HYPOTH	WP_HYPOTH	25	0.77551398	2.2656307	0.00191939	0.01459531	0.00876805	4850	tags=48%, list=13%, signal=42%
BIOCARTA_EI	BIOCARTA_EI	22	0.72154175	2.05460764	0.00192678	0.01459531	0.00876805	4981	tags=55%, list=14%, signal=47%
KEGG_GLYCC	KEGG_GLYCC	22	0.69890601	1.99015182	0.00192678	0.01459531	0.00876805	8014	tags=64%, list=22%, signal=50%
PID_HEDGEH	PID_HEDGEH	22	0.71931411	2.04826438	0.00192678	0.01459531	0.00876805	3823	tags=50%, list=10%, signal=45%
PID_S1P_ME	PID_S1P_ME	21	0.75424597	2.12089474	0.00192678	0.01459531	0.00876805	5964	tags=57%, list=16%, signal=48%
PID_S1P_S1P	PID_S1P_S1P	21	0.72369057	2.03497476	0.00192678	0.01459531	0.00876805	5236	tags=57%, list=14%, signal=49%
REACTOME_I	REACTOME_I	21	0.77015596	2.16563266	0.00192678	0.01459531	0.00876805	4235	tags=71%, list=11%, signal=63%
REACTOME_I	REACTOME_I	21	0.79326984	2.23062753	0.00192678	0.01459531	0.00876805	2226	tags=33%, list=6%, signal=31%
WP_COMPLE	WP_COMPLE	22	0.71099755	2.02458277	0.00192678	0.01459531	0.00876805	2980	tags=55%, list=8%, signal=50%
BIOCARTA_C	BIOCARTA_C	20	0.76417105	2.11961325	0.00195313	0.01459531	0.00876805	7348	tags=75%, list=20%, signal=60%
REACTOME_(	REACTOME_(	16	0.76239258	1.9990614	0.00195313	0.01459531	0.00876805	4198	tags=56%, list=11%, signal=50%
REACTOME_I	REACTOME_I	20	0.75115991	2.08352371	0.00195313	0.01459531	0.00876805	4101	tags=45%, list=11%, signal=40%
WP_HEDGEH	WP_HEDGEH	16	0.74908134	1.96415815	0.00195313	0.01459531	0.00876805	5852	tags=56%, list=16%, signal=47%
REACTOME_T	REACTOME_T	11	-0.7801641	-1.8646005	0.00195695	0.01459531	0.00876805	15	tags=27%, list=0%, signal=27%
REACTOME_(	REACTOME_(	10	-0.8170218	-1.8813789	0.00196078	0.01459531	0.00876805	2088	tags=60%, list=6%, signal=57%
BIOCARTA_B	BIOCARTA_B	14	0.74855018	1.92848175	0.00197628	0.01459531	0.00876805	2362	tags=21%, list=6%, signal=20%
BIOCARTA_B	BIOCARTA_B	17	0.74588741	1.97934752	0.00197628	0.01459531	0.00876805	2866	tags=29%, list=8%, signal=27%
BIOCARTA_CI	BIOCARTA_CI	14	0.78441761	2.02088664	0.00197628	0.01459531	0.00876805	2980	tags=64%, list=8%, signal=59%
BIOCARTA_C(	BIOCARTA_C(	19	0.74230025	2.03360879	0.00197628	0.01459531	0.00876805	2980	tags=58%, list=8%, signal=53%
BIOCARTA_D	BIOCARTA_D	17	0.74167228	1.9681619	0.00197628	0.01459531	0.00876805	6609	tags=76%, list=18%, signal=63%
BIOCARTA_L/	BIOCARTA_L/	17	0.75586393	2.00582203	0.00197628	0.01459531	0.00876805	4976	tags=65%, list=13%, signal=56%
BIOCARTA_LY	BIOCARTA_LY	14	0.81540385	2.10071615	0.00197628	0.01459531	0.00876805	4976	tags=79%, list=13%, signal=68%
BIOCARTA_T(	BIOCARTA_T(	19	0.80004686	2.19181162	0.00197628	0.01459531	0.00876805	6015	tags=79%, list=16%, signal=66%
REACTOME_/	REACTOME_/	17	0.75315732	1.99863953	0.00197628	0.01459531	0.00876805	2157	tags=35%, list=6%, signal=33%
REACTOME_I	REACTOME_I	14	0.73169319	1.88505328	0.00197628	0.01459531	0.00876805	9480	tags=79%, list=26%, signal=58%
REACTOME_I	REACTOME_I	14	0.75706253	1.95041204	0.00197628	0.01459531	0.00876805	4330	tags=64%, list=12%, signal=57%
REACTOME_(	REACTOME_(	19	0.7610027	2.08484609	0.00197628	0.01459531	0.00876805	5294	tags=63%, list=14%, signal=54%
REACTOME_I	REACTOME_I	14	0.80363647	2.07039998	0.00197628	0.01459531	0.00876805	4976	tags=71%, list=13%, signal=62%
REACTOME_I	REACTOME_I	19	0.74866029	2.05103278	0.00197628	0.01459531	0.00876805	2191	tags=26%, list=6%, signal=25%
SA_TRKA_RE(	SA_TRKA_RE(	17	0.78428028	2.08122995	0.00197628	0.01459531	0.00876805	2179	tags=29%, list=6%, signal=28%
WP_AMPLIFI(	WP_AMPLIFI(	17	0.77393958	2.05378905	0.00197628	0.01459531	0.00876805	4952	tags=59%, list=13%, signal=51%
WP_CANONI(	WP_CANONI(	17	0.77425342	2.05462187	0.00197628	0.01459531	0.00876805	6113	tags=65%, list=17%, signal=54%
WP_CELLS_A	WP_CELLS_A	17	0.75586393	2.00582203	0.00197628	0.01459531	0.00876805	4976	tags=65%, list=13%, signal=56%
WP_PLATELE	WP_PLATELE	17	0.75769676	2.01068576	0.00197628	0.01459531	0.00876805	5992	tags=88%, list=16%, signal=74%
WP_REGULA	WP_REGULA	17	0.77882325	2.06674875	0.00197628	0.01459531	0.00876805	3851	tags=47%, list=10%, signal=42%
WP_SMALL_I	WP_SMALL_I	19	0.77025235	2.11018647	0.00197628	0.01459531	0.00876805	3759	tags=74%, list=10%, signal=66%
REACTOME_I	REACTOME_I	12	-0.7207296	-1.7859863	0.0019802	0.01459531	0.00876805	6968	tags=83%, list=19%, signal=68%
BIOCARTA_A(	BIOCARTA_A(	13	0.78830392	1.99026535	0.00198413	0.01459531	0.00876805	3409	tags=77%, list=9%, signal=70%
BIOCARTA_A:	BIOCARTA_A:	13	0.78308442	1.97708746	0.00198413	0.01459531	0.00876805	6906	tags=85%, list=19%, signal=69%
BIOCARTA_C	BIOCARTA_C	13	0.77375394	1.95353039	0.00198413	0.01459531	0.00876805	5772	tags=69%, list=16%, signal=58%
REACTOME_I	REACTOME_I	13	0.84377803	2.13032327	0.00198413	0.01459531	0.00876805	558	tags=38%, list=2%, signal=38%
WP_BMP2W	WP_BMP2W	13	0.75395364	1.90353972	0.00198413	0.01459531	0.00876805	2156	tags=31%, list=6%, signal=29%

WP_CONTRC	WP_CONTRC	13	0.76048977	1.92004175	0.00198413	0.01459531	0.00876805	6015	tags=69%, list=16%, signal=58%
WP_DEVELOI	WP_DEVELOI	13	0.74929711	1.89178317	0.00198413	0.01459531	0.00876805	8495	tags=92%, list=23%, signal=71%
REACTOME_/	REACTOME_/	18	0.79791768	2.15135806	0.00199203	0.01459531	0.00876805	3823	tags=44%, list=10%, signal=40%
REACTOME_(	REACTOME_(	18	0.82030077	2.21170769	0.00199203	0.01459531	0.00876805	2218	tags=44%, list=6%, signal=42%
REACTOME_(	REACTOME_(	18	0.87297196	2.35372059	0.00199203	0.01459531	0.00876805	4272	tags=94%, list=12%, signal=84%
WP_MFAP5_	WP_MFAP5_	18	0.75177578	2.02694956	0.00199203	0.01459531	0.00876805	4561	tags=44%, list=12%, signal=39%
WP_MIR5093	WP_MIR5093	18	0.87111219	2.34870623	0.00199203	0.01459531	0.00876805	1681	tags=61%, list=5%, signal=58%
BIOCARTA_R.	BIOCARTA_R.	15	0.773902	2.00182233	0.002	0.01459531	0.00876805	4692	tags=67%, list=13%, signal=58%
PID_LPA4_PA	PID_LPA4_PA	15	0.76823389	1.98716086	0.002	0.01459531	0.00876805	4110	tags=47%, list=11%, signal=41%
REACTOME_/	REACTOME_/	15	0.84467035	2.1848761	0.002	0.01459531	0.00876805	4272	tags=73%, list=12%, signal=65%
REACTOME_(	REACTOME_(	15	0.78149678	2.02146745	0.002	0.01459531	0.00876805	3992	tags=67%, list=11%, signal=59%
REACTOME_I	REACTOME_I	15	0.77796973	2.01234418	0.002	0.01459531	0.00876805	3035	tags=60%, list=8%, signal=55%
BIOCARTA_E.	BIOCARTA_E.	13	-0.7687522	-1.9459964	0.00200803	0.01459531	0.00876805	2882	tags=54%, list=8%, signal=50%
BIOCARTA_B.	BIOCARTA_B.	12	0.76149987	1.86660909	0.00201207	0.01459531	0.00876805	2362	tags=25%, list=6%, signal=23%
BIOCARTA_B	BIOCARTA_B	12	0.80783627	1.98019012	0.00201207	0.01459531	0.00876805	6906	tags=92%, list=19%, signal=75%
BIOCARTA_C	BIOCARTA_C	12	0.78836823	1.93246954	0.00201207	0.01459531	0.00876805	5874	tags=58%, list=16%, signal=49%
BIOCARTA_T(	BIOCARTA_T(	12	0.80724168	1.97873264	0.00201207	0.01459531	0.00876805	6906	tags=92%, list=19%, signal=75%
BIOCARTA_T(	BIOCARTA_T(	12	0.86963719	2.13167821	0.00201207	0.01459531	0.00876805	4816	tags=100%, list=13%, signal=87%
BIOCARTA_T	BIOCARTA_T	12	0.86963719	2.13167821	0.00201207	0.01459531	0.00876805	4816	tags=100%, list=13%, signal=87%
REACTOME_I	REACTOME_I	12	0.86109872	2.11074847	0.00201207	0.01459531	0.00876805	3163	tags=58%, list=9%, signal=53%
BIOCARTA_N	BIOCARTA_N	10	0.81253916	1.90305595	0.00203252	0.01459531	0.00876805	3409	tags=70%, list=9%, signal=64%
PID_VEGF_VI	PID_VEGF_VI	10	0.82390488	1.9296757	0.00203252	0.01459531	0.00876805	4984	tags=90%, list=14%, signal=78%
REACTOME_/	REACTOME_/	10	0.80071703	1.87536721	0.00203252	0.01459531	0.00876805	4110	tags=60%, list=11%, signal=53%
BIOCARTA_M	BIOCARTA_M	11	0.84738956	2.03270038	0.00203666	0.01459531	0.00876805	5635	tags=100%, list=15%, signal=85%
REACTOME_I	REACTOME_I	11	0.79339825	1.90318717	0.00203666	0.01459531	0.00876805	2386	tags=55%, list=6%, signal=51%
REACTOME_I	REACTOME_I	11	0.81282558	1.94978903	0.00203666	0.01459531	0.00876805	3600	tags=64%, list=10%, signal=57%
REACTOME_S	REACTOME_S	11	0.86637543	2.0782433	0.00203666	0.01459531	0.00876805	3057	tags=64%, list=8%, signal=58%
KEGG_NITRO	KEGG_NITRO	23	-0.6471194	-1.9422264	0.00209644	0.01459531	0.00876805	6968	tags=65%, list=19%, signal=53%
REACTOME_I	REACTOME_I	23	-0.7295704	-2.1896899	0.00209644	0.01459531	0.00876805	2244	tags=52%, list=6%, signal=49%
REACTOME_I	REACTOME_I	27	-0.6949343	-2.1602975	0.00209644	0.01459531	0.00876805	2244	tags=48%, list=6%, signal=45%
WP_DNA_RE	WP_DNA_RE	42	-0.5715485	-1.9553491	0.00215517	0.01459531	0.00876805	13242	tags=79%, list=36%, signal=50%
REACTOME_/	REACTOME_/	33	-0.5940945	-1.9215418	0.00217391	0.01459531	0.00876805	13242	tags=88%, list=36%, signal=56%
REACTOME_\	REACTOME_\	44	-0.4833293	-1.6665033	0.00217865	0.01459531	0.00876805	17327	tags=82%, list=47%, signal=43%
REACTOME_/	REACTOME_/	37	-0.5336438	-1.7731375	0.00218341	0.01459531	0.00876805	12410	tags=70%, list=34%, signal=47%
WP_ZINC_HC	WP_ZINC_HC	37	-0.5605357	-1.8624912	0.00218341	0.01459531	0.00876805	7939	tags=41%, list=22%, signal=32%
PID_HNF3B_I	PID_HNF3B_I	45	-0.5599387	-1.9434184	0.00218818	0.01459531	0.00876805	6628	tags=51%, list=18%, signal=42%
PID_PLK1_PA	PID_PLK1_PA	45	-0.51784	-1.7973033	0.00218818	0.01459531	0.00876805	10085	tags=53%, list=27%, signal=39%
REACTOME_S	REACTOME_S	46	-0.4922108	-1.7158085	0.00220751	0.01459531	0.00876805	16669	tags=80%, list=45%, signal=44%
KEGG_DNA_I	KEGG_DNA_I	36	-0.5367631	-1.7727883	0.00221729	0.01459531	0.00876805	13973	tags=75%, list=38%, signal=47%
REACTOME_I	REACTOME_I	52	-0.4867214	-1.7391299	0.00226244	0.01459531	0.00876805	17646	tags=79%, list=48%, signal=41%
WP_OXIDATI	WP_OXIDATI	52	-0.5065588	-1.8100117	0.00226244	0.01459531	0.00876805	11809	tags=71%, list=32%, signal=48%
REACTOME_T	REACTOME_T	58	-0.4926674	-1.7874574	0.0022779	0.01459531	0.00876805	16600	tags=88%, list=45%, signal=48%
REACTOME_I	REACTOME_I	63	-0.4930836	-1.8268621	0.00228311	0.01459531	0.00876805	12804	tags=68%, list=35%, signal=45%
REACTOME_I	REACTOME_I	64	-0.4667252	-1.7365611	0.00228311	0.01459531	0.00876805	16991	tags=89%, list=46%, signal=48%
REACTOME_S	REACTOME_S	54	-0.504707	-1.813848	0.00229358	0.01459531	0.00876805	16600	tags=85%, list=45%, signal=47%
REACTOME_/	REACTOME_/	60	-0.5113048	-1.866028	0.00229885	0.01459531	0.00876805	15087	tags=77%, list=41%, signal=45%
REACTOME_(	REACTOME_(	49	-0.4893969	-1.7167889	0.00229885	0.01459531	0.00876805	13777	tags=69%, list=37%, signal=44%
REACTOME_I	REACTOME_I	60	-0.4922729	-1.7965704	0.00229885	0.01459531	0.00876805	16659	tags=83%, list=45%, signal=46%
REACTOME_/	REACTOME_/	94	-0.5740071	-2.2893776	0.00230415	0.01459531	0.00876805	1859	tags=29%, list=5%, signal=27%
REACTOME_I	REACTOME_I	94	-0.5251574	-2.0945448	0.00230415	0.01459531	0.00876805	15230	tags=84%, list=41%, signal=49%
REACTOME_I	REACTOME_I	91	-0.4402177	-1.7476307	0.00230415	0.01459531	0.00876805	14410	tags=60%, list=39%, signal=37%
REACTOME_I	REACTOME_I	94	-0.5183253	-2.0672956	0.00230415	0.01459531	0.00876805	14496	tags=80%, list=39%, signal=49%
REACTOME_S	REACTOME_S	91	-0.4354396	-1.7286622	0.00230415	0.01459531	0.00876805	17260	tags=80%, list=47%, signal=43%
WP_PROXIM	WP_PROXIM	57	-0.4899813	-1.7676108	0.00230415	0.01459531	0.00876805	10315	tags=53%, list=28%, signal=38%
KEGG_RIBOS	KEGG_RIBOS	88	-0.5186192	-2.0427408	0.00230947	0.01459531	0.00876805	15230	tags=85%, list=41%, signal=50%
REACTOME_/	REACTOME_/	88	-0.4355228	-1.7154401	0.00230947	0.01459531	0.00876805	17260	tags=78%, list=47%, signal=42%
REACTOME_I	REACTOME_I	56	-0.4525127	-1.6347203	0.00230947	0.01459531	0.00876805	14195	tags=68%, list=39%, signal=42%
WP_CYTOPL/	WP_CYTOPL/	88	-0.4993767	-1.9669484	0.00230947	0.01459531	0.00876805	15648	tags=84%, list=42%, signal=49%
REACTOME_I	REACTOME_I	89	-0.4470579	-1.7603266	0.00232019	0.01459531	0.00876805	13114	tags=61%, list=36%, signal=39%
REACTOME_I	REACTOME_I	90	-0.4330224	-1.7116603	0.00232019	0.01459531	0.00876805	12804	tags=59%, list=35%, signal=39%
REACTOME_I	REACTOME_I	89	-0.5275075	-2.0771036	0.00232019	0.01459531	0.00876805	13777	tags=75%, list=37%, signal=47%
REACTOME_I	REACTOME_I	89	-0.4633513	-1.8244834	0.00232019	0.01459531	0.00876805	15423	tags=72%, list=42%, signal=42%
WP_ELECTRC	WP_ELECTRC	90	-0.5137106	-2.0306063	0.00232019	0.01459531	0.00876805	13209	tags=77%, list=36%, signal=49%
WP_MITOCH	WP_MITOCH	50	-0.4938961	-1.7405117	0.00232019	0.01459531	0.00876805	13777	tags=70%, list=37%, signal=44%
KEGG_PERO)	KEGG_PERO)	78	-0.4376826	-1.6911542	0.00232558	0.01459531	0.00876805	12463	tags=53%, list=34%, signal=35%
REACTOME_I	REACTOME_I	66	-0.4480497	-1.6761523	0.00232558	0.01459531	0.00876805	14967	tags=65%, list=41%, signal=39%
REACTOME_S	REACTOME_S	66	-0.4858575	-1.8175912	0.00232558	0.01459531	0.00876805	14195	tags=71%, list=39%, signal=44%
REACTOME_I	REACTOME_I	98	-0.4138159	-1.6662914	0.002331	0.01459531	0.00876805	12340	tags=52%, list=33%, signal=35%
WP_RETINOE	WP_RETINOE	87	-0.4890267	-1.917762	0.00234192	0.01459531	0.00876805	13486	tags=66%, list=37%, signal=42%
REACTOME_I	REACTOME_I	85	-0.4948876	-1.9343626	0.00235294	0.01459531	0.00876805	15617	tags=81%, list=42%, signal=47%
REACTOME_(	REACTOME_(	95	-0.4579673	-1.8208076	0.00235294	0.01459531	0.00876805	12340	tags=55%, list=33%, signal=37%
REACTOME_I	REACTOME_I	85	-0.4686472	-1.8317971	0.00235294	0.01459531	0.00876805	12804	tags=65%, list=35%, signal=42%
REACTOME_I	REACTOME_I	73	-0.5359298	-2.0319577	0.00236407	0.01459531	0.00876805	12804	tags=73%, list=35%, signal=47%
REACTOME_I	REACTOME_I	67	-0.4545911	-1.69638	0.00236407	0.01459531	0.00876805	12507	tags=75%, list=34%, signal=49%

REACTOME_I	REACTOME_I	102	-0.5010767	-2.0242331	0.00236407	0.01459531	0.00876805	15230	tags=81%, list=41%, signal=48%
REACTOME_/	REACTOME_/	74	-0.4507973	-1.7137939	0.00236967	0.01459531	0.00876805	16950	tags=78%, list=46%, signal=42%
REACTOME_/	REACTOME_/	68	-0.4839404	-1.812702	0.00236967	0.01459531	0.00876805	14937	tags=79%, list=41%, signal=47%
REACTOME_I	REACTOME_I	74	-0.440726	-1.675506	0.00236967	0.01459531	0.00876805	15394	tags=72%, list=42%, signal=42%
REACTOME_(	REACTOME_(	71	-0.4431795	-1.6625409	0.0023753	0.01459531	0.00876805	15617	tags=76%, list=42%, signal=44%
REACTOME_I	REACTOME_I	71	-0.4655159	-1.7463337	0.0023753	0.01459531	0.00876805	13059	tags=65%, list=35%, signal=42%
REACTOME_¯	REACTOME_¯	84	-0.4422851	-1.7189367	0.0023753	0.01459531	0.00876805	16600	tags=73%, list=45%, signal=40%
KEGG_METAI	KEGG_METAI	69	-0.4588126	-1.7202978	0.00238095	0.01459531	0.00876805	5905	tags=42%, list=16%, signal=35%
REACTOME_(	REACTOME_(	72	-0.4854788	-1.8307516	0.00239808	0.01459531	0.00876805	12804	tags=68%, list=35%, signal=45%
REACTOME_I	REACTOME_I	107	-0.4332368	-1.762913	0.00239808	0.01459531	0.00876805	15423	tags=65%, list=42%, signal=38%
REACTOME_I	REACTOME_I	146	-0.3742977	-1.6058782	0.00240385	0.01459531	0.00876805	15423	tags=59%, list=42%, signal=34%
REACTOME_I	REACTOME_I	113	-0.4597186	-1.8842851	0.00240385	0.01459531	0.00876805	16600	tags=73%, list=45%, signal=40%
REACTOME_I	REACTOME_I	149	-0.4267327	-1.8369503	0.00240385	0.01459531	0.00876805	13263	tags=60%, list=36%, signal=38%
REACTOME_I	REACTOME_I	104	-0.4146645	-1.6754286	0.00240385	0.01459531	0.00876805	15394	tags=65%, list=42%, signal=38%
REACTOME_!	REACTOME_!	113	-0.5073168	-2.0793793	0.00240385	0.01459531	0.00876805	15700	tags=84%, list=43%, signal=48%
REACTOME_¯	REACTOME_¯	105	-0.4533571	-1.8365801	0.00240385	0.01459531	0.00876805	17395	tags=82%, list=47%, signal=43%
REACTOME_I	REACTOME_I	111	-0.5128329	-2.1017636	0.00241546	0.01459531	0.00876805	13777	tags=74%, list=37%, signal=46%
REACTOME_(	REACTOME_(	168	-0.463155	-2.037609	0.00242718	0.01459531	0.00876805	14195	tags=65%, list=39%, signal=40%
REACTOME_I	REACTOME_I	116	-0.4848313	-1.988595	0.00242718	0.01459531	0.00876805	15230	tags=78%, list=41%, signal=46%
REACTOME_¯	REACTOME_¯	108	-0.4554558	-1.8564377	0.00242718	0.01459531	0.00876805	18008	tags=85%, list=49%, signal=44%
KEGG_OXIDA	KEGG_OXIDA	118	-0.4890305	-2.0199467	0.00243309	0.01459531	0.00876805	11534	tags=62%, list=31%, signal=43%
REACTOME_I	REACTOME_I	106	-0.4344442	-1.7598095	0.00243309	0.01459531	0.00876805	10009	tags=53%, list=27%, signal=39%
REACTOME_!	REACTOME_!	118	-0.4276825	-1.7665482	0.00243309	0.01459531	0.00876805	15269	tags=81%, list=41%, signal=48%
REACTOME_I	REACTOME_I	120	-0.5070744	-2.093657	0.00243902	0.01459531	0.00876805	15230	tags=80%, list=41%, signal=47%
REACTOME_I	REACTOME_I	163	-0.3963969	-1.7292109	0.00245098	0.01459531	0.00876805	17173	tags=74%, list=47%, signal=40%
REACTOME_I	REACTOME_I	159	-0.3421702	-1.4838494	0.002457	0.01459531	0.00876805	16600	tags=64%, list=45%, signal=35%
KEGG_HUNTI	KEGG_HUNTI	174	-0.3895199	-1.7235006	0.00246305	0.01459531	0.00876805	13831	tags=60%, list=38%, signal=38%
REACTOME_I	REACTOME_I	141	-0.3965138	-1.6815386	0.00246305	0.01459531	0.00876805	17395	tags=73%, list=47%, signal=39%
REACTOME_(	REACTOME_(	136	-0.4078488	-1.7231502	0.00246914	0.01459531	0.00876805	15721	tags=74%, list=43%, signal=42%
REACTOME_I	REACTOME_I	138	-0.4153195	-1.7572564	0.00247525	0.01459531	0.00876805	12507	tags=51%, list=34%, signal=34%
REACTOME_!	REACTOME_!	162	-0.4203454	-1.8318576	0.00247525	0.01459531	0.00876805	17277	tags=78%, list=47%, signal=42%
REACTOME_¯	REACTOME_¯	162	-0.4665582	-2.0332523	0.00247525	0.01459531	0.00876805	14306	tags=71%, list=39%, signal=44%
REACTOME_I	REACTOME_I	157	-0.4722288	-2.0405569	0.00248756	0.01459531	0.00876805	17327	tags=85%, list=47%, signal=45%
REACTOME_I	REACTOME_I	143	-0.3840187	-1.6257752	0.00248756	0.01459531	0.00876805	13816	tags=61%, list=37%, signal=38%
REACTOME_I	REACTOME_I	126	-0.3948497	-1.6488058	0.00249377	0.01459531	0.00876805	10153	tags=44%, list=28%, signal=32%
REACTOME_I	REACTOME_I	166	-0.3625699	-1.5845854	0.0025	0.01459531	0.00876805	17277	tags=68%, list=47%, signal=36%
KEGG_SPLICE	KEGG_SPLICE	127	-0.4200327	-1.7545832	0.00250627	0.01459531	0.00876805	17864	tags=76%, list=48%, signal=40%
REACTOME_I	REACTOME_I	128	-0.4632355	-1.9397897	0.00250627	0.01459531	0.00876805	15641	tags=84%, list=42%, signal=48%
REACTOME_I	REACTOME_I	128	-0.7740927	-3.2414985	0.00250627	0.01459531	0.00876805	2251	tags=46%, list=6%, signal=43%
REACTOME_I	REACTOME_I	139	-0.3938679	-1.6642822	0.00251889	0.01463297	0.00879068	14195	tags=49%, list=39%, signal=30%
REACTOME_(	REACTOME_(	140	-0.441431	-1.8600731	0.00253807	0.01470843	0.00883601	13266	tags=60%, list=36%, signal=39%
REACTOME_I	REACTOME_I	188	-0.4110672	-1.8362264	0.00257732	0.01486338	0.00892909	17747	tags=74%, list=48%, signal=39%
REACTOME_!	REACTOME_!	191	-0.3877472	-1.7341179	0.00257732	0.01486338	0.00892909	17327	tags=74%, list=47%, signal=39%
REACTOME_I	REACTOME_I	203	-0.4890904	-2.1922665	0.00277778	0.01594791	0.00958062	17723	tags=88%, list=48%, signal=46%
REACTOME_I	REACTOME_I	236	-0.3927744	-1.7908478	0.00278552	0.01594791	0.00958062	16243	tags=66%, list=44%, signal=37%
REACTOME_I	REACTOME_I	242	-0.4176582	-1.9153859	0.00278552	0.01594791	0.00958062	17864	tags=75%, list=48%, signal=39%
REACTOME_I	REACTOME_I	202	-0.3591079	-1.6073673	0.0027933	0.01595402	0.00958429	14431	tags=53%, list=39%, signal=32%
REACTOME_I	REACTOME_I	216	-0.7289172	-3.2910973	0.00284091	0.0161713	0.00971481	4994	tags=50%, list=14%, signal=43%
REACTOME_I	REACTOME_I	475	0.31197185	1.43240841	0.00284495	0.0161713	0.00971481	6610	tags=27%, list=18%, signal=23%
REACTOME_(	REACTOME_(	292	-0.4351668	-2.0450256	0.00286533	0.01624826	0.00976105	12410	tags=67%, list=34%, signal=45%
REACTOME_¯	REACTOME_¯	293	-0.4869554	-2.2873424	0.00287356	0.01625616	0.00976579	17146	tags=85%, list=47%, signal=46%
REACTOME_I	REACTOME_I	330	-0.3544744	-1.6758158	0.00294118	0.01659913	0.00997183	17338	tags=64%, list=47%, signal=34%
REACTOME_I	REACTOME_I	304	0.33265417	1.4615806	0.0030349	0.0170875	0.01026522	5996	tags=20%, list=16%, signal=17%
KEGG_OLFAC	KEGG_OLFAC	377	-0.3256918	-1.5591852	0.0030581	0.01713692	0.01029491	7840	tags=26%, list=21%, signal=21%
REACTOME_I	REACTOME_I	374	-0.3565787	-1.7036484	0.0030581	0.01713692	0.01029491	13499	tags=64%, list=37%, signal=41%
REACTOME_I	REACTOME_I	414	-0.3771558	-1.8317692	0.00310559	0.01736208	0.01043017	17395	tags=69%, list=47%, signal=37%
WP_TGFBET/	WP_TGFBET/	132	0.41921748	1.67854923	0.00331126	0.01845573	0.01108717	7401	tags=27%, list=20%, signal=21%
REACTOME_¯	REACTOME_¯	127	0.41335764	1.64143467	0.00331675	0.01845573	0.01108717	8367	tags=29%, list=23%, signal=23%
WP_INSULIN_	WP_INSULIN_	159	0.39794646	1.62382748	0.00336134	0.01866017	0.01120999	7558	tags=28%, list=21%, signal=22%
WP_THERMC	WP_THERMC	108	0.4382644	1.69224845	0.00338983	0.01874054	0.01125827	5407	tags=19%, list=15%, signal=17%
REACTOME_I	REACTOME_I	110	0.44007159	1.70296372	0.00340136	0.01874054	0.01125827	6250	tags=34%, list=17%, signal=28%
REACTOME_(	REACTOME_(	110	0.43805109	1.6951449	0.00340136	0.01874054	0.01125827	4750	tags=33%, list=13%, signal=29%
NABA_ECM_/	NABA_ECM_/	169	0.39057998	1.59923172	0.00341297	0.01874054	0.01125827	5294	tags=40%, list=14%, signal=35%
KEGG_MELAI	KEGG_MELAI	71	0.51680954	1.84419419	0.00344234	0.01874054	0.01125827	4373	tags=28%, list=12%, signal=25%
REACTOME_I	REACTOME_I	67	0.50958245	1.79455952	0.00345423	0.01874054	0.01125827	4730	tags=46%, list=13%, signal=40%
REACTOME_I	REACTOME_I	102	0.44832922	1.70601457	0.00345423	0.01874054	0.01125827	6250	tags=31%, list=17%, signal=26%
KEGG_MELAI	KEGG_MELAI	101	0.46350733	1.76259405	0.00346021	0.01874054	0.01125827	5504	tags=34%, list=15%, signal=29%
WP_22Q112_	WP_22Q112_	95	0.46441351	1.75337048	0.0034662	0.01874054	0.01125827	1828	tags=15%, list=5%, signal=14%
KEGG_FC_GA	KEGG_FC_GA	96	0.47009569	1.77712881	0.00347826	0.01874054	0.01125827	7348	tags=38%, list=20%, signal=30%
PID_CD8_TCI	PID_CD8_TCI	65	0.51779747	1.81484213	0.00347826	0.01874054	0.01125827	7363	tags=40%, list=20%, signal=32%
WP_SMALL_(	WP_SMALL_(	96	0.45775826	1.73048895	0.00347826	0.01874054	0.01125827	4976	tags=25%, list=13%, signal=22%
REACTOME_(	REACTOME_(	80	0.50941789	1.85217175	0.0034904	0.01874054	0.01125827	7017	tags=41%, list=19%, signal=33%
REACTOME_!	REACTOME_!	79	0.47879479	1.73549497	0.0034965	0.01874054	0.01125827	6180	tags=27%, list=17%, signal=22%
PID_FAK_PA1	PID_FAK_PA1	59	0.49240516	1.694246	0.00350263	0.01874054	0.01125827	8400	tags=41%, list=23%, signal=31%

REACTOME_(	REACTOME_(	62	0.49072052	1.7053766	0.00350877	0.01874054	0.01125827	4787 tags=31%, list=13%, signal=27%
WNT_SIGNAI	WNT_SIGNAI	88	0.47102077	1.75078138	0.00351494	0.01874054	0.01125827	5683 tags=25%, list=15%, signal=21%
KEGG_ACUTE	KEGG_ACUTE	57	0.53204165	1.81920016	0.00352113	0.01874054	0.01125827	6167 tags=32%, list=17%, signal=26%
PID_IL2_1PA	PID_IL2_1PA	55	0.5302035	1.79410351	0.00353357	0.01874054	0.01125827	8596 tags=44%, list=23%, signal=34%
WP_NOCGM	WP_NOCGM	48	0.57279648	1.90265164	0.00353357	0.01874054	0.01125827	2777 tags=29%, list=8%, signal=27%
PID_PTP1B_F	PID_PTP1B_F	51	0.54266409	1.80891783	0.00355872	0.01883188	0.01131315	5782 tags=43%, list=16%, signal=36%
REACTOME_S	REACTOME_S	36	0.62072179	1.95750481	0.00362976	0.01905984	0.01145009	5333 tags=44%, list=14%, signal=38%
KEGG_TYPE_	KEGG_TYPE_	41	0.59883939	1.92451608	0.00365631	0.01905984	0.01145009	7324 tags=59%, list=20%, signal=47%
PID_CXCR3_F	PID_CXCR3_F	43	0.57399622	1.85167851	0.00365631	0.01905984	0.01145009	5571 tags=37%, list=15%, signal=32%
PID_THROME	PID_THROME	43	0.58695153	1.89347161	0.00365631	0.01905984	0.01145009	7505 tags=49%, list=20%, signal=39%
PID_EPHA_FV	PID_EPHA_FV	34	0.61656258	1.93318662	0.00366972	0.01905984	0.01145009	3350 tags=35%, list=9%, signal=32%
SIG_CHEMOT	SIG_CHEMOT	45	0.55388524	1.79744861	0.00366972	0.01905984	0.01145009	2651 tags=22%, list=7%, signal=21%
WP_PROSTA(	WP_PROSTA(	45	0.56379293	1.82960069	0.00366972	0.01905984	0.01145009	5783 tags=42%, list=16%, signal=36%
PID_INTEGRI	PID_INTEGRI	33	0.64432718	2.00989322	0.00369004	0.01905984	0.01145009	5185 tags=39%, list=14%, signal=34%
WP_DISRUPT	WP_DISRUPT	33	0.65194891	2.03366821	0.00369004	0.01905984	0.01145009	3862 tags=39%, list=10%, signal=35%
WP_PRION_D	WP_PRION_D	33	0.63984154	1.99590089	0.00369004	0.01905984	0.01145009	4307 tags=36%, list=12%, signal=32%
WP_RESISTIN	WP_RESISTIN	33	0.63226442	1.97226505	0.00369004	0.01905984	0.01145009	8170 tags=45%, list=22%, signal=35%
PID_IL27_PA	PID_IL27_PA	26	0.65174432	1.93013858	0.00374532	0.01926164	0.01157132	6575 tags=62%, list=18%, signal=51%
REACTOME_/	REACTOME_/	26	0.64567146	1.91215383	0.00374532	0.01926164	0.01157132	6521 tags=46%, list=18%, signal=38%
REACTOME_/	REACTOME_/	28	0.65114542	1.95322592	0.00376648	0.01928697	0.01158654	7017 tags=54%, list=19%, signal=43%
REACTOME_T	REACTOME_T	28	0.65525057	1.96554004	0.00376648	0.01928697	0.01158654	6239 tags=57%, list=17%, signal=48%
BIOCARTA_IN	BIOCARTA_IN	27	0.68307041	2.03127857	0.00380952	0.01932399	0.01160877	8305 tags=78%, list=23%, signal=60%
PID_WNT_SI(	PID_WNT_SI(	27	0.65498281	1.94775315	0.00380952	0.01932399	0.01160877	5683 tags=48%, list=15%, signal=41%
BIOCARTA_B/	BIOCARTA_B/	25	0.63701581	1.86101425	0.00383877	0.01932399	0.01160877	7558 tags=48%, list=21%, signal=38%
PID_INTEGRI	PID_INTEGRI	25	0.65292333	1.9074874	0.00383877	0.01932399	0.01160877	3415 tags=40%, list=9%, signal=36%
REACTOME_(	REACTOME_(	25	0.68443588	1.99954995	0.00383877	0.01932399	0.01160877	3546 tags=40%, list=10%, signal=36%
REACTOME_I	REACTOME_I	25	0.63607229	1.85825779	0.00383877	0.01932399	0.01160877	4294 tags=40%, list=12%, signal=35%
WP_DIFFERE	WP_DIFFERE	25	0.6644027	1.94102388	0.00383877	0.01932399	0.01160877	1639 tags=28%, list=4%, signal=27%
WP_RELATIO	WP_RELATIO	25	0.65534087	1.91455011	0.00383877	0.01932399	0.01160877	2285 tags=28%, list=6%, signal=26%
BIOCARTA_EI	BIOCARTA_EI	22	0.68533215	1.95149993	0.00385356	0.01935744	0.01162887	7348 tags=55%, list=20%, signal=44%
REACTOME_I	REACTOME_I	24	0.66364025	1.91244699	0.00389105	0.01942256	0.01166799	8255 tags=54%, list=22%, signal=42%
REACTOME_I	REACTOME_I	24	0.66683405	1.92165072	0.00389105	0.01942256	0.01166799	4103 tags=38%, list=11%, signal=33%
WP_ANGIOG	WP_ANGIOG	24	0.70515598	2.03208505	0.00389105	0.01942256	0.01166799	4984 tags=46%, list=14%, signal=40%
BIOCARTA_SI	BIOCARTA_SI	16	0.70726043	1.85449999	0.00390625	0.01945484	0.01168738	5852 tags=50%, list=16%, signal=42%
REACTOME_I	REACTOME_I	11	-0.7662059	-1.8312403	0.00391389	0.01945484	0.01168738	7939 tags=82%, list=22%, signal=64%
BIOCARTA_C(	BIOCARTA_C(	17	0.721208	1.91385623	0.00395257	0.0195154	0.01172377	4204 tags=35%, list=11%, signal=31%
PID_S1P_S1P	PID_S1P_S1P	14	0.72266069	1.86178295	0.00395257	0.0195154	0.01172377	5964 tags=50%, list=16%, signal=42%
WP_SIMPLIFI	WP_SIMPLIFI	18	0.70049491	1.88868528	0.00398406	0.0195154	0.01172377	6751 tags=56%, list=18%, signal=45%
BIOCARTA_EI	BIOCARTA_EI	15	0.71309527	1.84453592	0.004	0.0195154	0.01172377	3836 tags=53%, list=10%, signal=48%
BIOCARTA_G	BIOCARTA_G	15	0.75998992	1.9658365	0.004	0.0195154	0.01172377	8116 tags=87%, list=22%, signal=68%
BIOCARTA_N	BIOCARTA_N	15	0.73397134	1.89853526	0.004	0.0195154	0.01172377	5500 tags=73%, list=15%, signal=62%
REACTOME_I	REACTOME_I	15	0.73433757	1.89948256	0.004	0.0195154	0.01172377	5097 tags=67%, list=14%, signal=57%
REACTOME_Y	REACTOME_Y	15	0.74347287	1.92311248	0.004	0.0195154	0.01172377	7884 tags=60%, list=21%, signal=47%
SA_MMP_CY	SA_MMP_CY	15	0.76311703	1.97392528	0.004	0.0195154	0.01172377	6967 tags=80%, list=19%, signal=65%
REACTOME_I	REACTOME_I	12	0.75389994	1.84797994	0.00402414	0.01955234	0.01174595	5097 tags=67%, list=14%, signal=57%
KEGG_STERO	KEGG_STERO	17	-0.6898583	-1.9032928	0.00403226	0.01955234	0.01174595	8783 tags=76%, list=24%, signal=58%
REACTOME_I	REACTOME_I	17	-0.6614544	-1.8249276	0.00403226	0.01955234	0.01174595	7281 tags=71%, list=20%, signal=57%
REACTOME_I	REACTOME_I	16	-0.7034417	-1.8937943	0.00408163	0.01975144	0.01186557	4436 tags=62%, list=12%, signal=55%
WP_UREA_CY	WP_UREA_CY	21	-0.6381578	-1.8715935	0.00414079	0.01999697	0.01201307	11476 tags=81%, list=31%, signal=56%
REACTOME_(	REACTOME_(	25	-0.6177455	-1.9119378	0.004158	0.02003939	0.01203855	8708 tags=76%, list=24%, signal=58%
WP_BLOOD_	WP_BLOOD_	23	-0.6152641	-1.8466178	0.00419287	0.02016653	0.01211493	1433 tags=30%, list=4%, signal=29%
WP_PPAR_AI	WP_PPAR_AI	26	-0.6124238	-1.8862984	0.0042735	0.02051282	0.01232296	2333 tags=42%, list=6%, signal=40%
WP_PREGNA	WP_PREGNA	33	-0.5440676	-1.7597346	0.00434783	0.02082749	0.012512	5999 tags=39%, list=16%, signal=33%
REACTOME_T	REACTOME_T	43	-0.4887785	-1.6739472	0.0043956	0.021014	0.01262404	16600 tags=81%, list=45%, signal=45%
PID_FANCON	PID_FANCON	46	-0.4699151	-1.6380874	0.00441501	0.02106439	0.01265431	12429 tags=59%, list=34%, signal=39%
REACTOME_I	REACTOME_I	63	-0.4273618	-1.5833642	0.00456621	0.02169863	0.01303533	14461 tags=67%, list=39%, signal=41%
REACTOME_I	REACTOME_I	63	-0.4267518	-1.5811042	0.00456621	0.02169863	0.01303533	19017 tags=78%, list=52%, signal=38%
REACTOME_I	REACTOME_I	48	-0.467966	-1.6340909	0.00458716	0.02175466	0.01306899	2056 tags=27%, list=6%, signal=26%
REACTOME_S	REACTOME_S	60	-0.4385251	-1.6004156	0.0045977	0.02176123	0.01307294	18036 tags=85%, list=49%, signal=43%
WP_FOLATE_	WP_FOLATE_	70	-0.4401157	-1.6522326	0.00469484	0.0221768	0.01332258	4257 tags=29%, list=12%, signal=25%
KEGG_DRUG_	KEGG_DRUG_	71	-0.4399362	-1.650374	0.00475059	0.02239566	0.01345406	5905 tags=39%, list=16%, signal=33%
REACTOME_I	REACTOME_I	69	-0.4404195	-1.6513339	0.0047619	0.02240453	0.01345939	15324 tags=67%, list=42%, signal=39%
KEGG_PARKI	KEGG_PARKI	115	-0.3937021	-1.6148661	0.00479616	0.02248637	0.01350856	11534 tags=61%, list=31%, signal=42%
REACTOME_I	REACTOME_I	149	-0.3502995	-1.5079293	0.00480769	0.02248637	0.01350856	17862 tags=71%, list=48%, signal=37%
REACTOME_T	REACTOME_T	113	-0.4118689	-1.6881595	0.00480769	0.02248637	0.01350856	17329 tags=77%, list=47%, signal=41%
REACTOME_I	REACTOME_I	111	-0.417344	-1.7104178	0.00483092	0.02250639	0.01352059	12399 tags=73%, list=34%, signal=49%
REACTOME_I	REACTOME_I	110	-0.3838509	-1.572746	0.00483092	0.02250639	0.01352059	18420 tags=68%, list=50%, signal=34%
REACTOME_I	REACTOME_I	117	-0.3719064	-1.5276687	0.00487805	0.0226372	0.01359917	14195 tags=61%, list=39%, signal=37%
REACTOME_I	REACTOME_I	109	-0.3846565	-1.5716447	0.00487805	0.0226372	0.01359917	15423 tags=66%, list=42%, signal=39%
REACTOME_I	REACTOME_I	172	-0.3552185	-1.5682259	0.004914	0.0227596	0.0136727	15648 tags=74%, list=42%, signal=43%
REACTOME_I	REACTOME_I	135	-0.3547999	-1.497488	0.00493827	0.0228275	0.01371349	16600 tags=67%, list=45%, signal=37%
REACTOME_I	REACTOME_I	124	0.41098811	1.62773202	0.00500835	0.02310647	0.01388108	4260 tags=35%, list=12%, signal=31%
WP_HEPATIT	WP_HEPATIT	152	0.39926475	1.62433565	0.00502513	0.02313895	0.01390059	5992 tags=22%, list=16%, signal=19%
REACTOME_T	REACTOME_T	120	0.41601724	1.63889767	0.00506757	0.02328925	0.01399088	942 tags=11%, list=3%, signal=11%

WP_PRIMARY	WP_PRIMARY	72	0.47678176	1.71200668	0.00512821	0.02352242	0.01413096	7276	tags=42%, list=20%, signal=34%
KEGG_SMALL	KEGG_SMALL	84	0.46684458	1.72542967	0.00516351	0.02359327	0.01417352	4976	tags=25%, list=13%, signal=22%
WP_NONGEN	WP_NONGEN	71	0.48298279	1.72348611	0.00516351	0.02359327	0.01417352	8203	tags=42%, list=22%, signal=33%
REACTOME_(	REACTOME_(	75	0.47555229	1.71209918	0.00519031	0.02362487	0.0141925	6250	tags=41%, list=17%, signal=34%
REACTOME_I	REACTOME_I	86	0.45548387	1.69281861	0.00519031	0.02362487	0.0141925	3823	tags=17%, list=10%, signal=16%
KEGG_RENAL	KEGG_RENAL	70	0.48242664	1.71102402	0.00520833	0.02365748	0.0142121	6180	tags=27%, list=17%, signal=23%
REACTOME_I	REACTOME_I	92	0.45070395	1.6956196	0.00521739	0.02365748	0.0142121	3350	tags=14%, list=9%, signal=13%
PID_TRKR_PA	PID_TRKR_PA	61	0.47841687	1.65750482	0.00527241	0.02386141	0.01433461	10063	tags=44%, list=27%, signal=32%
PID_ANGIOP(	PID_ANGIOP(	49	0.52945622	1.76689198	0.00529101	0.02389685	0.0143559	5880	tags=37%, list=16%, signal=31%
REACTOME_I	REACTOME_I	48	0.54094736	1.79685875	0.00530035	0.02389685	0.0143559	9009	tags=54%, list=24%, signal=41%
REACTOME_S	REACTOME_S	249	-0.2990382	-1.3804497	0.00534759	0.02406417	0.01445641	6096	tags=24%, list=17%, signal=21%
WP_MECHAN	WP_MECHAN	47	0.55266456	1.81553962	0.00541516	0.02427628	0.01458383	5403	tags=30%, list=15%, signal=25%
WP_THYMIC_	WP_THYMIC_	47	0.55287187	1.81622065	0.00541516	0.02427628	0.01458383	8687	tags=51%, list=24%, signal=39%
REACTOME_(	REACTOME_(	35	0.60165832	1.89257872	0.00544465	0.02435716	0.01463242	7348	tags=51%, list=20%, signal=41%
WP_ENVELO	WP_ENVELO	46	0.54829862	1.7962576	0.00546448	0.02435716	0.01463242	4110	tags=24%, list=11%, signal=21%
PID_INTEGRI	PID_INTEGRI	43	0.56463915	1.82149316	0.00548446	0.02435716	0.01463242	5091	tags=65%, list=14%, signal=56%
PID_RAC1_RE	PID_RAC1_RE	38	0.59272101	1.88772932	0.00548446	0.02435716	0.01463242	2819	tags=29%, list=8%, signal=27%
WP_GENES_(	WP_GENES_(	38	0.59185661	1.88497634	0.00548446	0.02435716	0.01463242	4976	tags=47%, list=13%, signal=41%
BIOCARTA_A	BIOCARTA_A	37	0.58106231	1.83447035	0.00551471	0.02439926	0.01465772	5142	tags=35%, list=14%, signal=30%
WP_OXIDATI	WP_OXIDATI	40	0.58937035	1.88711996	0.00551471	0.02439926	0.01465772	3273	tags=30%, list=9%, signal=27%
BIOCARTA_A	BIOCARTA_A	33	0.60219625	1.8784714	0.00553506	0.02439926	0.01465772	6751	tags=42%, list=18%, signal=35%
WP_MONOA	WP_MONOA	33	0.58908504	1.83757271	0.00553506	0.02439926	0.01465772	3429	tags=36%, list=9%, signal=33%
WP_WHITE_I	WP_WHITE_I	32	0.59212756	1.82880562	0.00554529	0.02439926	0.01465772	7513	tags=56%, list=20%, signal=45%
WP_COMMC	WP_COMMC	42	0.57445663	1.83665672	0.00557621	0.02448996	0.01471221	5526	tags=29%, list=15%, signal=24%
REACTOME_I	REACTOME_I	29	0.63131583	1.90982853	0.00560748	0.02453658	0.01474021	5761	tags=34%, list=16%, signal=29%
WP_TCELL_R	WP_TCELL_R	29	0.62674462	1.8959999	0.00560748	0.02453658	0.01474021	8927	tags=48%, list=24%, signal=37%
BIOCARTA_V	BIOCARTA_V	26	0.6159652	1.82417885	0.00561798	0.02453734	0.01474067	5185	tags=27%, list=14%, signal=23%
REACTOME_S	REACTOME_S	28	0.63538678	1.90595509	0.00564972	0.02458558	0.01476965	6113	tags=39%, list=17%, signal=33%
WP_NANOPA	WP_NANOPA	28	0.61947807	1.85823411	0.00564972	0.02458558	0.01476965	4976	tags=32%, list=13%, signal=28%
BIOCARTA_G	BIOCARTA_G	27	0.6463772	1.92216225	0.00571429	0.02477581	0.01488393	8038	tags=52%, list=22%, signal=41%
REACTOME_I	REACTOME_I	27	0.64322805	1.91279746	0.00571429	0.02477581	0.01488393	9009	tags=70%, list=24%, signal=53%
WP_TRANSCI	WP_TRANSCI	22	0.66840634	1.90330328	0.00578035	0.02501658	0.01502857	7513	tags=55%, list=20%, signal=43%
BIOCARTA_C	BIOCARTA_C	20	0.70109229	1.94464906	0.00585938	0.02526656	0.01517874	7401	tags=70%, list=20%, signal=56%
REACTOME_I	REACTOME_I	20	0.70361177	1.95163743	0.00585938	0.02526656	0.01517874	2456	tags=40%, list=7%, signal=37%
WP_STEROID	WP_STEROID	10	-0.7413793	-1.707195	0.00588235	0.02531969	0.01521066	6192	tags=70%, list=17%, signal=58%
BIOCARTA_PI	BIOCARTA_PI	17	0.71256427	1.89091853	0.00592885	0.02533625	0.01522061	3376	tags=41%, list=9%, signal=37%
REACTOME_I	REACTOME_I	19	0.69050089	1.89169905	0.00592885	0.02533625	0.01522061	2191	tags=32%, list=6%, signal=30%
REACTOME_T	REACTOME_T	17	0.70089934	1.85996352	0.00592885	0.02533625	0.01522061	6729	tags=65%, list=18%, signal=53%
WP_LEPTIN_	WP_LEPTIN_	17	0.70735418	1.8770926	0.00592885	0.02533625	0.01522061	10400	tags=82%, list=28%, signal=59%
WP_CHOLEST	WP_CHOLEST	15	-0.6677659	-1.774864	0.0059761	0.02549229	0.01531435	9954	tags=80%, list=27%, signal=58%
REACTOME_I	REACTOME_I	12	0.73869068	1.81069857	0.00603622	0.0257026	0.01544069	2002	tags=42%, list=5%, signal=39%
REACTOME_I	REACTOME_I	10	0.79317246	1.85769699	0.00609756	0.02591736	0.01556971	3611	tags=50%, list=10%, signal=45%
REACTOME_I	REACTOME_I	292	0.32269501	1.40929769	0.00612557	0.02598994	0.01561331	5377	tags=19%, list=15%, signal=17%
REACTOME_(	REACTOME_(	385	-0.289866	-1.3900147	0.00632911	0.02680566	0.01610335	7840	tags=25%, list=21%, signal=20%
REACTOME_T	REACTOME_T	42	-0.479685	-1.641071	0.00646552	0.02733464	0.01642113	16644	tags=79%, list=45%, signal=43%
REACTOME_I	REACTOME_I	32	-0.5649349	-1.8168032	0.00650759	0.02742593	0.01647597	13242	tags=78%, list=36%, signal=50%
WP_MITOCH	WP_MITOCH	31	-0.5945669	-1.9002744	0.00652174	0.02742593	0.01647597	13329	tags=90%, list=36%, signal=58%
WP_TRANSSU	WP_TRANSSU	31	-0.5833848	-1.8645356	0.00652174	0.02742593	0.01647597	12080	tags=74%, list=33%, signal=50%
WP_G1_TO_S	WP_G1_TO_S	64	-0.4352663	-1.619511	0.00684932	0.0287526	0.01727296	13242	tags=56%, list=36%, signal=36%
REACTOME_A	REACTOME_A	55	-0.4390142	-1.5831183	0.00688073	0.02883355	0.01732159	18036	tags=84%, list=49%, signal=43%
REACTOME_I	REACTOME_I	75	0.45544983	1.63972564	0.00692042	0.02889667	0.01735951	6175	tags=31%, list=17%, signal=26%
REACTOME_I	REACTOME_I	76	-0.4162115	-1.5993435	0.00692841	0.02889667	0.01735951	13198	tags=59%, list=36%, signal=38%
KEGG_PROGI	KEGG_PROGI	85	0.43201846	1.59665912	0.00693241	0.02889667	0.01735951	5571	tags=26%, list=15%, signal=22%
KEGG_LONG_	KEGG_LONG_	70	0.4747296	1.68372488	0.00694444	0.02889667	0.01735951	6282	tags=33%, list=17%, signal=27%
WP_CARDIAC	WP_CARDIAC	53	0.51875683	1.75028446	0.0069808	0.0289509	0.01739209	4272	tags=40%, list=12%, signal=35%
WP_DNA_IRI	WP_DNA_IRI	80	-0.4104565	-1.5920879	0.00699301	0.0289509	0.01739209	15932	tags=62%, list=43%, signal=36%
REACTOME_A	REACTOME_A	65	-0.4244704	-1.5772933	0.00702576	0.0289509	0.01739209	14195	tags=74%, list=39%, signal=45%
REACTOME_I	REACTOME_I	65	-0.4130815	-1.5349734	0.00702576	0.0289509	0.01739209	18420	tags=74%, list=50%, signal=37%
REACTOME_A	REACTOME_A	94	0.42195136	1.5858884	0.00704225	0.0289509	0.01739209	6424	tags=29%, list=17%, signal=24%
REACTOME_I	REACTOME_I	91	0.43026097	1.61015453	0.00704225	0.0289509	0.01739209	8058	tags=42%, list=22%, signal=33%
REACTOME_I	REACTOME_I	55	0.49127694	1.66238377	0.00706714	0.0289509	0.01739209	4950	tags=29%, list=13%, signal=25%
WP_CARDIAC	WP_CARDIAC	55	0.488118	1.65169451	0.00706714	0.0289509	0.01739209	5648	tags=24%, list=15%, signal=20%
WP_MIRNAS_	WP_MIRNAS_	55	0.49176294	1.66402828	0.00706714	0.0289509	0.01739209	8255	tags=47%, list=22%, signal=37%
WP_COMPLE	WP_COMPLE	58	0.48754775	1.66719399	0.0071048	0.02902233	0.017435	3932	tags=40%, list=11%, signal=35%
REACTOME_I	REACTOME_I	68	-0.426939	-1.599191	0.007109	0.02902233	0.017435	14195	tags=63%, list=39%, signal=39%
PID_KIT_PAT	PID_KIT_PAT	52	0.5113528	1.71188129	0.00714286	0.02911051	0.01748797	9009	tags=42%, list=24%, signal=32%
KEGG_PRION	KEGG_PRION	35	0.5781628	1.81867113	0.00725953	0.02943454	0.01768263	4307	tags=34%, list=12%, signal=30%
WP_PHOTOD	WP_PHOTOD	35	0.58010332	1.82477523	0.00725953	0.02943454	0.01768263	8251	tags=54%, list=22%, signal=42%
WP_WNT_SI(	WP_WNT_SI(	36	0.56125618	1.76997439	0.00725953	0.02943454	0.01768263	4892	tags=31%, list=13%, signal=27%
REACTOME_V	REACTOME_V	43	0.53722834	1.73306749	0.00731261	0.0295203	0.01773415	5185	tags=37%, list=14%, signal=32%
BIOCARTA_F(	BIOCARTA_F(	39	0.58090208	1.84966861	0.00732601	0.0295203	0.01773415	9314	tags=49%, list=25%, signal=36%
WP_INTERAC	WP_INTERAC	39	0.56187225	1.78907514	0.00732601	0.0295203	0.01773415	6877	tags=56%, list=19%, signal=46%
PID_ILK_PATI	PID_ILK_PATI	45	0.53958598	1.75104515	0.00733945	0.0295203	0.01773415	2780	tags=22%, list=8%, signal=21%
REACTOME_I	REACTOME_I	40	0.56676168	1.81472866	0.00735294	0.0295203	0.01773415	5782	tags=42%, list=16%, signal=36%

BIOCARTA_B	BIOCARTA_B	33	0.56834929	1.77289028	0.00738007	0.0295203	0.01773415	9009	tags=42%, list=24%, signal=32%
REACTOME_	REACTOME_	33	0.57613756	1.79718475	0.00738007	0.0295203	0.01773415	4035	tags=61%, list=11%, signal=54%
WP_TYPE_I_	WP_TYPE_I_	33	0.57550304	1.79520545	0.00738007	0.0295203	0.01773415	5558	tags=42%, list=15%, signal=36%
PID_NETRIN_	PID_NETRIN_	32	0.58248542	1.79902555	0.00739372	0.02952516	0.01773707	6057	tags=34%, list=16%, signal=29%
PID_GLYPICA	PID_GLYPICA	27	0.61771662	1.83693294	0.00761905	0.03032304	0.0182164	4984	tags=52%, list=14%, signal=45%
SIG_IL4RECEI	SIG_IL4RECEI	27	0.62969335	1.87254869	0.00761905	0.03032304	0.0182164	8058	tags=41%, list=22%, signal=32%
WP_SIGNAL_	WP_SIGNAL_	25	0.60975306	1.78136731	0.00767754	0.03036839	0.01824364	5964	tags=40%, list=16%, signal=34%
BIOCARTA_TI	BIOCARTA_TI	21	0.66542441	1.87113379	0.00770713	0.03036839	0.01824364	6906	tags=62%, list=19%, signal=50%
PID_PDGFRA	PID_PDGFRA	22	0.63171577	1.79882598	0.00770713	0.03036839	0.01824364	9251	tags=68%, list=25%, signal=51%
REACTOME_I	REACTOME_I	21	0.65475865	1.8411423	0.00770713	0.03036839	0.01824364	4815	tags=48%, list=13%, signal=41%
REACTOME_S	REACTOME_S	21	0.65949	1.8544466	0.00770713	0.03036839	0.01824364	4976	tags=43%, list=13%, signal=37%
WP_PKCGAM	WP_PKCGAM	22	0.63181957	1.79912155	0.00770713	0.03036839	0.01824364	6282	tags=50%, list=17%, signal=42%
BIOCARTA_C	BIOCARTA_C	24	0.63321465	1.82476794	0.0077821	0.030512	0.01832991	5571	tags=42%, list=15%, signal=35%
REACTOME_I	REACTOME_I	24	0.63889253	1.8411302	0.0077821	0.030512	0.01832991	7174	tags=54%, list=19%, signal=44%
WP_GPCRS_	WP_GPCRS_	24	0.63037919	1.81659685	0.0077821	0.030512	0.01832991	6638	tags=54%, list=18%, signal=44%
REACTOME_	REACTOME_	20	0.67338419	1.86779395	0.0078125	0.03053043	0.01834098	6641	tags=65%, list=18%, signal=53%
REACTOME_I	REACTOME_I	20	0.68283158	1.89399855	0.0078125	0.03053043	0.01834098	5185	tags=40%, list=14%, signal=34%
WP_ACE_INH	WP_ACE_INH	17	0.67421872	1.78916166	0.00790514	0.03084172	0.01852799	4235	tags=47%, list=11%, signal=42%
WP_CELLTYP	WP_CELLTYP	13	0.71746721	1.81142081	0.00793651	0.03091335	0.01857102	2477	tags=46%, list=7%, signal=43%
WP_SIMPLIFI	WP_SIMPLIFI	18	0.68360026	1.84313366	0.00796813	0.03098571	0.01861449	2448	tags=33%, list=7%, signal=31%
WP_MAJOR_	WP_MAJOR_	15	0.68742583	1.77813778	0.008	0.03105882	0.01865841	8091	tags=67%, list=22%, signal=52%
REACTOME_I	REACTOME_I	19	-0.6255925	-1.7951887	0.00806452	0.03125822	0.0187782	458	tags=32%, list=1%, signal=31%
REACTOME_I	REACTOME_I	10	0.78184303	1.83116223	0.00813008	0.03146103	0.01890003	4462	tags=60%, list=12%, signal=53%
REACTOME_	REACTOME_	21	-0.61488	-1.8033242	0.00828157	0.03199515	0.0192209	2086	tags=29%, list=6%, signal=27%
KEGG_NEUR	KEGG_NEUR	126	0.39283513	1.55630353	0.00831947	0.03208937	0.01927751	5879	tags=21%, list=16%, signal=18%
REACTOME_	REACTOME_	31	-0.5646122	-1.8045371	0.00869565	0.03348601	0.02011653	10858	tags=58%, list=29%, signal=41%
REACTOME_	REACTOME_	43	0.53211991	1.71658799	0.00914077	0.03514315	0.02111204	6231	tags=47%, list=17%, signal=39%
REACTOME_I	REACTOME_I	54	-0.4312398	-1.5498167	0.00917431	0.03515833	0.02112117	18036	tags=83%, list=49%, signal=43%
ST_T_CELL_S	ST_T_CELL_S	45	0.53149036	1.72477354	0.00917431	0.03515833	0.02112117	5782	tags=33%, list=16%, signal=28%
REACTOME_I	REACTOME_I	61	-0.4151566	-1.5205092	0.00923788	0.03534491	0.02123326	18105	tags=82%, list=49%, signal=42%
WP_SPLICING	WP_SPLICING	42	0.5401231	1.72688531	0.00929368	0.03545337	0.02129841	7701	tags=40%, list=21%, signal=32%
PID_REG_GR	PID_REG_GR	82	-0.395739	-1.5397811	0.00930233	0.03545337	0.02129841	194	tags=7%, list=1%, signal=7%
PID_RAS_PAT	PID_RAS_PAT	30	0.59649196	1.81840468	0.00931099	0.03545337	0.02129841	5374	tags=33%, list=15%, signal=28%
REACTOME_I	REACTOME_I	29	0.61356177	1.85611974	0.00934579	0.03552897	0.02134383	2001	tags=24%, list=5%, signal=23%
PID_S1P_S1P	PID_S1P_S1P	24	0.62291292	1.79508092	0.00972763	0.03692147	0.02218036	6751	tags=42%, list=18%, signal=34%
BIOCARTA_IL	BIOCARTA_IL	16	0.67765234	1.77686493	0.00976563	0.03694765	0.02219609	8058	tags=75%, list=22%, signal=59%
REACTOME_I	REACTOME_I	16	0.67863795	1.77944929	0.00976563	0.03694765	0.02219609	4478	tags=50%, list=12%, signal=44%
REACTOME_	REACTOME_	11	-0.7265764	-1.7365254	0.00978474	0.0369611	0.02220417	3403	tags=55%, list=9%, signal=50%
BIOCARTA_IL	BIOCARTA_IL	19	0.67527642	1.84999001	0.00988142	0.03714915	0.02231714	6751	tags=53%, list=18%, signal=43%
REACTOME_I	REACTOME_I	17	0.67306898	1.78611062	0.00988142	0.03714915	0.02231714	5185	tags=41%, list=14%, signal=35%
REACTOME_S	REACTOME_S	19	0.66718393	1.8278198	0.00988142	0.03714915	0.02231714	2112	tags=32%, list=6%, signal=30%
REACTOME_I	REACTOME_I	13	0.71470228	1.80444009	0.00992063	0.03723764	0.0223703	1243	tags=15%, list=3%, signal=15%
BIOCARTA_IL	BIOCARTA_IL	15	0.68398857	1.76924676	0.01	0.03735849	0.0224429	4224	tags=60%, list=11%, signal=53%
BIOCARTA_L	BIOCARTA_L	15	0.68414885	1.76966133	0.01	0.03735849	0.0224429	1801	tags=27%, list=5%, signal=25%
KEGG_SYSTE	KEGG_SYSTE	134	0.37583076	1.50423476	0.01	0.03735849	0.0224429	5996	tags=28%, list=16%, signal=23%
WP_MRNA_	WP_MRNA_	127	-0.3539619	-1.4785886	0.01002506	0.03739333	0.02246383	17806	tags=74%, list=48%, signal=38%
REACTOME_	REACTOME_	122	0.39517585	1.5600996	0.01011804	0.0376698	0.02262992	6330	tags=28%, list=17%, signal=23%
REACTOME_I	REACTOME_I	10	0.75494994	1.76817568	0.0101626	0.0376698	0.02262992	8596	tags=90%, list=23%, signal=69%
REACTOME_I	REACTOME_I	10	0.76994238	1.8032896	0.0101626	0.0376698	0.02262992	4595	tags=60%, list=12%, signal=53%
WP_LEPTIN_	WP_LEPTIN_	10	0.76822994	1.79927888	0.0101626	0.0376698	0.02262992	2606	tags=30%, list=7%, signal=28%
REACTOME_	REACTOME_	105	0.3952334	1.51477613	0.01023891	0.03789353	0.02276432	2502	tags=13%, list=7%, signal=12%
KEGG_ADHE	KEGG_ADHE	73	0.44584001	1.5991845	0.01036269	0.03829201	0.02300371	5073	tags=26%, list=14%, signal=22%
WP_METAPA	WP_METAPA	184	-0.3083863	-1.367962	0.01049869	0.03873429	0.0232694	5905	tags=32%, list=16%, signal=27%
KEGG_NOD_	KEGG_NOD_	62	0.45139491	1.56871025	0.01052632	0.03877601	0.02329447	5265	tags=27%, list=14%, signal=24%
PID_PI3KCI_	PID_PI3KCI_	48	0.51673456	1.71643137	0.01060071	0.0389896	0.02342278	9037	tags=50%, list=25%, signal=38%
KEGG_TYPE_	KEGG_TYPE_	47	0.51667435	1.69730939	0.01083032	0.03965	0.02381951	2367	tags=21%, list=6%, signal=20%
PID_HEDGEH	PID_HEDGEH	47	0.52820416	1.73518559	0.01083032	0.03965	0.02381951	5852	tags=26%, list=16%, signal=22%
ST_DIFFEREN	ST_DIFFEREN	47	0.5241785	1.72196103	0.01083032	0.03965	0.02381951	7401	tags=34%, list=20%, signal=27%
KEGG_CITRA	KEGG_CITRA	31	-0.5336893	-1.7057055	0.01086957	0.03973244	0.02386904	14013	tags=77%, list=38%, signal=48%
PID_ARF6_P	PID_ARF6_P	35	0.55716209	1.75261121	0.01088929	0.03974341	0.02387562	4661	tags=29%, list=13%, signal=25%
REACTOME_I	REACTOME_I	37	-0.5070479	-1.6847674	0.01091703	0.03978353	0.02389973	16600	tags=86%, list=45%, signal=48%
REACTOME_I	REACTOME_I	39	-0.4797731	-1.6107594	0.01096491	0.03985039	0.0239399	12375	tags=54%, list=34%, signal=36%
WP_SLEEP_R	WP_SLEEP_R	38	0.53681279	1.70966987	0.01096892	0.03985039	0.0239399	5178	tags=34%, list=14%, signal=29%
REACTOME_I	REACTOME_I	31	0.58162787	1.78927808	0.01107011	0.0400954	0.02408708	2921	tags=26%, list=8%, signal=24%
REACTOME_I	REACTOME_I	31	0.57461066	1.76769082	0.01107011	0.0400954	0.02408708	2339	tags=23%, list=6%, signal=21%
WP_CELL_MI	WP_CELL_MI	30	0.59180225	1.80410811	0.01117318	0.04040713	0.02427435	3297	tags=27%, list=9%, signal=24%
PID_ALK1_P	PID_ALK1_P	26	0.59757418	1.76971391	0.01123596	0.04051082	0.02433664	5142	tags=38%, list=14%, signal=33%
PID_VEGFR1_	PID_VEGFR1_	26	0.60341132	1.78700057	0.01123596	0.04051082	0.02433664	5571	tags=38%, list=15%, signal=33%
PID_TCR_CA	PID_TCR_CA	28	0.58248142	1.7472561	0.01129944	0.04061643	0.02440009	8606	tags=68%, list=23%, signal=52%
REACTOME_	REACTOME_	28	0.57330681	1.71973524	0.01129944	0.04061643	0.02440009	5782	tags=39%, list=16%, signal=33%
REACTOME_I	REACTOME_I	23	0.61400733	1.76504393	0.01142857	0.04101856	0.02464166	2442	tags=30%, list=7%, signal=28%
WP_PHYSIOL	WP_PHYSIOL	25	0.59155438	1.72820063	0.01151631	0.04122481	0.02476557	5504	tags=36%, list=15%, signal=31%
REACTOME_	REACTOME_	91	-0.3713787	-1.4743453	0.01152074	0.04122481	0.02476557	16600	tags=68%, list=45%, signal=38%
REACTOME_I	REACTOME_I	21	0.63960491	1.7985309	0.01156069	0.04130558	0.02481409	3592	tags=38%, list=10%, signal=34%

WP_SELENIU	WP_SELENIU	89	-0.37558	-1.4788767	0.01160093	0.04138709	0.02486306	4241	tags=22%, list=12%, signal=20%
REACTOME_I	REACTOME_I	16	0.67348381	1.76593467	0.01171875	0.04161996	0.02500295	1243	tags=12%, list=3%, signal=12%
WP_BMP_SI(	WP_BMP_SI(	20	0.6482771	1.79815336	0.01171875	0.04161996	0.02500295	1373	tags=20%, list=4%, signal=19%
WP_GENES_1	WP_GENES_1	16	0.67014276	1.75717416	0.01171875	0.04161996	0.02500295	6745	tags=62%, list=18%, signal=51%
REACTOME_(	REACTOME_(	11	-0.6865164	-1.6407817	0.01174168	0.04163916	0.02501449	10694	tags=91%, list=29%, signal=65%
REACTOME_1	REACTOME_1	85	-0.3892081	-1.5212942	0.01176471	0.04165863	0.02502618	6304	tags=29%, list=17%, signal=24%
BIOCARTA_PI	BIOCARTA_PI	14	0.69059332	1.77916813	0.01185771	0.04186317	0.02514906	5851	tags=71%, list=16%, signal=60%
REACTOME_/	REACTOME_/	14	0.68928232	1.77579061	0.01185771	0.04186317	0.02514906	5236	tags=57%, list=14%, signal=49%
REACTOME_(	REACTOME_(	84	-0.3884321	-1.5096374	0.01187648	0.04186725	0.02515151	18420	tags=70%, list=50%, signal=35%
REACTOME_I	REACTOME_I	13	0.70688961	1.7847151	0.01190476	0.04190476	0.02517405	1243	tags=15%, list=3%, signal=15%
WP_MECP2_	WP_MECP2_	72	0.43182626	1.55058247	0.01196581	0.04205735	0.02526571	3597	tags=19%, list=10%, signal=18%
WP_TFS_REG	WP_TFS_REG	12	0.71985472	1.76452736	0.01207243	0.04236943	0.02545319	5180	tags=33%, list=14%, signal=29%
REACTOME_I	REACTOME_I	14	-0.6467366	-1.6730328	0.01209677	0.04237863	0.02545872	1973	tags=43%, list=5%, signal=41%
WP_NCRNAS	WP_NCRNAS	86	0.40376175	1.5005919	0.01211073	0.04237863	0.02545872	5091	tags=26%, list=14%, signal=22%
WP_CELL_CY	WP_CELL_CY	120	-0.3527006	-1.4562639	0.01219512	0.0425224	0.02554509	14176	tags=56%, list=38%, signal=34%
REACTOME_(	REACTOME_(	11	0.75466746	1.81028053	0.01221996	0.0425224	0.02554509	5571	tags=64%, list=15%, signal=54%
REACTOME_I	REACTOME_I	151	-0.3335119	-1.4356928	0.01222494	0.0425224	0.02554509	9954	tags=41%, list=27%, signal=30%
REACTOME_I	REACTOME_I	59	0.45836127	1.57710929	0.01225919	0.0425224	0.02554509	6910	tags=34%, list=19%, signal=28%
REACTOME_S	REACTOME_S	50	0.49792964	1.6688665	0.01225919	0.0425224	0.02554509	2921	tags=18%, list=8%, signal=17%
WP_KIT_REC	WP_KIT_REC	59	0.45802361	1.57594748	0.01225919	0.0425224	0.02554509	9009	tags=41%, list=24%, signal=31%
WP_SYNAPTI	WP_SYNAPTI	51	0.4845086	1.61506217	0.01245552	0.04314039	0.02591634	5658	tags=25%, list=15%, signal=22%
REACTOME_1	REACTOME_1	23	-0.5689252	-1.7075389	0.01257862	0.04344011	0.0260964	4436	tags=39%, list=12%, signal=34%
REACTOME_1	REACTOME_1	23	-0.5724744	-1.7181912	0.01257862	0.04344011	0.0260964	1796	tags=22%, list=5%, signal=21%
PID_NFAT_TF	PID_NFAT_TF	45	0.50325628	1.63314932	0.01284404	0.04429235	0.02660838	8471	tags=60%, list=23%, signal=46%
WP_TOLLLIKE	WP_TOLLLIKE	31	0.56987889	1.75313435	0.01291513	0.0444264	0.02668891	6225	tags=32%, list=17%, signal=27%
WP_METABC	WP_METABC	42	-0.4615342	-1.5789745	0.01293103	0.0444264	0.02668891	12316	tags=64%, list=33%, signal=43%
PID_WNT_N(	PID_WNT_N(	32	0.56381595	1.74136426	0.012939	0.0444264	0.02668891	4079	tags=25%, list=11%, signal=22%
KEGG_ALDOS	KEGG_ALDOS	42	0.50182804	1.60444807	0.01301115	0.04448129	0.02672188	2179	tags=24%, list=6%, signal=22%
PID_TCPTP_F	PID_TCPTP_F	42	0.51101558	1.63382255	0.01301115	0.04448129	0.02672188	7401	tags=43%, list=20%, signal=34%
REACTOME_/	REACTOME_/	42	0.50363654	1.61023022	0.01301115	0.04448129	0.02672188	5468	tags=38%, list=15%, signal=32%
WP_EICOSAN	WP_EICOSAN	30	0.58021275	1.76877753	0.01303538	0.0445001	0.02673318	1821	tags=23%, list=5%, signal=22%
WP_CYTOKIN	WP_CYTOKIN	26	0.59197813	1.75314124	0.01310861	0.04468589	0.02684479	8305	tags=73%, list=23%, signal=57%
KEGG_AMIN(	KEGG_AMIN(	41	-0.4639378	-1.5745876	0.01318681	0.04482385	0.02692767	18750	tags=88%, list=51%, signal=43%
REACTOME_I	REACTOME_I	41	-0.4691694	-1.5923436	0.01318681	0.04482385	0.02692767	16600	tags=76%, list=45%, signal=42%
REACTOME_1	REACTOME_1	36	-0.4975573	-1.6433017	0.01330377	0.04515679	0.02712769	16600	tags=83%, list=45%, signal=46%
BIOCARTA_R.	BIOCARTA_R.	21	0.62190367	1.74875606	0.01348748	0.04558498	0.02738492	5665	tags=38%, list=15%, signal=32%
REACTOME_I	REACTOME_I	22	0.59629265	1.69795779	0.01348748	0.04558498	0.02738492	6521	tags=41%, list=18%, signal=34%
REACTOME_I	REACTOME_I	21	0.61512876	1.72970543	0.01348748	0.04558498	0.02738492	2442	tags=33%, list=7%, signal=31%
REACTOME_S	REACTOME_S	20	0.61205618	1.69768587	0.01367188	0.04607713	0.02768057	2191	tags=20%, list=6%, signal=19%
REACTOME_S	REACTOME_S	16	0.66018459	1.73106294	0.01367188	0.04607713	0.02768057	8131	tags=50%, list=22%, signal=39%
REACTOME_(	REACTOME_(	10	-0.7215641	-1.661566	0.01372549	0.0461923	0.02774976	716	tags=60%, list=2%, signal=59%
KEGG_STERO	KEGG_STERO	55	-0.4259933	-1.5361642	0.01376147	0.04624788	0.02778315	5274	tags=42%, list=14%, signal=36%
REACTOME_S	REACTOME_S	57	-0.4315635	-1.5568681	0.01382488	0.04638338	0.02786455	18036	tags=81%, list=49%, signal=41%
WP_WNT_SI(	WP_WNT_SI(	101	0.3947058	1.50096026	0.01384083	0.04638338	0.02786455	5091	tags=23%, list=14%, signal=20%
REACTOME_I	REACTOME_I	59	-0.4189229	-1.5208085	0.01392111	0.04649297	0.02793039	15617	tags=76%, list=42%, signal=44%
PID_ANTHRA	PID_ANTHRA	18	0.66165022	1.78395161	0.01394422	0.04649297	0.02793039	2828	tags=28%, list=8%, signal=26%
REACTOME_1	REACTOME_1	78	-0.3854362	-1.4892804	0.01395349	0.04649297	0.02793039	18883	tags=73%, list=51%, signal=36%
KEGG_ANTIG	KEGG_ANTIG	81	0.41608187	1.51749077	0.01396161	0.04649297	0.02793039	5871	tags=30%, list=16%, signal=25%
WP_ASSOCIA	WP_ASSOCIA	66	0.44426045	1.55810409	0.01398601	0.04649297	0.02793039	3360	tags=20%, list=9%, signal=18%
REACTOME_I	REACTOME_I	215	-0.2950199	-1.3344695	0.0140056	0.04649297	0.02793039	10356	tags=46%, list=28%, signal=33%
KEGG_AUTOI	KEGG_AUTOI	50	0.4812541	1.61297657	0.01401051	0.04649297	0.02793039	7324	tags=50%, list=20%, signal=40%
REACTOME_I	REACTOME_I	13	-0.668862	-1.6931372	0.01405622	0.04657962	0.02798244	4397	tags=46%, list=12%, signal=41%
REACTOME_1	REACTOME_1	70	-0.4047019	-1.5192863	0.01408451	0.04660834	0.02799969	19119	tags=76%, list=52%, signal=37%
REACTOME_I	REACTOME_I	19	-0.5808933	-1.6669206	0.0141129	0.04663735	0.02801712	1975	tags=21%, list=5%, signal=20%
REACTOME_(	REACTOME_(	68	-0.4015162	-1.5039646	0.01421801	0.04678692	0.02810697	15617	tags=66%, list=42%, signal=38%
SIG_INSULIN	SIG_INSULIN_	51	0.47722272	1.5907754	0.01423488	0.04678692	0.02810697	5571	tags=22%, list=15%, signal=18%
BIOCARTA_IL	BIOCARTA_IL	11	0.73314358	1.75864952	0.01425662	0.04678692	0.02810697	8325	tags=82%, list=23%, signal=63%
BIOCARTA_V	BIOCARTA_V	11	0.74375391	1.78410136	0.01425662	0.04678692	0.02810697	4600	tags=64%, list=12%, signal=56%
REACTOME_S	REACTOME_S	11	0.73816017	1.7706832	0.01425662	0.04678692	0.02810697	5088	tags=55%, list=14%, signal=47%
REACTOME_(	REACTOME_(	25	-0.5397205	-1.6704484	0.01455301	0.04769374	0.02865174	7208	tags=48%, list=20%, signal=39%
PID_AR_NON	PID_AR_NON	31	0.55267206	1.7002005	0.01476015	0.04830594	0.02901952	5571	tags=29%, list=15%, signal=25%
WP_MONOA	WP_MONOA	32	0.55839339	1.7246165	0.01478743	0.04832866	0.02903317	5458	tags=44%, list=15%, signal=37%
PID_BMP_PA	PID_BMP_PA	42	0.49928888	1.59632986	0.01486989	0.04852495	0.02915109	5332	tags=29%, list=14%, signal=24%
KEGG_CELL_(	KEGG_CELL_(	124	-0.3535006	-1.4693647	0.01488834	0.04852495	0.02915109	14176	tags=56%, list=38%, signal=34%
REACTOME_(	REACTOME_(	29	-0.5389341	-1.7009461	0.01498929	0.04878707	0.02930855	15509	tags=86%, list=42%, signal=50%
BIOCARTA_N	BIOCARTA_N	28	0.56971778	1.7089693	0.01506591	0.04896834	0.02941745	5418	tags=43%, list=15%, signal=37%
REACTOME_I	REACTOME_I	42	-0.457742	-1.5660008	0.01508621	0.04896834	0.02941745	17600	tags=81%, list=48%, signal=42%
WP_STATIN_	WP_STATIN_	31	-0.5215883	-1.6670298	0.01521739	0.04932677	0.02963277	5379	tags=45%, list=15%, signal=39%
REACTOME_I	REACTOME_I	34	-0.5023202	-1.6346501	0.01531729	0.04958293	0.02978666	16600	tags=85%, list=45%, signal=47%
WP_15Q133_	WP_15Q133_	21	0.60557392	1.70283777	0.01541426	0.04982895	0.02993446	7884	tags=62%, list=21%, signal=49%
KEGG_PROTE	KEGG_PROTE	46	-0.4479559	-1.5615393	0.01545254	0.04988483	0.02996802	15617	tags=78%, list=42%, signal=45%
WP_AMPACT	WP_AMPACT	68	0.43959741	1.55082976	0.01551724	0.05002573	0.03005267	4105	tags=18%, list=11%, signal=16%
REACTOME_S	REACTOME_S	19	0.64482643	1.76656911	0.01581028	0.05090138	0.03057871	2951	tags=58%, list=8%, signal=53%
WP_REGULA	WP_REGULA	15	-0.6244707	-1.6597891	0.01593625	0.05123754	0.03078066	9799	tags=67%, list=27%, signal=49%

REACTOME_(	REACTOME_(	12	0.70279055	1.72269922	0.01609658	0.05168307	0.03104831	5996	tags=58%, list=16%, signal=49%
WP_PREIMPL	WP_PREIMPL	59	-0.4125748	-1.4977633	0.0162413	0.05207736	0.03128518	6143	tags=36%, list=17%, signal=30%
BIOCARTA_M	BIOCARTA_M	11	0.71703165	1.72000056	0.01629328	0.05216322	0.03133676	665	tags=18%, list=2%, signal=18%
REACTOME_I	REACTOME_I	80	-0.3788029	-1.4693089	0.01631702	0.05216322	0.03133676	14195	tags=56%, list=39%, signal=35%
SIG_PIP3_SIG	SIG_PIP3_SIG	36	0.51956881	1.63850934	0.01633394	0.05216322	0.03133676	4244	tags=31%, list=12%, signal=27%
WP_FACTORS	WP_FACTORS	37	0.52047337	1.64318516	0.01654412	0.05274491	0.0316862	2079	tags=14%, list=6%, signal=13%
KEGG_ABC_T	KEGG_ABC_T	44	0.49371777	1.59773285	0.01657459	0.05274491	0.0316862	3765	tags=25%, list=10%, signal=22%
PID_AVB3_O	PID_AVB3_O	31	0.5451973	1.67720568	0.01660517	0.05274491	0.0316862	7348	tags=39%, list=20%, signal=31%
REACTOME_T	REACTOME_T	31	0.53539181	1.64704079	0.01660517	0.05274491	0.0316862	6781	tags=39%, list=18%, signal=32%
REACTOME_I	REACTOME_I	71	-0.3894141	-1.4608458	0.01662708	0.05274491	0.0316862	2088	tags=18%, list=6%, signal=17%
REACTOME_I	REACTOME_I	69	-0.3931552	-1.4741185	0.01666667	0.0528	0.0317193	15405	tags=55%, list=42%, signal=32%
WP_IL2_SIGN	WP_IL2_SIGN	42	0.49194045	1.57283541	0.01672862	0.0528133	0.03172729	9009	tags=43%, list=24%, signal=32%
PID_IL2_STAT	PID_IL2_STAT	30	0.56638601	1.72662675	0.01675978	0.0528133	0.03172729	9009	tags=63%, list=24%, signal=48%
PID_P38_ALP	PID_P38_ALP	30	0.56728362	1.72936311	0.01675978	0.0528133	0.03172729	4011	tags=30%, list=11%, signal=27%
REACTOME_I	REACTOME_I	30	0.55121179	1.68036818	0.01675978	0.0528133	0.03172729	2993	tags=27%, list=8%, signal=25%
WP_PDGFRB	WP_PDGFRB	29	0.55078279	1.66620356	0.01682243	0.05294052	0.03180372	10122	tags=52%, list=27%, signal=38%
REACTOME_(	REACTOME_(	28	0.56343028	1.69010883	0.01694915	0.0531984	0.03195863	4338	tags=29%, list=12%, signal=25%
REACTOME_I	REACTOME_I	28	0.56205981	1.68599788	0.01694915	0.0531984	0.03195863	4260	tags=43%, list=12%, signal=38%
REACTOME_I	REACTOME_I	146	0.35502138	1.42965783	0.01706485	0.05349086	0.03213433	4787	tags=19%, list=13%, signal=17%
REACTOME_I	REACTOME_I	34	-0.5004946	-1.6287091	0.01750547	0.05479973	0.03292063	11053	tags=56%, list=30%, signal=39%
WP_IMATINI	WP_IMATINI	20	0.60331103	1.67342909	0.01757813	0.05495477	0.03301376	5460	tags=45%, list=15%, signal=38%
KEGG_RENIN	KEGG_RENIN	17	0.65784851	1.74572032	0.01778656	0.05531527	0.03323033	3635	tags=41%, list=10%, signal=37%
PID_INTEGRI	PID_INTEGRI	17	0.65287405	1.7325197	0.01778656	0.05531527	0.03323033	5403	tags=71%, list=15%, signal=60%
REACTOME_A	REACTOME_A	14	0.67342111	1.73492755	0.01778656	0.05531527	0.03323033	5418	tags=43%, list=15%, signal=37%
REACTOME_I	REACTOME_I	14	0.67299765	1.73383659	0.01778656	0.05531527	0.03323033	6521	tags=50%, list=18%, signal=41%
BIOCARTA_FI	BIOCARTA_FI	12	-0.6623001	-1.6411964	0.01782178	0.05535236	0.03325261	1433	tags=50%, list=4%, signal=48%
REACTOME_I	REACTOME_I	12	0.70016109	1.71625381	0.01810865	0.05616992	0.03374376	9009	tags=83%, list=24%, signal=63%
REACTOME_I	REACTOME_I	55	-0.4187139	-1.5099139	0.01834862	0.05669224	0.03405754	18036	tags=80%, list=49%, signal=41%
REACTOME_I	REACTOME_I	48	-0.4284783	-1.4962038	0.01834862	0.05669224	0.03405754	13576	tags=52%, list=37%, signal=33%
WP_TYPE_2_	WP_TYPE_2_	34	0.53530406	1.67840649	0.01834862	0.05669224	0.03405754	4952	tags=26%, list=13%, signal=23%
REACTOME_I	REACTOME_I	49	-0.4352659	-1.526899	0.0183908	0.05674877	0.0340915	4511	tags=27%, list=12%, signal=23%
WP_MESODE	WP_MESODE	152	0.35260071	1.43449153	0.01842546	0.05678196	0.03411144	6717	tags=30%, list=18%, signal=24%
REACTOME_I	REACTOME_I	33	0.54140702	1.68884743	0.01845018	0.05678451	0.03411297	2921	tags=21%, list=8%, signal=20%
REACTOME_T	REACTOME_T	358	-0.2671199	-1.2717208	0.01851852	0.05692109	0.03419502	17304	tags=56%, list=47%, signal=30%
KEGG_RNA_[	KEGG_RNA_[	59	-0.4090535	-1.48498	0.01856148	0.056966	0.034222	18035	tags=71%, list=49%, signal=36%
REACTOME_(	REACTOME_(	145	0.35406173	1.4289934	0.01858108	0.056966	0.034222	2502	tags=11%, list=7%, signal=10%
REACTOME_(	REACTOME_(	22	-0.5526887	-1.6480089	0.01863354	0.05697978	0.03423028	2941	tags=41%, list=8%, signal=38%
REACTOME_I	REACTOME_I	21	-0.5558886	-1.6303139	0.01863354	0.05697978	0.03423028	12507	tags=71%, list=34%, signal=47%
KEGG_ASCOI	KEGG_ASCOI	25	-0.5309835	-1.6434071	0.01871102	0.05714316	0.03432843	7804	tags=52%, list=21%, signal=41%
REACTOME_I	REACTOME_I	87	-0.3693916	-1.448602	0.01873536	0.05714406	0.03432897	18682	tags=77%, list=51%, signal=38%
REACTOME_(	REACTOME_(	28	0.55908444	1.67707273	0.01883239	0.05736636	0.03446251	7017	tags=50%, list=19%, signal=41%
REACTOME_I	REACTOME_I	24	0.58122408	1.67494398	0.01945525	0.05918781	0.03555674	2339	tags=25%, list=6%, signal=23%
REACTOME_I	REACTOME_I	32	-0.5102424	-1.6409147	0.01952278	0.05931729	0.03563452	1973	tags=34%, list=5%, signal=33%
BIOCARTA_G	BIOCARTA_G	13	0.67530345	1.70496814	0.01984127	0.0601312	0.03612347	2459	tags=38%, list=7%, signal=36%
BIOCARTA_IL	BIOCARTA_IL	13	0.67145479	1.69525125	0.01984127	0.0601312	0.03612347	8495	tags=69%, list=23%, signal=53%
BIOCARTA_C.	BIOCARTA_C.	18	0.63596061	1.71468688	0.01992032	0.06014063	0.03612914	5504	tags=39%, list=15%, signal=33%
REACTOME_I	REACTOME_I	15	-0.6149335	-1.6344402	0.01992032	0.06014063	0.03612914	10777	tags=80%, list=29%, signal=57%
WP_MITOCH	WP_MITOCH	15	-0.6175486	-1.6413907	0.01992032	0.06014063	0.03612914	12967	tags=87%, list=35%, signal=56%
REACTOME_S	REACTOME_S	35	-0.4822377	-1.5824362	0.01995565	0.06017086	0.0361473	16600	tags=86%, list=45%, signal=47%
BIOCARTA_IL	BIOCARTA_IL	37	0.51352289	1.62124184	0.02022059	0.06089242	0.03658077	9379	tags=51%, list=25%, signal=38%
WP_REGUCA	WP_REGUCA	33	0.53091286	1.6561123	0.0202952	0.06103975	0.03666928	4535	tags=24%, list=12%, signal=21%
BIOCARTA_IL	BIOCARTA_IL	11	0.70134594	1.68237399	0.0203666	0.06117704	0.03675176	9009	tags=64%, list=24%, signal=48%
WP_OLIGODI	WP_OLIGODI	29	0.5451712	1.64922764	0.02056075	0.06168224	0.03705525	4373	tags=31%, list=12%, signal=27%
REACTOME_I	REACTOME_I	23	0.58442027	1.67999208	0.02095238	0.06277788	0.03771345	5571	tags=35%, list=15%, signal=30%
WP_HAIR_FC	WP_HAIR_FC	87	-0.3660694	-1.4355738	0.02107728	0.06307258	0.03789049	2682	tags=26%, list=7%, signal=25%
REACTOME_(	REACTOME_(	85	-0.3715389	-1.4522307	0.02117647	0.06320522	0.03797017	17260	tags=73%, list=47%, signal=39%
WP_NICOTIN	WP_NICOTIN	21	0.5776808	1.62440399	0.02119461	0.06320522	0.03797017	5236	tags=38%, list=14%, signal=33%
REACTOME_I	REACTOME_I	54	0.46089355	1.55780667	0.02120141	0.06320522	0.03797017	7348	tags=37%, list=20%, signal=30%
PID_NOTCH_	PID_NOTCH_	58	0.44770857	1.53096192	0.02131439	0.06346239	0.03812467	6106	tags=26%, list=17%, signal=22%
REACTOME_T	REACTOME_T	24	0.57366747	1.65316769	0.02140078	0.06359743	0.03820579	4356	tags=33%, list=12%, signal=29%
REACTOME_I	REACTOME_I	29	-0.5124477	-1.6173515	0.02141328	0.06359743	0.03820579	13213	tags=69%, list=36%, signal=44%
REACTOME_I	REACTOME_I	30	-0.5106804	-1.6234112	0.02150538	0.06379123	0.03832222	12740	tags=60%, list=35%, signal=39%
BIOCARTA_P.	BIOCARTA_P.	19	0.62713403	1.71809893	0.02173913	0.06392596	0.03840315	1801	tags=26%, list=5%, signal=25%
REACTOME_I	REACTOME_I	17	0.63588158	1.6874271	0.02173913	0.06392596	0.03840315	7174	tags=59%, list=19%, signal=47%
REACTOME_I	REACTOME_I	14	0.66314666	1.70845761	0.02173913	0.06392596	0.03840315	3922	tags=64%, list=11%, signal=57%
REACTOME_I	REACTOME_I	19	0.62390925	1.70926431	0.02173913	0.06392596	0.03840315	3546	tags=37%, list=10%, signal=33%
REACTOME_S	REACTOME_S	14	0.66547007	1.71444336	0.02173913	0.06392596	0.03840315	5880	tags=57%, list=16%, signal=48%
SA_PTEN_PA	SA_PTEN_PA	17	0.6355969	1.68667166	0.02173913	0.06392596	0.03840315	3184	tags=24%, list=9%, signal=22%
WP_NOTCH1	WP_NOTCH1	17	0.64292023	1.70610543	0.02173913	0.06392596	0.03840315	6469	tags=53%, list=18%, signal=44%
WP_SRF_ANI	WP_SRF_ANI	12	0.69301055	1.69872621	0.0221328	0.06500312	0.03905025	6175	tags=58%, list=17%, signal=49%
REACTOME_I	REACTOME_I	36	-0.4706914	-1.5545705	0.02217295	0.06504065	0.0390728	16600	tags=78%, list=45%, signal=43%
KEGG_COMP	KEGG_COMP	69	0.42516382	1.50639457	0.02233677	0.06542112	0.03930137	3932	tags=38%, list=11%, signal=34%
REACTOME_I	REACTOME_I	10	0.71053527	1.66415163	0.02235772	0.06542112	0.03930137	8471	tags=80%, list=23%, signal=62%
REACTOME_I	REACTOME_I	26	0.56600184	1.67621252	0.02247191	0.06567436	0.0394535	5701	tags=35%, list=15%, signal=29%

BIOCARTA_PI	BIOCARTA_PI	28	0.54663376	1.63972471	0.02259887	0.06596427	0.03962766	9024	tags=50%, list=24%, signal=38%
REACTOME_I	REACTOME_I	23	0.56869787	1.634796	0.02285714	0.06663628	0.04003137	2921	tags=26%, list=8%, signal=24%
BIOCARTA_N	BIOCARTA_N	21	0.57406403	1.61423386	0.02312139	0.06732404	0.04044453	11617	tags=67%, list=32%, signal=46%
REACTOME_(	REACTOME_(	20	0.58547024	1.62394334	0.0234375	0.06799451	0.04084731	3546	tags=35%, list=10%, signal=32%
REACTOME_I	REACTOME_I	20	0.59044536	1.63774304	0.0234375	0.06799451	0.04084731	5265	tags=40%, list=14%, signal=34%
REACTOME_I	REACTOME_I	20	0.58934705	1.63469659	0.0234375	0.06799451	0.04084731	2442	tags=30%, list=7%, signal=28%
REACTOME_I	REACTOME_I	101	-0.3513074	-1.4152736	0.02358491	0.0683387	0.04105408	18036	tags=74%, list=49%, signal=38%
WP_FRAGILE	WP_FRAGILE	120	0.35820389	1.41114228	0.02364865	0.06843994	0.0411149	7062	tags=25%, list=19%, signal=20%
REACTOME_I	REACTOME_I	40	0.48692961	1.55911234	0.02389706	0.06895314	0.04142321	8186	tags=45%, list=22%, signal=35%
REACTOME_I	REACTOME_I	33	-0.4891785	-1.582201	0.02391304	0.06895314	0.04142321	16600	tags=88%, list=45%, signal=48%
REACTOME_(	REACTOME_(	33	-0.4922433	-1.5921139	0.02391304	0.06895314	0.04142321	14464	tags=79%, list=39%, signal=48%
BIOCARTA_M	BIOCARTA_M	33	0.51741581	1.61401005	0.02398524	0.06895443	0.04142398	8927	tags=42%, list=24%, signal=32%
REACTOME_(	REACTOME_(	33	0.51646144	1.61103302	0.02398524	0.06895443	0.04142398	5996	tags=33%, list=16%, signal=28%
REACTOME_I	REACTOME_I	37	-0.4635618	-1.540276	0.02401747	0.06895443	0.04142398	16600	tags=76%, list=45%, signal=42%
REACTOME_T	REACTOME_T	32	0.52638947	1.62577132	0.02402957	0.06895443	0.04142398	6098	tags=38%, list=17%, signal=31%
BIOCARTA_LE	BIOCARTA_LE	12	0.68269618	1.67344335	0.02414487	0.06913408	0.0415319	2951	tags=42%, list=8%, signal=38%
WP_TNF_REL	WP_TNF_REL	42	0.4733003	1.51323898	0.02416357	0.06913408	0.0415319	8255	tags=36%, list=22%, signal=28%
REACTOME_I	REACTOME_I	19	-0.5484412	-1.5737967	0.02419355	0.06913408	0.0415319	2088	tags=32%, list=6%, signal=30%
BIOCARTA_G	BIOCARTA_G	30	0.52350337	1.59589909	0.02420857	0.06913408	0.0415319	8786	tags=40%, list=24%, signal=30%
WP_THYROID	WP_THYROID	95	0.38835121	1.466201	0.02426343	0.06920758	0.04157606	3867	tags=16%, list=10%, signal=14%
WP_LNCRNA	WP_LNCRNA	96	0.38925883	1.47153673	0.02434783	0.06936503	0.04167065	5091	tags=23%, list=14%, signal=20%
KEGG_FC_EP	KEGG_FC_EP	79	0.39812649	1.44309532	0.02447552	0.06943646	0.04171356	7348	tags=35%, list=20%, signal=28%
PID_REELIN_	PID_REELIN_	28	0.53103808	1.5929427	0.02448211	0.06943646	0.04171356	5879	tags=32%, list=16%, signal=27%
REACTOME_I	REACTOME_I	16	-0.6006349	-1.6170195	0.0244898	0.06943646	0.04171356	10939	tags=81%, list=30%, signal=57%
REACTOME_I	REACTOME_I	16	-0.6012315	-1.6186258	0.0244898	0.06943646	0.04171356	12926	tags=69%, list=35%, signal=45%
REACTOME_I	REACTOME_I	27	0.55079208	1.63791627	0.0247619	0.0701243	0.04212677	5327	tags=33%, list=14%, signal=29%
REACTOME_I	REACTOME_I	16	0.63933771	1.67640056	0.02539063	0.0718192	0.04314497	2921	tags=44%, list=8%, signal=40%
REACTOME_(	REACTOME_(	11	-0.6643375	-1.5877738	0.02544031	0.07187418	0.043178	11295	tags=91%, list=31%, signal=63%
KEGG_HOMC	KEGG_HOMC	28	-0.5044569	-1.5722542	0.02547771	0.07189434	0.04319011	8031	tags=50%, list=22%, signal=39%
WP_AMYOTF	WP_AMYOTF	38	0.49963752	1.5912721	0.02559415	0.07207022	0.04329577	3191	tags=18%, list=9%, signal=17%
ST_WNT_BET	ST_WNT_BET	34	0.50856513	1.59456855	0.02568807	0.07207022	0.04329577	2443	tags=24%, list=7%, signal=22%
BIOCARTA_C	BIOCARTA_C	19	0.60378167	1.6541227	0.0256917	0.07207022	0.04329577	1959	tags=16%, list=5%, signal=15%
PID_TCR_JNK	PID_TCR_JNK	14	0.65929588	1.69853687	0.0256917	0.07207022	0.04329577	7059	tags=43%, list=19%, signal=35%
REACTOME_(	REACTOME_(	14	0.66087604	1.70260782	0.0256917	0.07207022	0.04329577	2243	tags=36%, list=6%, signal=34%
WP_OSTEOP	WP_OSTEOP	13	0.66045255	1.66747341	0.02579365	0.07227089	0.04341632	5585	tags=38%, list=15%, signal=33%
BIOCARTA_M	BIOCARTA_M	18	-0.5693729	-1.6058847	0.026	0.07276254	0.04371168	13231	tags=78%, list=36%, signal=50%
WP_CONSTIT	WP_CONSTIT	32	-0.4954755	-1.5934251	0.02603037	0.07276254	0.04371168	6060	tags=69%, list=16%, signal=57%
REACTOME_I	REACTOME_I	29	0.52008134	1.5733269	0.02616822	0.07306193	0.04389154	8358	tags=52%, list=23%, signal=40%
KEGG_ONE_(	KEGG_ONE_(	17	-0.5734582	-1.5821495	0.02620968	0.07309178	0.04390947	12080	tags=76%, list=33%, signal=51%
REACTOME_I	REACTOME_I	10	0.70548681	1.65232758	0.02642276	0.07359963	0.04421456	7277	tags=60%, list=20%, signal=48%
REACTOME_I	REACTOME_I	55	0.43837474	1.4833732	0.02650177	0.07373325	0.04429483	2999	tags=24%, list=8%, signal=22%
BIOCARTA_N	BIOCARTA_N	23	0.55034834	1.58204787	0.02666667	0.07410526	0.04451831	8170	tags=43%, list=22%, signal=34%
PID_DELTA_N	PID_DELTA_N	47	-0.4288502	-1.4974192	0.02678571	0.07434913	0.04466482	2006	tags=32%, list=5%, signal=30%
WP_OVERVIE	WP_OVERVIE	35	0.50317114	1.58277708	0.02722323	0.07534884	0.04526538	8337	tags=46%, list=23%, signal=35%
REACTOME_(	REACTOME_(	27	-0.51292	-1.5944813	0.02725367	0.07534884	0.04526538	10717	tags=59%, list=29%, signal=42%
REACTOME_I	REACTOME_I	27	-0.5097511	-1.5846303	0.02725367	0.07534884	0.04526538	16600	tags=85%, list=45%, signal=47%
WP_VITAMIN	WP_VITAMIN	51	-0.4137553	-1.4683274	0.02727273	0.07534884	0.04526538	3993	tags=31%, list=11%, signal=28%
KEGG_GLUTA	KEGG_GLUTA	48	-0.4214773	-1.471757	0.02752294	0.07586368	0.04557467	12821	tags=65%, list=35%, signal=42%
WP_DNA_IRD	WP_DNA_IRD	55	-0.4051141	-1.4608719	0.02752294	0.07586368	0.04557467	16959	tags=65%, list=46%, signal=35%
KEGG_CHROI	KEGG_CHROI	73	0.41250056	1.47959915	0.02763385	0.07608115	0.04570532	5571	tags=22%, list=15%, signal=19%
REACTOME_S	REACTOME_S	33	0.51183986	1.59661659	0.02767528	0.07610701	0.04572085	6098	tags=36%, list=17%, signal=30%
BIOCARTA_C	BIOCARTA_C	18	0.62125951	1.67504956	0.02788845	0.07656695	0.04599716	8606	tags=44%, list=23%, signal=34%
REACTOME_T	REACTOME_T	79	-0.3620064	-1.3977125	0.02790698	0.07656695	0.04599716	14520	tags=62%, list=39%, signal=38%
REACTOME_(	REACTOME_(	129	0.35064112	1.39766594	0.02809917	0.07700535	0.04626052	3708	tags=21%, list=10%, signal=19%
REACTOME_I	REACTOME_I	12	0.66533548	1.63088832	0.02816901	0.07710781	0.04632208	8038	tags=58%, list=22%, signal=46%
REACTOME_V	REACTOME_V	100	-0.3445768	-1.3891021	0.02823529	0.0772003	0.04637764	713	tags=18%, list=2%, signal=18%
REACTOME_I	REACTOME_I	48	0.45525128	1.51220305	0.02826855	0.07720239	0.04637889	7348	tags=38%, list=20%, signal=30%
REACTOME_(	REACTOME_(	271	-0.2717658	-1.2639811	0.02857143	0.07785059	0.0467683	17438	tags=51%, list=47%, signal=27%
REACTOME_I	REACTOME_I	23	0.54452761	1.56531543	0.02857143	0.07785059	0.0467683	7348	tags=48%, list=20%, signal=38%
REACTOME_A	REACTOME_A	107	-0.3423676	-1.3931507	0.02877698	0.07826878	0.04701952	11855	tags=49%, list=32%, signal=33%
REACTOME_S	REACTOME_S	25	0.54461854	1.59107961	0.02879079	0.07826878	0.04701952	8038	tags=48%, list=22%, signal=38%
PID_RHOA_R	PID_RHOA_R	46	0.46578584	1.52594104	0.0291439	0.07913817	0.04754181	7366	tags=35%, list=20%, signal=28%
REACTOME_X	REACTOME_X	23	-0.5180252	-1.5547705	0.0293501	0.07960713	0.04782353	10285	tags=57%, list=28%, signal=41%
KEGG_DRUG	KEGG_DRUG	51	-0.4122851	-1.4631101	0.02954545	0.07994297	0.04802528	11150	tags=57%, list=30%, signal=40%
WP_TRANSLA	WP_TRANSLA	51	-0.4131888	-1.4663172	0.02954545	0.07994297	0.04802528	17115	tags=67%, list=46%, signal=36%
WP_DEVELOI	WP_DEVELOI	32	0.50830814	1.56992656	0.02957486	0.07994297	0.04802528	7307	tags=53%, list=20%, signal=43%
BIOCARTA_C	BIOCARTA_C	19	0.58861834	1.61258119	0.02964427	0.07994867	0.04802871	1256	tags=16%, list=3%, signal=15%
PID_IL5_PATI	PID_IL5_PATI	14	0.65295008	1.68218826	0.02964427	0.07994867	0.04802871	9860	tags=64%, list=27%, signal=47%
REACTOME_I	REACTOME_I	66	0.42028851	1.47403002	0.02972028	0.07998211	0.0480488	3158	tags=17%, list=9%, signal=15%
REACTOME_I	REACTOME_I	28	-0.4959832	-1.5458441	0.02972399	0.07998211	0.0480488	14967	tags=71%, list=41%, signal=42%
KEGG_PROST	KEGG_PROST	89	0.38399592	1.43228765	0.02977233	0.08002155	0.04807249	5571	tags=21%, list=15%, signal=18%
REACTOME_I	REACTOME_I	55	-0.401231	-1.4468693	0.02981651	0.08004976	0.04808944	18036	tags=80%, list=49%, signal=41%
WP_AMINO_	WP_AMINO_	91	-0.3458707	-1.3730802	0.02995392	0.08027027	0.04822191	12880	tags=65%, list=35%, signal=42%
PID_BARD1_I	PID_BARD1_I	29	-0.4979444	-1.5715773	0.02997859	0.08027027	0.04822191	16527	tags=86%, list=45%, signal=48%

WP_TCA_CY	WP_TCA_CY	18	-0.5484974	-1.5470064	0.03	0.08027027	0.04822191	12316	tags=72%, list=33%, signal=48%
WP_IL10_AN	WP_IL10_AN	12	0.66343087	1.62621969	0.03018109	0.08057386	0.04840429	8495	tags=67%, list=23%, signal=51%
BIOCARTA_P	BIOCARTA_P	19	-0.5354679	-1.5365687	0.03024194	0.08057386	0.04840429	16229	tags=95%, list=44%, signal=53%
REACTOME_	REACTOME_	19	-0.5407707	-1.5517855	0.03024194	0.08057386	0.04840429	10717	tags=58%, list=29%, signal=41%
REACTOME_	REACTOME_	51	0.45574153	1.51916995	0.03024911	0.08057386	0.04840429	6424	tags=31%, list=17%, signal=26%
REACTOME_	REACTOME_	11	0.68426098	1.64139094	0.0305499	0.08119302	0.04877624	5418	tags=45%, list=15%, signal=39%
REACTOME_	REACTOME_	11	0.68579171	1.64506284	0.0305499	0.08119302	0.04877624	10746	tags=82%, list=29%, signal=58%
REACTOME_	REACTOME_	20	-0.5269285	-1.5342015	0.03061224	0.08125542	0.04881373	13242	tags=70%, list=36%, signal=45%
WP_MIRNA_	WP_MIRNA_	47	0.45308766	1.48842291	0.03068592	0.08125542	0.04881373	7513	tags=28%, list=20%, signal=22%
BIOCARTA_H	BIOCARTA_H	25	0.54209475	1.58370646	0.03071017	0.08125542	0.04881373	8606	tags=56%, list=23%, signal=43%
REACTOME_	REACTOME_	25	0.54323825	1.58704715	0.03071017	0.08125542	0.04881373	3449	tags=24%, list=9%, signal=22%
REACTOME_	REACTOME_	47	-0.4247758	-1.4831927	0.03125	0.08259177	0.04961653	18611	tags=74%, list=50%, signal=37%
WP_VITAMIN	WP_VITAMIN	183	-0.2937436	-1.3026185	0.03133159	0.0827154	0.04969081	4708	tags=22%, list=13%, signal=20%
PID_SMAD2_	PID_SMAD2_	82	0.39354237	1.44039807	0.03146853	0.08298472	0.04985259	8305	tags=33%, list=23%, signal=26%
PID_ATF2_PA	PID_ATF2_PA	59	0.42970714	1.47851742	0.03152364	0.08303789	0.04988454	6019	tags=29%, list=16%, signal=24%
WP_TGFB_SI	WP_TGFB_SI	18	0.61151014	1.64876315	0.03187251	0.08386388	0.05038075	4961	tags=39%, list=13%, signal=34%
REACTOME_	REACTOME_	29	-0.4952253	-1.5629954	0.03211991	0.08442137	0.05071565	14145	tags=79%, list=38%, signal=49%
WP_BONE_M	WP_BONE_M	12	0.65833882	1.61373792	0.03219316	0.08452038	0.05077514	6739	tags=42%, list=18%, signal=34%
REACTOME_	REACTOME_	10	0.6886205	1.61282483	0.03252033	0.08519106	0.05117804	7273	tags=80%, list=20%, signal=64%
REACTOME_	REACTOME_	10	0.68513196	1.6046543	0.03252033	0.08519106	0.05117804	960	tags=20%, list=3%, signal=19%
WP_METABO	WP_METABO	16	-0.5835885	-1.5711274	0.03265306	0.0853893	0.05129713	2088	tags=38%, list=6%, signal=35%
REACTOME_	REACTOME_	35	0.4927026	1.5498472	0.03266788	0.0853893	0.05129713	4009	tags=34%, list=11%, signal=31%
REACTOME_	REACTOME_	24	-0.516421	-1.5776272	0.03278689	0.0856062	0.05142743	12507	tags=92%, list=34%, signal=61%
WP_DNA_MI	WP_DNA_MI	23	-0.5146514	-1.5446448	0.03354298	0.08743273	0.05252472	12507	tags=61%, list=34%, signal=40%
PID_TGFBR_	PID_TGFBR_	54	0.43689877	1.47670501	0.0335689	0.08743273	0.05252472	4850	tags=19%, list=13%, signal=16%
BIOCARTA_T	BIOCARTA_T	19	0.58330318	1.59801975	0.03359684	0.08743273	0.05252472	7401	tags=37%, list=20%, signal=29%
REACTOME_	REACTOME_	12	-0.6400296	-1.5860095	0.03366337	0.08751002	0.05257114	12410	tags=92%, list=34%, signal=61%
WP_SULFATI	WP_SULFATI	18	-0.5384434	-1.5186497	0.034	0.08828852	0.05303883	3997	tags=50%, list=11%, signal=45%
REACTOME_	REACTOME_	27	0.52039561	1.54752488	0.03428571	0.08893325	0.05342614	6618	tags=33%, list=18%, signal=27%
REACTOME_	REACTOME_	10	0.67809852	1.5881812	0.03455285	0.08908608	0.05351795	4072	tags=50%, list=11%, signal=44%
REACTOME_	REACTOME_	11	0.67136672	1.61046046	0.03462322	0.08908608	0.05351795	51	tags=9%, list=0%, signal=9%
BIOCARTA_IG	BIOCARTA_IG	21	0.56485563	1.58834038	0.03468208	0.08908608	0.05351795	9009	tags=48%, list=24%, signal=36%
BIOCARTA_IL	BIOCARTA_IL	21	0.56474093	1.58801785	0.03468208	0.08908608	0.05351795	10122	tags=62%, list=27%, signal=45%
BIOCARTA_IN	BIOCARTA_IN	21	0.55608365	1.5636741	0.03468208	0.08908608	0.05351795	9009	tags=48%, list=24%, signal=36%
BIOCARTA_N	BIOCARTA_N	21	0.56576716	1.59090353	0.03468208	0.08908608	0.05351795	2445	tags=24%, list=7%, signal=22%
REACTOME_	REACTOME_	22	0.55621651	1.58384001	0.03468208	0.08908608	0.05351795	7017	tags=50%, list=19%, signal=41%
REACTOME_	REACTOME_	21	0.55703194	1.56634063	0.03468208	0.08908608	0.05351795	2921	tags=29%, list=8%, signal=26%
REACTOME_	REACTOME_	21	0.56601811	1.59160919	0.03468208	0.08908608	0.05351795	2921	tags=29%, list=8%, signal=26%
REACTOME_	REACTOME_	97	0.36911056	1.39755521	0.03484321	0.08935602	0.05368012	7004	tags=22%, list=19%, signal=18%
PID_HDAC_C	PID_HDAC_C	34	0.5010216	1.57091635	0.03486239	0.08935602	0.05368012	6175	tags=35%, list=17%, signal=29%
REACTOME_	REACTOME_	37	0.48787743	1.54027662	0.03492647	0.08937296	0.05369029	3027	tags=22%, list=8%, signal=20%
PID_INSULIN_	PID_INSULIN_	44	0.45927059	1.48625743	0.03499079	0.08937296	0.05369029	9323	tags=32%, list=25%, signal=24%
REACTOME_	REACTOME_	24	0.55046205	1.58629543	0.03501946	0.08937296	0.05369029	3802	tags=21%, list=10%, signal=19%
WP_IL1_AND	WP_IL1_AND	24	0.54280618	1.56423312	0.03501946	0.08937296	0.05369029	5992	tags=54%, list=16%, signal=45%
WP_MIRNA_	WP_MIRNA_	84	-0.3495892	-1.3586749	0.03562945	0.09083217	0.05456691	11668	tags=61%, list=32%, signal=42%
KEGG_GLYCO	KEGG_GLYCO	15	0.63412646	1.64027036	0.036	0.09167846	0.05507531	7387	tags=53%, list=20%, signal=43%
REACTOME_	REACTOME_	102	0.36083943	1.37309213	0.03626943	0.0922657	0.05542809	5332	tags=19%, list=14%, signal=16%
WP_NEURAL_	WP_NEURAL_	100	0.36038119	1.36814115	0.03639515	0.09240652	0.05551269	5648	tags=31%, list=15%, signal=26%
KEGG_LINOL	KEGG_LINOL	29	-0.4836771	-1.5265479	0.03640257	0.09240652	0.05551269	5905	tags=48%, list=16%, signal=41%
WP_AGERAG	WP_AGERAG	66	0.41219548	1.44564627	0.03671329	0.0930958	0.05592677	5088	tags=21%, list=14%, signal=18%
REACTOME_	REACTOME_	32	-0.4779139	-1.5369477	0.03687636	0.09340962	0.05611529	16600	tags=88%, list=45%, signal=48%
BIOCARTA_TI	BIOCARTA_TI	24	0.53271252	1.53514566	0.03696498	0.09344398	0.05613594	10122	tags=54%, list=27%, signal=39%
PID_ALPHA_	PID_ALPHA_	32	0.49545379	1.53022545	0.03696858	0.09344398	0.05613594	7037	tags=34%, list=19%, signal=28%
REACTOME_	REACTOME_	53	-0.4068282	-1.455815	0.03729604	0.0941715	0.05657299	18883	tags=74%, list=51%, signal=36%
KEGG_CARDI	KEGG_CARDI	74	0.3986469	1.43403048	0.03793103	0.09567318	0.05747512	4294	tags=24%, list=12%, signal=22%
REACTOME_	REACTOME_	13	-0.6157768	-1.5587589	0.03815261	0.09613001	0.05774956	9834	tags=62%, list=27%, signal=45%
WP_SYNAPTI	WP_SYNAPTI	50	0.43608621	1.46159139	0.0385289	0.09688073	0.05820055	5991	tags=24%, list=16%, signal=20%
REACTOME_	REACTOME_	34	0.49340472	1.54703421	0.03853211	0.09688073	0.05820055	3449	tags=18%, list=9%, signal=16%
REACTOME_	REACTOME_	10	0.67030794	1.56993482	0.03861789	0.09699376	0.05826845	5774	tags=50%, list=16%, signal=42%
REACTOME_	REACTOME_	11	0.66639073	1.59852416	0.03869654	0.09700637	0.05827602	8495	tags=64%, list=23%, signal=49%
PID_CD40_PA	PID_CD40_PA	31	0.50248905	1.54582111	0.03874539	0.09700637	0.05827602	6751	tags=35%, list=18%, signal=29%
WP_MAPK_C	WP_MAPK_C	33	0.48846872	1.52371344	0.03874539	0.09700637	0.05827602	4951	tags=21%, list=13%, signal=18%
WP_NRF2_PA	WP_NRF2_PA	145	-0.3098348	-1.321924	0.03902439	0.09749212	0.05856784	5477	tags=26%, list=15%, signal=22%
REACTOME_	REACTOME_	32	-0.4594932	-1.4777077	0.03904555	0.09749212	0.05856784	13576	tags=53%, list=37%, signal=34%
BIOCARTA_N	BIOCARTA_N	20	0.55889726	1.55023674	0.0390625	0.09749212	0.05856784	7348	tags=45%, list=20%, signal=36%
REACTOME_	REACTOME_	328	0.28864079	1.27802378	0.03933434	0.09806757	0.05891353	5101	tags=15%, list=14%, signal=13%
WP_LTF_DAN	WP_LTF_DAN	19	0.5703284	1.562474	0.03952569	0.09838757	0.05910577	6575	tags=37%, list=18%, signal=30%
PID_INTEGRI	PID_INTEGRI	28	0.50597511	1.5177619	0.03954802	0.09838757	0.05910577	5091	tags=71%, list=14%, signal=62%
PID_CMYB_P	PID_CMYB_P	84	0.38259722	1.41405646	0.03958692	0.09838757	0.05910577	6609	tags=29%, list=18%, signal=24%
WP_ANGIOP	WP_ANGIOP	132	0.34177931	1.36848635	0.0397351	0.09865266	0.05926502	5879	tags=19%, list=16%, signal=16%
REACTOME_	REACTOME_	27	-0.4920624	-1.5296425	0.03983229	0.09879072	0.05934796	92	tags=11%, list=0%, signal=11%
KEGG_APOPT	KEGG_APOPT	87	0.37647568	1.39884779	0.04	0.09910323	0.0595357	9508	tags=34%, list=26%, signal=26%
BIOCARTA_SI	BIOCARTA_SI	14	-0.5949075	-1.538957	0.04032258	0.09969454	0.05989092	8205	tags=86%, list=22%, signal=67%
REACTOME_	REACTOME_	14	-0.5958825	-1.541479	0.04032258	0.09969454	0.05989092	7939	tags=64%, list=22%, signal=50%

WP_GLOBO_	WP_GLOBO_	21	0.5379618	1.51271653	0.04046243	0.09993631	0.06003617	4849	tags=33%, list=13%, signal=29%
PID_UPA_UP	PID_UPA_UP	42	0.46094041	1.47372185	0.04089219	0.10089289	0.06061083	5624	tags=57%, list=15%, signal=48%
REACTOME_(	REACTOME_(	24	-0.5082484	-1.5526605	0.04098361	0.10095483	0.06064804	12878	tags=67%, list=35%, signal=43%
REACTOME_I	REACTOME_I	58	-0.3897378	-1.4140163	0.04100228	0.10095483	0.06064804	17647	tags=66%, list=48%, signal=34%
PID_P75_NTI	PID_P75_NTI	68	0.40645677	1.43391485	0.04137931	0.10177768	0.06114236	5877	tags=19%, list=16%, signal=16%
PID_LIS1_PA	PID_LIS1_PA	28	0.50340152	1.51004196	0.04143126	0.10180008	0.06115582	5624	tags=25%, list=15%, signal=21%
REACTOME_S	REACTOME_S	39	-0.4368223	-1.4665594	0.04166667	0.10227273	0.06143976	17862	tags=77%, list=48%, signal=40%
REACTOME_V	REACTOME_V	15	0.62486284	1.61630852	0.042	0.10298452	0.06186736	4079	tags=27%, list=11%, signal=24%
REACTOME_S	REACTOME_S	43	0.45384198	1.46406791	0.04204753	0.10299478	0.06187353	7348	tags=40%, list=20%, signal=32%
KEGG_COLOI	KEGG_COLOI	62	0.41788884	1.45226827	0.04210526	0.10302997	0.06189467	4850	tags=21%, list=13%, signal=18%
REACTOME_I	REACTOME_I	61	0.41387649	1.43390066	0.04217926	0.10310486	0.06193966	6175	tags=31%, list=17%, signal=26%
PID_FRA_PA	PID_FRA_PA	37	0.47609082	1.50306515	0.04227941	0.1031934	0.06199285	6019	tags=41%, list=16%, signal=34%
BIOCARTA_A	BIOCARTA_A	22	0.53885382	1.53439933	0.04238921	0.1031934	0.06199285	8190	tags=41%, list=22%, signal=32%
BIOCARTA_IL	BIOCARTA_IL	22	0.53036808	1.51023597	0.04238921	0.1031934	0.06199285	9009	tags=55%, list=24%, signal=41%
WP_TYPE_II_	WP_TYPE_II_	22	0.53946878	1.53615043	0.04238921	0.1031934	0.06199285	1716	tags=14%, list=5%, signal=13%
WP_ALZHEIN	WP_ALZHEIN	133	0.33723047	1.3517897	0.04297521	0.10451289	0.06278552	5649	tags=23%, list=15%, signal=19%
REACTOME_I	REACTOME_I	22	-0.5124347	-1.5279795	0.04347826	0.10552027	0.0633907	13242	tags=77%, list=36%, signal=50%
REACTOME_I	REACTOME_I	14	0.6236084	1.6065956	0.04347826	0.10552027	0.0633907	5759	tags=57%, list=16%, signal=48%
PID_GMCSF_	PID_GMCSF_	36	0.47825466	1.50822127	0.04355717	0.10560391	0.06344095	9009	tags=44%, list=24%, signal=34%
REACTOME_(	REACTOME_(	15	-0.5931736	-1.5766041	0.0438247	0.10614423	0.06376554	92	tags=20%, list=0%, signal=20%
WP_PROTEAS	WP_PROTEAS	62	-0.3834752	-1.4104776	0.04398148	0.10635198	0.06389035	15617	tags=73%, list=42%, signal=42%
BIOCARTA_TA	BIOCARTA_TA	15	0.61465469	1.5899035	0.044	0.10635198	0.06389035	8299	tags=47%, list=23%, signal=36%
WP_SIGNAL_	WP_SIGNAL_	33	0.48268575	1.50567424	0.04428044	0.10692107	0.06423222	6751	tags=27%, list=18%, signal=22%
REACTOME_I	REACTOME_I	51	0.43066337	1.43557436	0.04448399	0.1073035	0.06446197	3350	tags=16%, list=9%, signal=14%
WP_CANNAB	WP_CANNAB	29	0.50140312	1.51682237	0.04485981	0.10810032	0.06494065	5185	tags=31%, list=14%, signal=27%
REACTOME_I	REACTOME_I	20	0.54677319	1.51660771	0.04492188	0.1081402	0.06496461	8936	tags=60%, list=24%, signal=45%
REACTOME_(	REACTOME_(	11	-0.6374567	-1.5235283	0.04500978	0.10824215	0.06502586	10285	tags=73%, list=28%, signal=52%
BIOCARTA_G	BIOCARTA_G	24	-0.495165	-1.5126916	0.04508197	0.10830612	0.06506428	7790	tags=42%, list=21%, signal=33%
REACTOME_T	REACTOME_T	17	0.60010339	1.59248318	0.04545455	0.10909091	0.06553574	6282	tags=41%, list=17%, signal=34%
WP_MFAP5N	WP_MFAP5N	13	0.63579117	1.60520976	0.04563492	0.10934432	0.06568798	1261	tags=23%, list=3%, signal=22%
REACTOME_I	REACTOME_I	33	-0.455941	-1.4746975	0.04565217	0.10934432	0.06568798	1975	tags=18%, list=5%, signal=17%
BIOCARTA_P	BIOCARTA_P	18	0.58064922	1.56555547	0.04581673	0.1094076	0.06572599	9009	tags=39%, list=24%, signal=29%
REACTOME_I	REACTOME_I	15	-0.5888429	-1.5650935	0.04581673	0.1094076	0.06572599	92	tags=20%, list=0%, signal=20%
WP_CODEINI	WP_CODEINI	15	-0.5890447	-1.56563	0.04581673	0.1094076	0.06572599	7208	tags=73%, list=20%, signal=59%
REACTOME_(	REACTOME_(	34	-0.4486259	-1.459918	0.04595186	0.10948461	0.06577225	10694	tags=62%, list=29%, signal=44%
ST_JNK_MAP	ST_JNK_MAP	40	0.45944195	1.47109889	0.04595588	0.10948461	0.06577225	9314	tags=35%, list=25%, signal=26%
REACTOME_I	REACTOME_I	25	0.51465344	1.50353786	0.04606526	0.10948461	0.06577225	8936	tags=56%, list=24%, signal=42%
WP_MAMMA	WP_MAMMA	25	0.5157474	1.50673381	0.04606526	0.10948461	0.06577225	5143	tags=36%, list=14%, signal=31%
BIOCARTA_IL	BIOCARTA_IL	31	0.49419751	1.52031361	0.04612546	0.10948461	0.06577225	6751	tags=29%, list=18%, signal=24%
PID_EPO_PA	PID_EPO_PA	33	0.48200345	1.50354587	0.04612546	0.10948461	0.06577225	6180	tags=33%, list=17%, signal=28%
REACTOME_(	REACTOME_(	21	0.52649905	1.48048395	0.04624277	0.10965353	0.06587373	7925	tags=43%, list=21%, signal=34%
KEGG_PENT(	KEGG_PENT(	28	-0.4737374	-1.4765097	0.04670913	0.11053874	0.06640551	4702	tags=68%, list=13%, signal=59%
REACTOME_I	REACTOME_I	28	-0.4762628	-1.4843807	0.04670913	0.11053874	0.06640551	16407	tags=68%, list=45%, signal=38%
KEGG_PANCI	KEGG_PANCI	70	0.40085777	1.42172345	0.046875	0.11071074	0.06650884	4850	tags=21%, list=13%, signal=19%
REACTOME_I	REACTOME_I	20	0.54177958	1.50275672	0.046875	0.11071074	0.06650884	4072	tags=35%, list=11%, signal=31%
REACTOME_I	REACTOME_I	26	-0.4817751	-1.4838933	0.04700855	0.1109159	0.06663209	1973	tags=23%, list=5%, signal=22%
REACTOME_T	REACTOME_T	13	0.63407329	1.60087255	0.04761905	0.11213365	0.06736365	5808	tags=46%, list=16%, signal=39%
WP_GANGLI(	WP_GANGLI(	13	0.63345157	1.59930286	0.04761905	0.11213365	0.06736365	4630	tags=46%, list=13%, signal=40%
REACTOME_I	REACTOME_I	51	-0.3998751	-1.4190698	0.04772727	0.11227723	0.0674499	17600	tags=76%, list=48%, signal=40%
REACTOME_I	REACTOME_I	231	-0.2700955	-1.2301479	0.04788732	0.11254232	0.06760915	17327	tags=66%, list=47%, signal=35%
PID_SYNDEC	PID_SYNDEC	33	0.47870938	1.49327045	0.04797048	0.11262634	0.06765963	4976	tags=24%, list=13%, signal=21%
KEGG_BUTAN	KEGG_BUTAN	34	-0.4445534	-1.4466652	0.04814004	0.11291288	0.06783177	10537	tags=59%, list=29%, signal=42%
REACTOME_I	REACTOME_I	30	0.49271111	1.50202895	0.04841713	0.1134508	0.06815492	8195	tags=43%, list=22%, signal=34%
REACTOME_I	REACTOME_I	56	-0.3847117	-1.3897864	0.04849885	0.1135303	0.06820268	19348	tags=75%, list=52%, signal=36%
REACTOME_T	REACTOME_T	62	-0.3767778	-1.3858436	0.04861111	0.1136811	0.06829327	17243	tags=63%, list=47%, signal=34%
KEGG_LYSOS	KEGG_LYSOS	121	0.33503521	1.3208424	0.04882155	0.11406096	0.06852147	9786	tags=35%, list=27%, signal=26%
REACTOME_I	REACTOME_I	16	-0.5642681	-1.5191133	0.04897959	0.11431779	0.06867576	9782	tags=62%, list=27%, signal=46%
WP_BIOMAR	WP_BIOMAR	12	-0.6085424	-1.5079836	0.04950495	0.11543058	0.06934426	5849	tags=58%, list=16%, signal=49%
REACTOME_I	REACTOME_I	18	0.57365922	1.54670893	0.0498008	0.11600656	0.06969028	3449	tags=33%, list=9%, signal=30%
REACTOME_T	REACTOME_T	31	-0.4503791	-1.4394408	0.05	0.11635651	0.06990051	3483	tags=19%, list=9%, signal=18%
KEGG_PENT(	KEGG_PENT(	27	-0.4685732	-1.4566231	0.05031447	0.11697375	0.07027131	16250	tags=78%, list=44%, signal=44%
REACTOME_I	REACTOME_I	14	-0.5837962	-1.5102131	0.05040323	0.11706556	0.07032646	13242	tags=79%, list=36%, signal=50%
KEGG_BASE_	KEGG_BASE_	35	-0.4469152	-1.4665275	0.05099778	0.11826779	0.0710487	12507	tags=71%, list=34%, signal=47%
WP_TCA_CY(	WP_TCA_CY(	16	-0.5617968	-1.5124602	0.05102041	0.11826779	0.0710487	14013	tags=81%, list=38%, signal=50%
WP_INTERFE	WP_INTERFE	54	0.4235602	1.43162102	0.05123675	0.11852739	0.07120465	8495	tags=35%, list=23%, signal=27%
REACTOME_A	REACTOME_A	26	-0.473035	-1.4569735	0.05128205	0.11852739	0.07120465	16950	tags=77%, list=46%, signal=42%
REACTOME_I	REACTOME_I	26	-0.4709692	-1.4506107	0.05128205	0.11852739	0.07120465	15867	tags=73%, list=43%, signal=42%
BIOCARTA_U	BIOCARTA_U	14	0.61043946	1.5726686	0.0513834	0.11864622	0.07127604	4976	tags=50%, list=13%, signal=43%
KEGG_ALZHE	KEGG_ALZHE	159	-0.2859749	-1.2401537	0.05159705	0.11902388	0.07150291	12700	tags=53%, list=34%, signal=35%
KEGG_ENDO(	KEGG_ENDO(	181	0.31214931	1.29961171	0.05186386	0.1195233	0.07180294	8400	tags=25%, list=23%, signal=20%
WP_NUCLEO	WP_NUCLEO	19	-0.5180318	-1.4865345	0.05241935	0.12060433	0.07245236	14042	tags=68%, list=38%, signal=42%
KEGG_GLYC(	KEGG_GLYC(	26	0.50773674	1.50366064	0.05243446	0.12060433	0.07245236	7987	tags=38%, list=22%, signal=30%
REACTOME_I	REACTOME_I	43	-0.4171102	-1.4285009	0.05274725	0.12120645	0.07281408	17243	tags=67%, list=47%, signal=36%
WP_THE_INF	WP_THE_INF	37	0.46052212	1.45391325	0.05330882	0.12226953	0.07345272	7283	tags=24%, list=20%, signal=20%

BIOCARTA_G	BIOCARTA_G	17	0.57267111	1.51968665	0.05335968	0.12226953	0.07345272	7348	tags=41%, list=20%, signal=33%
REACTOME_I	REACTOME_I	90	-0.3344028	-1.3218346	0.05336427	0.12226953	0.07345272	13131	tags=56%, list=36%, signal=36%
REACTOME_I	REACTOME_I	79	-0.3451943	-1.3328006	0.05348837	0.12243581	0.07355261	14195	tags=61%, list=39%, signal=37%
REACTOME_/	REACTOME_/	52	0.42209424	1.41306594	0.05357143	0.12250791	0.07359592	4449	tags=31%, list=12%, signal=27%
KEGG_TERPE	KEGG_TERPE	15	-0.5768283	-1.5331597	0.05378486	0.12287772	0.07381809	13104	tags=80%, list=36%, signal=52%
REACTOME_(	REACTOME_(	22	0.5179517	1.47488004	0.0539499	0.12313638	0.07397348	5996	tags=36%, list=16%, signal=30%
REACTOME_I	REACTOME_I	12	0.6304015	1.54525722	0.05432596	0.12387569	0.07441761	2376	tags=42%, list=6%, signal=39%
KEGG_NUCLE	KEGG_NUCLE	44	-0.41767	-1.4401123	0.05446623	0.12397692	0.07447843	18420	tags=73%, list=50%, signal=36%
REACTOME_(	REACTOME_(	24	0.51211883	1.47579974	0.05447471	0.12397692	0.07447843	4932	tags=33%, list=13%, signal=29%
REACTOME_I	REACTOME_I	37	-0.4324374	-1.4368589	0.05458515	0.1241094	0.07455801	17600	tags=78%, list=48%, signal=41%
REACTOME_I	REACTOME_I	55	-0.3881284	-1.3996205	0.05504587	0.12503728	0.07511543	12316	tags=56%, list=33%, signal=38%
BIOCARTA_E(	BIOCARTA_E(	19	0.54460613	1.49200517	0.05533597	0.12557618	0.07543917	9019	tags=47%, list=24%, signal=36%
REACTOME_I	REACTOME_I	61	-0.3674577	-1.3458121	0.05542725	0.12566331	0.07549152	11720	tags=54%, list=32%, signal=37%
REACTOME_I	REACTOME_I	140	-0.3002872	-1.2653306	0.05583756	0.12647288	0.07597786	12399	tags=45%, list=34%, signal=30%
PID_EPHRINE	PID_EPHRINE	30	-0.4539295	-1.443005	0.05591398	0.12652535	0.07600938	92	tags=10%, list=0%, signal=10%
REACTOME_T	REACTOME_T	18	-0.5210964	-1.4697235	0.056	0.12659943	0.07605388	5251	tags=39%, list=14%, signal=33%
REACTOME_I	REACTOME_I	13	-0.5976622	-1.5129042	0.0562249	0.12698704	0.07628674	1796	tags=46%, list=5%, signal=44%
PID_TNF_PAT	PID_TNF_PAT	46	0.42564659	1.39444254	0.0564663	0.12741114	0.07654152	6575	tags=20%, list=18%, signal=16%
WP_IL5_SIGN	WP_IL5_SIGN	40	0.452169	1.44781144	0.05698529	0.12843119	0.07715431	5571	tags=25%, list=15%, signal=21%
PID_INTEGRI	PID_INTEGRI	11	0.63225103	1.51663056	0.05702648	0.12843119	0.07715431	4260	tags=36%, list=12%, signal=32%
REACTOME_/	REACTOME_/	44	0.43354848	1.40301745	0.05709024	0.12844979	0.07716548	2442	tags=20%, list=7%, signal=19%
KEGG_PURIN	KEGG_PURIN	159	0.3171221	1.29402226	0.05714286	0.12844979	0.07716548	4110	tags=16%, list=11%, signal=15%
BIOCARTA_CI	BIOCARTA_CI	13	0.60680164	1.53201861	0.05753968	0.12897574	0.07748144	4170	tags=23%, list=11%, signal=20%
REACTOME_/	REACTOME_/	13	0.61970046	1.56458482	0.05753968	0.12897574	0.07748144	2226	tags=23%, list=6%, signal=22%
REACTOME_V	REACTOME_V	13	0.61315376	1.54805609	0.05753968	0.12897574	0.07748144	4079	tags=31%, list=11%, signal=27%
REACTOME_I	REACTOME_I	18	0.56118887	1.51308616	0.05776892	0.12932301	0.07769006	4750	tags=33%, list=13%, signal=29%
WP_PATHOG	WP_PATHOG	21	0.51964391	1.46120771	0.05780347	0.12932301	0.07769006	6906	tags=67%, list=19%, signal=54%
WP_NOTCH_	WP_NOTCH_	61	0.40248689	1.39444072	0.05799649	0.12963278	0.07787615	6008	tags=25%, list=16%, signal=21%
REACTOME_(	REACTOME_(	26	0.49760553	1.4736571	0.05805243	0.12963589	0.07787802	6475	tags=35%, list=18%, signal=29%
WP_NUCLEAI	WP_NUCLEAI	38	0.45836084	1.45981192	0.05850091	0.13051472	0.07840597	4610	tags=26%, list=13%, signal=23%
SIG_PIP3_SIG	SIG_PIP3_SIG	67	0.3975084	1.39987646	0.05872193	0.13088491	0.07862836	5407	tags=19%, list=15%, signal=17%
WP_LIVER_X_	WP_LIVER_X_	10	-0.6441603	-1.4833261	0.05882353	0.13098848	0.07869058	8197	tags=80%, list=22%, signal=62%
KEGG_PORPH	KEGG_PORPH	41	-0.4160231	-1.4119671	0.05934066	0.1318928	0.07923385	7208	tags=32%, list=20%, signal=26%
REACTOME_T	REACTOME_T	43	-0.404051	-1.3837762	0.05934066	0.1318928	0.07923385	18008	tags=84%, list=49%, signal=43%
REACTOME_I	REACTOME_I	18	0.55751915	1.5031918	0.05976096	0.13270283	0.07972047	4838	tags=50%, list=13%, signal=43%
REACTOME_(	REACTOME_(	24	0.50862651	1.46573575	0.06031128	0.13379982	0.08037948	1197	tags=17%, list=3%, signal=16%
WP_NUCLEO	WP_NUCLEO	44	-0.4134059	-1.4254095	0.06100218	0.13520632	0.08122442	18420	tags=70%, list=50%, signal=35%
REACTOME_I	REACTOME_I	199	-0.2770499	-1.2432613	0.06117021	0.1354524	0.08137226	14708	tags=51%, list=40%, signal=31%
KEGG_PROTE	KEGG_PROTE	24	-0.4771463	-1.457646	0.06147541	0.13600147	0.0817021	15700	tags=71%, list=43%, signal=41%
REACTOME_I	REACTOME_I	93	0.35240167	1.32112603	0.06183746	0.13667516	0.08210682	5241	tags=27%, list=14%, signal=23%
REACTOME_I	REACTOME_I	141	0.32423639	1.30404446	0.06208054	0.1370849	0.08235297	6577	tags=20%, list=18%, signal=16%
WP_G13_SIG	WP_G13_SIG	39	0.45373771	1.44476055	0.06227106	0.13737794	0.08252901	4487	tags=21%, list=12%, signal=18%
WP_VITAMIN	WP_VITAMIN	40	0.44700481	1.43127609	0.0625	0.1377551	0.08275559	5176	tags=30%, list=14%, signal=26%
BIOCARTA_LI	BIOCARTA_LI	11	0.62019078	1.4877007	0.06313646	0.13902893	0.08352084	3577	tags=27%, list=10%, signal=25%
WP_OSTEOB	WP_OSTEOB	14	0.59444157	1.53145341	0.06324111	0.13913043	0.08358181	4561	tags=57%, list=12%, signal=50%
PID_FGF_PAT	PID_FGF_PAT	55	0.41842928	1.41588171	0.06360424	0.13979988	0.08398398	7348	tags=35%, list=20%, signal=28%
REACTOME_T	REACTOME_T	95	0.35148126	1.32700031	0.06412478	0.14067979	0.08451258	5808	tags=17%, list=16%, signal=14%
REACTOME_S	REACTOME_S	71	-0.3521197	-1.3209397	0.06413302	0.14067979	0.08451258	16779	tags=68%, list=46%, signal=37%
WP_GLYCER(	WP_GLYCER(	22	-0.4903029	-1.4619866	0.06418219	0.14067979	0.08451258	13624	tags=59%, list=37%, signal=37%
BIOCARTA_CI	BIOCARTA_CI	12	0.61854555	1.5161956	0.06438632	0.14099713	0.08470322	1813	tags=25%, list=5%, signal=24%
REACTOME_S	REACTOME_S	33	0.46877985	1.46229662	0.06457565	0.14128152	0.08487407	6385	tags=39%, list=17%, signal=33%
KEGG_INSULI	KEGG_INSULI	137	0.32436224	1.30436343	0.06467662	0.14137226	0.08492857	5185	tags=18%, list=14%, signal=15%
REACTOME_I	REACTOME_I	23	0.50677353	1.45678642	0.0647619	0.14142857	0.08496241	6424	tags=35%, list=17%, signal=29%
REACTOME_(	REACTOME_(	76	0.37964436	1.36306856	0.06502636	0.1418757	0.08523102	6910	tags=26%, list=19%, signal=21%
PID_P73PATH	PID_P73PATH	79	-0.3400411	-1.3129041	0.06511628	0.14190158	0.08524656	7168	tags=25%, list=19%, signal=20%
BIOCARTA_TI	BIOCARTA_TI	14	0.58948013	1.51867131	0.06521739	0.14190158	0.08524656	1433	tags=21%, list=4%, signal=21%
WP_CANCER_	WP_CANCER_	14	0.5904886	1.52126942	0.06521739	0.14190158	0.08524656	8757	tags=64%, list=24%, signal=49%
REACTOME_I	REACTOME_I	189	-0.2755169	-1.2300093	0.06544503	0.14220423	0.08542838	12121	tags=45%, list=33%, signal=30%
WP_ESTROGI	WP_ESTROGI	13	0.60037367	1.51578965	0.06547619	0.14220423	0.08542838	3090	tags=31%, list=8%, signal=28%
WP_CHROM(	WP_CHROM(	73	0.38067267	1.36543563	0.0656304	0.14240897	0.08555138	4850	tags=16%, list=13%, signal=14%
PID_ATR_PAT	PID_ATR_PAT	38	-0.429211	-1.4273371	0.06593407	0.14280706	0.08579052	11867	tags=79%, list=32%, signal=54%
REACTOME_I	REACTOME_I	41	-0.4116553	-1.3971429	0.06593407	0.14280706	0.08579052	18420	tags=73%, list=50%, signal=37%
BIOCARTA_FI	BIOCARTA_FI	34	0.45686273	1.43245948	0.06605505	0.14293879	0.08586966	8786	tags=35%, list=24%, signal=27%
PID_IL23_PAT	PID_IL23_PAT	37	0.45217854	1.42757173	0.06617647	0.14299652	0.08590434	6575	tags=46%, list=18%, signal=38%
REACTOME_I	REACTOME_I	97	0.34640237	1.31157566	0.06620209	0.14299652	0.08590434	7390	tags=25%, list=20%, signal=20%
REACTOME_T	REACTOME_T	16	0.57614659	1.51070781	0.06640625	0.14330722	0.08609099	6355	tags=31%, list=17%, signal=26%
REACTOME_I	REACTOME_I	48	-0.3958223	-1.3821723	0.06651376	0.14332731	0.08610306	12558	tags=50%, list=34%, signal=33%
REACTOME_/	REACTOME_/	11	-0.6150751	-1.4700362	0.0665362	0.14332731	0.08610306	2968	tags=45%, list=8%, signal=42%
KEGG_MISM/	KEGG_MISM/	23	-0.4835051	-1.4511639	0.06708595	0.14438064	0.08673584	11867	tags=83%, list=32%, signal=56%
PID_MYC_AC	PID_MYC_AC	79	-0.3374098	-1.3027446	0.06744186	0.14501526	0.08711709	14747	tags=56%, list=40%, signal=33%
PID_TAP63_F	PID_TAP63_F	53	-0.3910863	-1.3994833	0.06759907	0.14514527	0.08719519	3557	tags=25%, list=10%, signal=22%
REACTOME_I	REACTOME_I	38	0.45069726	1.43540457	0.06764168	0.14514527	0.08719519	2442	tags=16%, list=7%, signal=15%
KEGG_PYRU\	KEGG_PYRU\	40	-0.4170615	-1.4054413	0.06768559	0.14514527	0.08719519	16044	tags=80%, list=44%, signal=45%
BIOCARTA_M	BIOCARTA_M	81	0.36926317	1.34673842	0.06806283	0.14582261	0.0876021	4535	tags=14%, list=12%, signal=12%

REACTOME_!	REACTOME_!	78	0.37112695	1.33883535	0.06818182	0.14594595	0.08767619	3515	tags=15%, list=10%, signal=14%
PID_NCADHE	PID_NCADHE	33	0.46353097	1.44592341	0.06826568	0.14599394	0.08770502	8038	tags=39%, list=22%, signal=31%
WP_DRUG_II	WP_DRUG_II	17	-0.5248402	-1.4480143	0.06854839	0.14638429	0.08793953	9782	tags=59%, list=27%, signal=43%
WP_CANONI	WP_CANONI	27	0.49171505	1.46223613	0.06857143	0.14638429	0.08793953	6106	tags=37%, list=17%, signal=31%
WP_DOPAMI	WP_DOPAMI	30	0.47850896	1.45873371	0.0689013	0.14695646	0.08828325	4910	tags=30%, list=13%, signal=26%
BIOCARTA_C	BIOCARTA_C	19	0.53195558	1.45734768	0.06916996	0.14726508	0.08846865	8305	tags=63%, list=23%, signal=49%
PID_SYNDEC	PID_SYNDEC	17	0.55814465	1.48113806	0.06916996	0.14726508	0.08846865	8111	tags=47%, list=22%, signal=37%
REACTOME_I	REACTOME_I	183	0.30330029	1.26615786	0.06946688	0.14776483	0.08876888	3024	tags=14%, list=8%, signal=13%
WP_BASE_EX	WP_BASE_EX	31	-0.4357707	-1.3927514	0.06956522	0.14784164	0.08881502	12507	tags=71%, list=34%, signal=47%
BIOCARTA_N	BIOCARTA_N	20	0.5237345	1.45270433	0.0703125	0.14911309	0.08957884	1801	tags=15%, list=5%, signal=14%
REACTOME_I	REACTOME_I	38	-0.426396	-1.4179758	0.07032967	0.14911309	0.08957884	3352	tags=32%, list=9%, signal=29%
WP_ENDODE	WP_ENDODE	144	0.31722925	1.28104431	0.07035176	0.14911309	0.08957884	4867	tags=19%, list=13%, signal=17%
KEGG_SELEN	KEGG_SELEN	25	-0.4608445	-1.4263253	0.07068607	0.14955486	0.08984423	9702	tags=80%, list=26%, signal=59%
REACTOME_(	REACTOME_(	25	-0.4592587	-1.4214172	0.07068607	0.14955486	0.08984423	17600	tags=80%, list=48%, signal=42%
PID_PI3K_PL(	PID_PI3K_PL(	36	0.44973708	1.41828837	0.0707804	0.1496212	0.08988408	6180	tags=28%, list=17%, signal=23%
WP_ONE_CA	WP_ONE_CA	30	-0.437263	-1.3900234	0.07096774	0.14985528	0.0900247	13040	tags=63%, list=35%, signal=41%
REACTOME_/	REACTOME_/	25	0.49385651	1.44278053	0.07101727	0.14985528	0.0900247	4356	tags=28%, list=12%, signal=25%
BIOCARTA_PI	BIOCARTA_PI	11	0.6165844	1.47904979	0.0712831	0.15028273	0.09028149	7243	tags=45%, list=20%, signal=37%
KEGG_GLYO)	KEGG_GLYO)	16	-0.5543016	-1.4922819	0.07142857	0.15045593	0.09038554	12065	tags=81%, list=33%, signal=55%
WP_TYPE_II_	WP_TYPE_II_	37	0.44656387	1.40984567	0.07169118	0.15087532	0.09063749	8058	tags=43%, list=22%, signal=34%
REACTOME_T	REACTOME_T	15	0.57543909	1.48846603	0.072	0.15139115	0.09094737	4103	tags=33%, list=11%, signal=30%
WP_INTRACE	WP_INTRACE	27	0.48582959	1.44473428	0.07238095	0.1520576	0.09134773	1772	tags=15%, list=5%, signal=14%
REACTOME_2	REACTOME_2	17	-0.5211479	-1.4378274	0.07258065	0.15234241	0.09151883	5301	tags=71%, list=14%, signal=60%
PID_RET_PAT	PID_RET_PAT	39	0.44444689	1.41517734	0.07326007	0.15363277	0.09229401	8337	tags=33%, list=23%, signal=26%
BIOCARTA_A	BIOCARTA_A	20	-0.4853784	-1.4132246	0.07346939	0.15393586	0.09247609	371	tags=35%, list=1%, signal=35%
PID_VEGFR1_	PID_VEGFR1_	69	0.38434053	1.36175388	0.07388316	0.15466642	0.09291497	6322	tags=23%, list=17%, signal=19%
BIOCARTA_CI	BIOCARTA_CI	15	0.57458947	1.48626834	0.074	0.15477465	0.09297999	8255	tags=47%, list=22%, signal=36%
REACTOME_(	REACTOME_(	22	-0.4804229	-1.4325265	0.07453416	0.15574863	0.0935651	18092	tags=86%, list=49%, signal=44%
REACTOME_T	REACTOME_T	19	-0.4940383	-1.4176832	0.07459677	0.15574863	0.0935651	17600	tags=84%, list=48%, signal=44%
WP_IL6_SIGN	WP_IL6_SIGN	43	0.42629572	1.37520529	0.0749543	0.15635769	0.09393099	8058	tags=35%, list=22%, signal=27%
WP_SOMATI	WP_SOMATI	14	0.57545547	1.48253973	0.07509881	0.15652174	0.09402954	3240	tags=29%, list=9%, signal=26%
REACTOME_T	REACTOME_T	34	-0.4264041	-1.387604	0.07658643	0.15924096	0.0956631	17600	tags=74%, list=48%, signal=38%
WP_FATTY_A	WP_FATTY_A	34	-0.426191	-1.3869103	0.07658643	0.15924096	0.0956631	12620	tags=56%, list=34%, signal=37%
REACTOME_I	REACTOME_I	21	-0.4887167	-1.4333118	0.07660455	0.15924096	0.0956631	16644	tags=81%, list=45%, signal=44%
REACTOME_I	REACTOME_I	26	-0.4553265	-1.4024303	0.07692308	0.15976331	0.0959769	3348	tags=35%, list=9%, signal=31%
REACTOME_I	REACTOME_I	29	-0.4448494	-1.4040026	0.07708779	0.15996559	0.09609841	17690	tags=72%, list=48%, signal=38%
REACTOME_I	REACTOME_I	37	0.44420607	1.40240186	0.07720588	0.16007083	0.09616164	9284	tags=46%, list=25%, signal=34%
REACTOME_/	REACTOME_/	24	-0.4610819	-1.4085703	0.07786885	0.16113281	0.09679962	16950	tags=92%, list=46%, signal=50%
WP_PATHW/	WP_PATHW/	85	0.35291805	1.30431886	0.0779896	0.16113281	0.09679962	4984	tags=21%, list=14%, signal=18%
BIOCARTA_P(	BIOCARTA_P(	15	0.56914546	1.47218652	0.078	0.16113281	0.09679962	5616	tags=33%, list=15%, signal=28%
WP_ENDOMI	WP_ENDOMI	63	0.39211764	1.36277724	0.07801418	0.16113281	0.09679962	3950	tags=16%, list=11%, signal=14%
REACTOME_I	REACTOME_I	16	0.56274677	1.47557226	0.078125	0.16113281	0.09679962	1243	tags=12%, list=3%, signal=12%
WP_HYPERTI	WP_HYPERTI	20	0.51145384	1.41864094	0.078125	0.16113281	0.09679962	2032	tags=20%, list=6%, signal=19%
REACTOME_I	REACTOME_I	13	-0.5748756	-1.4552229	0.07831325	0.161381	0.09694871	5672	tags=54%, list=15%, signal=46%
REACTOME_T	REACTOME_T	12	0.60751524	1.48915779	0.07847082	0.16156558	0.0970596	3535	tags=33%, list=10%, signal=30%
PID_RHOA_P	PID_RHOA_P	45	0.41466512	1.34565644	0.07889908	0.16221602	0.09745035	6751	tags=31%, list=18%, signal=25%
BIOCARTA_G	BIOCARTA_G	14	0.570396	1.46950507	0.07905138	0.16221602	0.09745035	8305	tags=57%, list=23%, signal=44%
WP_OVERVIE	WP_OVERVIE	19	0.52306788	1.43299888	0.07905138	0.16221602	0.09745035	2589	tags=37%, list=7%, signal=34%
REACTOME_I	REACTOME_I	26	-0.4519998	-1.3921839	0.07905983	0.16221602	0.09745035	6304	tags=35%, list=17%, signal=29%
REACTOME_!	REACTOME_!	30	0.45991576	1.40205238	0.08007449	0.16415615	0.09861587	3225	tags=17%, list=9%, signal=15%
REACTOME_I	REACTOME_I	33	-0.4267047	-1.3801354	0.08043478	0.16475262	0.0989742	17243	tags=67%, list=47%, signal=36%
WP_DNA_DA	WP_DNA_DA	68	-0.3470517	-1.2999562	0.08056872	0.16488482	0.09905362	17277	tags=60%, list=47%, signal=32%
WP_FATTY_A	WP_FATTY_A	22	-0.4710173	-1.404481	0.08074534	0.16510407	0.09918533	12715	tags=64%, list=34%, signal=42%
BIOCARTA_H	BIOCARTA_H	14	0.56748137	1.46199615	0.08102767	0.16525471	0.09927582	6575	tags=36%, list=18%, signal=29%
REACTOME_I	REACTOME_I	14	0.56865282	1.46501414	0.08102767	0.16525471	0.09927582	2648	tags=43%, list=7%, signal=40%
REACTOME_I	REACTOME_I	14	0.55895198	1.44002196	0.08102767	0.16525471	0.09927582	1433	tags=14%, list=4%, signal=14%
REACTOME_T	REACTOME_T	39	-0.4178983	-1.4030252	0.08114035	0.1653426	0.09932862	13576	tags=51%, list=37%, signal=32%
KEGG_PYRIM	KEGG_PYRIM	98	-0.3171303	-1.2769725	0.08158508	0.16610639	0.09978746	13722	tags=55%, list=37%, signal=35%
REACTOME_(	REACTOME_(	23	-0.474889	-1.4253041	0.08176101	0.16632205	0.09991702	9620	tags=52%, list=26%, signal=39%
WP_HEPATIT	WP_HEPATIT	50	0.40435499	1.35524067	0.08231173	0.16723982	0.10046837	8495	tags=38%, list=23%, signal=29%
BIOCARTA_R.	BIOCARTA_R.	10	-0.6145031	-1.4150334	0.08235294	0.16723982	0.10046837	8511	tags=70%, list=23%, signal=54%
WP_SIGNALII	WP_SIGNALII	34	0.44624806	1.39917796	0.08256881	0.167535	0.10064569	8927	tags=35%, list=24%, signal=27%
REACTOME_T	REACTOME_T	100	0.33713604	1.27989393	0.08318891	0.16864919	0.10131503	6729	tags=26%, list=18%, signal=21%
BIOCARTA_A	BIOCARTA_A	11	0.60002025	1.43931604	0.08350305	0.16899766	0.10152438	6314	tags=36%, list=17%, signal=30%
WP_SEROTO	WP_SEROTO	11	0.59949568	1.43805771	0.08350305	0.16899766	0.10152438	2445	tags=36%, list=7%, signal=34%
WP_COVID19	WP_COVID19	15	0.55840444	1.44440315	0.084	0.16985872	0.10204166	8116	tags=73%, list=22%, signal=57%
REACTOME_V	REACTOME_V	26	0.47395717	1.40362255	0.08426966	0.1702511	0.10227737	5029	tags=27%, list=14%, signal=23%
WP_DISORDE	WP_DISORDE	13	-0.5606677	-1.4192576	0.08433735	0.1702511	0.10227737	12080	tags=69%, list=33%, signal=47%
BIOCARTA_PI	BIOCARTA_PI	12	0.58274864	1.42844922	0.08450704	0.17030427	0.10230932	4307	tags=42%, list=12%, signal=37%
REACTOME_I	REACTOME_I	12	0.58482506	1.43353902	0.08450704	0.17030427	0.10230932	10746	tags=67%, list=29%, signal=47%
REACTOME_(	REACTOME_(	60	0.39098849	1.34809111	0.08465608	0.17046005	0.1024029	4529	tags=30%, list=12%, signal=26%
REACTOME_(	REACTOME_(	25	-0.4470538	-1.3836426	0.08523909	0.17148863	0.10302081	12990	tags=64%, list=35%, signal=41%
REACTOME_I	REACTOME_I	76	-0.3347676	-1.2863854	0.08545035	0.17176821	0.10318877	16243	tags=61%, list=44%, signal=34%
REACTOME_!	REACTOME_!	18	0.53428654	1.44055168	0.08565737	0.17203881	0.10335133	6271	tags=33%, list=17%, signal=28%

REACTOME_I	REACTOME_I	80	-0.3252502	-1.2615877	0.08624709	0.17307692	0.10397497	17717	tags=62%, list=48%, signal=33%
REACTOME_I	REACTOME_I	35	-0.4164269	-1.3664818	0.0864745	0.17338685	0.10416116	6862	tags=37%, list=19%, signal=30%
REACTOME_I	REACTOME_I	23	0.48957024	1.40733331	0.08761905	0.17553361	0.10545081	3323	tags=26%, list=9%, signal=24%
REACTOME_(	REACTOME_(	11	-0.6012157	-1.4369119	0.08806262	0.17614679	0.10581918	14708	tags=100%, list=40%, signal=60%
WP_NEPHRO	WP_NEPHRO	45	0.4114911	1.33535624	0.08807339	0.17614679	0.10581918	4589	tags=22%, list=12%, signal=19%
BIOCARTA_C	BIOCARTA_C	10	-0.6103438	-1.4054557	0.08823529	0.17632217	0.10592454	14371	tags=100%, list=39%, signal=61%
REACTOME_S	REACTOME_S	21	0.49375652	1.38841392	0.08863198	0.17696605	0.10631134	3267	tags=24%, list=9%, signal=22%
REACTOME_(	REACTOME_(	16	0.55237194	1.44836855	0.08984375	0.17923489	0.10767433	6475	tags=38%, list=18%, signal=31%
REACTOME_I	REACTOME_I	55	0.39454044	1.33504663	0.09010601	0.17960728	0.10789804	5425	tags=22%, list=15%, signal=19%
REACTOME_I	REACTOME_I	28	0.46685691	1.40041991	0.09039548	0.17975266	0.10798538	9003	tags=50%, list=24%, signal=38%
REACTOME_S	REACTOME_S	33	0.44503345	1.38822287	0.0904059	0.17975266	0.10798538	6008	tags=24%, list=16%, signal=20%
WP_TUMOR_	WP_TUMOR_	31	0.45111084	1.38776489	0.0904059	0.17975266	0.10798538	3823	tags=19%, list=10%, signal=17%
WP_NUCLEA	WP_NUCLEA	318	-0.2489875	-1.1735135	0.09064327	0.18007393	0.10817838	5301	tags=22%, list=14%, signal=19%
REACTOME_I	REACTOME_I	39	0.42580079	1.35580573	0.09157509	0.18177311	0.10919916	6817	tags=38%, list=18%, signal=31%
REACTOME_S	REACTOME_S	87	0.34425171	1.27911514	0.09217391	0.18280903	0.10982148	4380	tags=15%, list=12%, signal=13%
BIOCARTA_R	BIOCARTA_R	21	0.48911359	1.37535826	0.09248555	0.18312139	0.11000913	3970	tags=24%, list=11%, signal=21%
REACTOME_I	REACTOME_I	21	0.49061431	1.3795782	0.09248555	0.18312139	0.11000913	2289	tags=24%, list=6%, signal=22%
REACTOME_(	REACTOME_(	71	-0.3389506	-1.2715373	0.09263658	0.1832677	0.11009703	18682	tags=76%, list=51%, signal=38%
PID_CDC42_I	PID_CDC42_I	30	0.45109436	1.37516036	0.09310987	0.18405079	0.11056746	8038	tags=37%, list=22%, signal=29%
REACTOME_I	REACTOME_I	15	-0.5294182	-1.4071477	0.0936255	0.18480205	0.11101878	17356	tags=100%, list=47%, signal=53%
WP_EGFEGFI	WP_EGFEGFI	161	0.30315972	1.23795267	0.09364548	0.18480205	0.11101878	9009	tags=27%, list=24%, signal=21%
REACTOME_I	REACTOME_I	26	-0.4454473	-1.3720019	0.09401709	0.18538142	0.11136683	13349	tags=69%, list=36%, signal=44%
REACTOME_I	REACTOME_I	43	-0.3867544	-1.3245394	0.09450549	0.18615754	0.11183308	18420	tags=67%, list=50%, signal=34%
REACTOME_I	REACTOME_I	12	0.57549664	1.41067293	0.0945674	0.18615754	0.11183308	7348	tags=50%, list=20%, signal=40%
PID_THROME	PID_THROME	15	0.54856988	1.41896447	0.096	0.18882119	0.11343325	4356	tags=33%, list=12%, signal=29%
WP_PHOTOD	WP_PHOTOD	50	0.39924602	1.3381174	0.09632224	0.18926616	0.11370056	6751	tags=28%, list=18%, signal=23%
BIOCARTA_R	BIOCARTA_R	13	-0.5550851	-1.4051258	0.09638554	0.18926616	0.11370056	7790	tags=46%, list=21%, signal=36%
WP_NOVEL_I	WP_NOVEL_I	60	0.38640955	1.33230338	0.09700176	0.1903189	0.11433299	10090	tags=40%, list=27%, signal=29%
REACTOME_(	REACTOME_(	62	-0.346747	-1.2753859	0.09722222	0.19052406	0.11445624	7872	tags=37%, list=21%, signal=29%
ST_B_CELL_A	ST_B_CELL_A	40	0.4169603	1.3350758	0.09742647	0.19052406	0.11445624	4141	tags=18%, list=11%, signal=16%
REACTOME_/	REACTOME_/	10	0.5881566	1.37752734	0.09756098	0.19052406	0.11445624	6751	tags=30%, list=18%, signal=25%
REACTOME_S	REACTOME_S	10	0.58912366	1.37979231	0.09756098	0.19052406	0.11445624	10400	tags=50%, list=28%, signal=36%
WP_TYPE_III_	WP_TYPE_III_	10	0.58971884	1.38118629	0.09756098	0.19052406	0.11445624	8058	tags=50%, list=22%, signal=39%
REACTOME_I	REACTOME_I	15	-0.5267857	-1.4001508	0.09760956	0.19052406	0.11445624	16945	tags=87%, list=46%, signal=47%
REACTOME_I	REACTOME_I	384	0.26279492	1.18153649	0.09766764	0.19052406	0.11445624	6250	tags=18%, list=17%, signal=15%
WP_NOTCH_	WP_NOTCH_	45	0.40455326	1.31284181	0.09908257	0.19312566	0.11601914	8936	tags=40%, list=24%, signal=30%
REACTOME_I	REACTOME_I	33	0.44004792	1.37267115	0.099631	0.19403545	0.11656569	3449	tags=18%, list=9%, signal=16%
REACTOME_I	REACTOME_I	79	-0.3238434	-1.2503646	0.1	0.19459459	0.11690159	19026	tags=72%, list=52%, signal=35%
REACTOME_T	REACTOME_T	153	0.30256045	1.23306538	0.10033445	0.19508564	0.11719658	7062	tags=26%, list=19%, signal=21%
KEGG_RETIN	KEGG_RETIN	64	-0.3396	-1.2635619	0.10045662	0.19516348	0.11724334	5905	tags=27%, list=16%, signal=22%
KEGG_GLYCO	KEGG_GLYCO	14	0.547596	1.41076567	0.10079051	0.19565217	0.11753693	4849	tags=29%, list=13%, signal=25%
BIOCARTA_EI	BIOCARTA_EI	27	0.46584566	1.38530712	0.10095238	0.19580641	0.11762958	1813	tags=11%, list=5%, signal=11%
REACTOME_I	REACTOME_I	32	0.45012691	1.3902319	0.10166359	0.19702502	0.11836166	2904	tags=25%, list=8%, signal=23%
REACTOME_I	REACTOME_I	18	-0.4870944	-1.3738229	0.102	0.19751589	0.11865654	14958	tags=72%, list=41%, signal=43%
KEGG_ERBB_	KEGG_ERBB_	87	0.34034152	1.26458629	0.1026087	0.19853279	0.11926744	5099	tags=21%, list=14%, signal=18%
BIOCARTA_BI	BIOCARTA_BI	29	0.45518846	1.37701584	0.10280374	0.19874832	0.11939692	3384	tags=14%, list=9%, signal=13%
WP_NAD_ME	WP_NAD_ME	16	0.53423085	1.40080098	0.10351563	0.19996189	0.12012596	2022	tags=12%, list=5%, signal=12%
BIOCARTA_A	BIOCARTA_A	15	0.54549125	1.41100109	0.104	0.20057143	0.12049214	5280	tags=33%, list=14%, signal=29%
REACTOME_I	REACTOME_I	18	-0.4867642	-1.3728915	0.104	0.20057143	0.12049214	14930	tags=67%, list=41%, signal=40%
KEGG_AMYO	KEGG_AMYO	53	0.38996961	1.31575664	0.10471204	0.20178087	0.1212187	7418	tags=28%, list=20%, signal=23%
REACTOME_I	REACTOME_I	128	-0.2917135	-1.2215445	0.10526316	0.2026785	0.12175795	14520	tags=62%, list=39%, signal=38%
REACTOME_I	REACTOME_I	21	-0.464858	-1.3633387	0.10559006	0.20314331	0.12203718	16950	tags=95%, list=46%, signal=51%
REACTOME_I	REACTOME_I	176	-0.2676434	-1.1833924	0.10579345	0.20336993	0.12217333	12708	tags=50%, list=34%, signal=33%
WP_RANKLR/	WP_RANKLR/	55	0.3912921	1.32405487	0.10600707	0.20361584	0.12232105	10333	tags=44%, list=28%, signal=31%
KEGG_STARC	KEGG_STARC	52	-0.3605453	-1.2882833	0.10633484	0.20408044	0.12260016	7208	tags=31%, list=20%, signal=25%
REACTOME_I	REACTOME_I	53	0.38805164	1.30928541	0.10645724	0.20415045	0.12264222	3225	tags=17%, list=9%, signal=16%
WP_PROTEO	WP_PROTEO	17	0.52891409	1.40356947	0.10671937	0.20422469	0.12268681	6452	tags=35%, list=18%, signal=29%
WP_EICOSAN	WP_EICOSAN	26	0.46562304	1.37894106	0.10674157	0.20422469	0.12268681	4498	tags=27%, list=12%, signal=24%
REACTOME_T	REACTOME_T	44	-0.384848	-1.326943	0.10675381	0.20422469	0.12268681	3182	tags=25%, list=9%, signal=23%
PID_ERBB_NI	PID_ERBB_NI	15	-0.5206841	-1.3839334	0.10756972	0.20557384	0.12349731	8356	tags=60%, list=23%, signal=46%
REACTOME_S	REACTOME_S	11	-0.5821201	-1.3912732	0.10763209	0.20557384	0.12349731	14257	tags=82%, list=39%, signal=50%
BIOCARTA_EI	BIOCARTA_EI	14	0.53978909	1.39065282	0.10869565	0.20721509	0.12448328	9009	tags=43%, list=24%, signal=32%
BIOCARTA_TI	BIOCARTA_TI	17	0.5277342	1.4004384	0.10869565	0.20721509	0.12448328	8255	tags=41%, list=22%, signal=32%
PID_NEPHRIN	PID_NEPHRIN	31	0.4392886	1.35139578	0.10885609	0.20721509	0.12448328	8038	tags=32%, list=22%, signal=25%
REACTOME_I	REACTOME_I	31	0.44200861	1.35976344	0.10885609	0.20721509	0.12448328	4886	tags=32%, list=13%, signal=28%
REACTOME_I	REACTOME_I	26	-0.4343421	-1.3377973	0.10897436	0.20721509	0.12448328	13266	tags=54%, list=36%, signal=34%
REACTOME_/	REACTOME_/	23	-0.4471473	-1.3420418	0.10901468	0.20721509	0.12448328	13684	tags=57%, list=37%, signal=36%
REACTOME_I	REACTOME_I	37	-0.4035034	-1.3407202	0.10917031	0.20734504	0.12456135	17837	tags=73%, list=48%, signal=38%
BIOCARTA_ST	BIOCARTA_ST	15	0.5354533	1.38503633	0.11	0.20875399	0.12540777	8305	tags=73%, list=23%, signal=57%
SIG_CD40PAT	SIG_CD40PAT	34	0.42833385	1.34300927	0.11009174	0.20876136	0.12541219	8255	tags=32%, list=22%, signal=25%
REACTOME_I	REACTOME_I	33	-0.4091345	-1.3233063	0.11086957	0.21006865	0.12619754	17225	tags=67%, list=47%, signal=36%
WP_TLR4_SI(	WP_TLR4_SI(	28	0.45434319	1.36288281	0.11111111	0.21035857	0.12637171	10335	tags=43%, list=28%, signal=31%
WP_CORTICO	WP_CORTICO	93	0.33268711	1.24721772	0.11130742	0.21043407	0.12641706	6098	tags=28%, list=17%, signal=23%
BIOCARTA_ST	BIOCARTA_ST	20	0.48576884	1.34739738	0.11132813	0.21043407	0.12641706	5185	tags=40%, list=14%, signal=34%

REACTOME_I	REACTOME_I	18	0.51326304	1.3838678	0.11155378	0.21062635	0.12653257	7348	tags=50%, list=20%, signal=40%
REACTOME_S	REACTOME_S	47	-0.3758383	-1.3123171	0.11160714	0.21062635	0.12653257	17862	tags=79%, list=48%, signal=41%
BIOCARTA_AT	BIOCARTA_AT	21	-0.460792	-1.3514139	0.11180124	0.2108252	0.12665203	15932	tags=76%, list=43%, signal=43%
PID_HIF2PAT	PID_HIF2PAT	34	0.42758167	1.34065088	0.11192661	0.21086529	0.12667612	4984	tags=26%, list=14%, signal=23%
REACTOME_A	REACTOME_A	18	-0.4829173	-1.3620415	0.112	0.21086529	0.12667612	6433	tags=50%, list=17%, signal=41%
WP_ROLE_OI	WP_ROLE_OI	29	0.44582405	1.34868705	0.11214953	0.21097964	0.12674482	6739	tags=45%, list=18%, signal=37%
REACTOME_I	REACTOME_I	113	0.31672578	1.22649123	0.11262799	0.2117121	0.12718483	6048	tags=19%, list=16%, signal=16%
BIOCARTA_EI	BIOCARTA_EI	24	0.46256061	1.33298524	0.11284047	0.21194383	0.12732405	665	tags=8%, list=2%, signal=8%
REACTOME_(	REACTOME_(	75	-0.3257633	-1.2444664	0.11320755	0.21226834	0.12751899	17246	tags=67%, list=47%, signal=36%
REACTOME_I	REACTOME_I	16	0.52711945	1.38215426	0.11328125	0.21226834	0.12751899	5327	tags=31%, list=14%, signal=27%
REACTOME_S	REACTOME_S	16	0.52784276	1.38405084	0.11328125	0.21226834	0.12751899	5880	tags=38%, list=16%, signal=32%
REACTOME_I	REACTOME_I	11	-0.5799223	-1.3860206	0.11350294	0.21251613	0.12766785	12961	tags=73%, list=35%, signal=47%
REACTOME_I	REACTOME_I	53	-0.3518868	-1.2592098	0.11421911	0.21364505	0.12834604	16767	tags=66%, list=45%, signal=36%
REACTOME_I	REACTOME_I	20	-0.4592547	-1.337163	0.11428571	0.21364505	0.12834604	19749	tags=95%, list=54%, signal=44%
REACTOME_I	REACTOME_I	18	0.50042418	1.34925146	0.11553785	0.21581598	0.12965022	6271	tags=33%, list=17%, signal=28%
KEGG_FRUCT	KEGG_FRUCT	34	-0.4060558	-1.3213864	0.11597374	0.21646002	0.13003712	14137	tags=59%, list=38%, signal=36%
WP_OVARIAI	WP_OVARIAI	32	0.43694564	1.34952109	0.11645102	0.21718023	0.13046978	6737	tags=28%, list=18%, signal=23%
BIOCARTA_M	BIOCARTA_M	18	0.50039842	1.349182	0.11752988	0.21902039	0.13157525	1841	tags=28%, list=5%, signal=26%
REACTOME_S	REACTOME_S	86	0.33415197	1.24188521	0.11764706	0.21906694	0.13160321	5808	tags=17%, list=16%, signal=15%
PID_E2F_PAT	PID_E2F_PAT	73	-0.3282898	-1.2446984	0.11820331	0.21993036	0.1321219	11558	tags=47%, list=31%, signal=32%
WP_ARYL_HY	WP_ARYL_HY	46	0.39371963	1.289848	0.11839709	0.22011853	0.13223495	3090	tags=13%, list=8%, signal=12%
REACTOME_I	REACTOME_I	19	0.49009058	1.34265415	0.11857708	0.22028079	0.13233243	3506	tags=16%, list=10%, signal=14%
PID_NECTIN_	PID_NECTIN_	30	0.43614729	1.32959424	0.11918063	0.22122905	0.13290209	5879	tags=30%, list=16%, signal=25%
WP_MAMMA	WP_MAMMA	18	0.49804566	1.34283845	0.11952191	0.22159669	0.13312295	3204	tags=28%, list=9%, signal=25%
REACTOME_I	REACTOME_I	31	-0.4123903	-1.3180261	0.11956522	0.22159669	0.13312295	12238	tags=68%, list=33%, signal=45%
KEGG_GLIOM	KEGG_GLIOM	65	0.36765236	1.28859455	0.12	0.22222915	0.13350289	4373	tags=22%, list=12%, signal=19%
REACTOME_I	REACTOME_I	13	0.54685925	1.38067945	0.12103175	0.22396529	0.13454587	523	tags=23%, list=1%, signal=23%
WP_THE_ALT	WP_THE_ALT	11	-0.5737575	-1.3712867	0.12133072	0.22434381	0.13477326	2968	tags=45%, list=8%, signal=42%
BIOCARTA_ET	BIOCARTA_ET	10	-0.5902403	-1.3591628	0.12156863	0.22446152	0.13484397	10559	tags=70%, list=29%, signal=50%
WP_PHOTOD	WP_PHOTOD	26	-0.4255662	-1.3107668	0.12179487	0.22446152	0.13484397	16950	tags=65%, list=46%, signal=35%
BIOCARTA_AT	BIOCARTA_AT	27	0.44865202	1.33417759	0.12190476	0.22446152	0.13484397	3384	tags=11%, list=9%, signal=10%
BIOCARTA_IL	BIOCARTA_IL	15	0.52379664	1.35488448	0.122	0.22446152	0.13484397	5088	tags=27%, list=14%, signal=23%
BIOCARTA_RI	BIOCARTA_RI	15	0.52417411	1.35586088	0.122	0.22446152	0.13484397	11617	tags=67%, list=32%, signal=46%
REACTOME_A	REACTOME_A	18	-0.4726966	-1.3332146	0.122	0.22446152	0.13484397	5477	tags=44%, list=15%, signal=38%
KEGG_RNA_F	KEGG_RNA_F	29	-0.4136671	-1.3055873	0.12205567	0.22446152	0.13484397	16196	tags=62%, list=44%, signal=35%
REACTOME_I	REACTOME_I	30	0.43447209	1.32448741	0.12290503	0.22584868	0.13567731	7348	tags=33%, list=20%, signal=27%
WP_INTERLE	WP_INTERLE	44	0.40215474	1.30142336	0.12338858	0.22656203	0.13610585	6889	tags=27%, list=19%, signal=22%
REACTOME_I	REACTOME_I	15	-0.5101807	-1.3560163	0.12350598	0.22660247	0.13613014	13242	tags=87%, list=36%, signal=56%
WP_RAC1PAI	WP_RAC1PAI	68	0.35942543	1.26799576	0.12413793	0.22758621	0.13672111	5571	tags=21%, list=15%, signal=18%
KEGG_NON_	KEGG_NON_	13	-0.5294259	-1.3401729	0.12449799	0.22796295	0.13694744	15324	tags=77%, list=42%, signal=45%
WP_TRYPTOI	WP_TRYPTOI	42	0.39643204	1.26747526	0.12453532	0.22796295	0.13694744	4513	tags=19%, list=12%, signal=17%
REACTOME_I	REACTOME_I	65	0.36444366	1.27734828	0.12695652	0.2321464	0.13946062	6331	tags=25%, list=17%, signal=20%
REACTOME_I	REACTOME_I	19	-0.461832	-1.3252647	0.12701613	0.2321464	0.13946062	15524	tags=58%, list=42%, signal=34%
REACTOME_A	REACTOME_A	42	-0.3785715	-1.2951473	0.12715517	0.2322219	0.13950598	14081	tags=57%, list=38%, signal=35%
REACTOME_I	REACTOME_I	26	0.45376959	1.34383711	0.12734082	0.23238233	0.13960236	5923	tags=42%, list=16%, signal=36%
REACTOME_I	REACTOME_I	38	-0.3913766	-1.3015191	0.12747253	0.23244415	0.13963949	371	tags=11%, list=1%, signal=10%
KEGG_TYROS	KEGG_TYROS	42	0.39487925	1.26251068	0.12825279	0.2336876	0.14038649	1060	tags=14%, list=3%, signal=14%
WP_SEROTO	WP_SEROTO	17	0.51338459	1.36235911	0.1284585	0.23388306	0.14050391	5094	tags=41%, list=14%, signal=36%
REACTOME_I	REACTOME_I	57	-0.338355	-1.2206178	0.12903226	0.2346812	0.14098339	15617	tags=70%, list=42%, signal=41%
REACTOME_(	REACTOME_(	21	0.46780383	1.31543648	0.12909441	0.2346812	0.14098339	5996	tags=29%, list=16%, signal=24%
WP_MET_IN_	WP_MET_IN_	59	0.37269191	1.2823419	0.1295972	0.23541509	0.14142427	8757	tags=31%, list=24%, signal=23%
REACTOME_S	REACTOME_S	451	0.25073801	1.14465793	0.13	0.23588189	0.1417047	4542	tags=14%, list=12%, signal=13%
KEGG_VEGF_	KEGG_VEGF_	76	0.34905983	1.25325839	0.13005272	0.23588189	0.1417047	5571	tags=24%, list=15%, signal=20%
WP_VALPROI	WP_VALPROI	13	-0.5260274	-1.33157	0.13052209	0.23655262	0.14210763	12342	tags=62%, list=33%, signal=41%
REACTOME_I	REACTOME_I	12	-0.5420259	-1.3431539	0.13069307	0.23668196	0.14218534	14042	tags=100%, list=38%, signal=62%
REACTOME_I	REACTOME_I	40	-0.3836091	-1.2927114	0.13100437	0.23691504	0.14232535	92	tags=8%, list=0%, signal=7%
BIOCARTA_CI	BIOCARTA_CI	22	0.46179242	1.31496512	0.13102119	0.23691504	0.14232535	7348	tags=27%, list=20%, signal=22%
WP_REGULAT	WP_REGULAT	46	0.38775264	1.27029982	0.13114754	0.23696316	0.14235427	5665	tags=20%, list=15%, signal=17%
REACTOME_A	REACTOME_A	33	-0.39398	-1.2742908	0.1326087	0.23931942	0.14376977	9953	tags=45%, list=27%, signal=33%
REACTOME_A	REACTOME_A	20	-0.4501355	-1.3106115	0.13265306	0.23931942	0.14376977	19749	tags=90%, list=54%, signal=42%
WP_VITAMIN	WP_VITAMIN	22	0.46023112	1.31051927	0.13294798	0.23951613	0.14388795	6575	tags=23%, list=18%, signal=19%
PID_BETA_CA	PID_BETA_CA	17	-0.4834497	-1.3338194	0.13306452	0.23951613	0.14388795	2008	tags=53%, list=5%, signal=50%
REACTOME_I	REACTOME_I	17	-0.4820324	-1.329909	0.13306452	0.23951613	0.14388795	14616	tags=65%, list=40%, signal=39%
KEGG_GNRH_	KEGG_GNRH_	101	0.32091305	1.22034624	0.13321799	0.23961086	0.14394486	4110	tags=18%, list=11%, signal=16%
REACTOME_(	REACTOME_(	42	0.3937592	1.25892963	0.133829	0.24052776	0.14449568	5236	tags=26%, list=14%, signal=22%
REACTOME_T	REACTOME_T	16	-0.4983823	-1.3417369	0.13469388	0.24189921	0.14531957	14145	tags=69%, list=38%, signal=42%
WP_GLYCOLY	WP_GLYCOLY	44	-0.3693858	-1.2736298	0.13507625	0.2424027	0.14562204	13882	tags=70%, list=38%, signal=44%
REACTOME_I	REACTOME_I	55	-0.341639	-1.2319761	0.1353211	0.24256555	0.14571987	14454	tags=51%, list=39%, signal=31%
REACTOME_I	REACTOME_I	37	-0.3856631	-1.281442	0.13537118	0.24256555	0.14571987	11867	tags=49%, list=32%, signal=33%
REACTOME_I	REACTOME_I	29	0.43514701	1.31638734	0.1364486	0.24431188	0.14676897	3690	tags=24%, list=10%, signal=22%
REACTOME_(	REACTOME_(	10	-0.5798336	-1.3351992	0.1372549	0.24557052	0.14752508	5277	tags=50%, list=14%, signal=43%
REACTOME_I	REACTOME_I	40	-0.3809401	-1.2837173	0.13755459	0.24592152	0.14773595	1859	tags=20%, list=5%, signal=19%
WP_EXERCISI	WP_EXERCISI	48	0.38306499	1.27242267	0.13780919	0.24608017	0.14783126	3618	tags=17%, list=10%, signal=15%
REACTOME_I	REACTOME_I	77	-0.312025	-1.1982248	0.13785047	0.24608017	0.14783126	12340	tags=44%, list=33%, signal=29%

WP_PRADER\	WP_PRADER\	63	0.36140235	1.2560284	0.13829787	0.2466935	0.14819971	2960	tags=17%, list=8%, signal=16%
WP_SEROTO	WP_SEROTO	20	0.47670304	1.32225119	0.13867188	0.24717508	0.14848901	1981	tags=15%, list=5%, signal=14%
REACTOME_2	REACTOME_2	10	-0.5771635	-1.3290506	0.13921569	0.24795837	0.14895958	5301	tags=80%, list=14%, signal=69%
REACTOME_I	REACTOME_I	15	-0.5005923	-1.330531	0.13944223	0.24817584	0.14909022	8570	tags=80%, list=23%, signal=61%
KEGG_GLYCC	KEGG_GLYCC	15	0.51001082	1.31922523	0.14	0.24898204	0.14957454	9623	tags=53%, list=26%, signal=39%
REACTOME_I	REACTOME_I	19	0.47607508	1.30425721	0.14031621	0.24935774	0.14980024	4009	tags=32%, list=11%, signal=28%
REACTOME_S	REACTOME_S	20	0.47490309	1.3172586	0.140625	0.24958666	0.14993776	1809	tags=20%, list=5%, signal=19%
WP_EXTRACE	WP_EXTRACE	22	0.45554524	1.29717612	0.14065511	0.24958666	0.14993776	4781	tags=32%, list=13%, signal=28%
WP_UREA_CY	WP_UREA_CY	21	-0.4428042	-1.2986593	0.14078675	0.24963382	0.14996609	14661	tags=71%, list=40%, signal=43%
WP_NCRNAS	WP_NCRNAS	17	0.50396253	1.33735598	0.14229249	0.25211555	0.15145698	2940	tags=29%, list=8%, signal=27%
REACTOME_I	REACTOME_I	11	0.55179295	1.3236294	0.14256619	0.25241227	0.15163523	4356	tags=27%, list=12%, signal=24%
WP_RETT_SY	WP_RETT_SY	48	0.38010243	1.26258195	0.14310954	0.25318561	0.15209981	7002	tags=29%, list=19%, signal=24%
WP_EXTRACE	WP_EXTRACE	30	0.42460623	1.29441134	0.1433892	0.25336247	0.15220605	4850	tags=33%, list=13%, signal=29%
REACTOME_I	REACTOME_I	24	-0.4203943	-1.2842729	0.14344262	0.25336247	0.15220605	9834	tags=38%, list=27%, signal=28%
REACTOME_(	REACTOME_(	100	-0.2944686	-1.1870994	0.14352941	0.25336247	0.15220605	13945	tags=53%, list=38%, signal=33%
WP_SEROTO	WP_SEROTO	13	0.53176977	1.34258237	0.14484127	0.25548839	0.15348319	5838	tags=54%, list=16%, signal=45%
REACTOME_I	REACTOME_I	29	-0.4038588	-1.274631	0.14561028	0.25665432	0.15418362	13678	tags=62%, list=37%, signal=39%
WP_BILE_ACI	WP_BILE_ACI	12	-0.5340417	-1.3233688	0.14653465	0.25789451	0.15492865	8607	tags=50%, list=23%, signal=38%
REACTOME_(	REACTOME_(	11	0.54692341	1.31194845	0.14663951	0.25789451	0.15492865	8606	tags=45%, list=23%, signal=35%
REACTOME_I	REACTOME_I	11	0.54798024	1.31448356	0.14663951	0.25789451	0.15492865	4294	tags=36%, list=12%, signal=32%
WP_RENIN_A	WP_RENIN_A	44	0.38993975	1.26189412	0.14732965	0.25886697	0.15551285	5701	tags=36%, list=15%, signal=31%
BIOCARTA_4	BIOCARTA_4	18	0.48379584	1.30441787	0.14741036	0.25886697	0.15551285	8170	tags=33%, list=22%, signal=26%
KEGG_DORS(	KEGG_DORS(	24	0.44737407	1.28922137	0.14785992	0.25946468	0.15587192	4163	tags=25%, list=11%, signal=22%
BIOCARTA_A	BIOCARTA_A	14	0.51200141	1.3190637	0.14822134	0.25990695	0.15613761	7348	tags=36%, list=20%, signal=29%
BIOCARTA_P	BIOCARTA_P	13	0.52483877	1.32508338	0.14880952	0.26070053	0.15661436	7884	tags=38%, list=21%, signal=30%
REACTOME_I	REACTOME_I	12	0.53188947	1.30378186	0.14889336	0.26070053	0.15661436	7324	tags=42%, list=20%, signal=33%
REACTOME_I	REACTOME_I	10	-0.5720047	-1.3171713	0.14901961	0.26072945	0.15663172	11546	tags=90%, list=31%, signal=62%
REACTOME_I	REACTOME_I	19	-0.4503498	-1.2923153	0.14919355	0.2608417	0.15669916	14967	tags=74%, list=41%, signal=44%
REACTOME_S	REACTOME_S	18	-0.4626944	-1.305004	0.15	0.26186627	0.15731467	161	tags=11%, list=0%, signal=11%
REACTOME_S	REACTOME_S	15	0.50631507	1.30966558	0.15	0.26186627	0.15731467	2150	tags=20%, list=6%, signal=19%
REACTOME_I	REACTOME_I	14	0.51181233	1.3185766	0.15019763	0.26198584	0.1573865	7993	tags=36%, list=22%, signal=28%
WP_PI3KAKT	WP_PI3KAKT	22	0.44884554	1.27809856	0.15028902	0.26198584	0.1573865	7394	tags=41%, list=20%, signal=33%
REACTOME_I	REACTOME_I	11	-0.5542043	-1.3245541	0.15068493	0.26244975	0.15766518	13742	tags=73%, list=37%, signal=46%
REACTOME_I	REACTOME_I	36	-0.3846042	-1.2702471	0.15077605	0.26244975	0.15766518	16813	tags=75%, list=46%, signal=41%
REACTOME_(	REACTOME_(	20	-0.4410402	-1.2841299	0.15102041	0.26268264	0.1578051	19749	tags=85%, list=54%, signal=39%
WP_WNT_SI(	WP_WNT_SI(	51	0.36978465	1.23264106	0.15124555	0.26276947	0.15785726	3851	tags=16%, list=10%, signal=14%
REACTOME_I	REACTOME_I	31	0.42286371	1.30086744	0.15129151	0.26276947	0.15785726	4826	tags=19%, list=13%, signal=17%
REACTOME_I	REACTOME_I	38	-0.3740258	-1.2438192	0.15164835	0.26319685	0.158114	18883	tags=74%, list=51%, signal=36%
KEGG_ENDO	KEGG_ENDO	52	0.37001668	1.23872331	0.15178571	0.26324296	0.1581417	3851	tags=15%, list=10%, signal=14%
REACTOME_S	REACTOME_S	20	0.46590127	1.29228986	0.15234375	0.26399061	0.15859085	5099	tags=25%, list=14%, signal=22%
REACTOME_/	REACTOME_/	10	0.55993369	1.3114262	0.15243902	0.26399061	0.15859085	8190	tags=40%, list=22%, signal=31%
REACTOME_I	REACTOME_I	31	0.4174008	1.28406173	0.15313653	0.26477419	0.15906158	5229	tags=19%, list=14%, signal=17%
REACTOME_S	REACTOME_S	17	-0.468864	-1.293578	0.15322581	0.26477419	0.15906158	16457	tags=94%, list=45%, signal=52%
REACTOME_T	REACTOME_T	14	-0.5006443	-1.2951088	0.15322581	0.26477419	0.15906158	12509	tags=57%, list=34%, signal=38%
REACTOME_/	REACTOME_/	38	-0.3730331	-1.240518	0.15384615	0.26565295	0.15958949	12109	tags=47%, list=33%, signal=32%
REACTOME_I	REACTOME_I	16	0.49563567	1.29960097	0.15429688	0.26623775	0.1599408	6877	tags=31%, list=19%, signal=25%
REACTOME_I	REACTOME_I	13	-0.5133467	-1.2994704	0.15461847	0.26659905	0.16015786	8356	tags=62%, list=23%, signal=48%
BIOCARTA_CI	BIOCARTA_CI	18	0.48087759	1.29654964	0.15537849	0.26767921	0.16080676	2618	tags=17%, list=7%, signal=15%
WP_IL7_SIGN	WP_IL7_SIGN	25	0.44072671	1.28756409	0.15547025	0.26767921	0.16080676	5571	tags=28%, list=15%, signal=24%
REACTOME_I	REACTOME_I	47	-0.3534334	-1.2340858	0.15625	0.26882694	0.16149625	17721	tags=66%, list=48%, signal=34%
WP_COMPUT	WP_COMPUT	11	-0.5523559	-1.3201366	0.15655577	0.26915812	0.1616952	15287	tags=82%, list=41%, signal=48%
REACTOME_S	REACTOME_S	45	-0.3579635	-1.2424089	0.15754923	0.27064278	0.1625871	92	tags=7%, list=0%, signal=7%
REACTOME_I	REACTOME_I	18	-0.4553773	-1.2843664	0.158	0.27064278	0.1625871	4618	tags=44%, list=13%, signal=39%
WP_GPR40_I	WP_GPR40_I	15	0.49773978	1.28748422	0.158	0.27064278	0.1625871	4141	tags=27%, list=11%, signal=24%
KEGG_TRYPT	KEGG_TRYPT	40	0.39469042	1.26376932	0.15808824	0.27064278	0.1625871	5655	tags=25%, list=15%, signal=21%
REACTOME_/	REACTOME_/	14	0.50674118	1.30551185	0.15810277	0.27064278	0.1625871	683	tags=7%, list=2%, signal=7%
WP_IL9_SIGN	WP_IL9_SIGN	17	0.49653597	1.31764824	0.15810277	0.27064278	0.1625871	8596	tags=47%, list=23%, signal=36%
REACTOME_S	REACTOME_S	49	0.37123683	1.23888502	0.15873016	0.27152114	0.16311477	6008	tags=24%, list=16%, signal=21%
REACTOME_U	REACTOME_U	11	0.54022302	1.29587569	0.15885947	0.27154684	0.16313021	2559	tags=18%, list=7%, signal=17%
REACTOME_S	REACTOME_S	34	0.40985203	1.28506088	0.15963303	0.27267295	0.16380672	5879	tags=21%, list=16%, signal=17%
REACTOME_I	REACTOME_I	15	0.49236446	1.27358009	0.16	0.27271162	0.16382995	5851	tags=47%, list=16%, signal=39%
REACTOME_I	REACTOME_I	85	-0.3007167	-1.1754086	0.16	0.27271162	0.16382995	18785	tags=73%, list=51%, signal=36%
REACTOME_V	REACTOME_V	27	0.42927864	1.27656606	0.16	0.27271162	0.16382995	4235	tags=22%, list=11%, signal=20%
WP_MAMMA	WP_MAMMA	13	0.51399077	1.29769496	0.16071429	0.27355322	0.16433553	2136	tags=38%, list=6%, signal=36%
REACTOME_I	REACTOME_I	10	-0.5644317	-1.2997327	0.16078431	0.27355322	0.16433553	1973	tags=30%, list=5%, signal=28%
PID_BETA_CA	PID_BETA_CA	78	0.33266589	1.20008759	0.16083916	0.27355322	0.16433553	4110	tags=19%, list=11%, signal=17%
WP_WNTBET	WP_WNTBET	26	0.43620619	1.29182316	0.16104869	0.27371365	0.16443191	5981	tags=31%, list=16%, signal=26%
REACTOME_S	REACTOME_S	62	0.35198555	1.22323784	0.16140351	0.27412061	0.16467639	7348	tags=24%, list=20%, signal=19%
REACTOME_S	REACTOME_S	37	0.39738633	1.25458739	0.16176471	0.27448602	0.16489591	8495	tags=35%, list=23%, signal=27%
BIOCARTA_P	BIOCARTA_P	21	0.44799173	1.25972605	0.16184971	0.27448602	0.16489591	3765	tags=14%, list=10%, signal=13%
REACTOME_/	REACTOME_/	52	-0.3390116	-1.2113401	0.16289593	0.27606328	0.16584344	12109	tags=40%, list=33%, signal=27%
KEGG_GLYCC	KEGG_GLYCC	61	-0.3288703	-1.204486	0.16397229	0.27764423	0.16679318	13991	tags=61%, list=38%, signal=38%
WP_OSTEOC	WP_OSTEOC	16	0.48998105	1.28477406	0.1640625	0.27764423	0.16679318	5865	tags=44%, list=16%, signal=37%
WP_LIPID_M	WP_LIPID_M	29	0.42230895	1.27755021	0.16448598	0.27816277	0.16710469	1069	tags=10%, list=3%, signal=10%

WP_TRANSSU	WP_TRANSSU	10	-0.5600267	-1.2895891	0.16470588	0.27833654	0.16720909		10694	tags=60%, list=29%, signal=43%
REACTOME_(	REACTOME_(	14	-0.4955751	-1.2819954	0.16532258	0.27898185	0.16759675		15814	tags=64%, list=43%, signal=37%
WP_MITOCH	WP_MITOCH	17	-0.4629654	-1.2773041	0.16532258	0.27898185	0.16759675		16368	tags=82%, list=44%, signal=46%
ST_ADRENER	ST_ADRENER	37	0.39381877	1.24332426	0.16544118	0.27898384	0.16759795		3195	tags=22%, list=9%, signal=20%
WP_CHOLEST	WP_CHOLEST	46	-0.3506359	-1.2222893	0.16556291	0.27899112	0.16760232		10537	tags=50%, list=29%, signal=36%
REACTOME_S	REACTOME_S	53	0.36091945	1.21774146	0.16928447	0.28506017	0.17124827		8927	tags=38%, list=24%, signal=29%
WP_PURINE_	WP_PURINE_	13	-0.5062016	-1.2813835	0.17068273	0.28721117	0.17254047		14042	tags=77%, list=38%, signal=48%
REACTOME_I	REACTOME_I	16	-0.468841	-1.2622062	0.17142857	0.28826206	0.17317178		18035	tags=81%, list=49%, signal=42%
WP_TYPE_I_I	WP_TYPE_I_I	29	0.41814852	1.26496428	0.17196262	0.28895557	0.17358841		11715	tags=59%, list=32%, signal=40%
REACTOME_S	REACTOME_S	74	0.33546384	1.20674552	0.17241379	0.28950896	0.17392085		9651	tags=35%, list=26%, signal=26%
WP_PYRIMID	WP_PYRIMID	84	-0.29886	-1.1615162	0.17339667	0.29095374	0.1747888		16407	tags=65%, list=45%, signal=36%
REACTOME_S	REACTOME_S	43	0.38281376	1.23493499	0.17367459	0.29120324	0.17493868		3449	tags=14%, list=9%, signal=13%
WP_HEMATC	WP_HEMATC	20	0.45136273	1.25196371	0.17382813	0.29120324	0.17493868		11287	tags=50%, list=31%, signal=35%
REACTOME_S	REACTOME_S	110	-0.2846323	-1.1662194	0.17391304	0.29120324	0.17493868		11942	tags=46%, list=32%, signal=31%
REACTOME_/	REACTOME_/	17	0.48419776	1.28490656	0.17588933	0.29424409	0.17676545		3600	tags=24%, list=10%, signal=21%
BIOCARTA_IN	BIOCARTA_IN	23	-0.4181878	-1.2551245	0.17610063	0.29424409	0.17676545		3352	tags=35%, list=9%, signal=32%
REACTOME_/	REACTOME_/	27	-0.3955378	-1.2295829	0.17610063	0.29424409	0.17676545		6433	tags=37%, list=17%, signal=31%
REACTOME_I	REACTOME_I	10	0.54650174	1.27996709	0.17682927	0.29525393	0.17737211		8131	tags=80%, list=22%, signal=62%
REACTOME_I	REACTOME_I	21	0.44168215	1.2419839	0.17726397	0.2957719	0.17768328		5782	tags=29%, list=16%, signal=24%
REACTOME_I	REACTOME_I	65	-0.3182251	-1.1824955	0.17798595	0.2967204	0.17825308		14520	tags=58%, list=39%, signal=35%
REACTOME_(	REACTOME_(	11	-0.5417356	-1.294754	0.17808219	0.2967204	0.17825308		2882	tags=27%, list=8%, signal=25%
REACTOME_I	REACTOME_I	13	-0.5037302	-1.2751276	0.17871486	0.29756588	0.178761		13742	tags=62%, list=37%, signal=39%
PID_TOLL_EN	PID_TOLL_EN	24	0.43539512	1.25470099	0.17898833	0.29781251	0.17890916		3600	tags=29%, list=10%, signal=26%
